# Chemical and Molecular Insights into the Arid Wild Plant Diversity of Saudi Arabia

**DOI:** 10.3390/plants15020295

**Published:** 2026-01-19

**Authors:** Najla A. Al Shaye

**Affiliations:** Department of Biology, College of Science, Princess Nourah Bint Abdulrahman University, P.O. Box 84428, Riyadh 11671, Saudi Arabia; naaalshaye@pnu.edu.sa

**Keywords:** desert plants, molecular identification, GC–MS profiling, arid adaptation, bioactive compounds, Saudi Arabia flora, ITS and rbcL barcoding, green bioeconomy

## Abstract

Arid and semi-arid ecosystems harbor a wealth of underexplored plant biodiversity with untapped ecological and pharmacological potential. This study integrates morphological and molecular barcoding (ITS and rbcL) to confirm the identity of eight wild plant species native to the Saudi Arabian desert: *Calligonum crinitum*, *Tribulus terrestris*, *Cornulaca monacantha*, *Cleome pallida*, *Leptadenia pyrotechnica*, *Cyperus conglomeratus*, *Indigofera argentea*, and *Artemisia monosperma*. High-resolution GC–MS analysis identified over 25 bioactive compounds across these taxa, grouped into functional classes including hydrocarbons, esters, fatty acids, quinones, terpenoids, and phenolics. Notable compounds such as n-hexadecanoic acid, 2,4-di-tert-butylphenol, lupeol, and D-limonene were linked to antioxidant activity, desiccation tolerance, and membrane protection under stress. *L. pyrotechnica* and *A. monosperma* emerged as chemical outliers with unique metabolite profiles, suggesting divergent strategies for climate resilience. Our results highlight the ecological and bioeconomic value of desert flora, positioning them as candidates for future research in metabolic engineering, dryland restoration, and plant-based pharmaceuticals. This integrative approach underscores the relevance of desert plants for sustainable development in the face of climate change.

## 1. Introduction

Wild medicinal plants have historically played a pivotal role in traditional healthcare systems and folk medicine remedies, offering a reservoir of bioactive compounds that contribute to the treatment and prevention of diverse diseases [1,2]. In arid and subtropical regions such as the Arabian Peninsula, wild flora represents a unique source of phytochemicals with potential therapeutic applications, yet its diversity remains underexplored and often underutilized [3]. Comprehensive identification and characterization of these species is therefore crucial, not only for their pharmacological potential but also for their conservation and sustainable use.

DNA barcoding has emerged as a reliable molecular tool to accurately identify plant species using short, standardized regions of the genome [4]. Among the most widely applied loci are the Internal Transcribed Spacer (ITS) and the ribulose-1,5-bisphosphate carboxylase large subunit (*rbc*L), which have demonstrated strong resolution in distinguishing closely related taxa [5,6,7,8,9]. For example, Hollingsworth et al. [4] established a global DNA barcode for land plants by combining *rbc*L with *mat*K, significantly advancing taxonomy and species authentication. Likewise, ITS2 has proven particularly effective for the authentication of medicinal plants in pharmacopeial contexts [10]. Such approaches reduce the risk of misidentification, a recurrent problem in traditional medicine that can lead to therapeutic failure or toxic side effects [11].

The integration of molecular data with chemical profiling is a particularly powerful approach. Gas Chromatography–Mass Spectrometry (GC-MS) enables the identification of volatile and semi-volatile metabolites that often underpin the medicinal properties of plants [12,13]. Studies on Saudi Arabian taxa have shown that such compounds vary substantially across species, sexes, and even ecological gradients, revealing important clues about their bioactivity and adaptive strategies [14,15]. GC-MS analyses not only aid in validating traditional uses of plants but also contribute to the discovery of novel compounds with antimicrobial, antioxidant, or anticancer potential [16,17].

Arid ecosystems, such as those of Saudi Arabia, present unique opportunities and challenges. Precise taxonomic identification of wild plants in these habitats contributes to biodiversity conservation, helps detect and protect rare or endangered species, and ensures sustainable harvesting of medicinal taxa [18]. Furthermore, understanding the genetic diversity of desert flora sheds light on adaptive responses to extreme environmental conditions, knowledge that is increasingly relevant under global climate change scenarios [19].

Against this background, the present study applies an integrated framework combining DNA barcoding (ITS and *rbc*L) with GC-MS chemical profiling to wild medicinal plants from suburban areas of Saudi Arabia. The objectives are: (i) to achieve precise taxonomic identification using molecular markers; (ii) to characterize non-polar bioactive compounds through GC-MS; and (iii) to develop a combined genetic and chemical database of these taxa. This dual approach enhances the accuracy of plant authentication while providing new perspectives on phytochemical diversity.

Despite extensive phytochemical and molecular studies on desert plants, comparative frameworks that simultaneously integrate taxonomic validation and metabolite profiling across multiple wild arid species remain scarce, particularly for the flora of Saudi Arabia. In this context, the present study introduces a comparative chemical–genetic survey in which morphologically identified desert plants are authenticated using dual DNA barcodes and analyzed under a unified GC–MS pipeline. This design enables direct cross-species evaluation of chemical diversity within a shared ecological setting, providing a new baseline for investigating adaptive traits and bioresource potential in arid-land flora. By bridging traditional botanical knowledge with molecular systematics and phytochemistry, this study contributes to pharmacognosy, conservation biology, and biotechnology, supporting biodiversity preservation, sustainable resource management, and future bioeconomic applications.

## 2. Results

### 2.1. Morphological Identification

**Figure 1:**
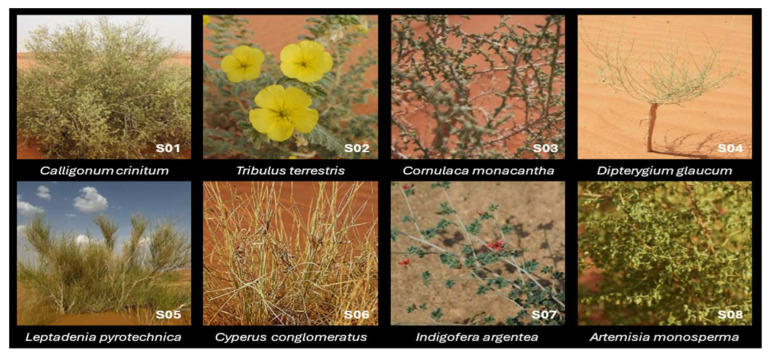
Representative images of the eight plant species collected and morphologically identified in this study. The specimens, shown from left to right and top to bottom, include *Calligonum crinitum* ssp. *arabicum* (S01), *Tribulus terrestris* (S02), *Cornulaca monacantha* (S03), *Cleome pallida* (S04), *Leptadenia pyrotechnica* (S05), *Cyperus conglomeratus* (S06), *Indigofera argentea* (S07), and *Artemisia monosperma* (S08). All plant samples were collected from arid and semi-arid habitats in the southern and northern regions of Saudi Arabia and identified based on detailed morphological characteristics using regional floras and references.

Field observations and classical morphological examination confirmed the identification of eight plant species from arid and semi-arid regions of Saudi Arabia, each belonging to a distinct botanical family (Figure 1). These species exhibit diverse adaptations to harsh environmental conditions, such as xeromorphic features, spinescence, reduced leaves, and extensive root systems.

From S01 to S08 in the same collection order: *Calligonum crinitum subsp. arabicum* (Soskov) Soskov (Polygonaceae) is a thick-stemmed, much-branched shrub reaching up to 2 m in height. Its young branches are green and slender, with deciduous leaves. The plant bears narrow elliptical fruits with bristles arising from four longitudinal ridges, contributing to its adaptation to wind dispersal in sandy habitats. *Tribulus terrestris* L. (Zygophyllaceae) is an annual prostrate herb with spreading branches and compound leaves bearing multiple pairs of pubescent leaflets. It produces small yellow flowers and spineless fruits (mericarps), distinguishing this variety from the spiny forms more commonly encountered.

*Cornulaca monacantha* Delile (Amaranthaceae) is a low-growing, heavily grazed perennial shrub with triquetrous, spine-tipped leaves and dense axillary wooly tufts. Its clustered axillary flowers and spiny perianth segments represent important xeromorphic adaptations for survival in gravelly soils. *Cleome pallida* Kotschy, (syn. *Dipterygium glaucum* Decne. Capparaceae) is a perennial, much-branched, sparsely glandular herb with reduced linear-oblong leaves and small yellow flowers in terminal racemes. The indehiscent winged fruits and glandular surfaces are characteristic features of this species in sandy and stony deserts. *Leptadenia pyrotechnica* Decne. (Apocynaceae) is a tall, leafless, spinescent shrub with terete, green stems and axillary, greenish-yellow flowers. This species is notable for its fiber-rich stems, often used in traditional ropemaking, and for its remarkable drought resistance.

*Cyperus conglomeratus* Rottb (Cyperaceae) is a perennial sedge forming dense tufts. It possesses terete, rigid culms and narrow, crescent-shaped leaves. Its capitate inflorescences are composed of densely packed spikelets, making it well-adapted to sand dune and desert environments. *Indigofera argentea* Burm. f. (Fabaceae) is a small pubescent undershrub with imparipinnate leaves and obovate leaflets. The axillary racemes bear red flowers and produce slender, cylindrical pods. This species is widespread in the southwestern Arabian Peninsula and eastern Africa.

*Artemisia monosperma* Delile (Asteraceae) is a perennial aromatic shrub with deeply dissected, linear-lobed leaves and small, ovoid capitula arranged in terminal panicles. Its achene-type fruits and silvery-green foliage reflect adaptations to sandy environments and high solar radiation. These morphological identifications were supported by expert-based keys and floristic references [3,20] and serve as the foundation for subsequent molecular and phytochemical analyses.

### 2.2. Molecular Identification

To verify species identity, ITS and *rbc*L regions were amplified from the eight selected samples and compared against NCBI GenBank and BOLD Systems databases. In most cases, the molecular results were consistent with morphological identification, offering high confidence in species assignment. For example, *Tribulus terrestris* (S02), *Cleome pallida* (S04), *Leptadenia pyrotechnica* (S05), *Cyperus conglomeratus* (S06), and *Indigofera argentea* (S07) were all confirmed at the species level through strong matches (>98% similarity) in both ITS and *rbc*L loci using NCBI. In these cases, the top molecular hits aligned fully with the morphological assessments (Table 1).

Some samples presented partial or incongruent molecular matches, necessitating deeper cross-validation. *Calligonum crinitum* ssp. *arabicum* (S01) matched *Calligonum squarrosum* in NCBI and *Calligonum polygonoides* in BOLD. Given the close morphological and taxonomic proximity among *Calligonum* species in Arabian deserts, the identification was retained based on morphology and regional distribution. Similarly, *Cornulaca monacantha* (S03) returned a top *rbc*L hit to the unrelated genus *Halogeton*, but the ITS match aligned with the expected genus, supporting the hypothesis that database limitations were responsible for this discrepancy. Finally, *Artemisia monosperma* (S08) showed molecular similarity to multiple *Artemisia* species, such as *A. japonica*, *A. dracunculus*, and *A. frigida*, indicating the presence of closely related taxa in databases rather than a precise match. However, its unique leaf morphology and regional adaptation clearly supported its identification as *A. monosperma*. These results highlight the importance of combining molecular and morphological data to overcome database limitations and confidently delimit species in underrepresented flora (Table 1).

### 2.3. Medicinal and Bioactive Compounds

#### 2.3.1. General Compound Class Distribution

**Figure 2:**
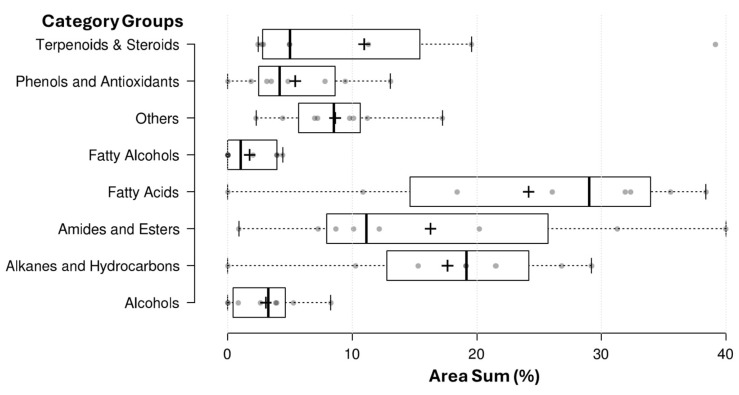
A boxplot summary of major compound classes detected across the eight analyzed plant species. The eight groups include: Alcohols, Alkanes and Hydrocarbons, Amides and Esters, Fatty Acids, Fatty Alcohols, Others (including alkenes, piperidine derivatives, quinones, and fluorophores), Phenols and Antioxidants, and Terpenoids and Steroids. Each box represents the interquartile range (IQR), with the horizontal line indicating the median, and whiskers extending to the lowest and highest values within 1.5 × IQR.

A total of 71 distinct phytochemical compounds were identified across the eight examined species (Supplementary Table S1), and their distribution among the major compound classes is summarized below. Boxplot analysis of the eight major compound classes revealed distinct patterns in abundance and distribution across the analyzed plant samples (Figure 1). Among the compound groups, fatty acids exhibited the highest overall abundance, with a median value of 28.99 and an upper whisker extending to 38.40, reflecting their broad presence and potential roles in membrane structure and stress resilience. Alkanes and hydrocarbons followed with a median of 19.13 and a wide range up to 29.20, indicating their common occurrence but highly variable accumulation, likely reflecting wax or cuticle-associated metabolic profiles. Amides and esters also showed strong representation, with a median of 11.12 and values extending to 40.00, suggesting their contribution to both primary and specialized metabolism across species. Phenols and antioxidants displayed moderate accumulation (median 4.16, max 13.05), pointing to species-dependent antioxidant profiles. Similarly, terpenoids and steroids exhibited a median of 4.96 with an upper whisker of 19.57, consistent with the sporadic but notable presence of phytosterols and triterpenoids in select species. Alcohols and fatty alcohols showed lower medians (3.22 and 1.01, respectively), with some samples displaying moderate enrichment, possibly linked to cuticular or volatile biosynthesis. Lastly, the others category (including alkenes, piperidine derivatives, quinones, and fluorophores) revealed a compressed but measurable distribution (median 8.48), highlighting the selective biosynthesis of these chemically diverse metabolites. Together, the distribution patterns reinforce a dominant biochemical core of lipids and hydrocarbons, with layered contributions from secondary metabolites that vary among species and are likely to contribute to ecological adaptation (see Figure 2).

#### 2.3.2. Clustering and Principal Component Analysis

**Figure 3:**
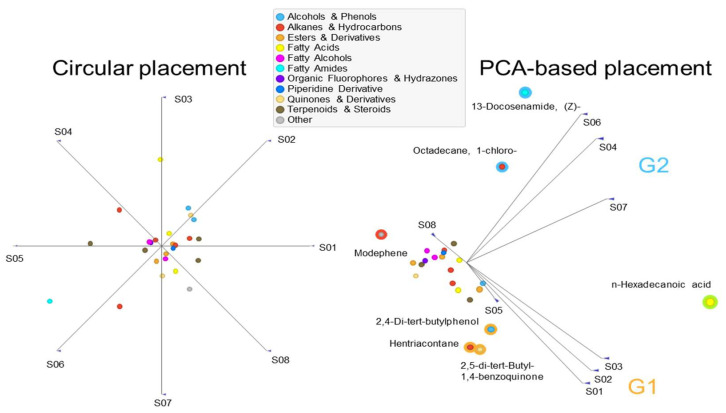
The corresponding FreeViz projection provides a visual summary of compound distribution across species. (**Left**), samples were initially arranged in a circular layout with equal spacing to reflect an unbiased start. (**Right**), PCA-based projection arranged the samples based on their chemical profiles, showing clear separation between Group 1 (G1: *C. crinitum* [S01], *T. terrestris* [S02], *C. monacantha* [S03]) and Group 2 (G2: *C. pallida* [S04], *C. conglomeratus* [S06], *I. argentea* [S07]), while *L. pyrotechnica* [S05] and *A. monosperma* [S08] appeared as outliers. Those compounds most associated with G1 and G2 are highlighted with group-specific outlines.

To explore the chemical relatedness among the eight analyzed plant species, multivariate clustering and principal component analysis (PCA) were performed based on the relative abundance of identified compounds. The FreeViz projection (Figure 3) initially positioned all samples at equidistant points in a circular layout (left panel), reflecting an unbiased input structure. When projected onto the principal component space (right panel), the samples clearly separated into two major chemical groups: Group 1 (G1), composed of *C. crinitum*, *T. terrestris*, and *C. monacantha*, and Group 2 (G2), consisting of *C. pallida*, *C. conglomeratus*, and *I. argentea*. The remaining two species, *L. pyrotechnica* and *A. monosperma*, were positioned away from both clusters, indicating their distinct metabolite profiles. PCA also enabled the identification of key compounds that contributed most to the observed clustering. The most abundant and broadly distributed compound, *n*-hexadecanoic acid, was among the top drivers in both groups. Other compounds showed stronger group-specific associations, supporting the chemical distinctiveness of G1 and G2. Additionally, the unique presence of the alkene modephene in *A. monosperma* explains its clear separation from all other species.

#### 2.3.3. Specific Profiles and Bioactive Compounds per Category

**Figure 4:**
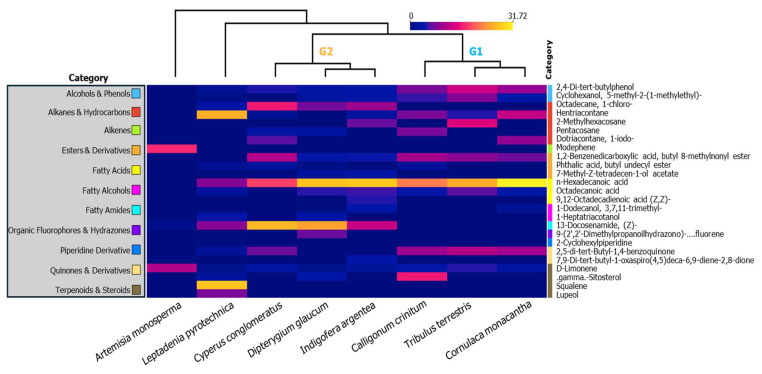
Heatmap representing the relative abundance (as area sum %) of 38 fully resolved and confidently identified compounds grouped into 8 major chemical categories across the eight studied desert plant species. Compounds are color-coded by functional class. Clustering was based on Ward’s method and standardized compound abundances. Color intensity corresponds to normalized abundance from low (blue) to high (yellow).

To better understand the chemical differentiation across species, results are organized by compound category, highlighting both dominant and rare metabolites and their association with species groups. These patterns are visually represented in the comprehensive heatmap and further supported by the PCA projection (Figure 4), which together confirmed the clustering of Group 1 and Group 2, while identifying *A. monosperma* and *L. pyrotechnica* as chemically distinct outliers.

Although a larger number of chromatographic peaks were detected, 71 compounds were confidently identified across the eight species, as detailed in Supplementary Table S1. Of these, 38 compounds were retained for the comparative heatmap analysis, as they were consistently detected, fully resolved, and quantifiable across multiple species. Peaks that were present at trace levels, showed poor resolution, or appeared only in a single species were excluded to avoid misinterpretation. A complete list of both identified and unidentified peaks is now provided in the Supplementary Table S1. As follows, the compounds are discussed by functional category, highlighting their distribution, abundance, and potential ecological or biochemical significance across the studied species:

Fatty Acids. Fatty acids were the most abundant compound group across all samples. *n*-Hexadecanoic acid (palmitic acid) dominated the profile, detected in all species except *A. monosperma*, with the highest level in *C. monacantha* and substantial amounts in *C. pallida* and *I. argentea*. Octadecanoic acid (stearic acid) was moderately distributed and shared between both clusters, absent only in *A. monosperma* and *C. conglomeratus*. Linoleic acid (9,12-octadecadienoic acid) appeared exclusively in *I. argentea*, distinguishing it within G2 and suggesting a role in unsaturated lipid-mediated stress adaptation.

Alkanes and Hydrocarbons. Hentriacontane was the most frequently detected hydrocarbon, showing maximum levels in *L. pyrotechnica*, which, like *A. monosperma*, emerged as a secondary outlier. Other widespread hydrocarbons included octadecane, 1-chloro- and pentacosane, variably distributed among both groups. 2-Methylhexacosane was rare, detected only in *T. terrestris* and *I. argentea*, highlighting a biochemical link across groups. Notably, *C. monacantha* accumulated a unique iodinated hydrocarbon, dotriacontane, 1-iodo-, while *A. monosperma* lacked any hydrocarbons, reinforcing its distinctiveness.

Amides and Esters. 13-Docosenamide (Z) was the dominant amide, most concentrated in *C. conglomeratus* and *C. pallida*, reinforcing G2 specificity. Esters were led by 1,2-benzenedicarboxylic acid, butyl 8-methylnonyl ester, detected in nearly all samples except *A. monosperma*, with the highest abundance in *C. conglomeratus* and *C. crinitum*. More restricted esters like phthalic acid, butyl undecyl ester were confined to four species with a minor quantity compared to other compounds, while 7-methyl-Z-tetradecen-1-ol acetate appeared uniquely in *C. pallida* and *I. argentea*, again supporting G2’s unique metabolic profile.

Phenols and Antioxidants. 2,4-Di-tert-butylphenol, a potent antioxidant, was abundant in G1 species (*T. terrestris*, *C. monacantha*, *C. crinitum*), contributing to their clustered profile. Cyclohexanol, 5-methyl-2-(1-methylethyl)-, although structurally closer to an alcohol, appeared in the same taxa and supported a shared oxidative stress mitigation strategy. These compounds were absent in *A. monosperma*, and only faintly present in *C. pallida* and *L. pyrotechnica*, marking a clear chemical division.

Alcohols. The cyclohexanol derivative was the only alcohol detected, with its strongest accumulation in *T. terrestris*, *C. crinitum*, and *C. monacantha*, showing consistent expression in G1. It was absent in *C. conglomeratus* and *A. monosperma*, further emphasizing chemical divergence between clusters.

Fatty Alcohols. This class showed low abundance overall. 1-Dodecanol, 3,7,11-trimethyl- was detected in *C. monacantha* and *I. argentea*, while 1-heptatriacotanol appeared only in *C. pallida* and *L. pyrotechnica*, suggesting lineage- or function-specific accumulation patterns.

Terpenoids and Steroids. These compounds varied significantly among taxa. D-limonene was moderately abundant across groups but reached a peak in *A. monosperma*. Squalene and lupeol were exclusively found in *L. pyrotechnica*, while γ-sitosterol appeared solely in *C. crinitum*, showing a highly taxon-specific distribution and supporting both outlier status and specialized biochemical roles.

Quinones and Derivatives. 2,5-Di-tert-butyl-1,4-benzoquinone was shared across multiple samples, including all G1 species, and thus serves as a common oxidative biomarker for this group. In contrast, the spirocyclic quinone, 7,9-di-tert-butyl-1-oxaspiro(4,5)deca-6,9-diene-2,8-dione, was rare, found only in *I. argentea* and at trace levels in *A. monosperma*, further underscoring their chemical uniqueness.

Organic Fluorophores and Hydrazones. The complex hydrazone compound was uniquely detected in *C. pallida*, potentially reflecting unique environmental interaction or biosynthetic capacity within G2.

Piperidine Derivatives. Only *L. pyrotechnica* accumulated 2-cyclohexylpiperidine, reinforcing its role as a chemically distinct taxon, alongside *A. monosperma*.

Alkenes. Modephene was found exclusively in *A. monosperma* at a remarkably high level, acting as the main biomarker for this outlier and reinforcing its extreme metabolic divergence from all other taxa.

## 3. Discussion

The use of molecular markers, particularly the ITS and *rbc*L regions, has proven instrumental in accurately identifying wild plant species in arid regions like Saudi Arabia [15,21,22]. ITS offers high resolution at the species level, especially for closely related taxa, while *rbc*L provides a more conserved region useful for genus- or family-level differentiation [9]. In this study, molecular identification based on ITS and *rbc*L sequences validated eight morphologically characterized species: *C. crinitum*, *T. terrestris*, *C. monacantha*, *C. pallida*, *L. pyrotechnica*, *C. conglomeratus*, *I. argentea*, and *A. monosperma*. Species names were confirmed with high certainty when database hits matched both morphological traits and their geographic distribution.

In cases of partial or incongruent matches, the observed discrepancies were often due to database limitations or the absence of region-specific entries, leading to illogical matches with species from distant geographic areas (e.g., *Artemisia japonica, Halogeton glomeratus*). However, partial matches are not solely attributable to gaps in reference databases. In some recently evolved taxa, such as many species in Asteraceae, DNA barcoding regions may not have accumulated sufficient divergence to clearly separate species, resulting in high sequence similarity across multiple taxa even when sequences are available, as demonstrated in *Artemisia* and related genera using nrDNA ITS and ETS sequences [23]. Such outcomes highlight the necessity of integrating barcoding results with expert morphological assessments, particularly when taxonomic resolution is low or conflicting. For example, *C. monacantha* returned a conflicting *rbc*L hit to the unrelated genus *Halogeton*, while *C. crinitum* matched *C. squarrosum* in NCBI and *C. polygonoides* in BOLD—both taxa ecologically and morphologically close to the target species. *Artemisia monosperma* also aligned with multiple Eurasian species due to high sequence similarity within the genus. In all such cases, morphological traits and regional floras guided the final taxonomic decisions. These findings reinforce the importance of combining molecular and classical approaches in species identification, especially in underrepresented floras [24,25]. Furthermore, these markers facilitate phylogenetic inference, genetic purity assessment, and trait-based screening for ecological and adaptive studies [7].

The identification of bioactive compounds within plant species in Saudi Arabia not only sheds light on their chemical composition but also opens up new avenues for biochemical and pharmacological research [8]. Compounds such as terpenoids, alkaloids, and phenols, documented in this study, are known for their medicinal properties, offering potential for drug development and industrial applications [11]. This diversity not only highlights the adaptive chemistry of desert flora but also demonstrates their potential in supporting a green economy through drug discovery, bioprospecting, and bio-based industries [8,11]. The comprehensive analysis of the chemical composition across various plant samples provides significant insights into the diversity and distribution of compounds within different species, categorizing them into distinct groups based on their properties and functions [17]. The chemical analysis revealed a wide range of bioactive compounds across the eight studied species, grouped into major functional categories that reflect their ecological and pharmacological roles.

Among hydrocarbons and fatty acid derivatives, *L. pyrotechnica* was notable for high levels of hentriacontane and squalene, suggesting adaptations to high radiation and oxidative stress. The *C. monacantha* and *C. conglomeratus* also accumulated long-chain alkanes and fatty acids (e.g., *n-*hexadecanoic acid), reinforcing their role in membrane stabilization and desiccation tolerance [13,26]. In parallel, esters and amides such as 1,2-benzenedicarboxylic acid derivatives in *C. crinitum*, *C. monacantha*, and *C. conglomeratus* may indicate protective and signaling functions under stress [27].

While compounds such as phthalic acid derivatives and 13-docosenamide were detected, the literature reports that phthalates may derive from plasticware or environmental contamination, and 13-docosenamide may originate from microbial sources and mostly represented in minute amounts [28]. The *C. pallida* exhibited a notably high relative abundance of the fatty amide 13-docosenamide, possibly contributing to drought resilience. The relative abundance of n-Hexadecanoic acid in all species except for *A. monosperma* underscores its role in membrane structure and energy storage [26]. In all cases, the functional roles attributed to these compounds are discussed with appropriate caution and framed as putative, literature-informed interpretations rather than definitive biological functions, acknowledging that further targeted assays are required for confirmation.

The phenol and alcohol, represented by 2,4-di-tert-butylphenol and cyclohexanol derivatives, respectively, were abundant in *T. terrestris*, *C. crinitum*, and *C. monacantha*, known for their antioxidant and antimicrobial activities [19]. 2,4-Di-tert-butylphenol, although sometimes considered synthetic, has been documented as a natural constituent in various plant species. Its consistent detection in our samples supports its biological relevance. Moreover, 2,4-DTBP commonly occurs in the volatile or essential oils of many seed-plant species and has been reported in at least 169 species across both dicots and monocots [29].

The *A. monosperma* was unique in accumulating modephene, a hydrocarbon alkene potentially involved in chemical signaling or allelopathy. This compound, along with other phenolics, may contribute to interspecific interactions in resource-limited environments [30]. Fatty Alcohols and Amides, such as 1-Dodecanol, 3,7,11-trimethyl- in *I. argentea* only, suggest specific biosynthetic pathways in these species [31].

The terpene and steroid profile varied across species. *A. monosperma* exhibited the highest level of D-limonene, a monoterpene with strong ecological and medicinal relevance. In contrast, *L. pyrotechnica* showed high squalene and lupeol levels, both linked to membrane function and pharmacological activity [32]. The presence of γ-sitosterol in *C. crinitum* and lupeol in *L. pyrotechnica* further highlights their potential in anti-inflammatory and anti-cancer applications.

Quinones, such as 2,5-di-tert-butyl-1,4-benzoquinone, were most abundant in *C. crinitum*, *T. terrestris*, and *C. monacantha*, supporting a role in oxidative defense and longevity under stress [1]. Notably, *C. monacantha* and *C. pallida* featured derivatives such as 7,9-di-tert-butyl-1-oxaspiro-dione, potentially linked to pigmentation and defense. *Cleome pallida* also harbored organic fluorophores like 9-(2′,2′-Dimethylpropanoilhydrazono)-3,6-dichloro-2,7-bis-[2-(diethylamino)-ethoxy]fluorene, indicating rare photoprotective traits possibly related to UV exposure or chemical communication [33], reinforcing the ecological relevance of these secondary metabolites in arid environments. The occurrence of piperidine derivatives in *L. pyrotechnica*, and minor alcohols and esters in various taxa, suggests less characterized but ecologically relevant pathways. These rare compounds warrant further exploration, particularly in the context of climate-resilient agriculture and ecological restoration [14,31].

Altogether, *A. monosperma* and *L. pyrotechnica* emerged as chemical outliers with distinct profiles, as supported by both heatmap clustering and PCA. Their unique compositions suggest divergent adaptive strategies to extreme environments, reinforcing their potential as candidate species for functional genomics, metabolic engineering, or dryland agroecosystem design. These biochemical profiles not only illuminate ecological adaptations but also hold translational value for biotechnological and pharmacological pursuits [16].

The chemical profiles observed in our GC–MS analysis broadly align with previous reports for several species, confirming both shared metabolites and lineage-specific compounds. In *T. terrestris*, studies detected Fatty Acids, Terpenoids, Amides and Esters, Alcohols, Phenols, Quinones, and Alkanes, with n-hexadecanoic acid, octadecanoic acid, and D-limonene matching compounds reported in previous GC-MS studies [34]. *Cleome pallida* contained Fatty Acids, Terpenoids and Steroids, and Alkanes and Hydrocarbons such as n-hexadecanoic acid and squalene, in agreement with previous data [35]. Similarly, *L. pyrotechnica* exhibited hydrocarbons, fatty acids, terpenoids, and sterols, matching the diversity we detected by Radwan et al. [36]. For *C. crinitum*, *C. monacantha*, and *I. argentea*, our results expand phytochemical knowledge, revealing a broad spectrum across Fatty Acids, Alkanes and Hydrocarbons, Amides and Esters, Phenols and Antioxidants, Alcohols, Fatty Alcohols, Terpenoids and Steroids, Quinones and Derivatives, Organic Fluorophores and Hydrazones, Piperidine Derivatives, and Alkenes. In *A. monosperma*, our findings highlight the presence of sesquiterpenes and acetylenes, including caryophyllene, cadina-1(10),6,8-triene, humulenol-II, 5,8,11-heptadecatriynoic acid methyl ester, and 3-methyl-3-phenyl-1,4-pentadiyne, consistent with previous reports on the plant’s terpenoid and acetylene composition [37,38]. These comparisons validate the ecological and biochemical relevance of core metabolites and highlight novel components in less-studied desert taxa.

The novelty of this study lies in the comparative integration of taxonomic validation and non-polar metabolite profiling across multiple wild desert plant species using a single, standardized analytical framework. Unlike previous studies that addressed molecular identification or phytochemistry separately, this work links DNA-authenticated species identities with directly comparable GC–MS profiles, enabling cross-species evaluation of chemical diversity within arid environments, which has not been previously reported for wild desert flora of Saudi Arabia.

In broader terms, this study contributes to climate resilience and the green economy by highlighting native plant resources with proven or potential applications in sustainable land use, medicinal chemistry, and bio-based industries. The identification of pharmacologically active compounds, along with their ecological implications, supports the valorization of desert flora not only for conservation but also for economic and therapeutic innovation [8,17,39].

## 4. Materials and Methods

### 4.1. Plant Collection and Morphological Identification

A total of eight wild plant species were collected from three distinct arid regions across the Kingdom of Saudi Arabia during targeted field surveys conducted in 2024. The sampling sites spanned the Rubʿ al-Khālī Margin (Najran Province), the Al-Līth Road (Jeddah), and the Northern Nūfūd Dunes (Al-Hai’l). Geographic coordinates of each site were recorded using a GPS device (Garmin eTrex 32x, Garmin, Schaffhausen, Switzerland), and preliminary species identification was conducted in the field based on characteristic vegetative and reproductive traits (Table 2).

Morphological identification was verified using regional floras with certainty down to the genus and/or species level using standard botanical keys and taxonomic references, including Flora of the Kingdom of Saudi Arabia [40], Flora of Egypt [20], and Wild Flowers of Saudi Arabia [3]. Diagnostic characters examined included growth form, branching pattern, leaf morphology, pubescence, flower structure, fruit and seed features whenever available.

The collected samples included representatives from diverse plant families typical of xeric environments of the sampled protected areas: *Calligonum crinitum* subsp. *arabicum* (Polygonaceae), *Tribulus terrestris* (Zygophyllaceae), *Cornulaca monacantha* (Amaranthaceae), *Cleome pallida* (Capparaceae), *Leptadenia pyrotechnica* (Apocynaceae), *Cyperus conglomeratus* (Cyperaceae), *Indigofera argentea* (Fabaceae), and *Artemisia monosperma* (Asteraceae). Species names were revised using Tropicos.org for recent updates. Voucher specimens were pressed and preserved according to standard herbarium protocols and deposited at the Herbarium of College of Sciences (Biology Dept.), Princess Nourah University, Ministry of Education, Saudi Arabia (PNUH), curated by the study author (Table 2).

### 4.2. DNA Extraction and Barcoding

Approximately 200 mg of dried ground plant material from each sample was used for DNA extraction. The extraction process followed a modified CTAB method [41], optimized for plant tissues. Two DNA regions, the ITS and *rbc*L, were amplified using polymerase chain reaction (PCR). The primers used for ITS amplification were ITS1 and ITS4 [42], and for *rbc*L, primers rbcL1 and rbcL2 were employed [43]. Following amplification, PCR products were purified by spin column using an EasyPure PCR Purification Kit (TransGen Biotech, Beijing, China), following the manufacturer’s instructions. The purified products were then submitted for commercial bidirectional Sanger sequencing through Macrogen Inc. (Seoul, Republic of Korea).

The obtained sequences were blasted against the NCBI nucleotide database (nr) and compared with the Barcode of Life Data System (BOLD; www.boldsystems.org) to identify the top five hits for each DNA region. These hits were refined and aligned using the software MEGA X [44], which facilitated multiple sequence alignment and revision.

### 4.3. GC-MS Analysis

The dried plant material was further analyzed for its chemical composition using Gas Chromatography-Mass Spectrometry (GC-MS). Approximately 200 mg of the ground samples were subjected to non-polar solvent extraction using chloroform. The extracts were concentrated and injected into a GC-MS system equipped with an HP-5MS column (30 m × 0.25 mm, 0.25 μm film thickness) (Agilent Technologies, Santa Clara, CA, USA). Samples were analyzed using splitless injection of 1 μL, with hydrogen as the carrier gas at a flow rate of 3.0 mL/min. The GC-MS analysis was conducted under the following conditions: the initial temperature 40 °C (held for 1 min), increased at 10 °C/min to 200 °C (held for 1 min), then at 20 °C/min to 220 °C (held for 1 min), and finally at 30 °C/min to 320 °C (held for 10 min). The injector and detector temperatures were maintained at 250 °C and 320 °C, respectively.

The mass spectrometer was operated in electron ionization mode at 70 eV, scanning from *m*/*z* 50 to 800, with a solvent delay of 4.7 min, an ion source temperature of 230 °C, and a quadrupole temperature of 150 °C. Compounds were identified by comparing their mass spectra with those in the integrated NIST library and confirmed by comparing their retention indices and mass spectra with literature data. This comprehensive GC-MS analysis allowed for the detection and identification of non-polar bioactive compounds present in the plant extracts, contributing to the understanding of their medicinal potential. Relative abundance for each compound was calculated as its percentage of the total ion chromatogram peak area.

### 4.4. Data Analysis

Data from the GC-MS analyses were integrated to provide a holistic characterization of the medicinal plant samples applying PCA and heatmaps with clustering in Orange software V3.36 [45].

## 5. Conclusions

This study demonstrates the power of combining molecular barcoding with chemical profiling to resolve plant identification and explore adaptive metabolite diversity in underrepresented desert ecosystems. Notably, it introduces a comparative chemical–genetic framework that integrates DNA barcoding with non-polar GC–MS profiling across multiple wild desert plant species within a single standardized analytical design. The eight confirmed wild species revealed distinct phytochemical fingerprints, with many compounds linked to stress tolerance, antioxidant capacity, and potential medicinal properties.

The metabolite profiles of *Leptadenia pyrotechnica* and *Artemisia monosperma*, as observed in this study, highlight their potential as model species for investigating climate adaptation and for developing sustainable applications in dryland agriculture, biotechnology, and pharmaceutical industries. While these profiles are distinct among the species analyzed here, further studies including additional taxa are needed to fully assess their comparative uniqueness. These findings contribute to regional biodiversity conservation while supporting global initiatives toward a greener bioeconomy. Further research on their biosynthetic pathways and bioactivities could unlock new resources for functional plant-based innovation in arid zones.

## Figures and Tables

**Table 1:** Molecular identification of the eight selected plant species based on ITS and rbcL sequences matched against NCBI and BOLD databases. Similarity indices (%) represent the top sequence matches per locus.

Database	NCBI				BOLD			
Locus	ITS	%	*rbc*L	%	ITS	%	*rbc*L	%
S01	*Calligonum squarrosum*	0.98	*C. squarrosum*	0.98	*C. polygonoides*	99	*C. polygonoides*	100
S02	*Tribulus terrestris*	0.94	*T. terrestris*	0.99	-	-	-	-
S03	*Cornulaca monacantha*	0.98	*Halogeton arachnoideus*	0.98	-	-	-	-
S04	*Cleome pallida*	0.99	*C. pallida*	0.99	-	-	-	-
S05	*Leptadenia pyrotechnica*	0.98	*L. pyrotechnica*	0.99	-	-	-	-
S06	*Cyperus conglomeratus*	0.96	*C. conglomeratus*	0.99	-	-	-	-
S07	*Indigofera argentea*	0.99	*I. argentea*	1.0	-	-	-	-
S08	*Artemisia japonica*	0.97	*A. dracunculus*	0.97	*A. frigida*	96	*A. dracunculus*	94.2

**Table 2:** Geographical coordinates of collection sites and corresponding taxonomic details of plant specimens sampled across the Kingdom of Saudi Arabia.

Province	Location	Latitude	Longitude	PNUH	Proposed Species Name	Family Name
Najran	Rubʿ al-Khālī Margin	19°09′05.3″ N	45°10′25.0″ E	6553	*Calligonum crinitum* ssp. *arabicum*	Polygonaceae
		19°09′07.4″ N	45°11′25.5″ E	6111	*Tribulus terrestris*	Zygophyllaceae
		19°13′05.3″ N	45°14′29.7″ E	6110	*Cornulaca monacantha*	Amaranthaceae
		19°10′22.8″ N	45°09′33.3″ E	6089-90	*Cleome pallida*	Capparaceae
		19°09′30.7″ N	45°08′50.0″ E	6041	*Leptadenia pyrotechnica*	Apocynaceae
		19°09′02.5″ N	45°11′20.1″ E	6076	*Cyperus conglomeratus*	Cyperaceae
Jeddah	Al-Līth Road	28°18′41.0″ N	35°50′46.0″ E	6123	*Indigofera argentea*	Leguminosae
Al-Hai’l	Northern Nūfūd Dunes	28°07′54.9″ N	41°38′19.6″ E	4420	*Artemisia monosperma*	Asteraceae

## Data Availability

Generated ITS and *rbc*L sequences are available at the NCBI database, accession numbers: in process of submission, and will be provided as soon as they become available.

## Supplementary Materials

The following supporting information can be downloaded at: https://www.mdpi.com/article/10.3390/plants15020295/s1, Supplementary Table S1. Phytochemical Profiles of Species in the Current Study (S01–S08) Detected by GC-MS Analysis.
