# Supplementary material for: Chemical and Molecular Insights into the Arid Wild Plant Diversity of Saudi Arabia
Source: Plants (Basel). 2026 Jan 19;15(2):295. doi: 10.3390/plants15020295 (PMC12845481; doi:10.3390/plants15020295)
Supplement: Supplementary file 1 [file plants-15-00295-s001.zip › Sample 1_AnalysisReport.pdf]

Qualitative Analysis Report

**Data Filename**	Sample 1-.D	**Sample Name**	Sample 1-
**Sample Type**		**Position**	1
**Instrument Name**	3	**User Name**	
**Acq Method**	Scan DB-5MS Hydrogen 2024.M	**Acquired Time**	6/24/2024 4:04:18 PM
**IRM Calibration Status**	Not Applicable	**DA Method**	SignalToNoiseCheckout.m
**Comment**			

**Expected Barcode**		**Sample Amount**	
**Dual Inj Vol**	0.2	**TuneName**	ATUNE.U
**TunePath**	D:\MassHunter\GCMS\3\5977 \	**TuneDateStamp**	2024-06-23T14:01:57+02:00
**MSFirmwareVersion**	6.00.34	**OperatorName**	
**RunCompletedFlag**	True	**Acquisition SW Version**	MassHunter GC/MS Acquisition 10.0.368 14-Feb-2019 Copyright © 1989-2018 Agilent Technologies, Inc

User Chromatograms

Fragmentor Voltage **Collision Energy** 0 **Ionization Mode** EI

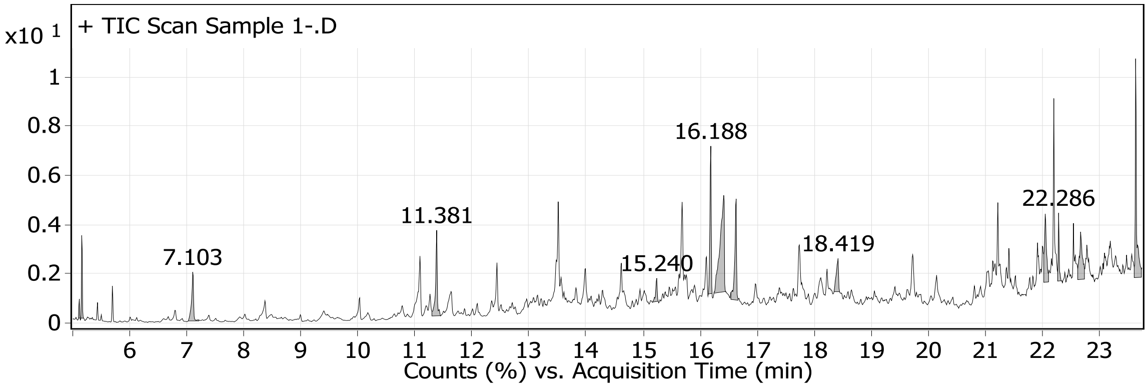

Integration Peak List

Peak	Start	RT	End	Height	Area	Area %
1	5.098	5.115	5.128	378286.46	263874.77	2.71
2	5.14	5.156	5.19	1636726.48	1655953.93	16.98
3	7.023	7.103	7.212	966486.26	2338380.82	23.97
4	11.297	11.381	11.447	1694119.01	3456162.63	35.43
5	15.167	15.24	15.265	459040.57	862947.21	8.85
6	16.144	16.188	16.21	2920675.3	4518632.87	46.32
7	16.242	16.414	16.446	1907701.35	9754552.68	100
8	16.54	16.632	16.666	2000671.27	4329226.54	44.38
9	18.347	18.419	18.447	657384.28	1799736.21	18.45
10	22.026	22.051	22.11	1354367.6	3380399.1	34.65
11	22.269	22.286	22.333	1345892.92	1546793.96	15.86
12	22.622	22.672	22.739	941218.34	3394335.44	34.8
13	23.612	23.637	23.737	4333038.78	7015049.43	71.92

User Spectra

Spectrum Source	Collision Energy	Ionization Mode
Peak (1) in "+ TIC Scan"	0	EI

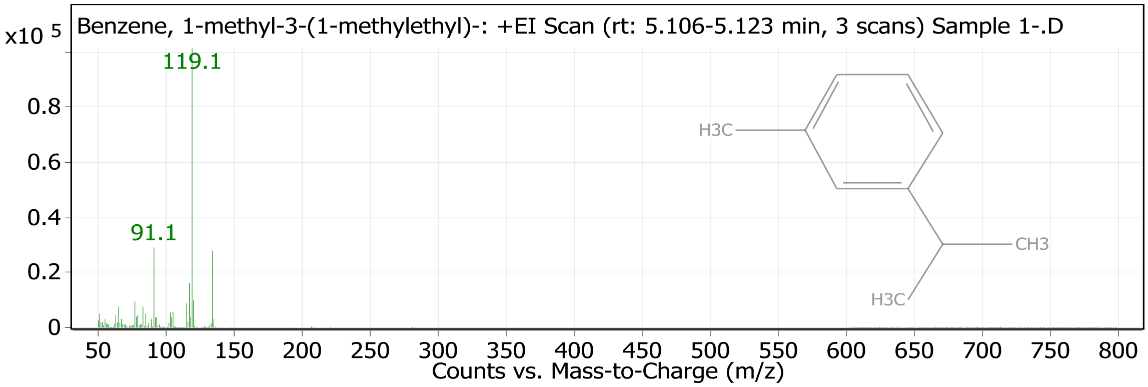

Library Spectrum

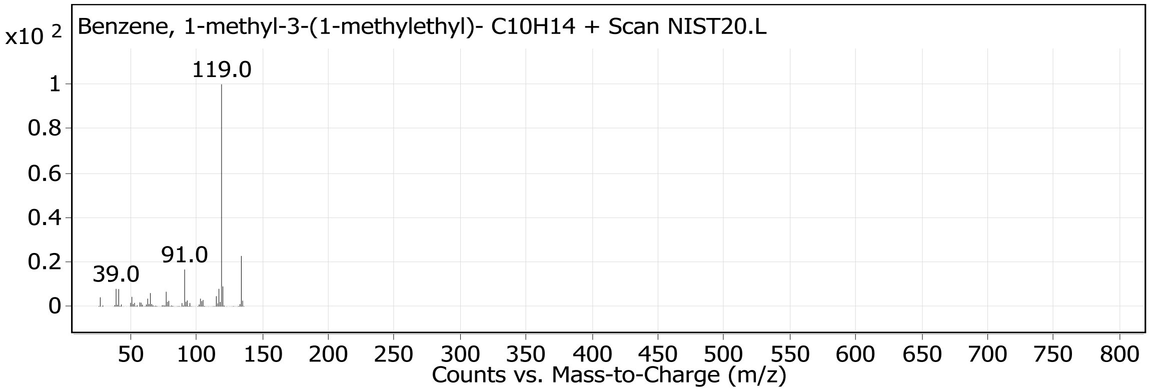

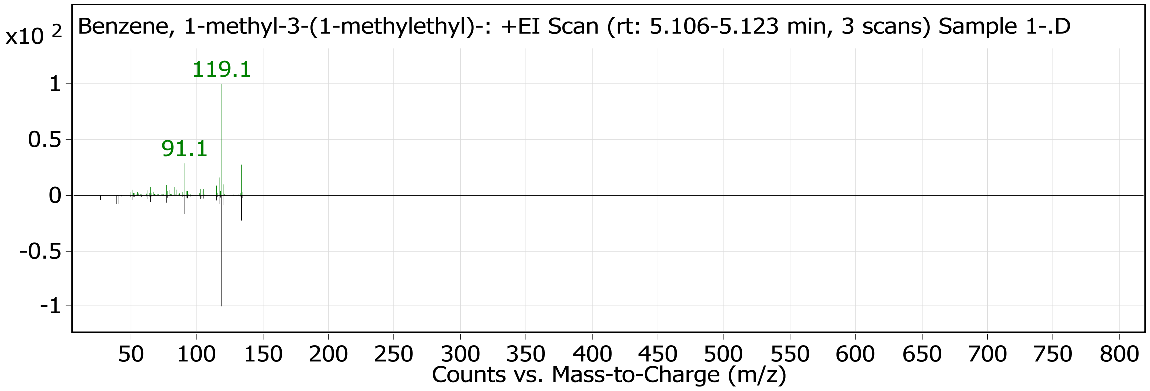

Spectrum Structure

Benzene, 1-methyl-3-(1-methylethyl)-

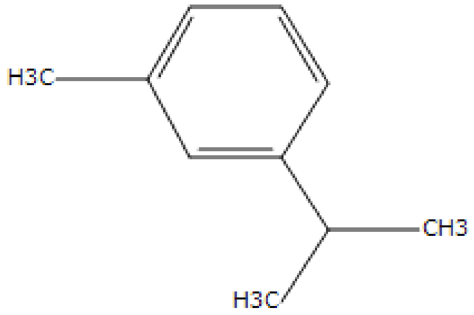

Qualitative Analysis Report

Spectrum Source	Collision Energy	Ionization Mode
Peak (2) in "+ TIC Scan"	0	EI

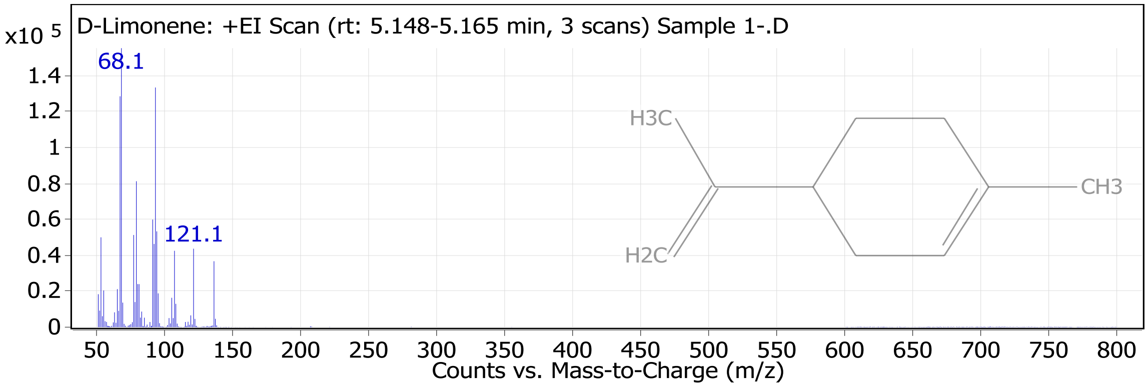

Library Spectrum

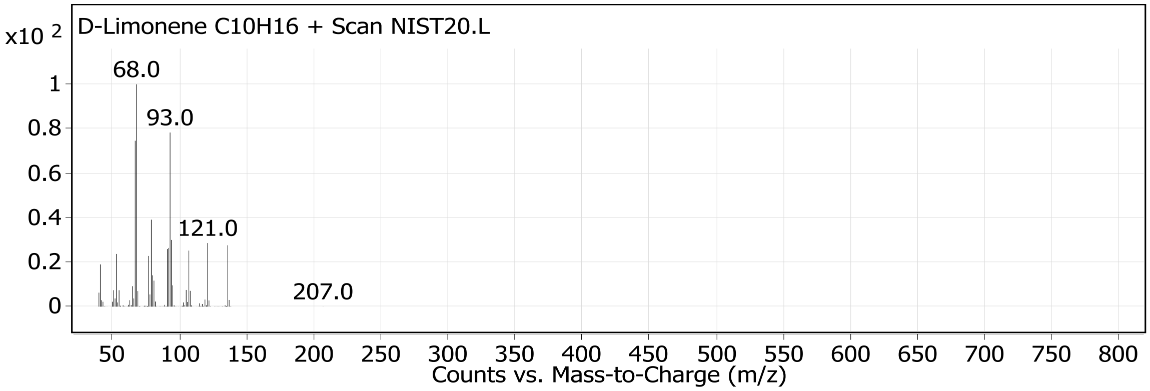

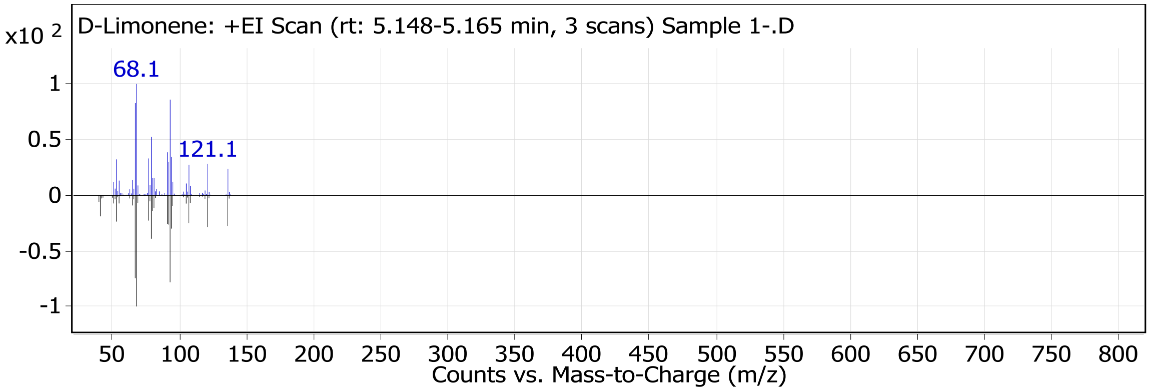

Spectrum Structure

D-Limonene

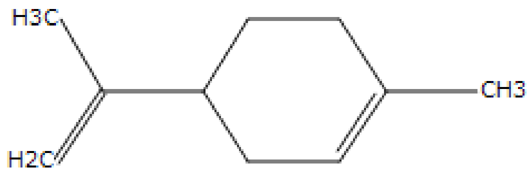

Spectrum Source	Collision Energy	Ionization Mode
Peak (3) in "+ TIC Scan"	0	EI

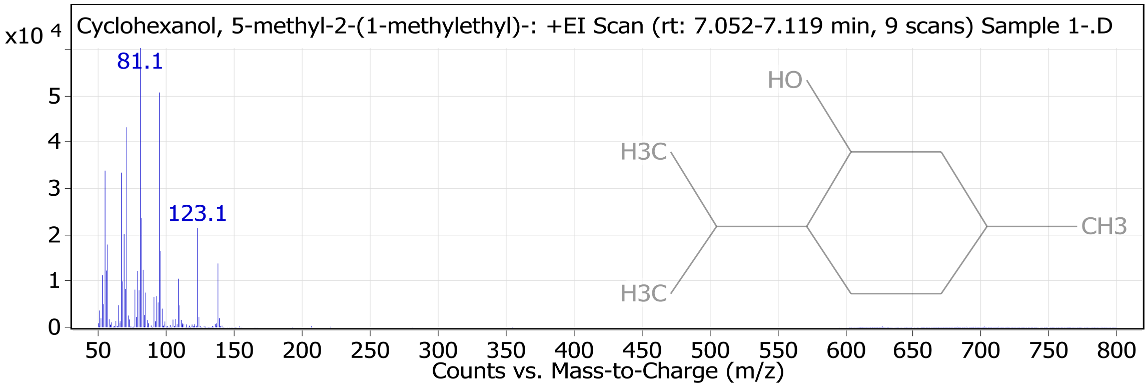

Library Spectrum

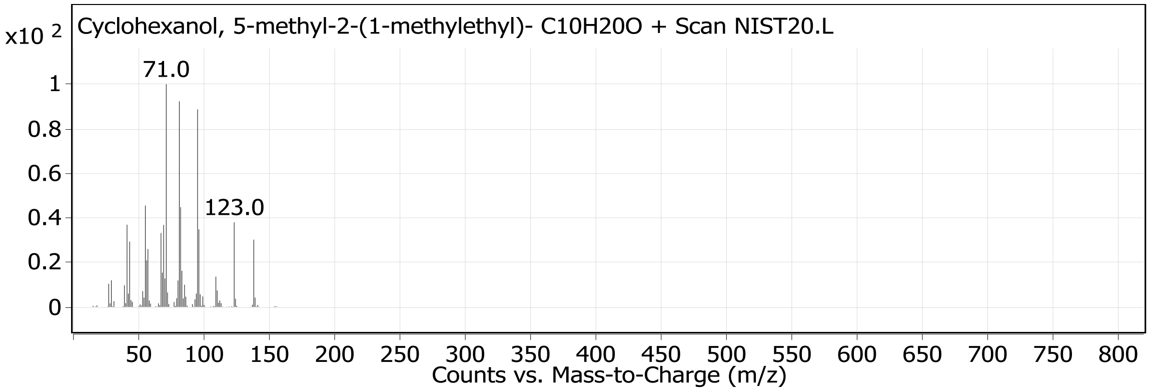

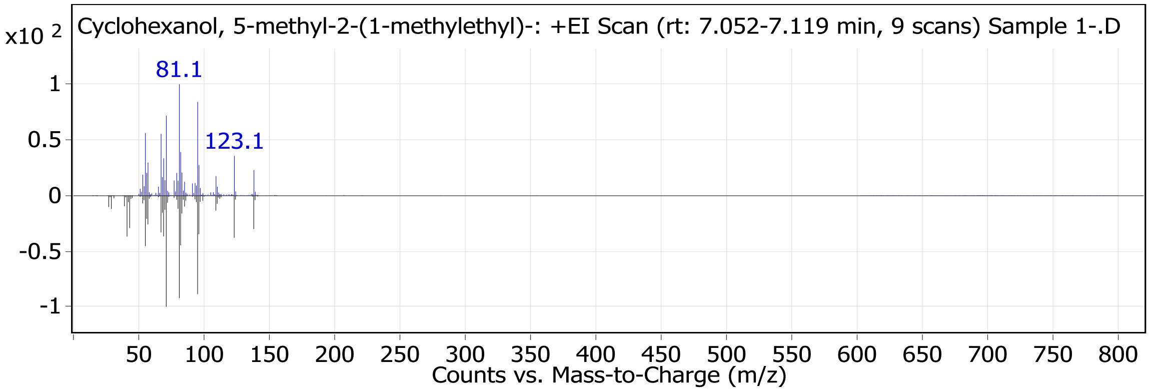

Spectrum Structure

Cyclohexanol, 5-methyl-2-(1-methylethyl)-

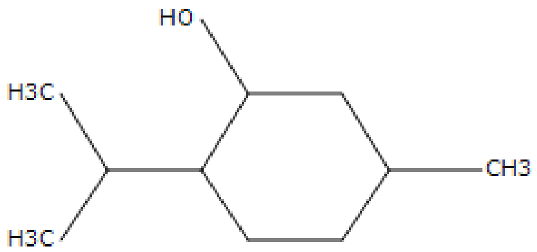

Spectrum Source	Collision Energy	Ionization Mode
Peak (4) in "+ TIC Scan"	0	EI

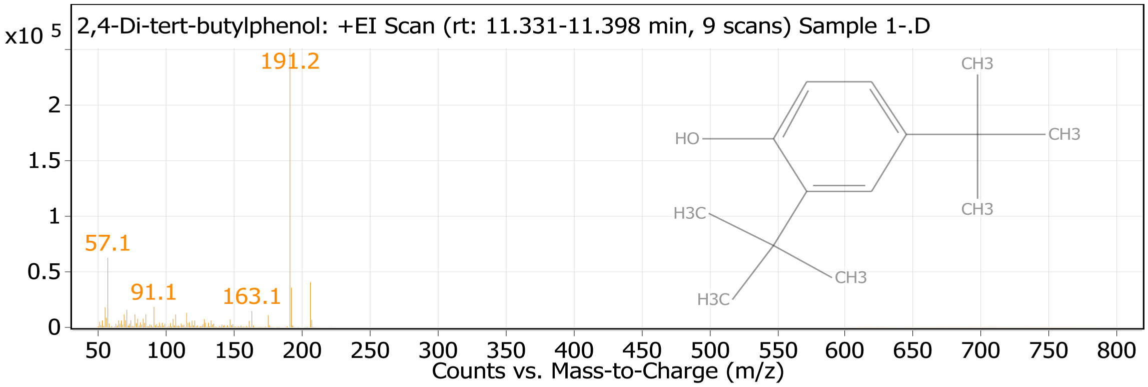

Library Spectrum

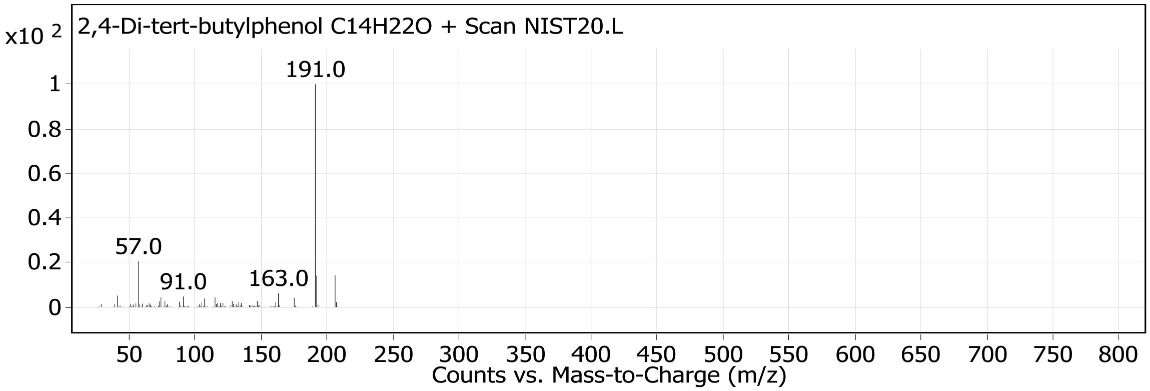

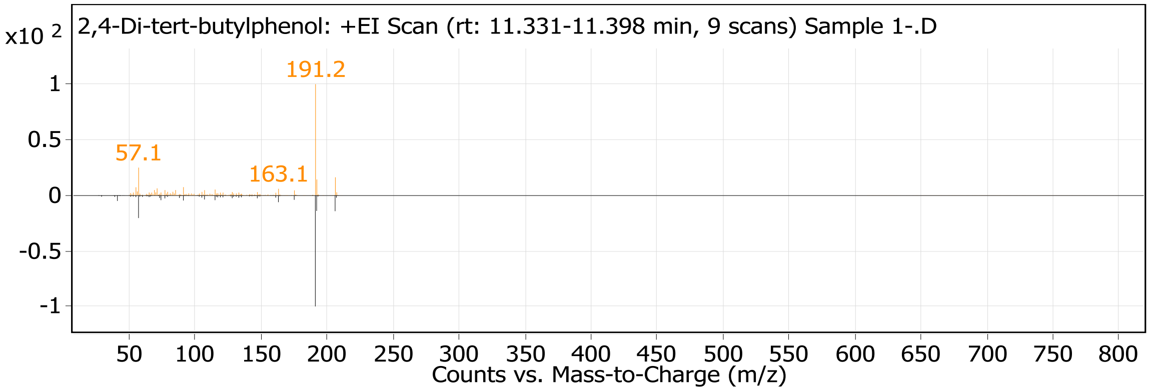

Spectrum Structure

2,4-Di-tert-butylphenol

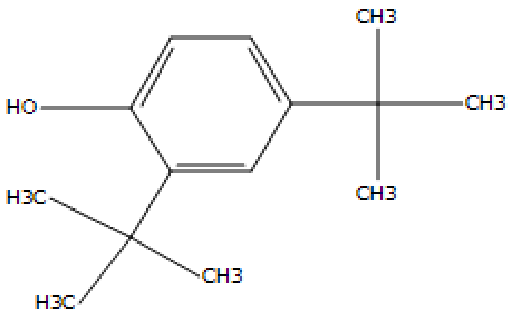

Spectrum Source	Collision Energy	Ionization Mode
Peak (5) in "+ TIC Scan"	0	EI

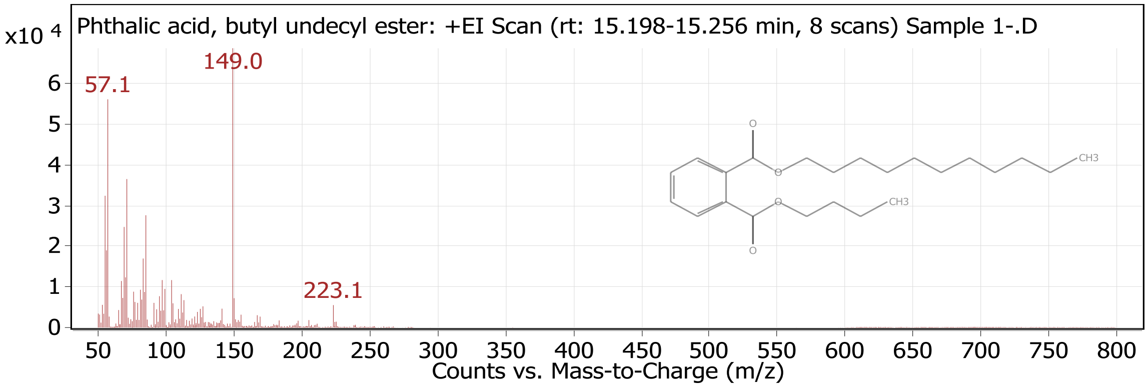

Library Spectrum

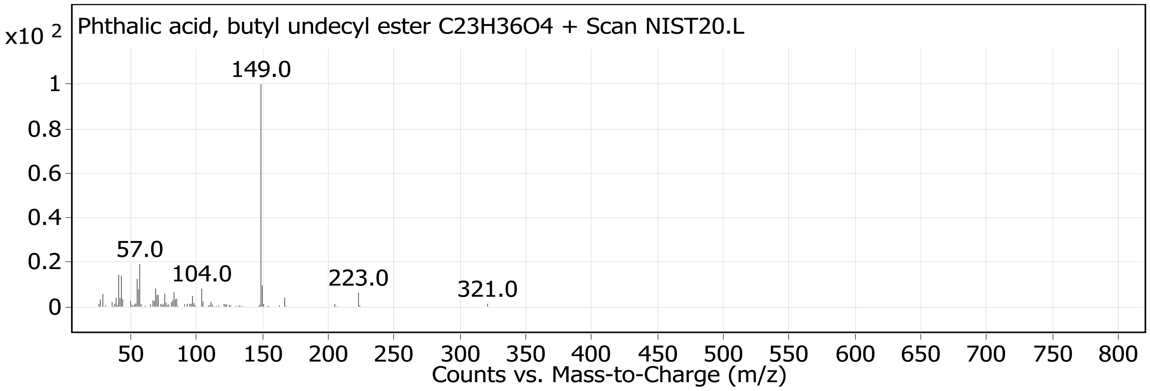

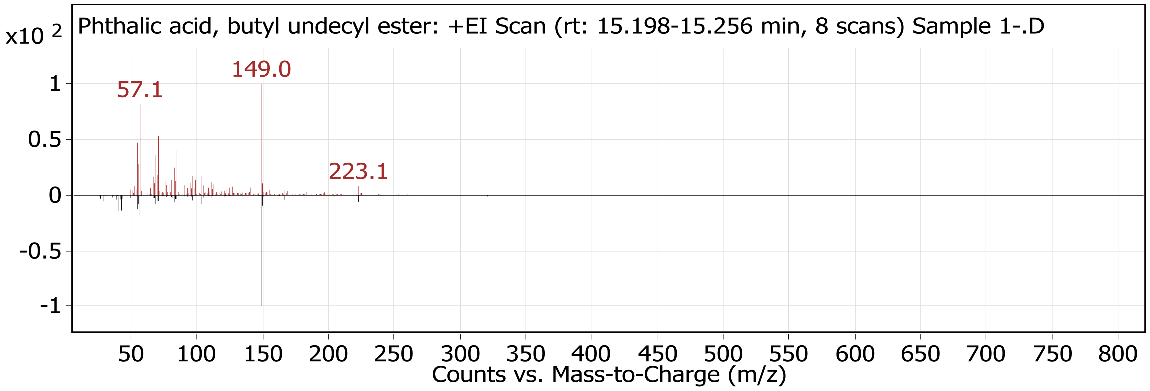

Spectrum Structure

Phthalic acid, butyl undecyl ester

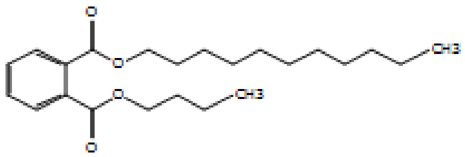

Spectrum Source	Collision Energy	Ionization Mode
Peak (6) in "+ TIC Scan"	0	EI

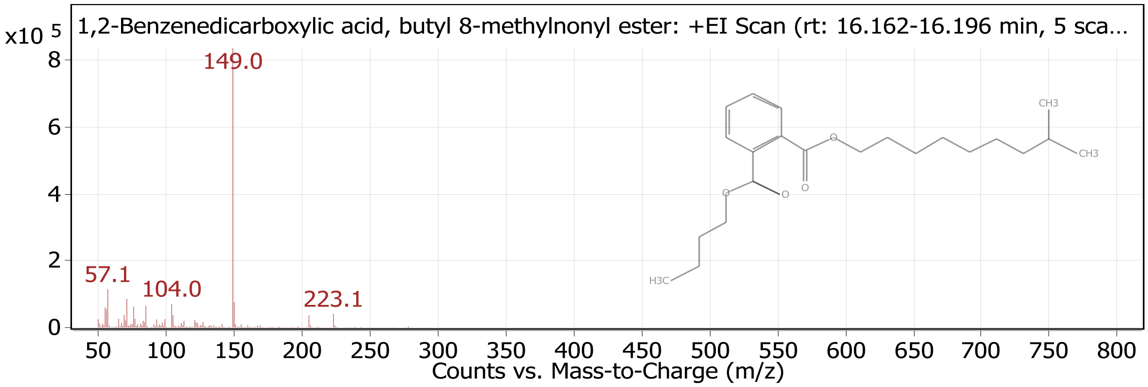

Library Spectrum

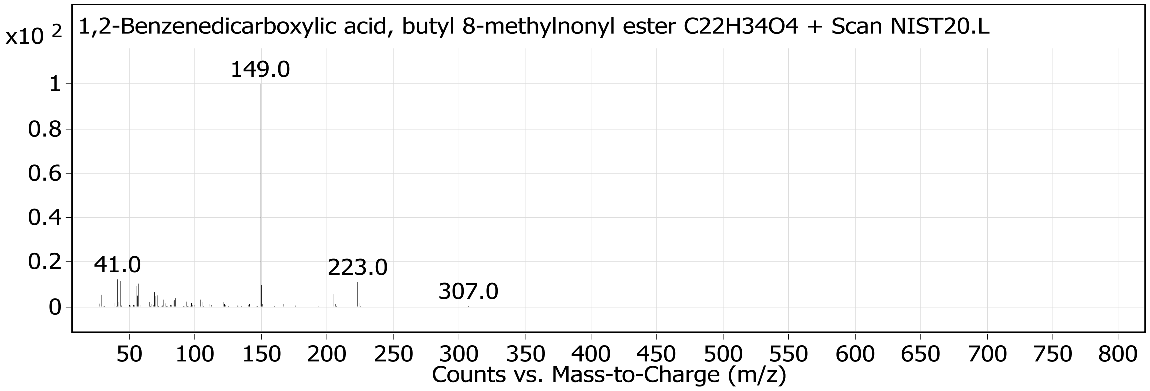

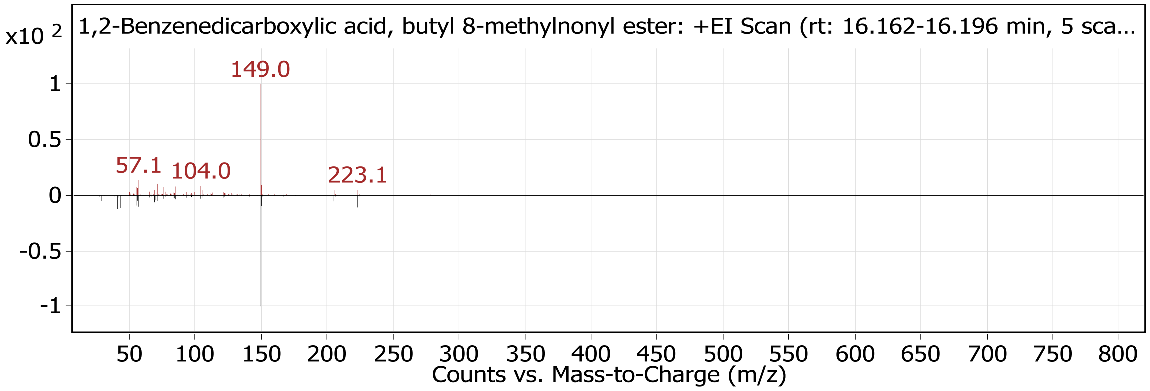

Spectrum Structure

1,2-Benzenedicarboxylic acid, butyl 8-methylnonyl ester

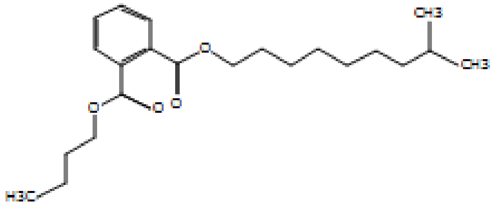

Spectrum Source	Collision Energy	Ionization Mode
Peak (7) in "+ TIC Scan"	0	EI

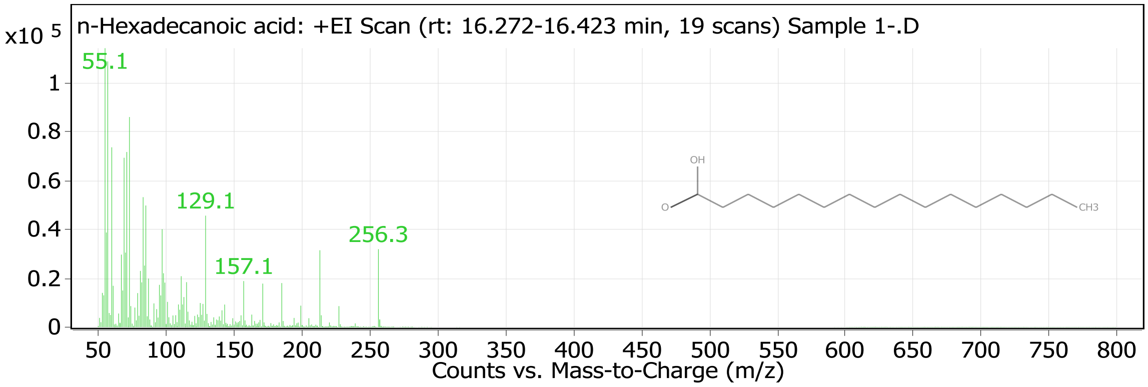

Library Spectrum

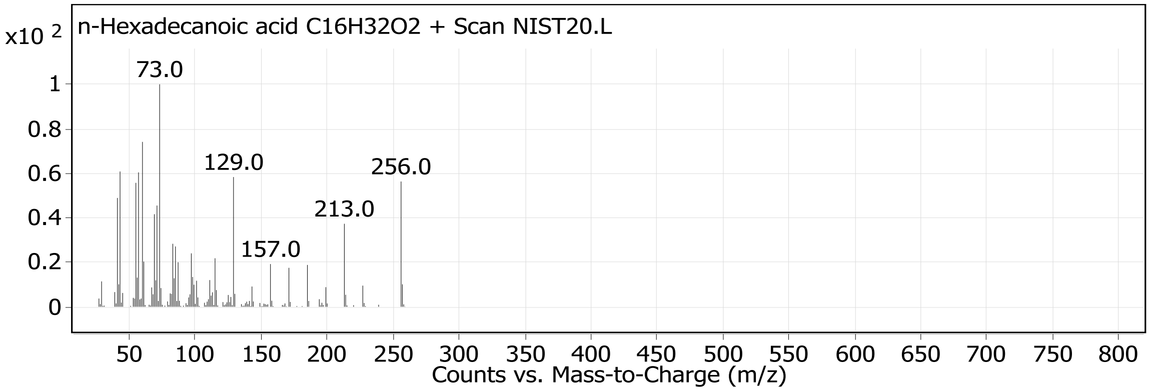

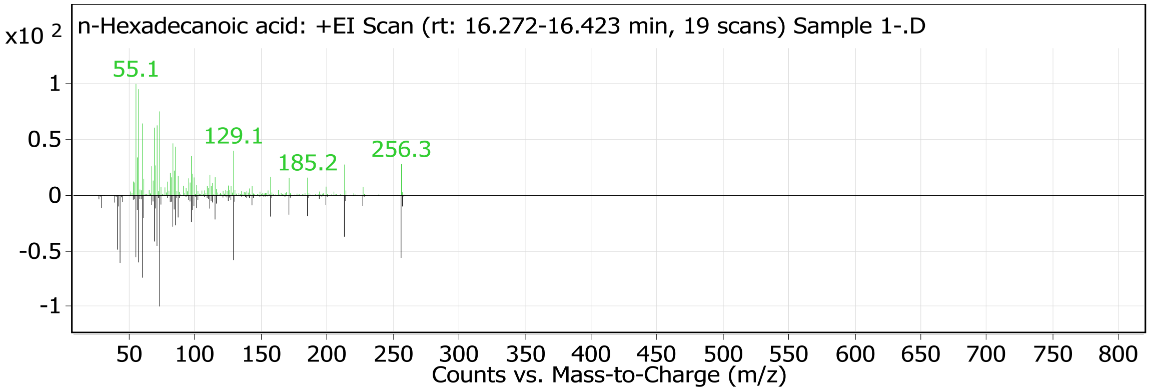

Spectrum Structure

n-Hexadecanoic acid

Qualitative Analysis Report

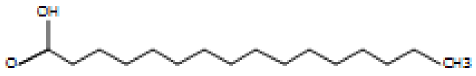

Spectrum Source	Collision Energy	Ionization Mode
Peak (8) in "+ TIC Scan"	0	EI

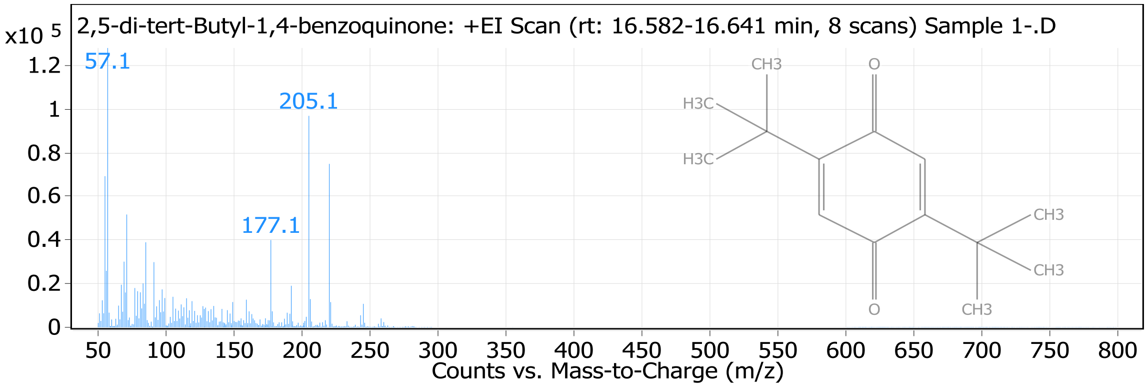

Library Spectrum

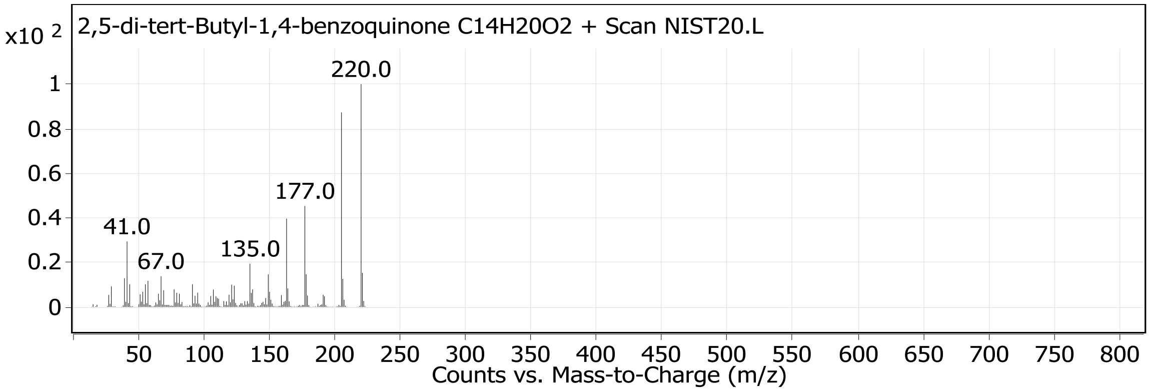

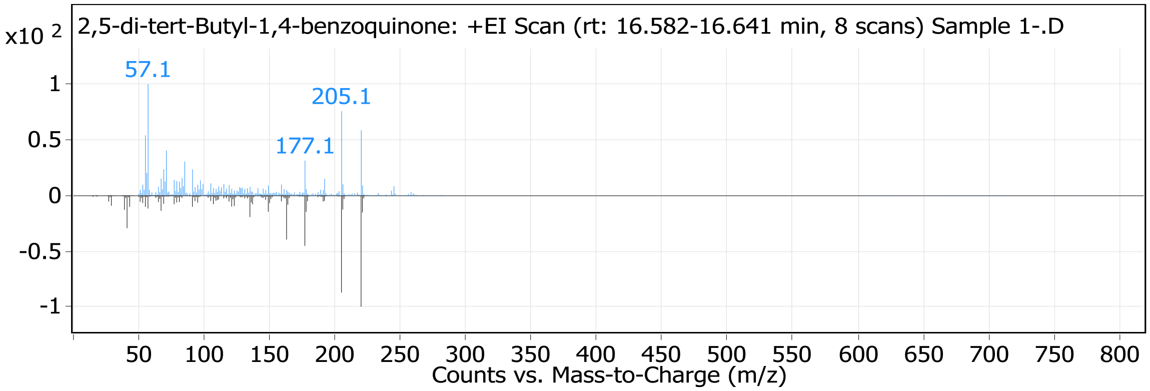

Qualitative Analysis Report

Spectrum Structure

2,5-di-tert-Butyl-1,4-benzoquinone

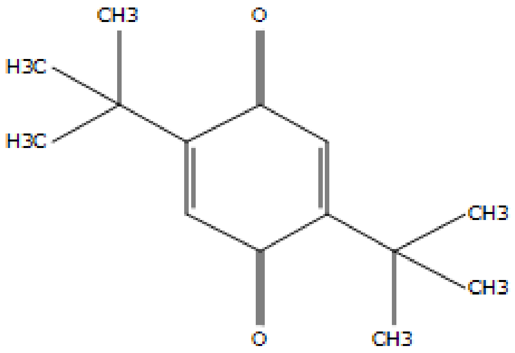

**Spectrum Source**	**Collision Energy**	**Ionization Mode**
Peak (9) in "+ TIC Scan"	0	EI

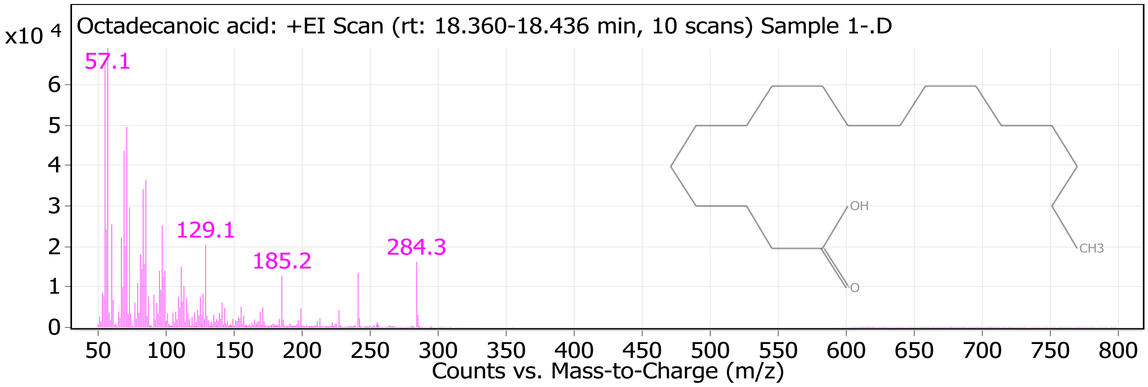

Library Spectrum

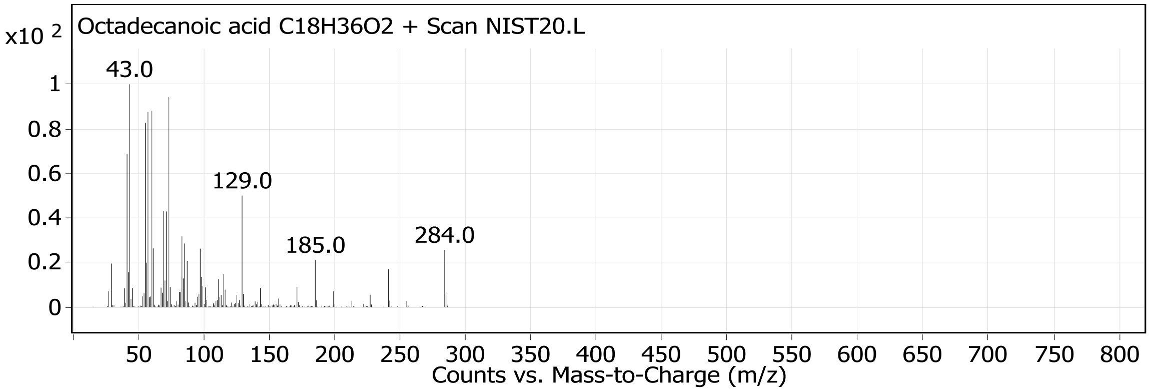

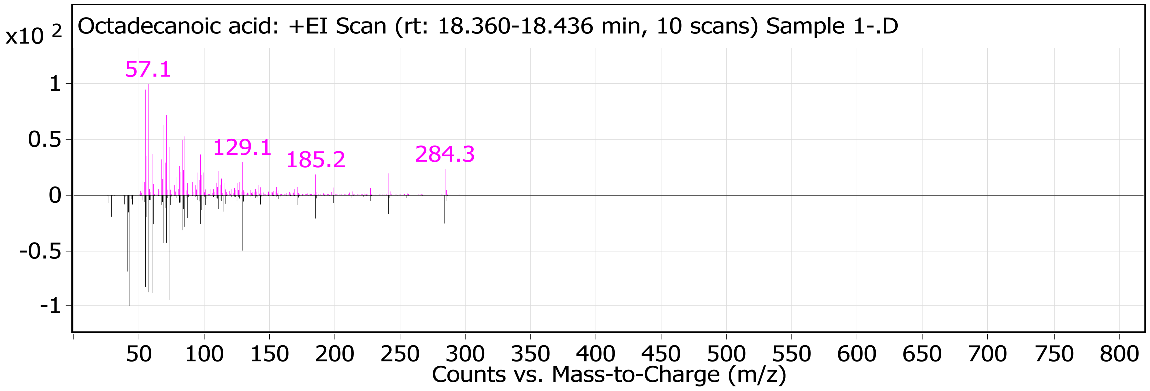

x10 2
Octadecanoic acid: +EI Scan (rt: 18.360-18.436 min, 10 scans) Sample 1-.D
57.1
129.1
185.2
284.3
1
0.5
0
-0.5
-1
50 100 150 200 250 300 350 400 450 500 550 600 650 700 750 800
Counts vs. Mass-to-Charge (m/z)

Spectrum Structure

Octadecanoic acid

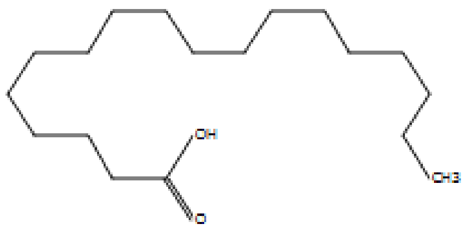

**Spectrum Source**	**Collision Energy**	**Ionization Mode**
Peak (10) in "+ TIC Scan"	0	EI

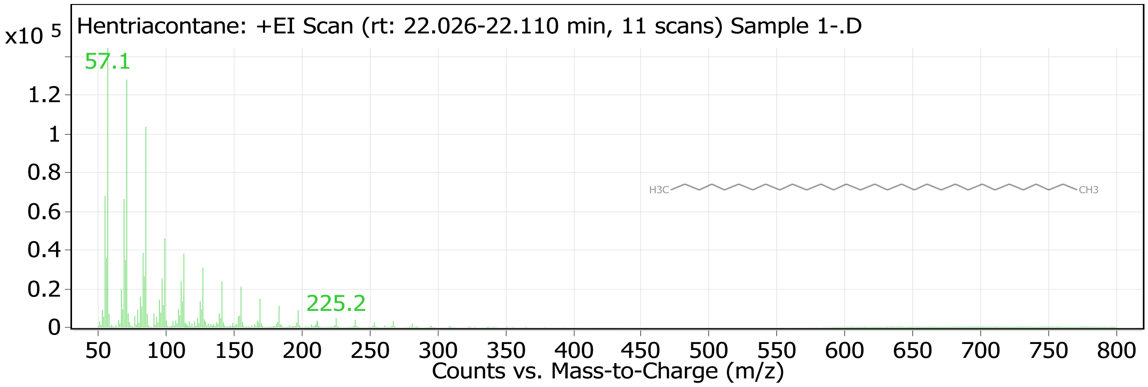

Library Spectrum

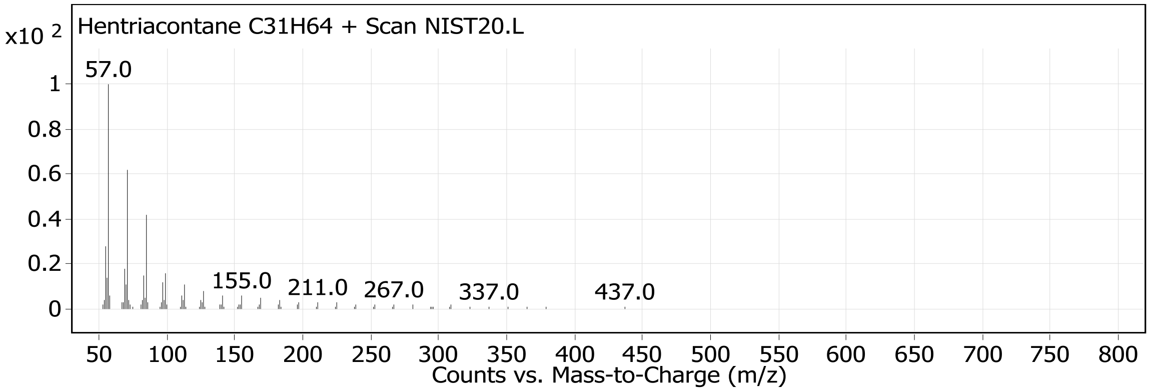

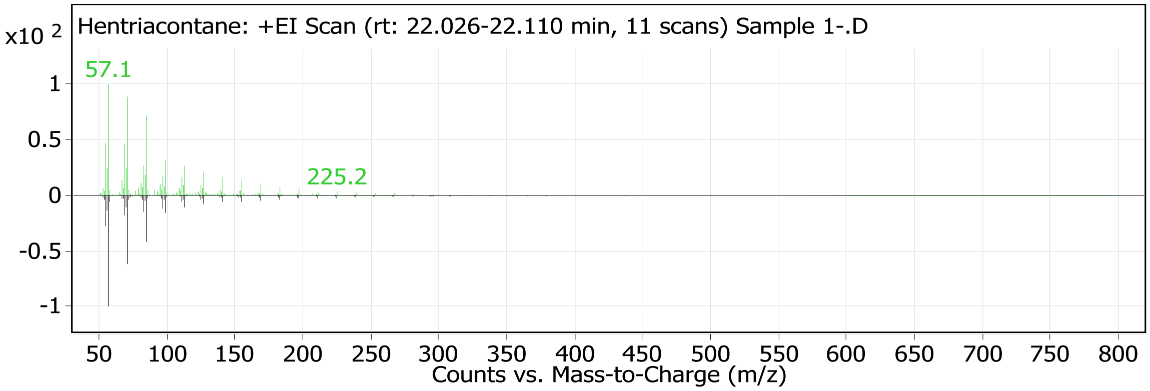

Spectrum Structure

Hentriacontane

**Spectrum Source**	**Collision Energy**	**Ionization Mode**
Peak (11) in "+ TIC Scan"	0	EI

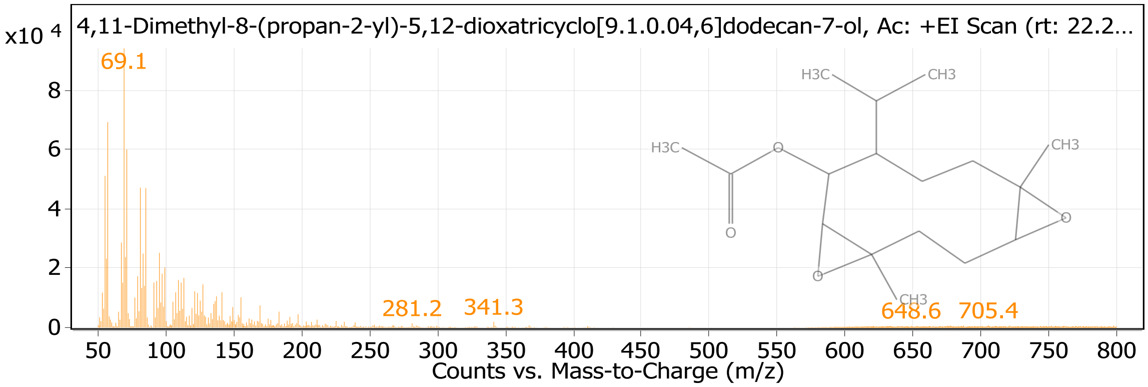

Library Spectrum

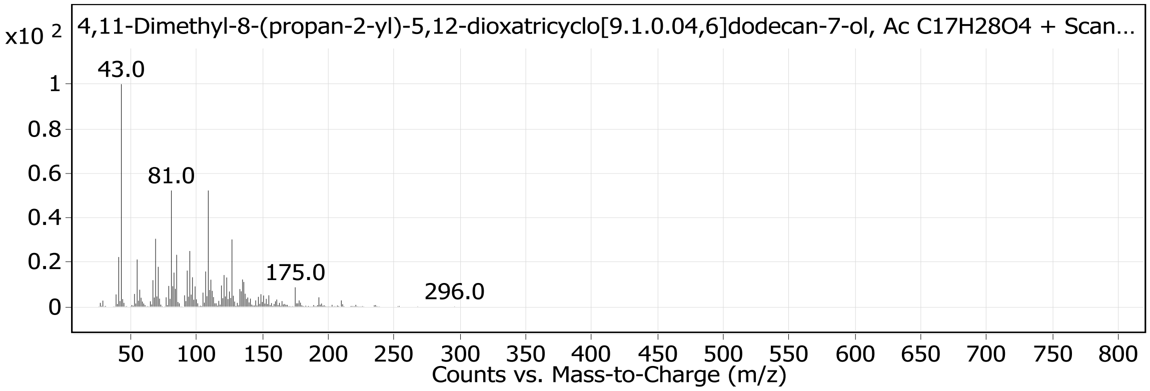

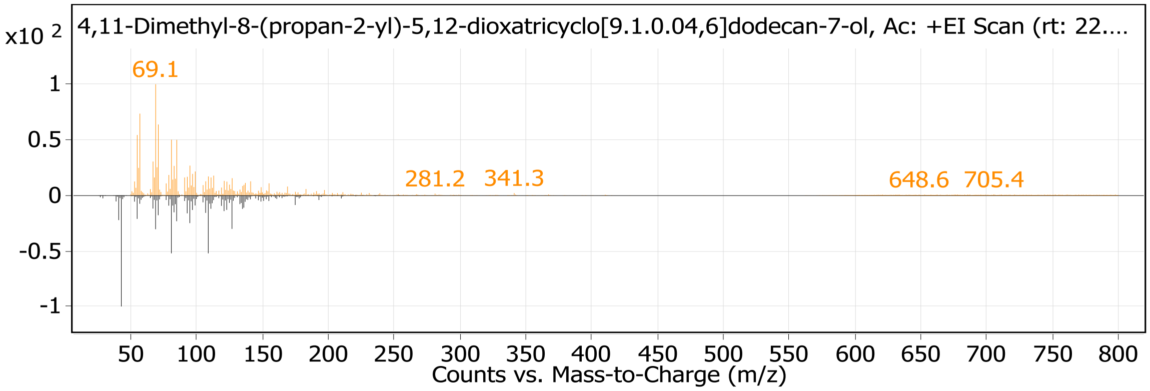

Spectrum Structure

4,11-Dimethyl-8-(propan-2-yl)-5,12-dioxatricyclo[9.1.0.04,6]dodecan-7-ol, Ac

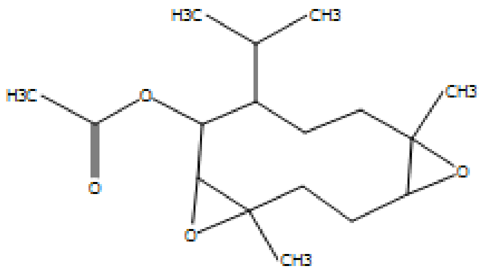

Spectrum Source	Collision Energy	Ionization Mode
Peak (12) in "+ TIC Scan"	0	EI

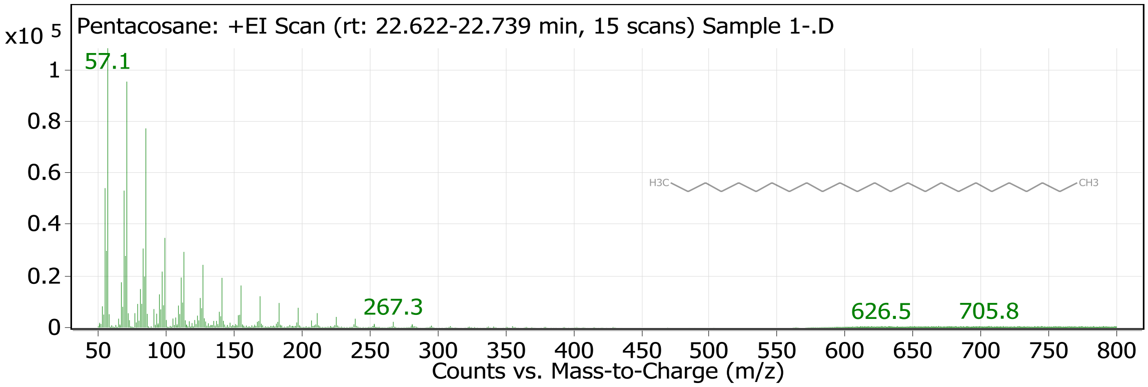

Library Spectrum

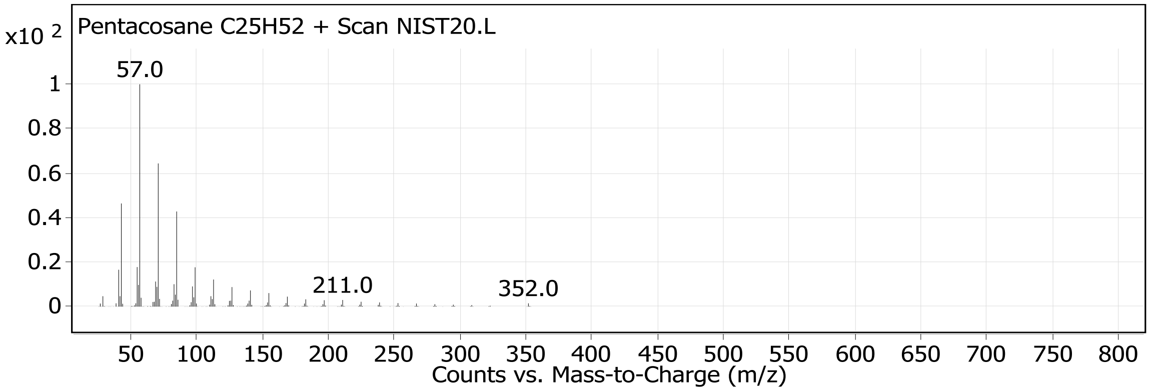

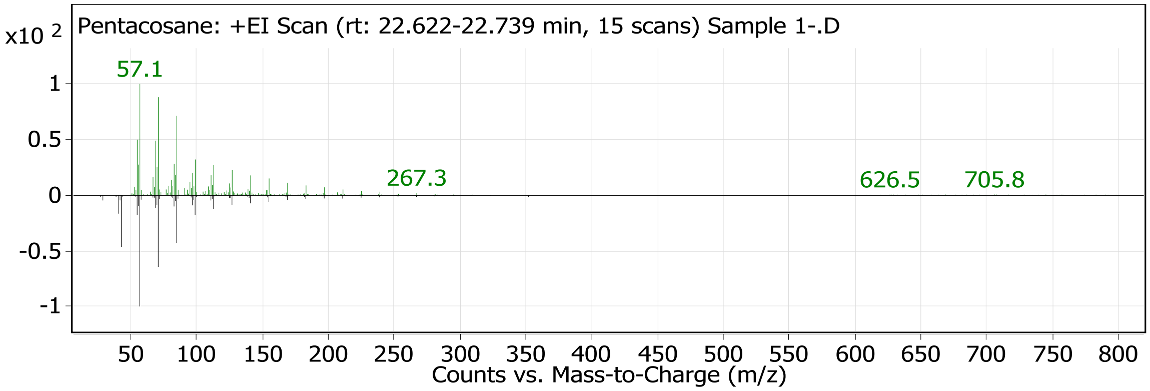

Spectrum Structure

Pentacosane

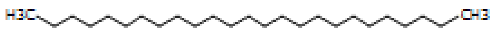

Qualitative Analysis Report

Spectrum Source	Collision Energy	Ionization Mode
Peak (13) in "+ TIC Scan"	0	EI

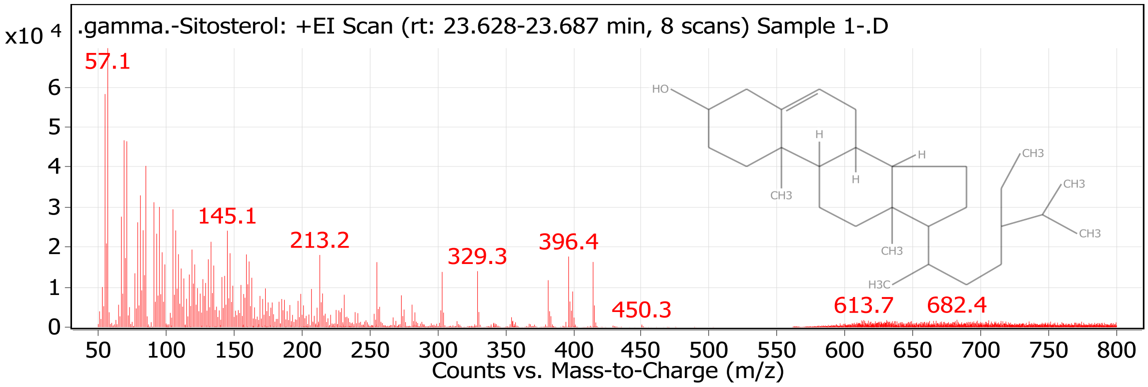

Library Spectrum

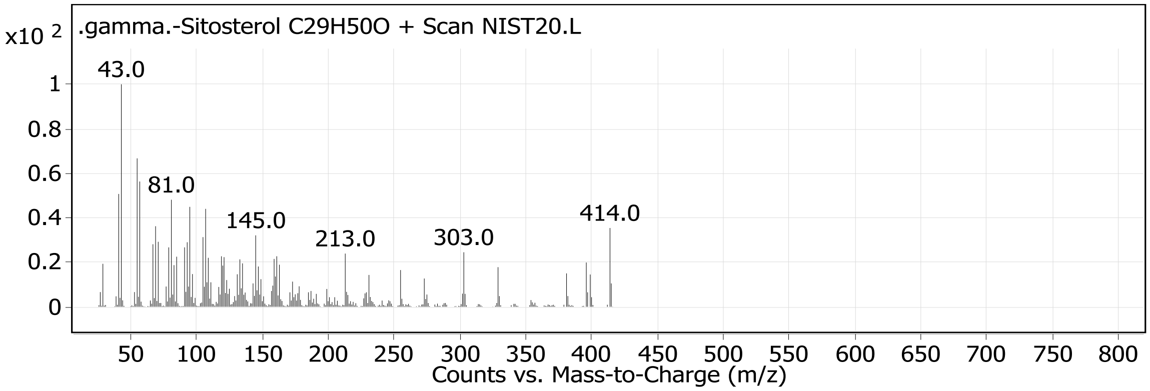

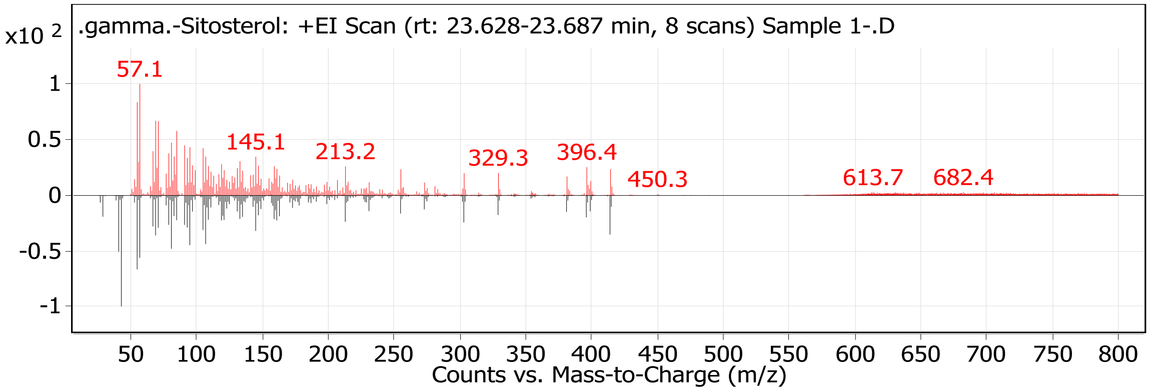

Spectrum Structure

.gamma.-Sitosterol

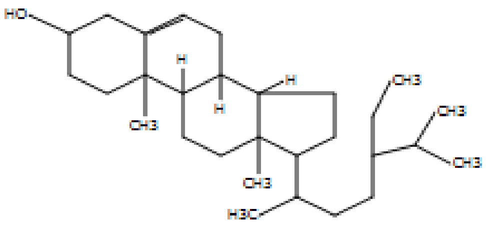

--- End Of Report ---
