# Supplementary material for: Chemical and Molecular Insights into the Arid Wild Plant Diversity of Saudi Arabia
Source: Plants (Basel). 2026 Jan 19;15(2):295. doi: 10.3390/plants15020295 (PMC12845481; doi:10.3390/plants15020295)
Supplement: Supplementary file 1 [file plants-15-00295-s001.zip › Sample 2_AnalysisReport.pdf]

Qualitative Analysis Report

**Data Filename**	Sample 2.D	**Sample Name**	Sample 2
**Sample Type**		**Position**	1
**Instrument Name**	3	**User Name**	
**Acq Method**	Scan DB-5MS Hydrogen 2024.M	**Acquired Time**	6/24/2024 4:46:51 PM
**IRM Calibration Status**	Not Applicable	**DA Method**	SignalToNoiseCheckout.m
**Comment**			

**Expected Barcode**		**Sample Amount**	
**Dual Inj Vol**	0.2	**TuneName**	ATUNE.U
**TunePath**	D:\MassHunter\GCMS\3\5977 \	**TuneDateStamp**	2024-06-23T14:01:57+02:00
**MSFirmwareVersion**	6.00.34	**OperatorName**	
**RunCompletedFlag**	True	**Acquisition SW Version**	MassHunter GC/MS Acquisition 10.0.368 14-Feb-2019 Copyright © 1989-2018 Agilent Technologies, Inc

User Chromatograms

Fragmentor Voltage **Collision Energy** 0 **Ionization Mode** EI

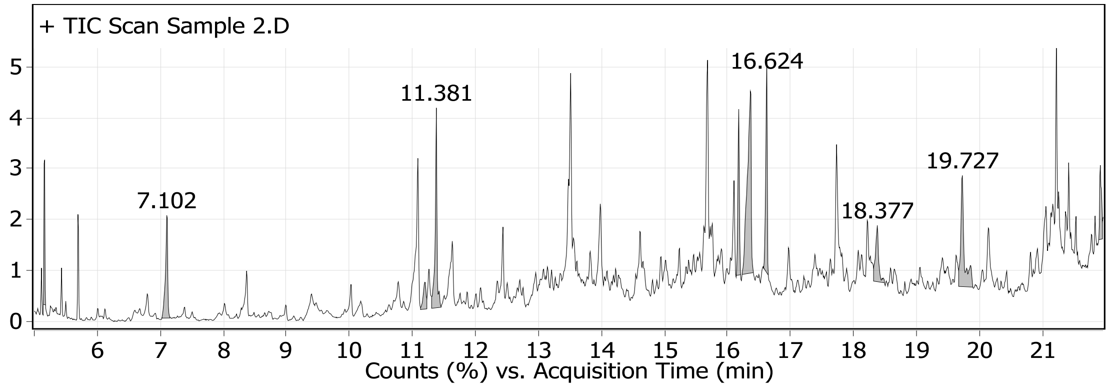

Integration Peak List

Peak	Start	RT	End	Height	Area	Area %
1	5.143	5.165	5.18	1036482.26	1084980.71	19.43
2	7.027	7.102	7.153	734280.53	1809066	32.4
3	11.129	11.196	11.23	192317.32	617335.45	11.06
4	11.305	11.381	11.456	1429953.58	2858424.22	51.2
5	16.139	16.179	16.209	1185466.06	1897039.51	33.98
6	16.232	16.364	16.411	1300683.19	5582923.17	100
7	16.574	16.624	16.648	1533384.1	2454011.36	43.96
8	18.318	18.377	18.492	401652.71	1406069.3	25.19
9	19.669	19.727	19.894	794544.89	3118216.15	55.85
10	21.891	21.917	21.95	530530.52	1070105.12	19.17

User Spectra

Spectrum Source	Collision Energy	Ionization Mode
Peak (1) in "+ TIC Scan"	0	EI

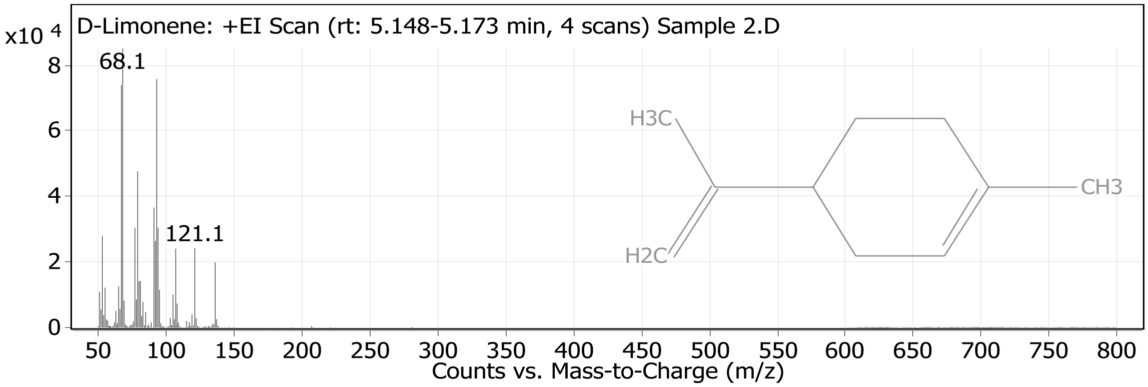

Library Spectrum

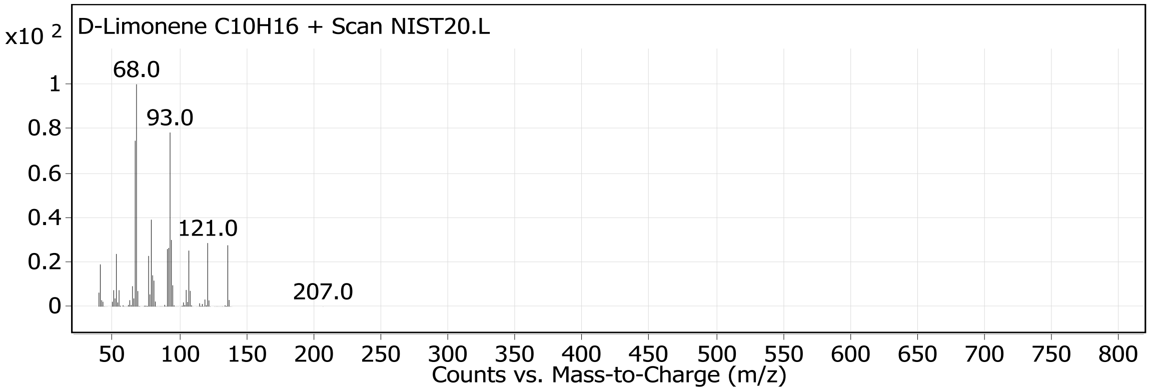

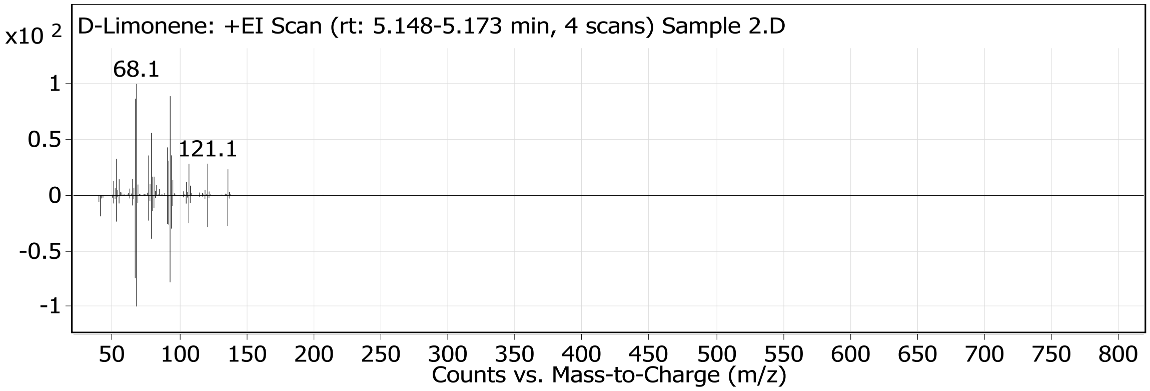

Spectrum Structure

D-Limonene

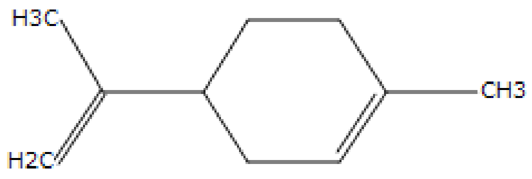

Qualitative Analysis Report

Spectrum Source	Collision Energy	Ionization Mode
Peak (2) in "+ TIC Scan"	0	EI

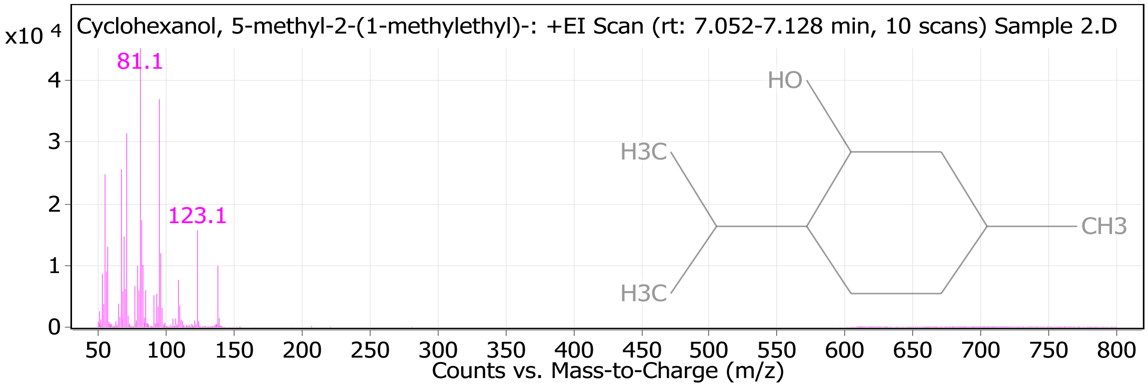

Library Spectrum

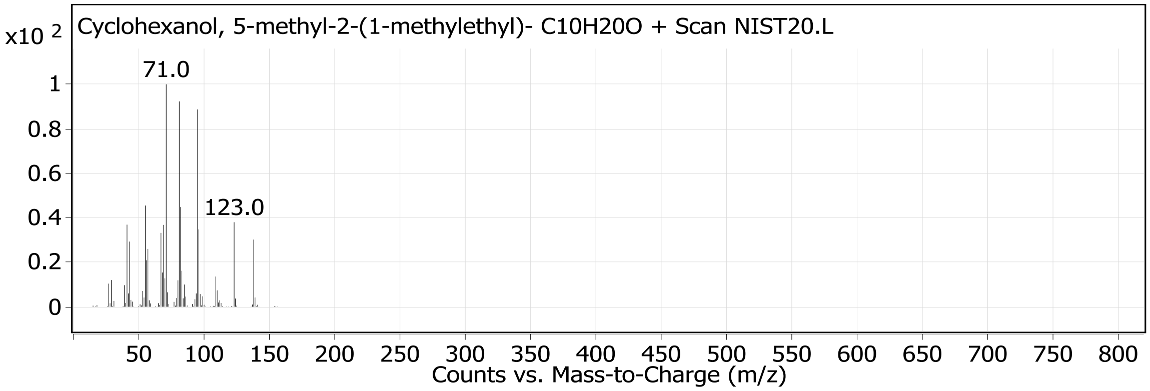

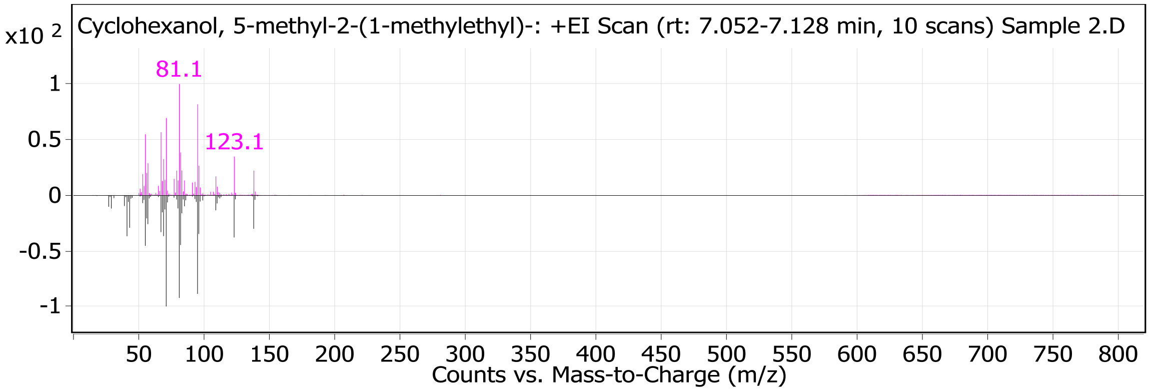

Spectrum Structure

Cyclohexanol, 5-methyl-2-(1-methylethyl)-

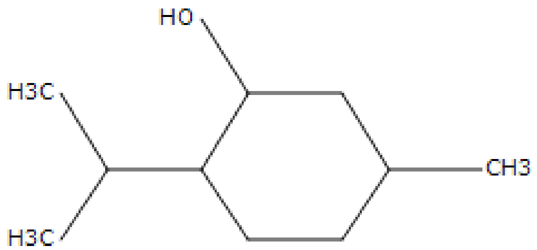

**Spectrum Source**	**Collision Energy**	**Ionization Mode**
Peak (3) in "+ TIC Scan"	0	EI

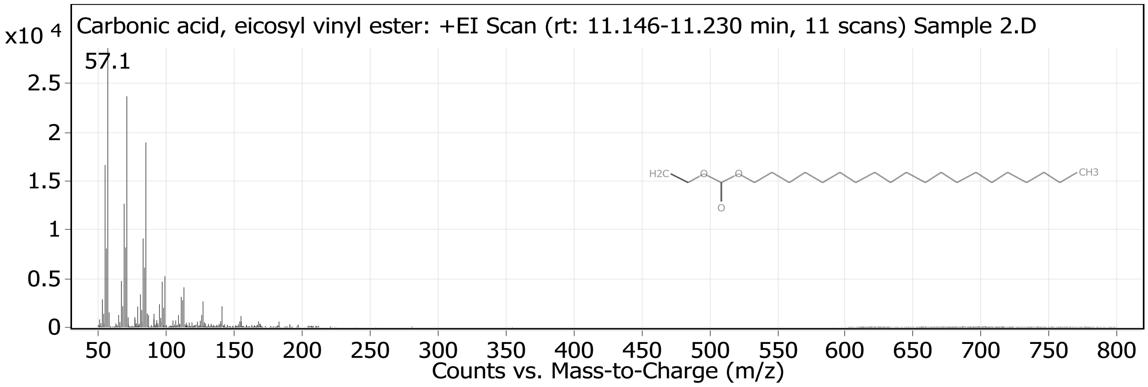

Library Spectrum

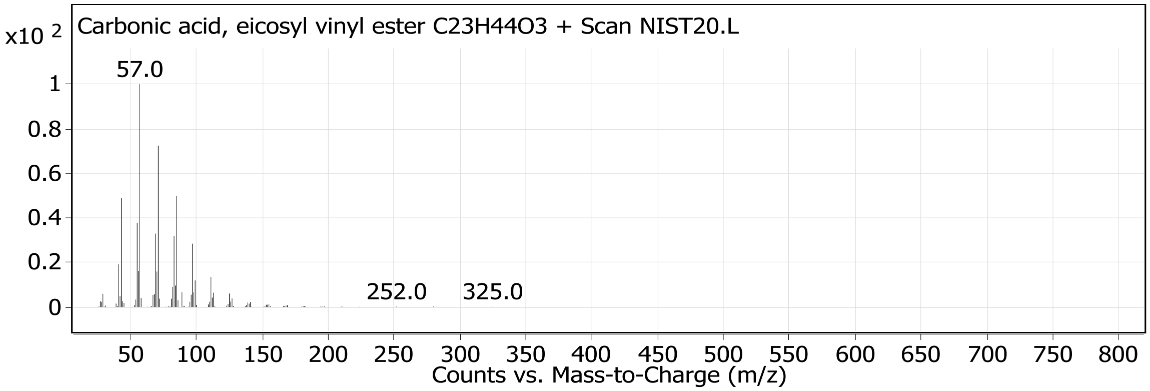

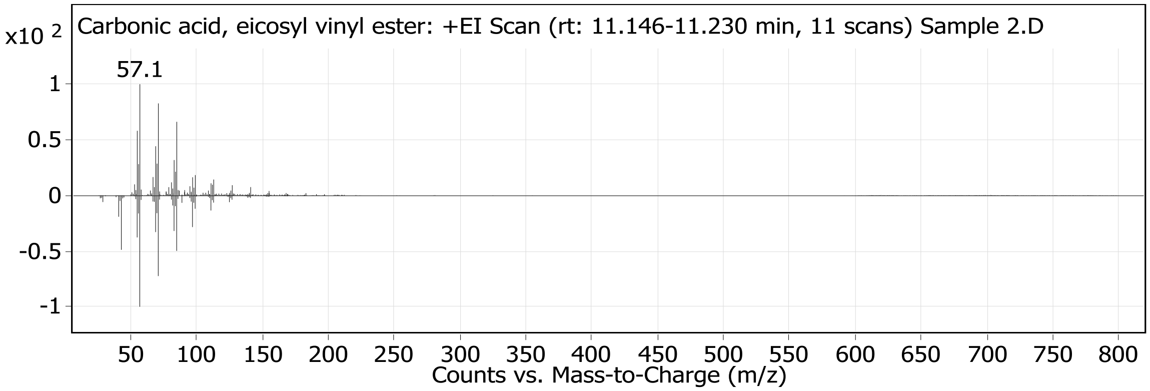

Spectrum Structure

Carbonic acid, eicosyl vinyl ester

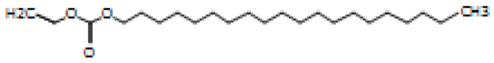

Spectrum Source	Collision Energy	Ionization Mode
Peak (4) in "+ TIC Scan"	0	EI

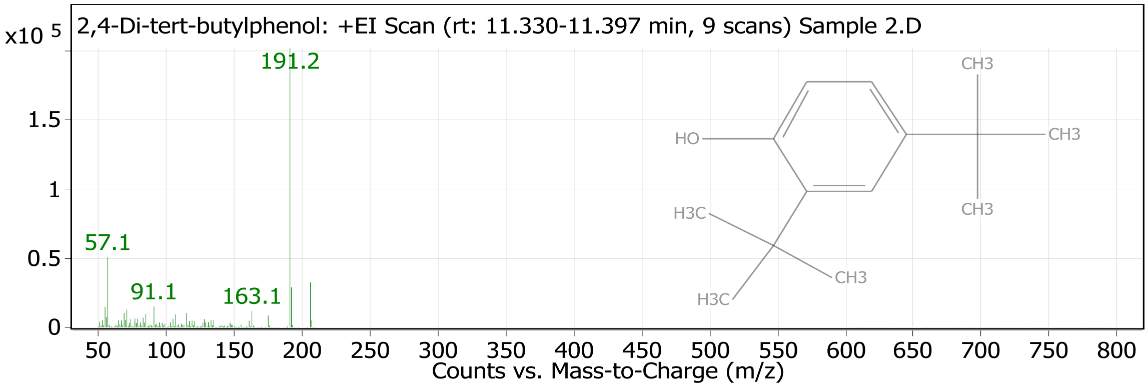

Library Spectrum

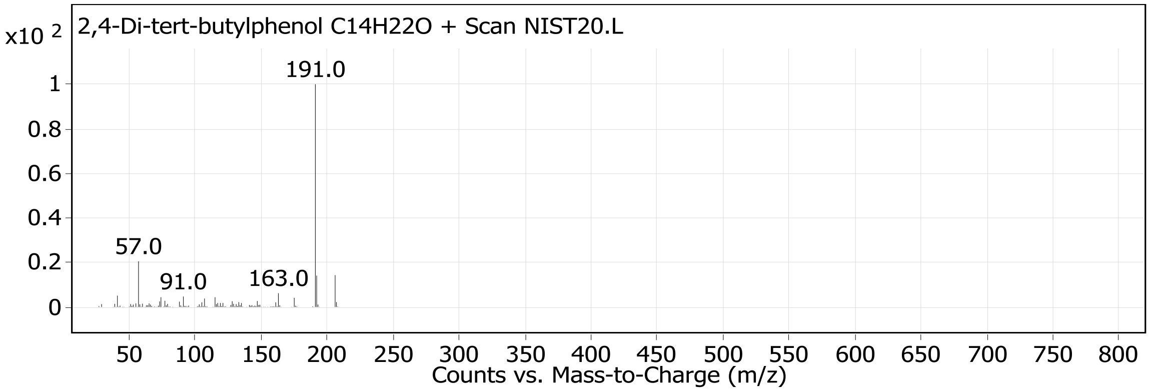

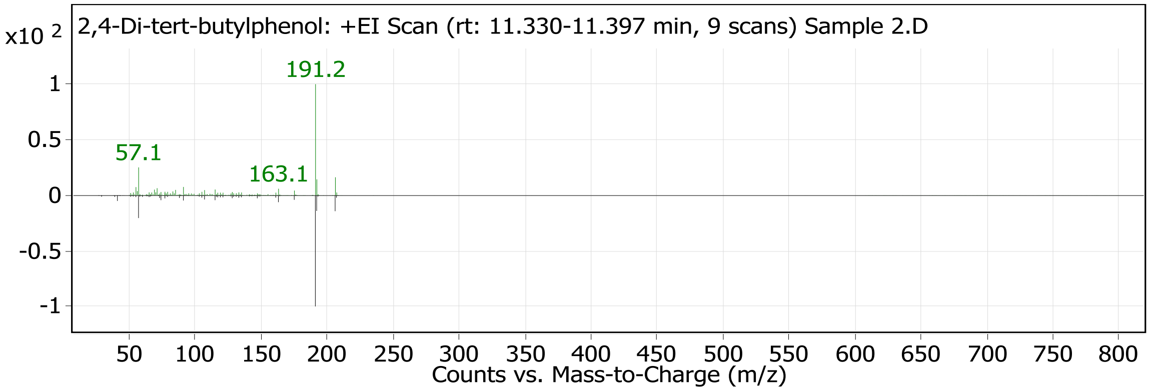

Spectrum Structure

2,4-Di-tert-butylphenol

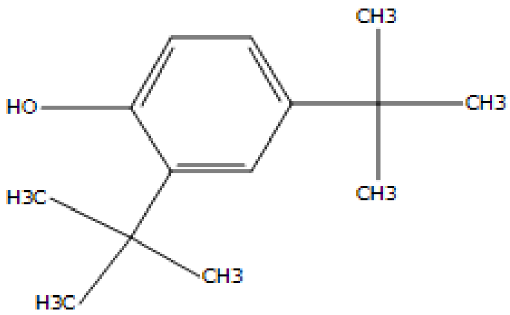

Spectrum Source	Collision Energy	Ionization Mode
Peak (5) in "+ TIC Scan"	0	EI

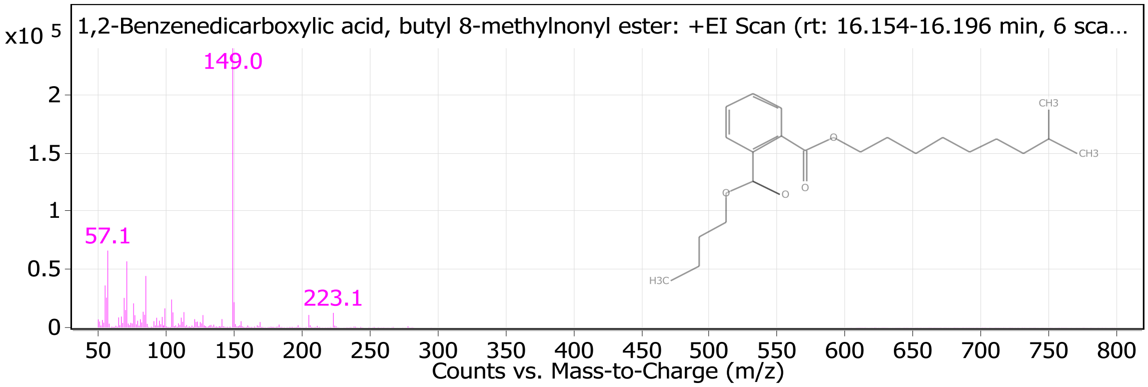

Library Spectrum

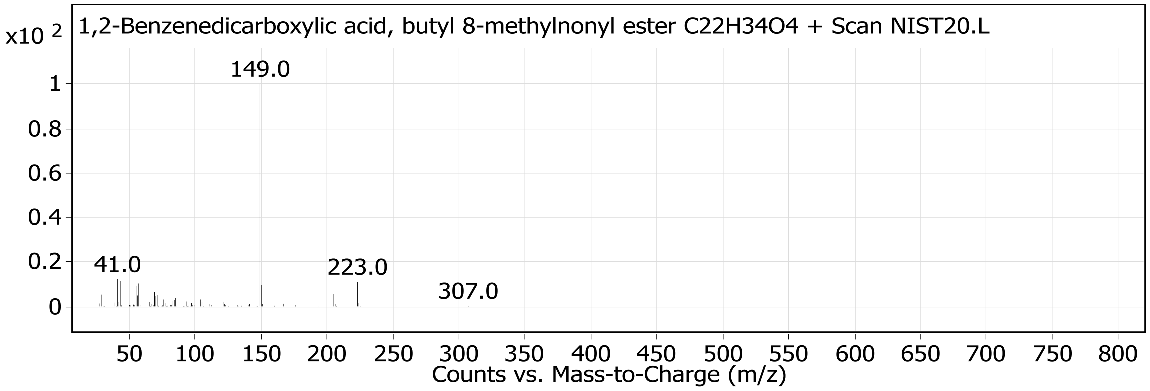

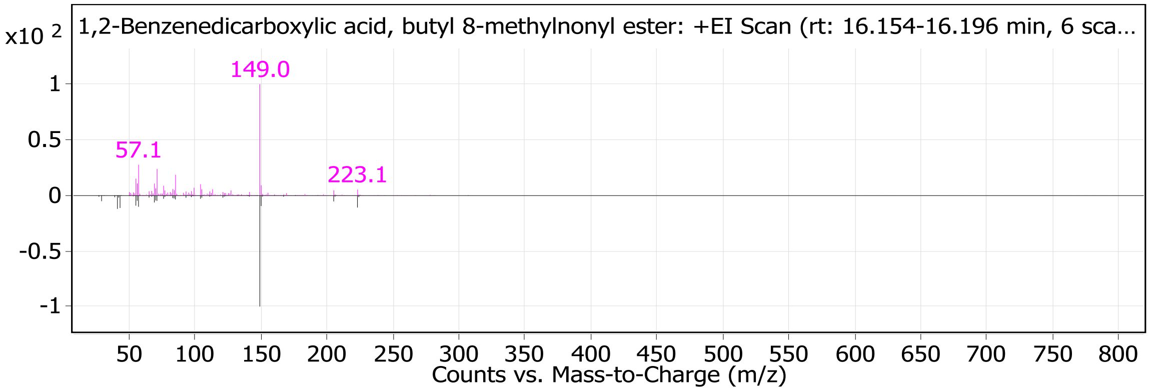

Spectrum Structure

1,2-Benzenedicarboxylic acid, butyl 8-methylnonyl ester

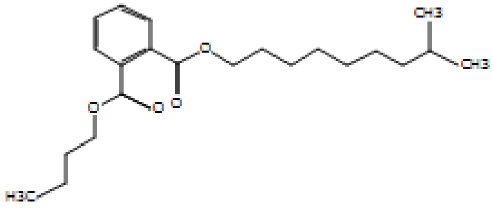

Spectrum Source	Collision Energy	Ionization Mode
Peak (6) in "+ TIC Scan"	0	EI

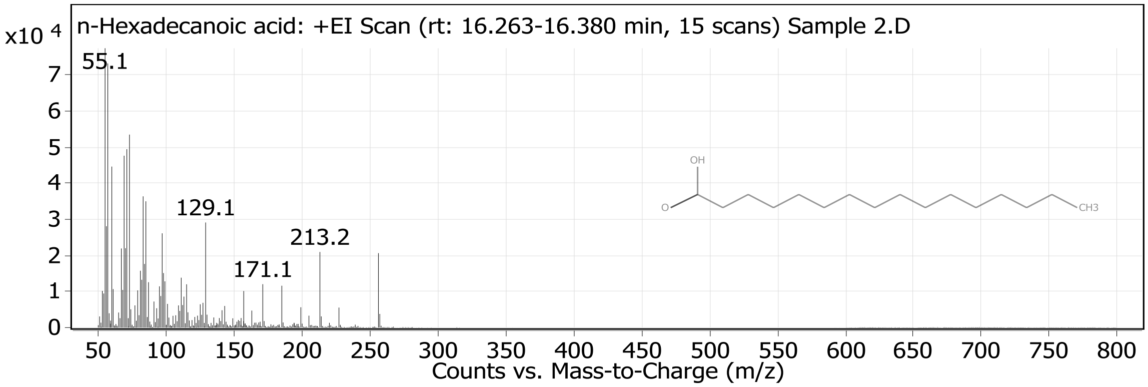

Library Spectrum

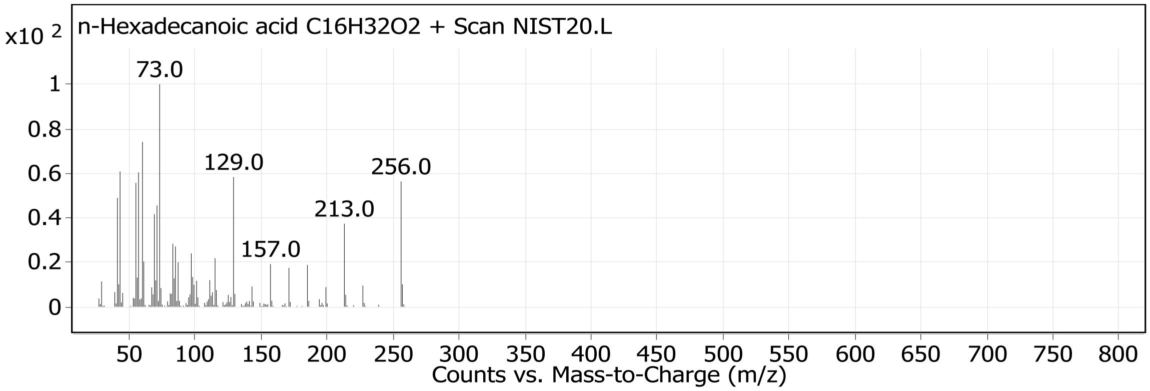

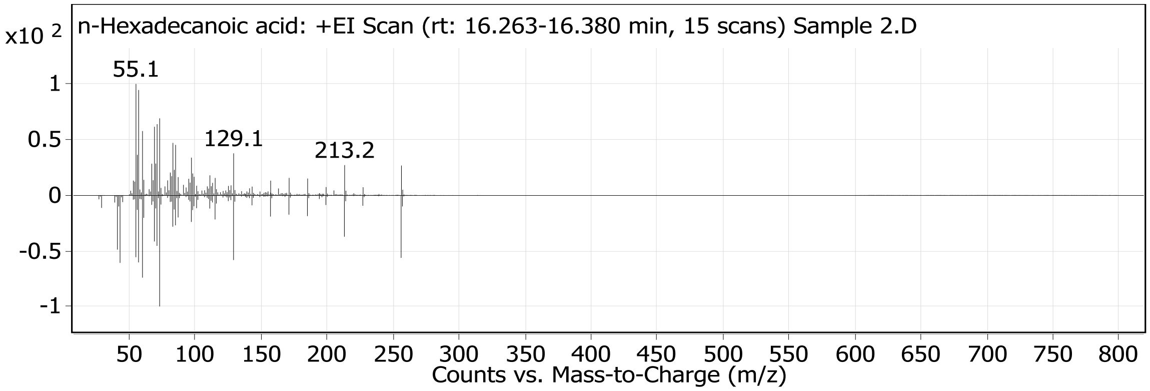

Spectrum Structure

n-Hexadecanoic acid

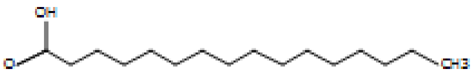

Qualitative Analysis Report

Spectrum Source	Collision Energy	Ionization Mode
Peak (7) in "+ TIC Scan"	0	EI

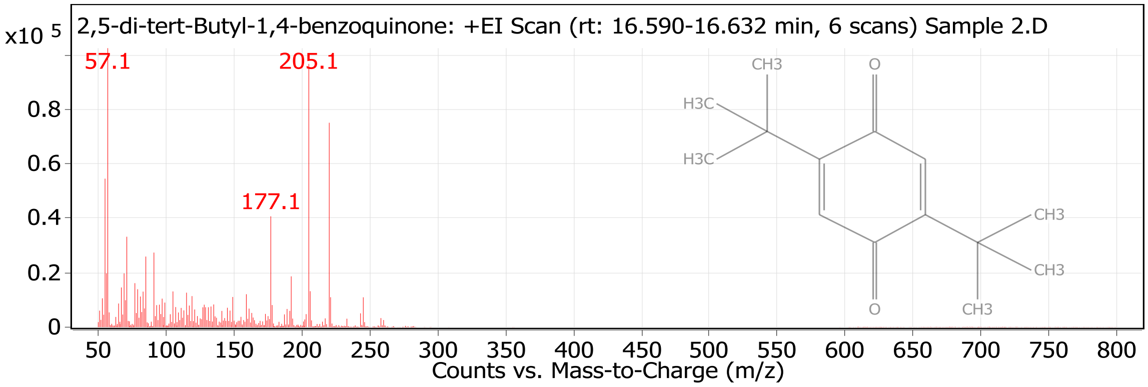

Library Spectrum

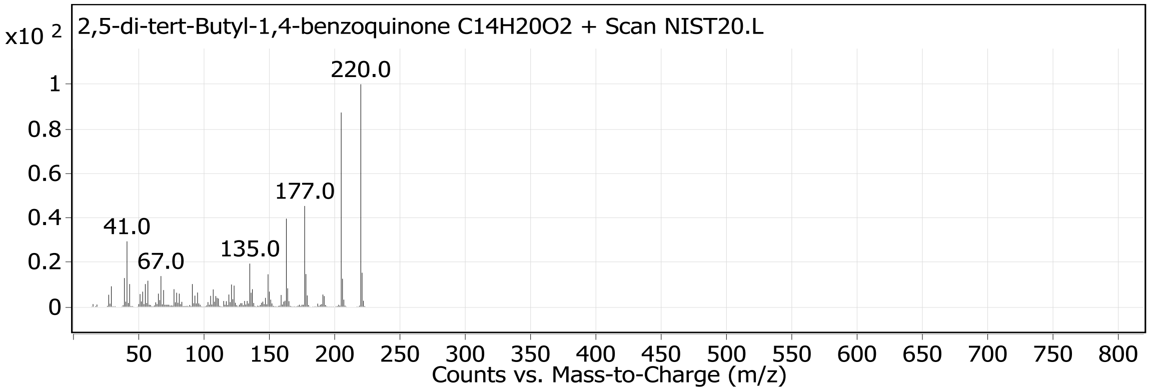

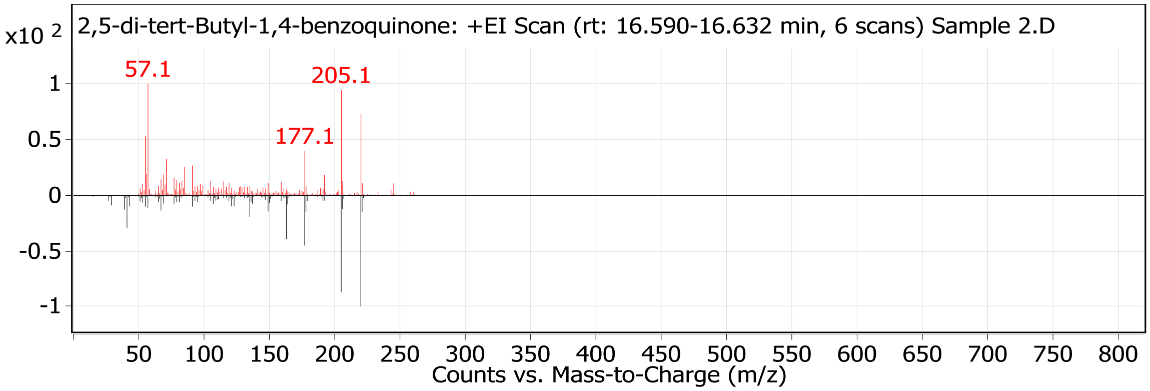

Spectrum Structure

2,5-di-tert-Butyl-1,4-benzoquinone

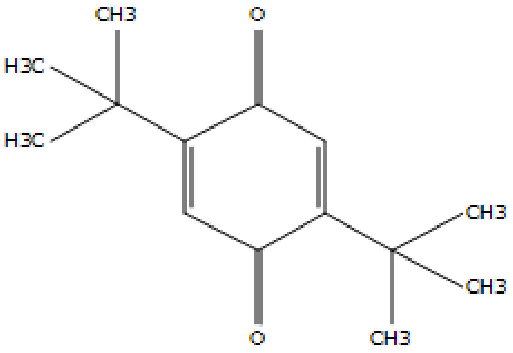

Spectrum Source	Collision Energy	Ionization Mode
Peak (8) in "+ TIC Scan"	0	EI

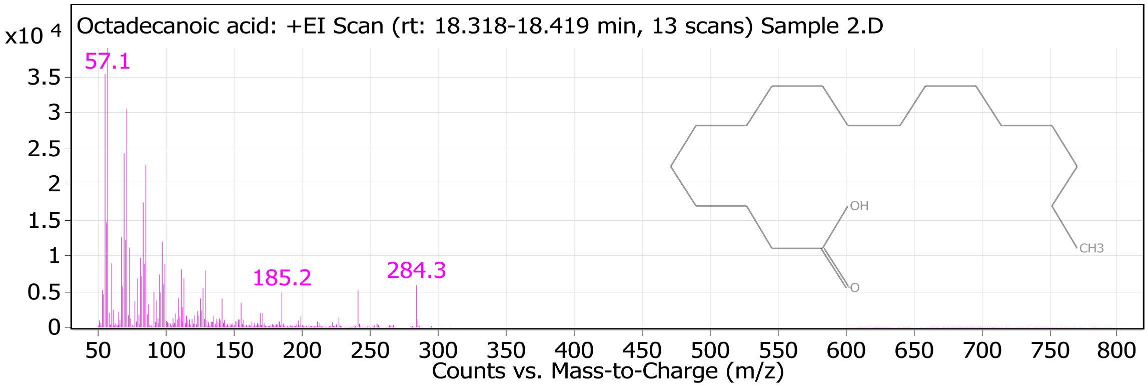

Library Spectrum

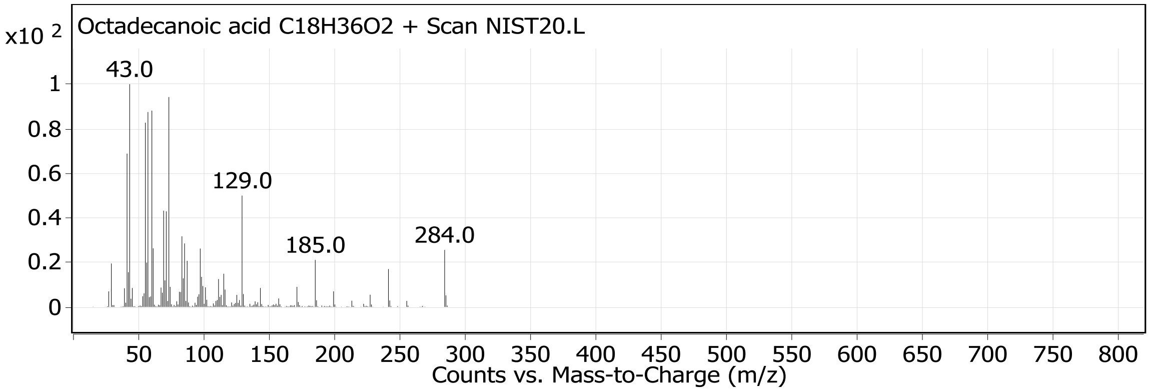

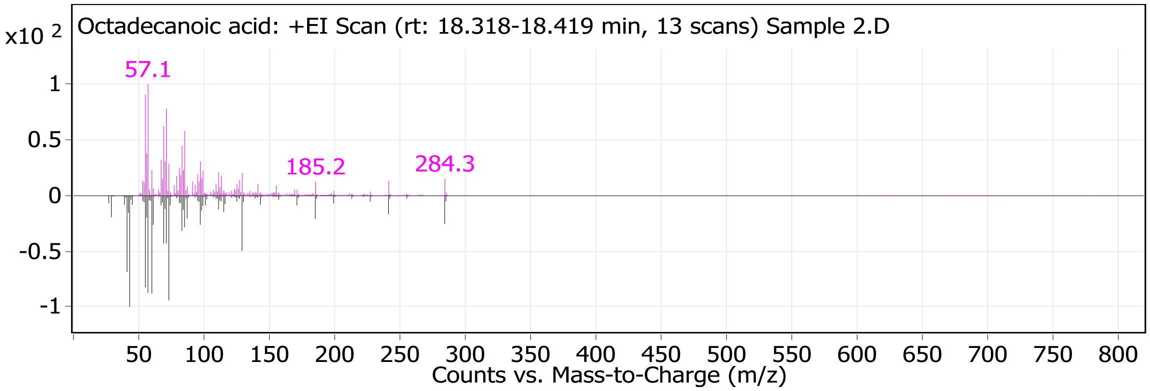

Spectrum Structure

Octadecanoic acid

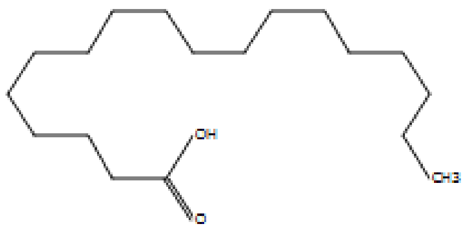

Spectrum Source	Collision Energy	Ionization Mode
Peak (9) in "+ TIC Scan"	0	EI

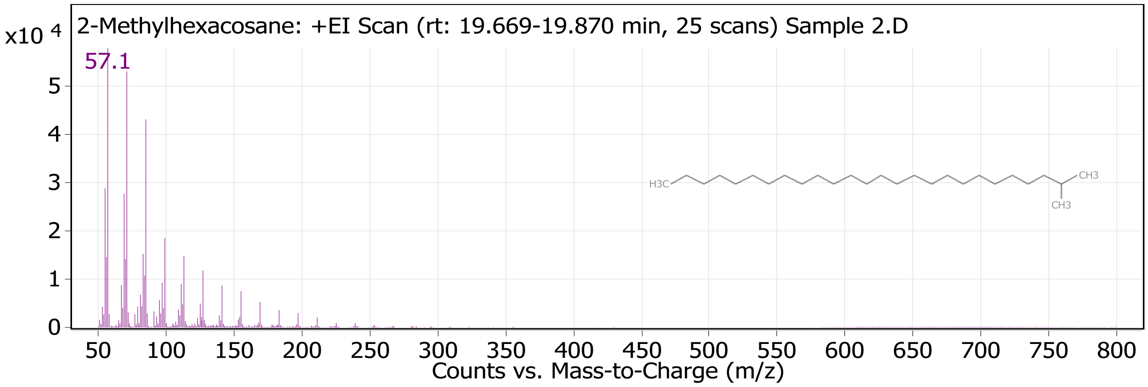

Library Spectrum

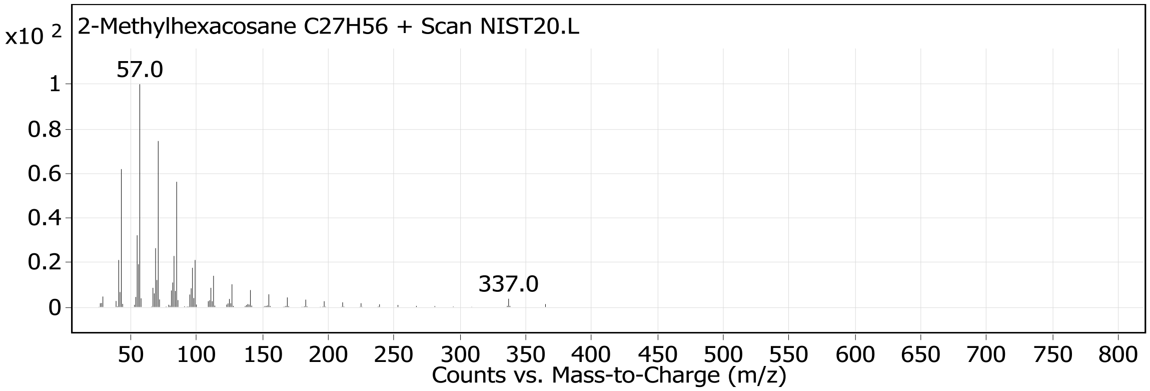

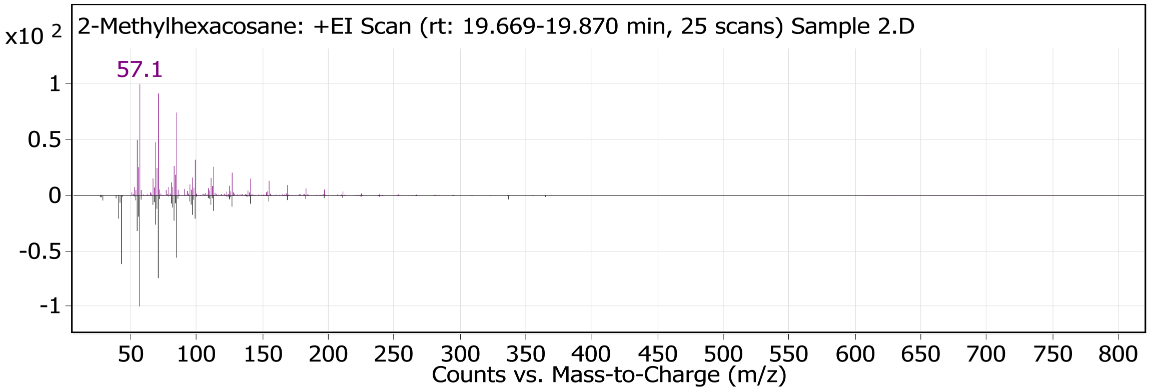
2-Methylhexacosane: +EI Scan (rt: 19.669-19.870 min, 25 scans) Sample 2.D
x10 2
57.1
1
0.5
0
-0.5
-1
50 100 150 200 250 300 350 400 450 500 550 600 650 700 750 800
Counts vs. Mass-to-Charge (m/z)

Spectrum Structure

2-Methylhexacosane

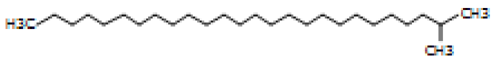

**Spectrum Source**	**Collision Energy**	**Ionization Mode**
Peak (10) in "+ TIC Scan"	0	EI

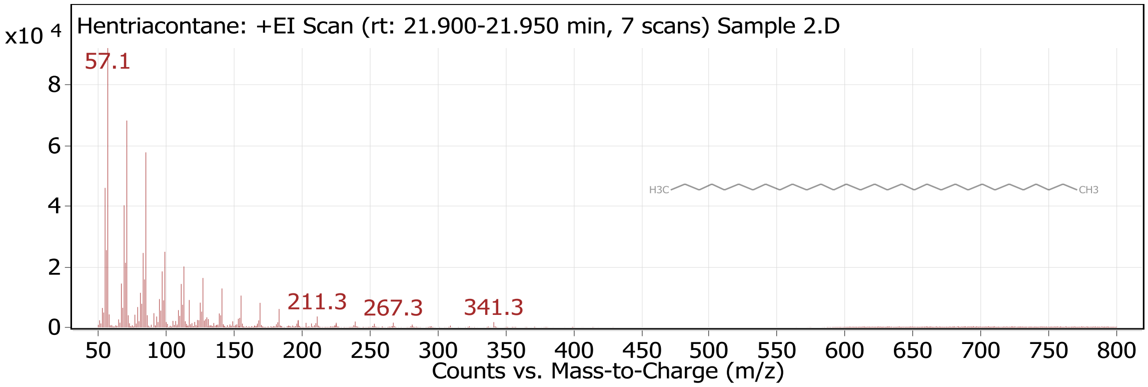

Library Spectrum

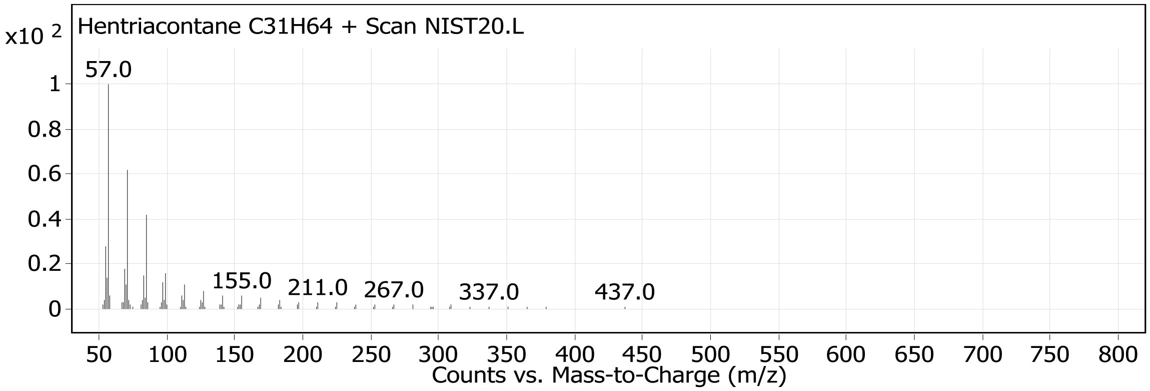

Qualitative Analysis Report

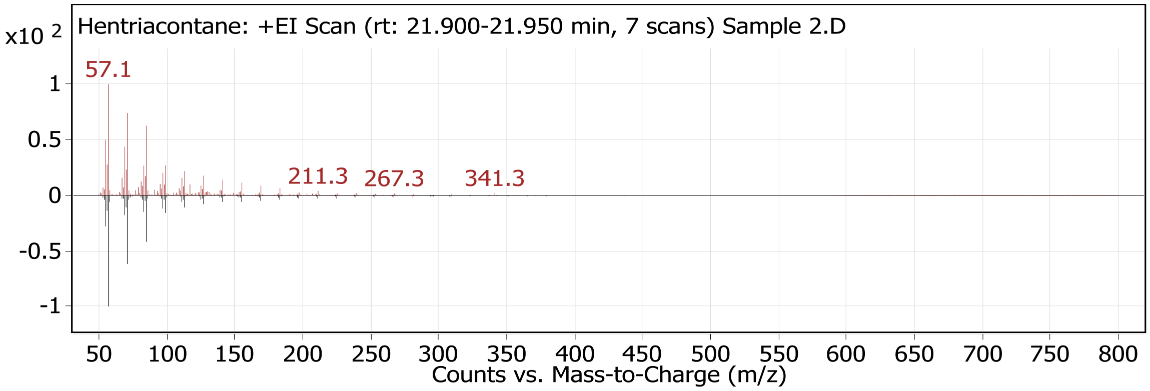

Spectrum Structure

Hentriacontane

--- End Of Report ---
