# Supplementary material for: Chemical and Molecular Insights into the Arid Wild Plant Diversity of Saudi Arabia
Source: Plants (Basel). 2026 Jan 19;15(2):295. doi: 10.3390/plants15020295 (PMC12845481; doi:10.3390/plants15020295)
Supplement: Supplementary file 1 [file plants-15-00295-s001.zip › Sample 3_AnalysisReport.pdf]

Qualitative Analysis Report

**Data Filename**	Sample 3.D	**Sample Name**	Sample 3
**Sample Type**		**Position**	1
**Instrument Name**	3	**User Name**	
**Acq Method**	Scan DB-5MS Hydrogen 2024.M	**Acquired Time**	6/24/2024 5:29:22 PM
**IRM Calibration Status**	Not Applicable	**DA Method**	SignalToNoiseCheckout.m
**Comment**			

**Expected Barcode**		**Sample Amount**	
**Dual Inj Vol**	0.2	**TuneName**	ATUNE.U
**TunePath**	D:\MassHunter\GCMS\3\5977 \	**TuneDateStamp**	2024-06-23T14:01:57+02:00
**MSFirmwareVersion**	6.00.34	**OperatorName**	
**RunCompletedFlag**	True	**Acquisition SW Version**	MassHunter GC/MS Acquisition 10.0.368 14-Feb-2019 Copyright © 1989-2018 Agilent Technologies, Inc

User Chromatograms

Fragmentor Voltage **Collision Energy** 0 **Ionization Mode** EI

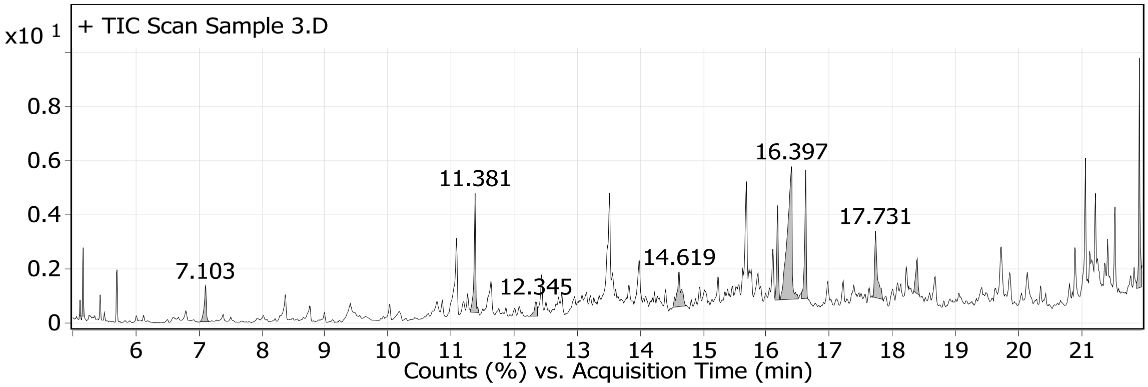

Integration Peak List

Peak	Start	RT	End	Height	Area	Area %
1	5.102	5.114	5.127	236905.15	143851.55	1.39
2	5.144	5.165	5.181	1007075.46	927723.7	8.94
3	7.019	7.103	7.161	525756.94	1255918.01	12.1
4	11.305	11.381	11.439	1753397.96	3085221.47	29.73
5	12.253	12.345	12.371	212630.75	664100.4	6.4
6	14.519	14.619	14.72	507304.78	2309146.4	22.25
7	16.137	16.179	16.221	1389141.22	2364805.73	22.79
8	16.221	16.397	16.506	1953527.82	10378354.41	100
9	16.557	16.624	16.656	1888420.27	3296702.58	31.77
10	17.691	17.731	17.846	978034.19	2965021.82	28.57
11	18.338	18.394	18.431	519532.7	1250244.36	12.05
12	21.875	21.917	21.951	3381443.28	4074898.1	39.26

User Spectra

**Spectrum Source**	**Collision Energy**	**Ionization Mode**
Peak (1) in "+ TIC Scan"	0	EI

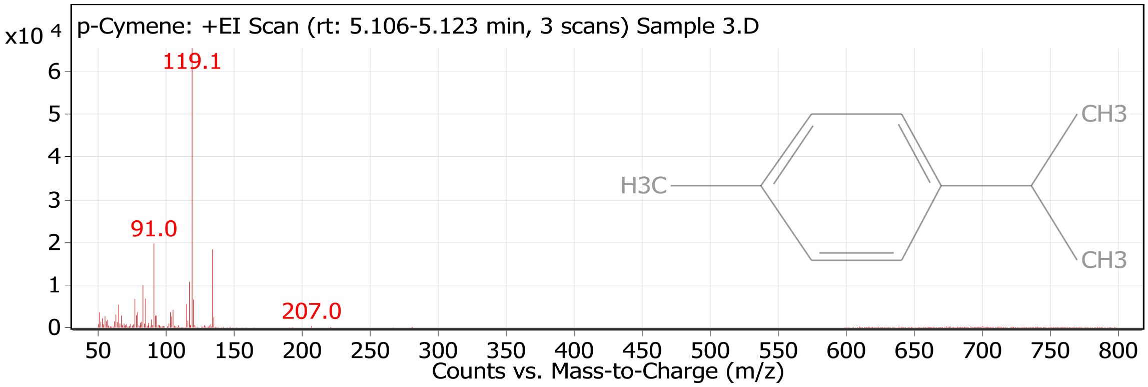

Library Spectrum

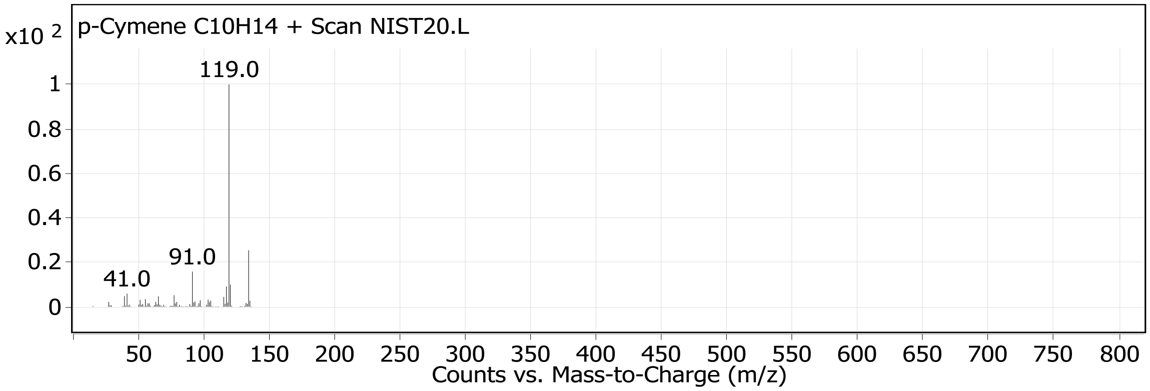

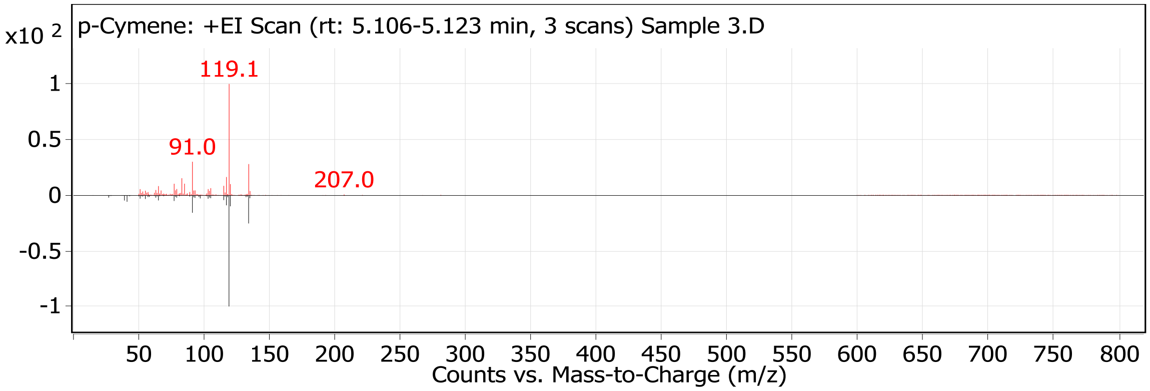

Spectrum Structure

p-Cymene

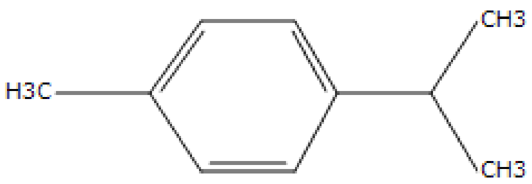

Qualitative Analysis Report

**Spectrum Source**	**Collision Energy**	**Ionization Mode**
Peak (2) in "+ TIC Scan"	0	EI

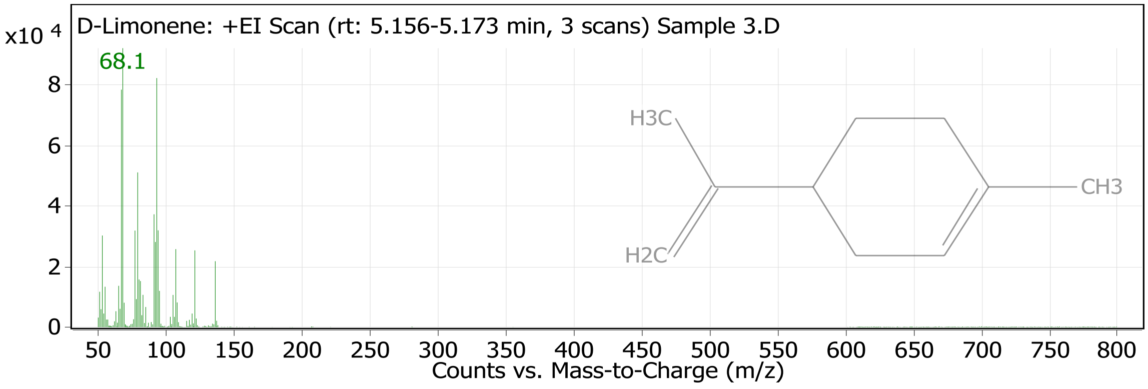

Library Spectrum

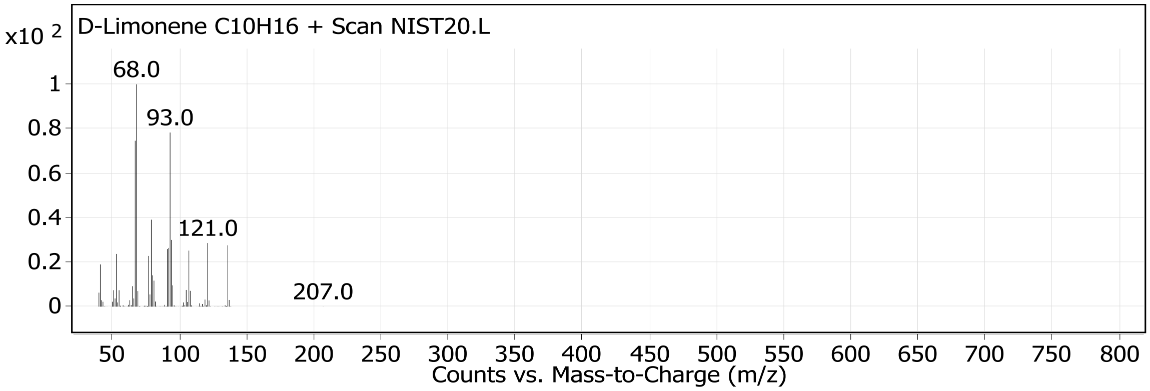

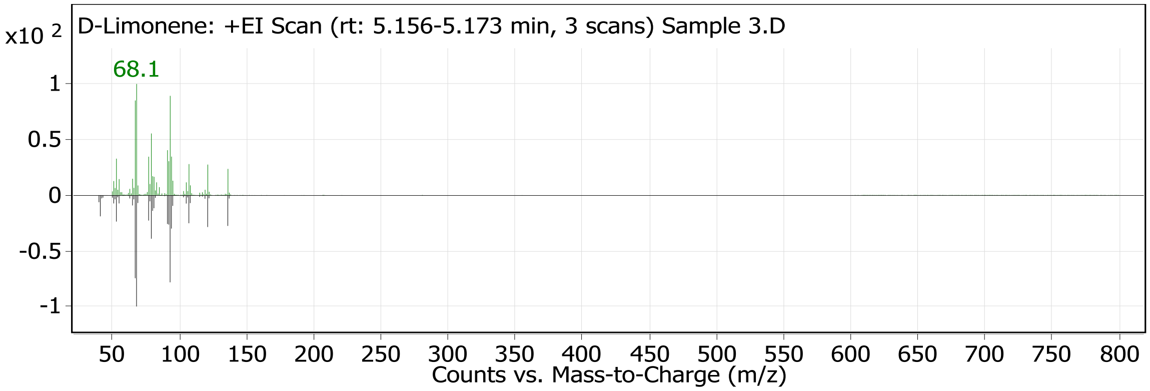

Spectrum Structure

D-Limonene

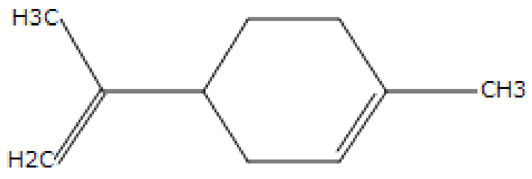

Spectrum Source	Collision Energy	Ionization Mode
Peak (3) in "+ TIC Scan"	0	EI

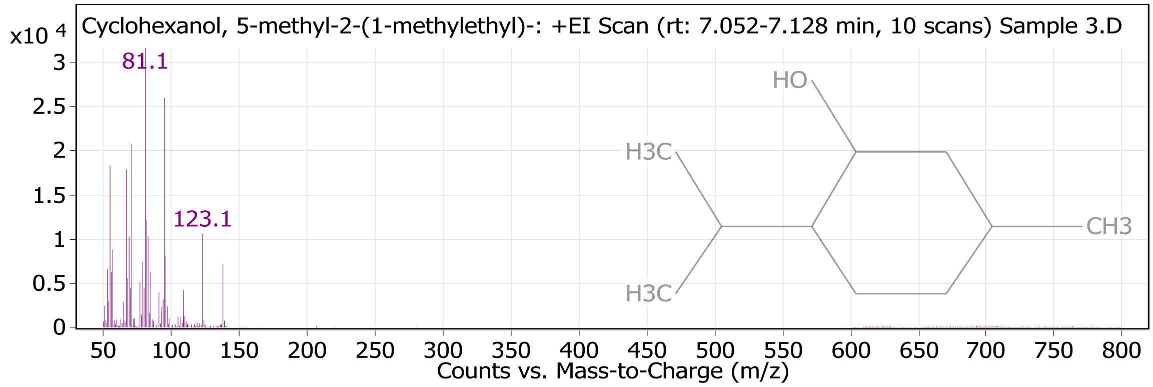

Library Spectrum

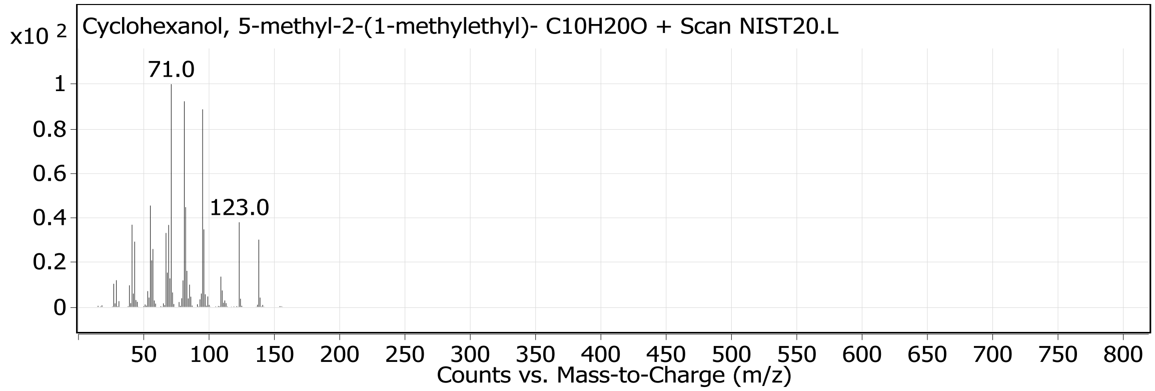

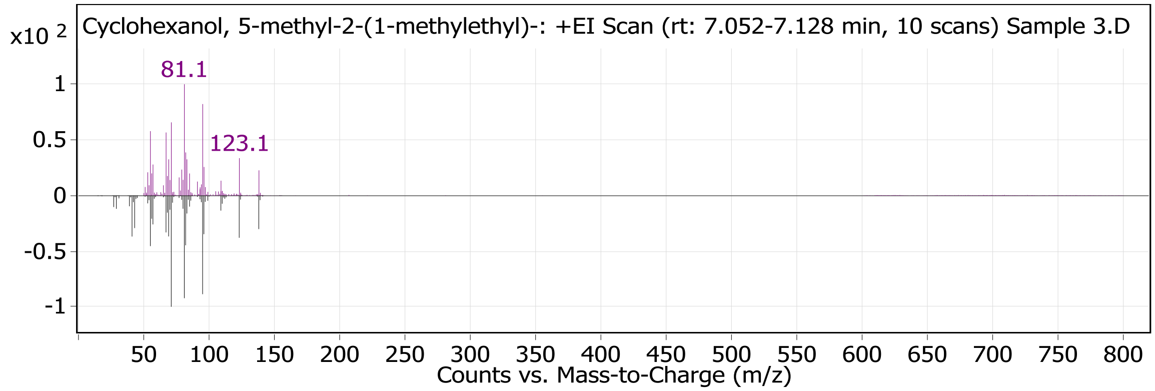

Spectrum Structure

Cyclohexanol, 5-methyl-2-(1-methylethyl)-

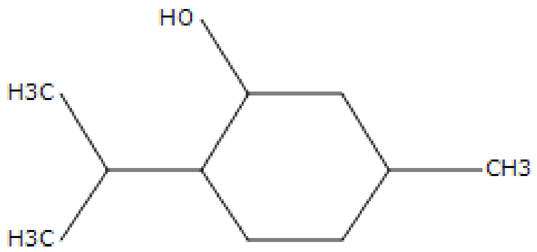

Spectrum Source	Collision Energy	Ionization Mode
Peak (4) in "+ TIC Scan"	0	EI

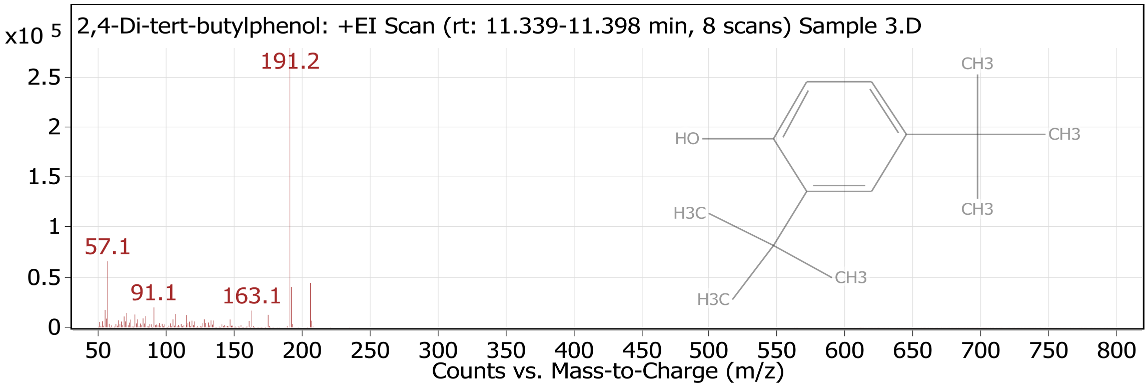

Library Spectrum

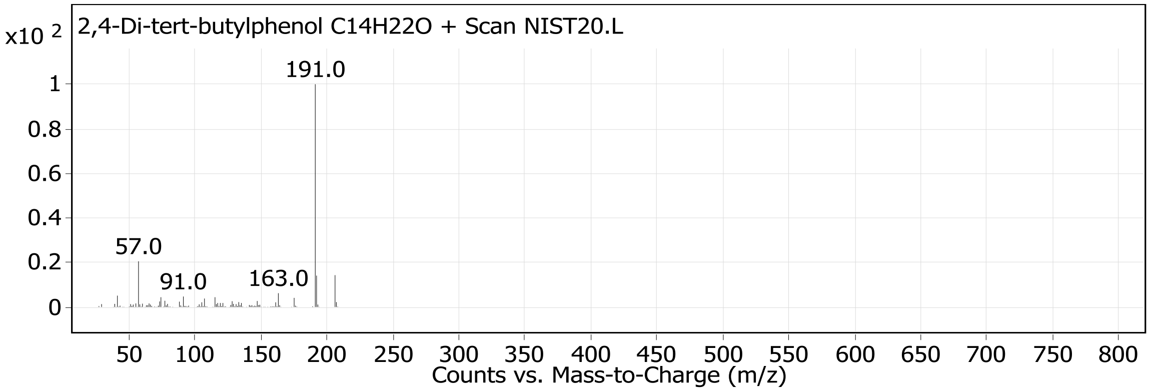

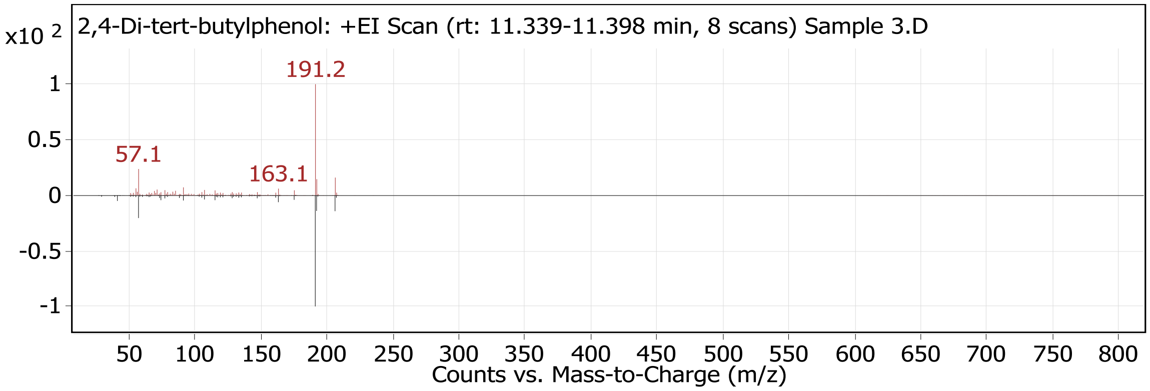

Spectrum Structure

2,4-Di-tert-butylphenol

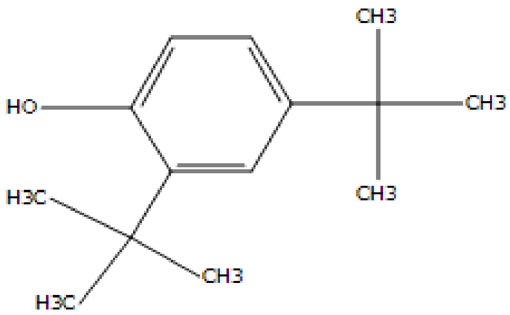

**Spectrum Source**	**Collision Energy**	**Ionization Mode**
Peak (5) in "+ TIC Scan"	0	EI

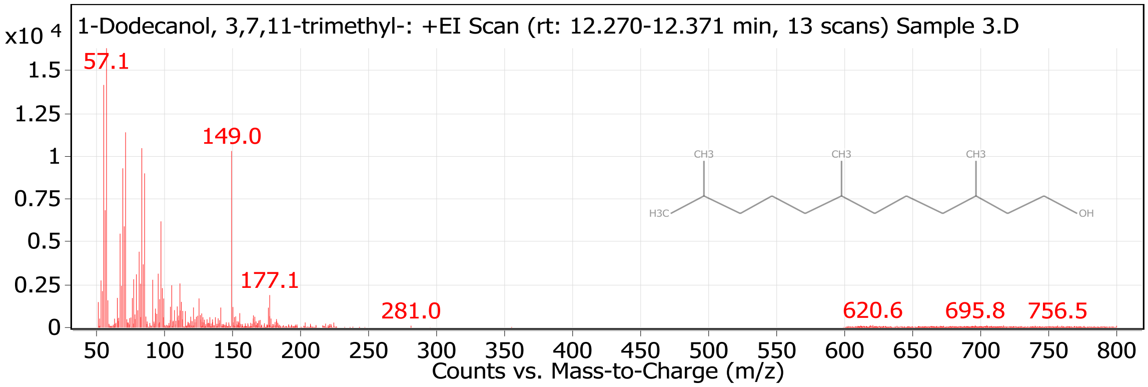

Library Spectrum

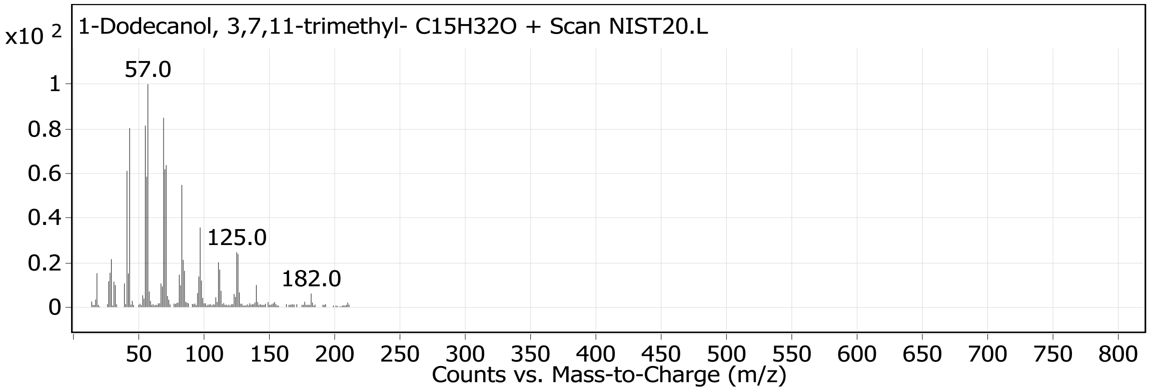

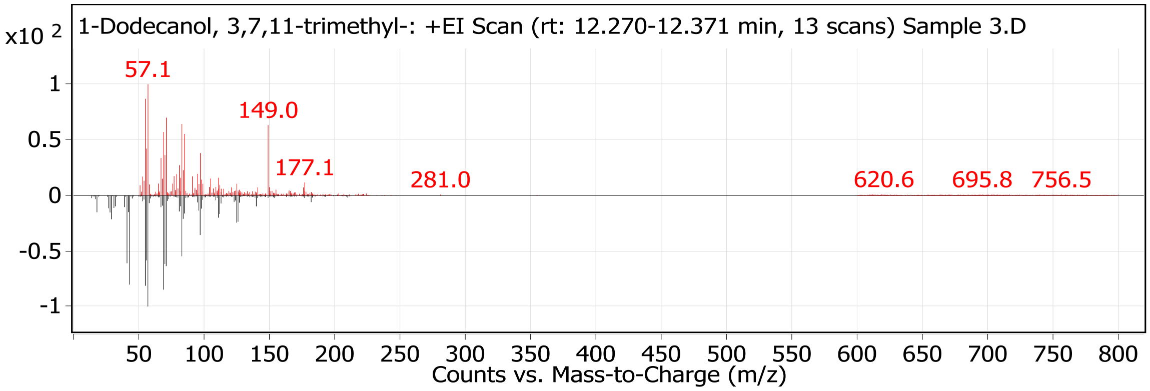

Spectrum Structure

1-Dodecanol, 3,7,11-trimethyl-

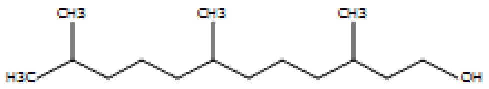

**Spectrum Source**	**Collision Energy**	**Ionization Mode**
Peak (6) in "+ TIC Scan"	0	EI

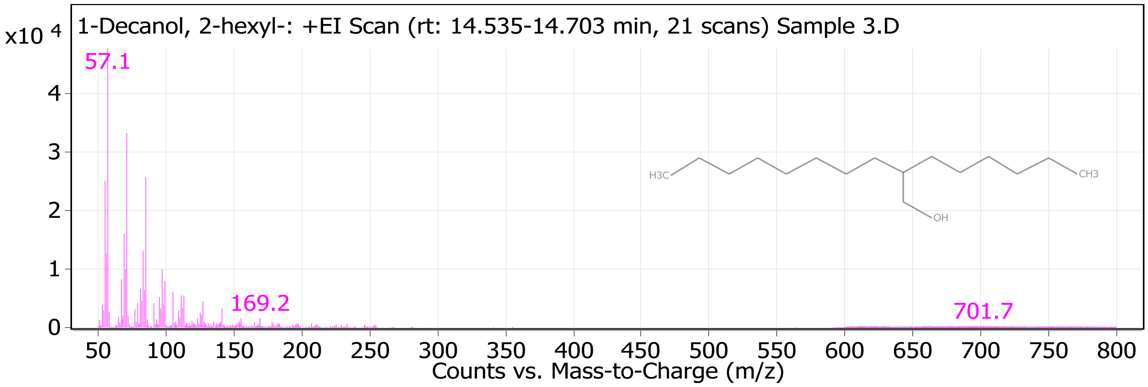

Library Spectrum

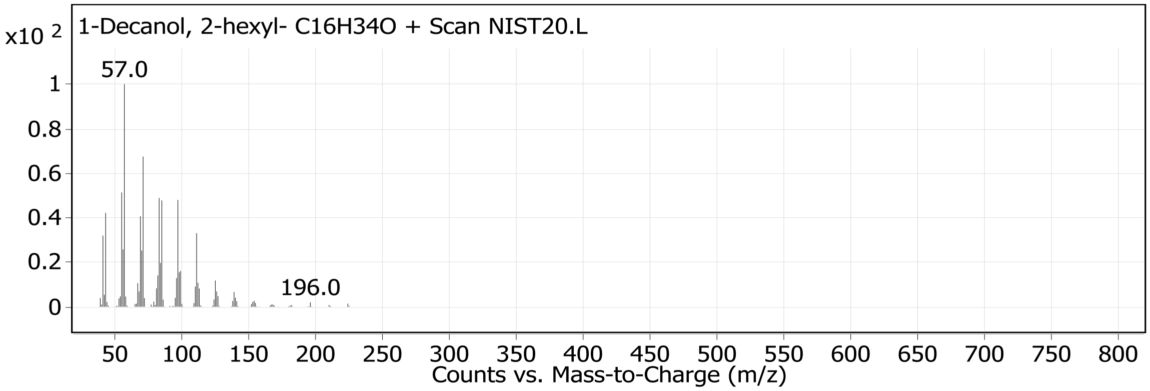

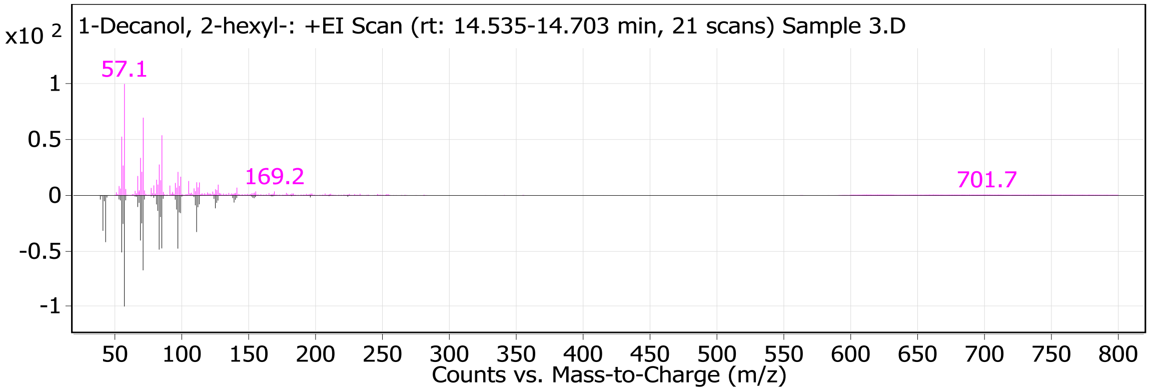

Spectrum Structure

1-Decanol, 2-hexyl-

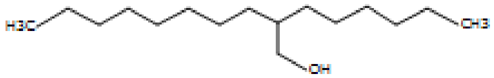

Qualitative Analysis Report

Spectrum Source	Collision Energy	Ionization Mode
Peak (7) in "+ TIC Scan"	0	EI

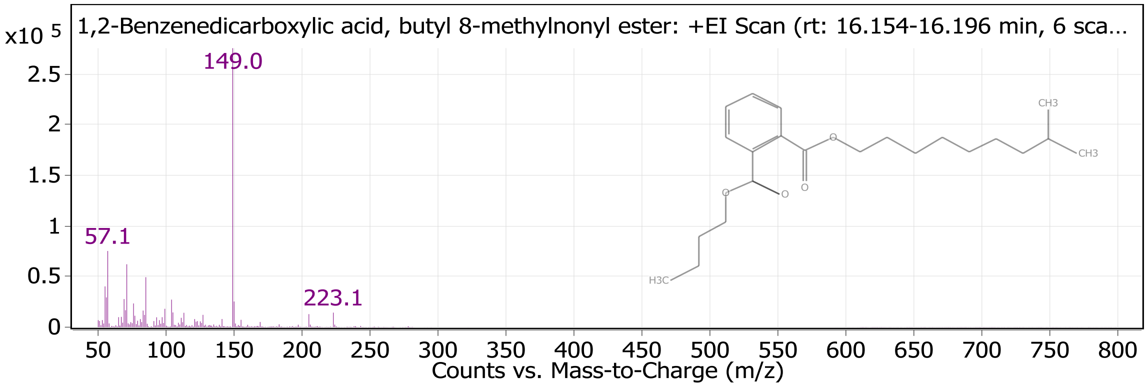

Library Spectrum

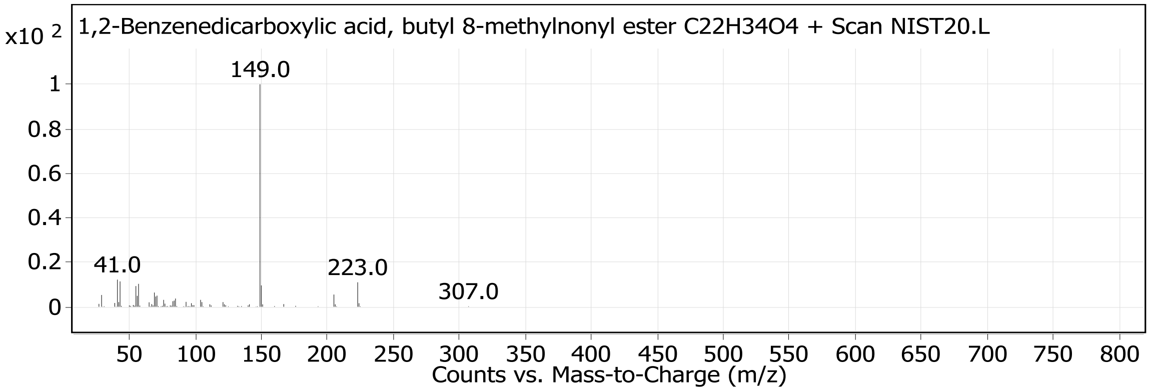

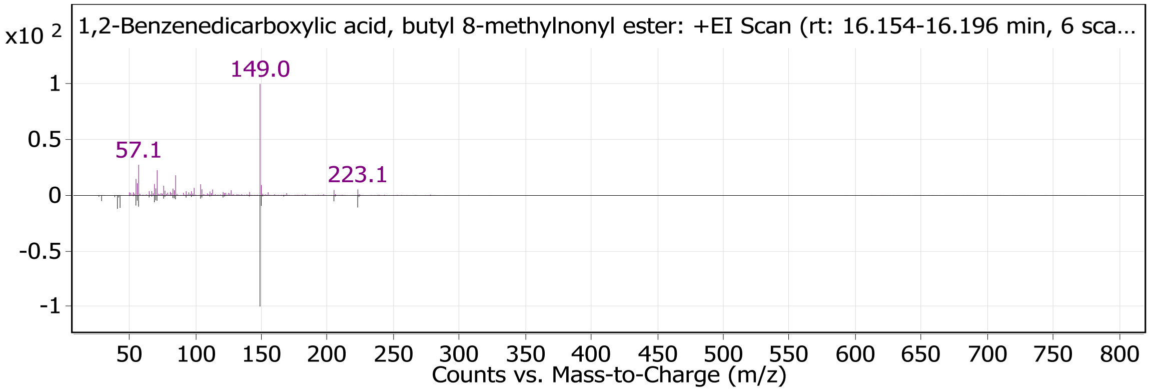

Spectrum Structure

1,2-Benzenedicarboxylic acid, butyl 8-methylnonyl ester

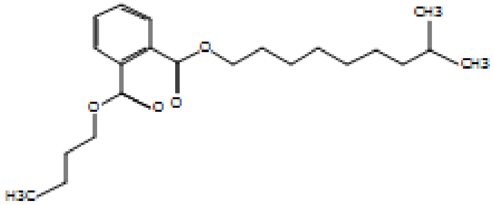

Spectrum Source	Collision Energy	Ionization Mode
Peak (8) in "+ TIC Scan"	0	EI

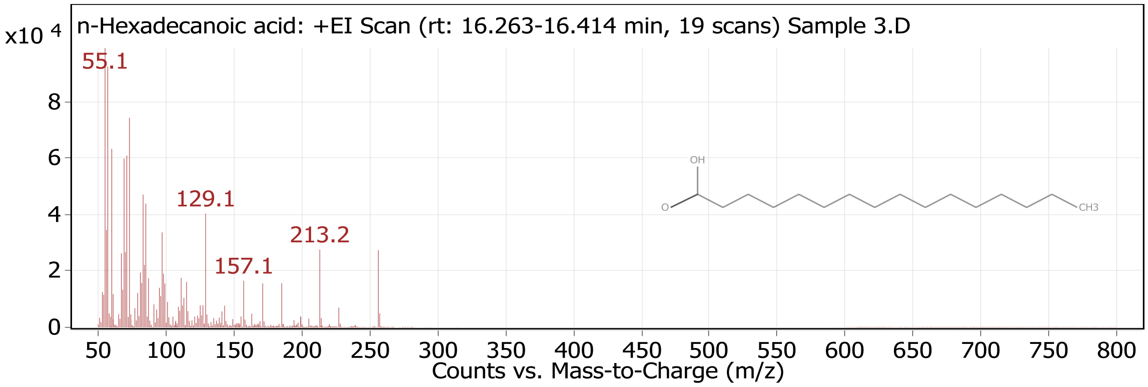

Library Spectrum

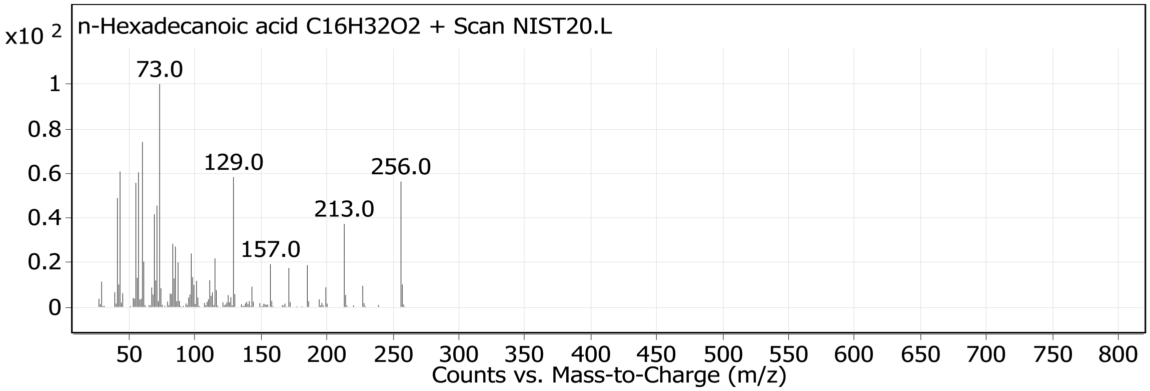

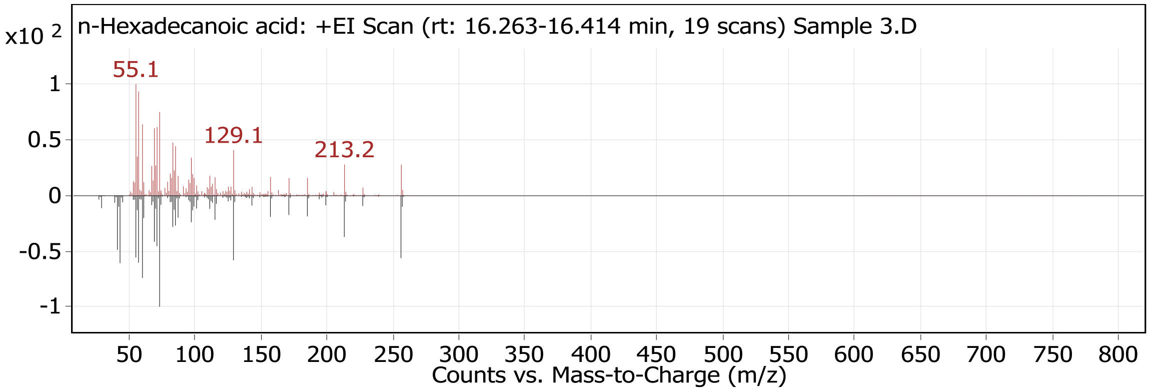

Qualitative Analysis Report

Spectrum Structure

n-Hexadecanoic acid

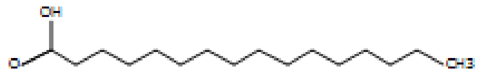

Spectrum Source	Collision Energy	Ionization Mode
Peak (9) in "+ TIC Scan"	0	EI

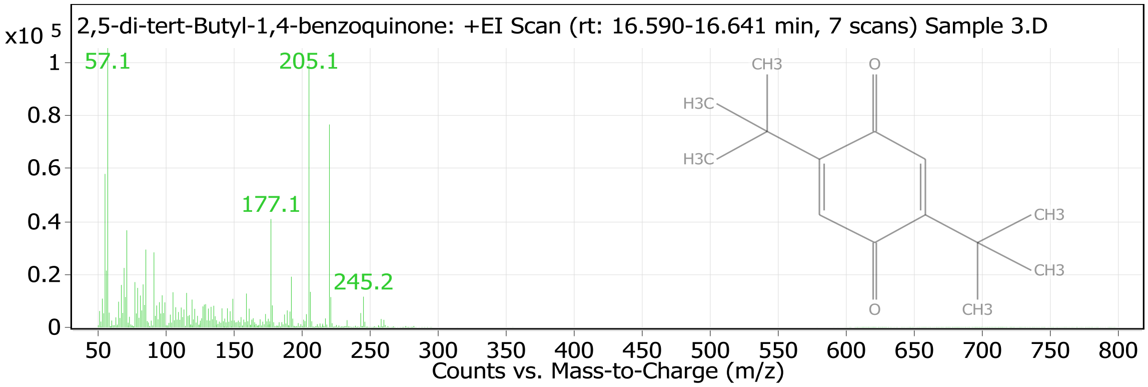

Library Spectrum

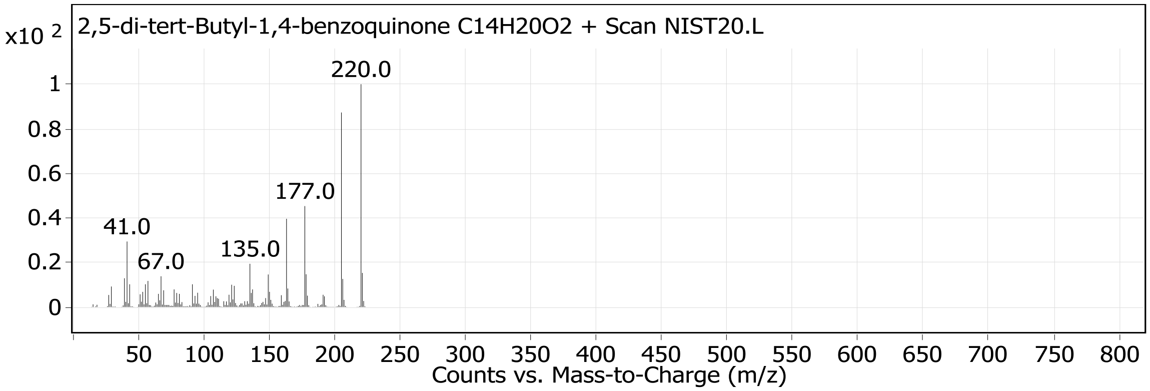

Qualitative Analysis Report

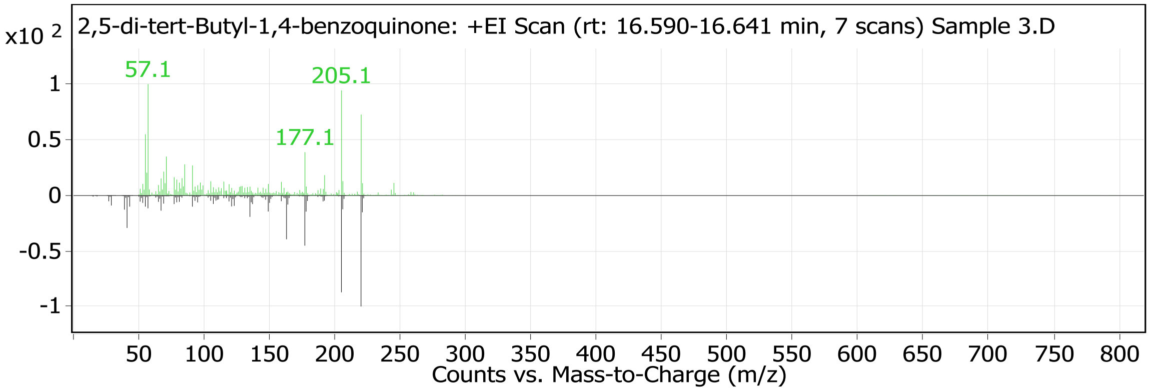

Qualitative Analysis Report

Spectrum Structure

2,5-di-tert-Butyl-1,4-benzoquinone

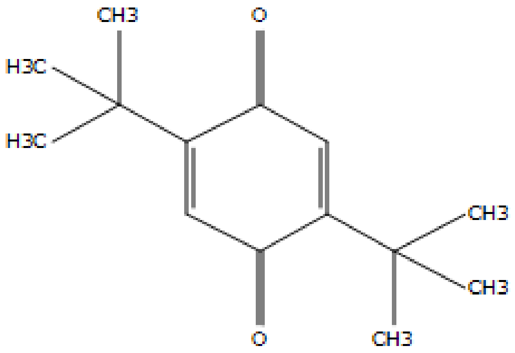

**Spectrum Source**	**Collision Energy**	**Ionization Mode**
Peak (10) in "+ TIC Scan"	0	EI

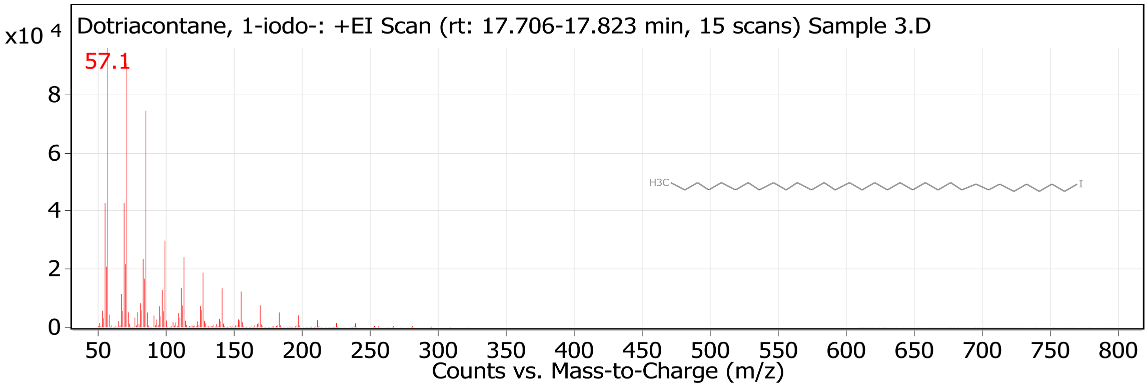

Library Spectrum

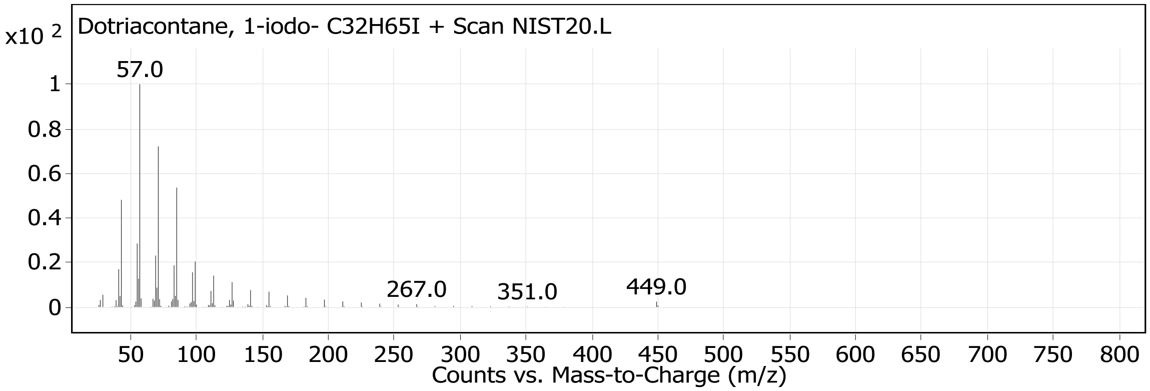

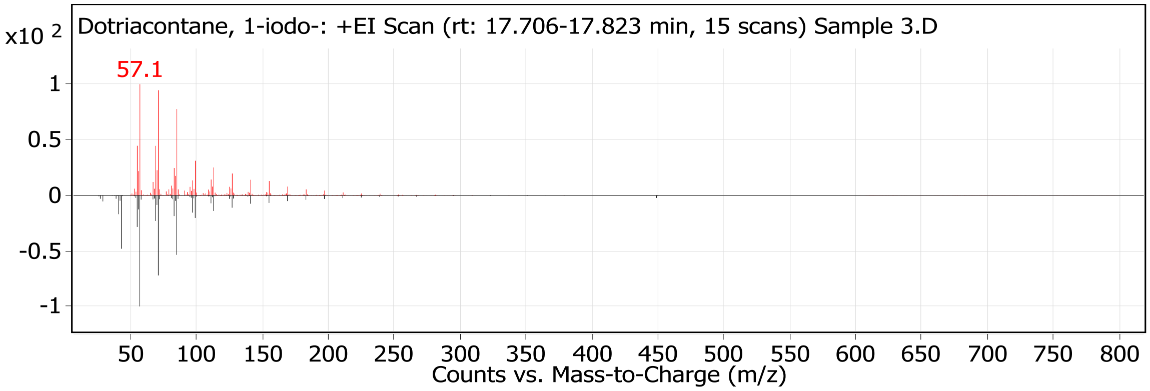

Spectrum Structure

Dotriacontane, 1-iodo-

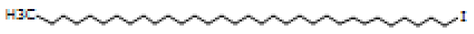

**Spectrum Source**	**Collision Energy**	**Ionization Mode**
Peak (11) in "+ TIC Scan"	0	EI

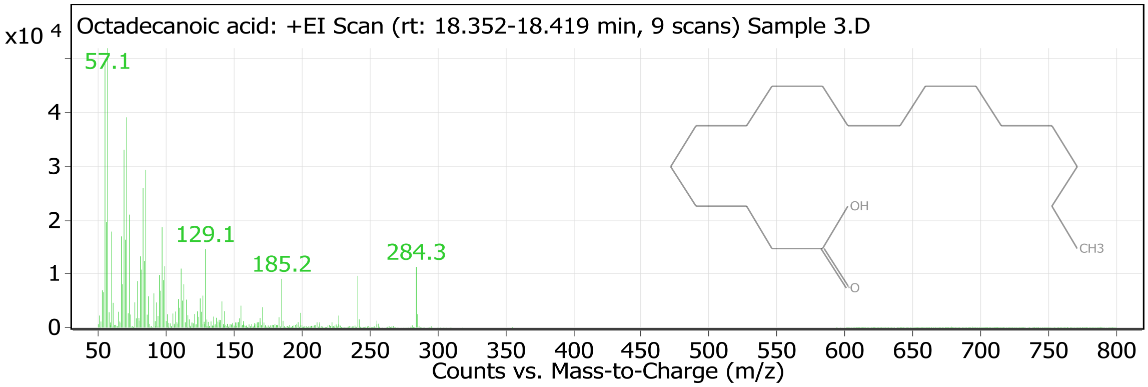

Library Spectrum

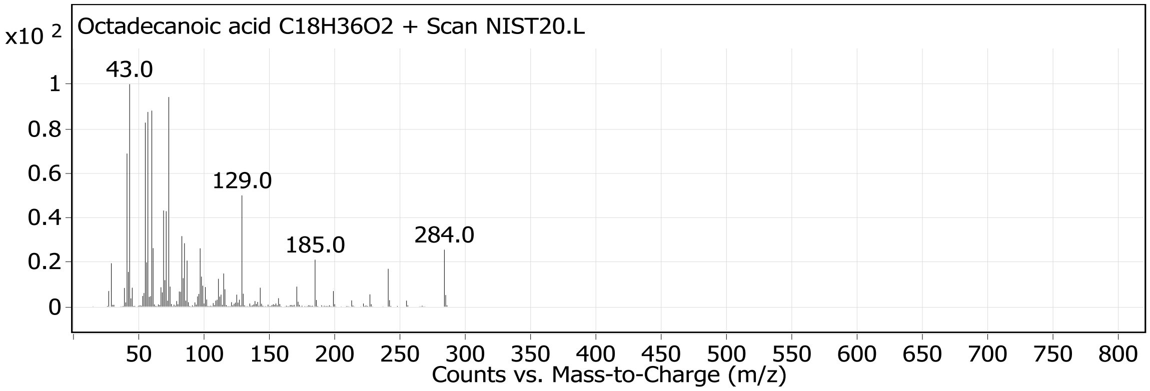

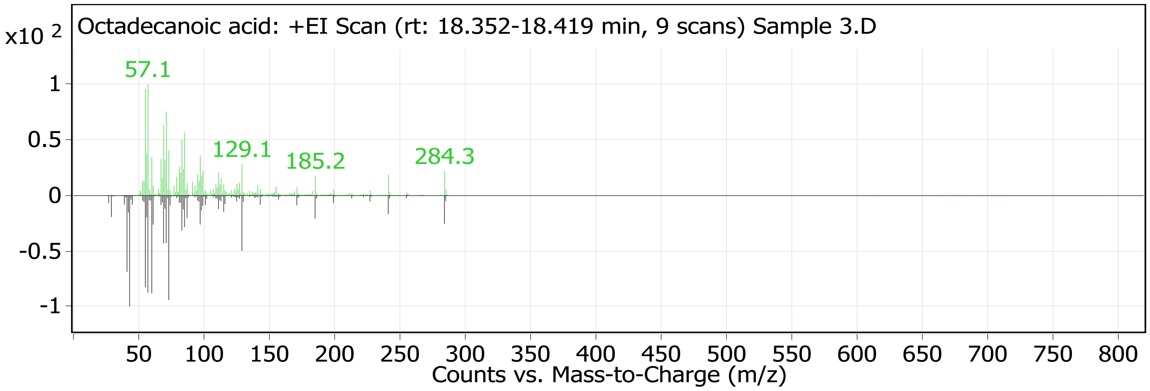

Spectrum Structure

Octadecanoic acid

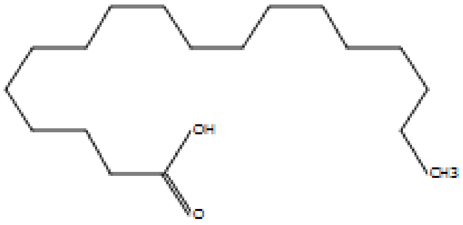

Spectrum Source	Collision Energy	Ionization Mode
Peak (12) in "+ TIC Scan"	0	EI

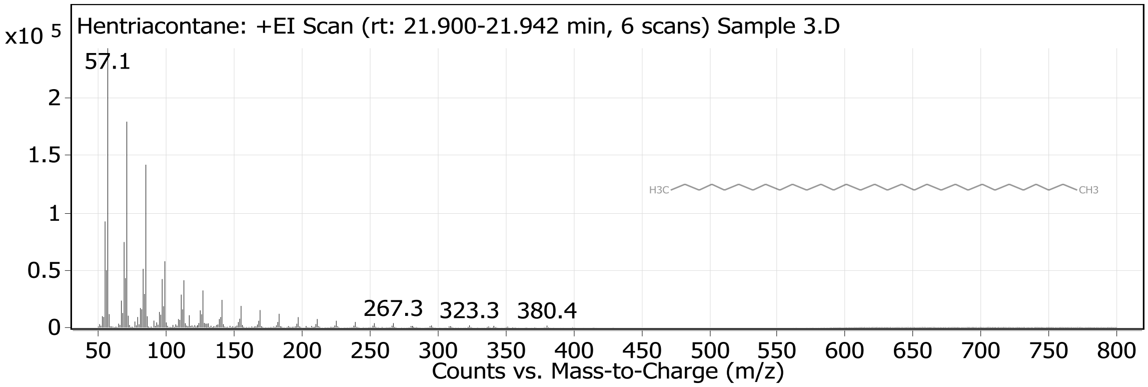

Library Spectrum

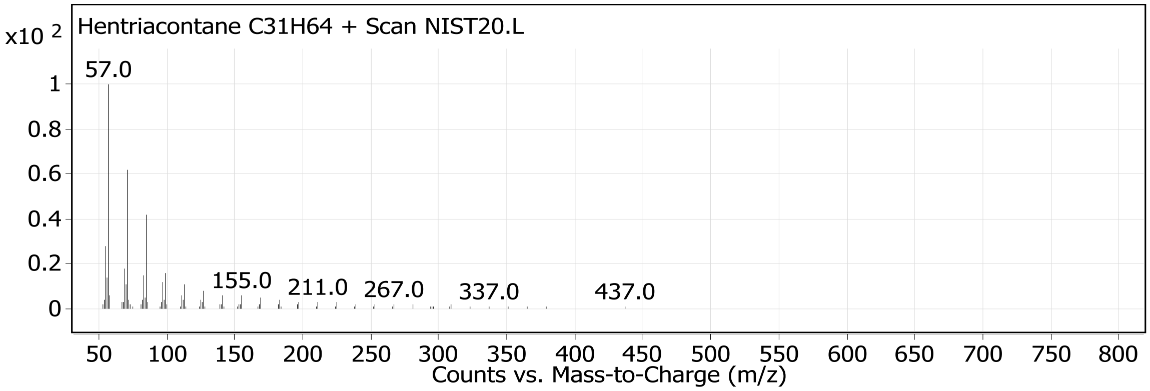

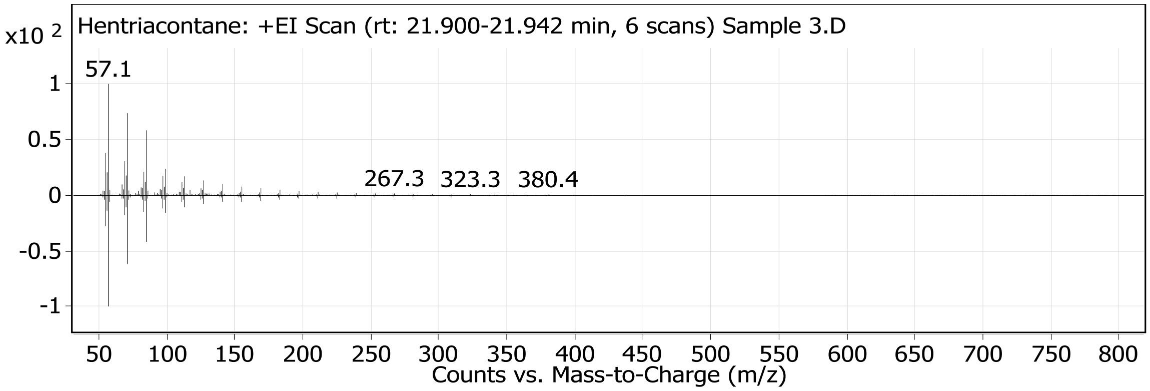

Spectrum Structure

Hentriacontane

--- End Of Report ---
