# Supplementary material for: Chemical and Molecular Insights into the Arid Wild Plant Diversity of Saudi Arabia
Source: Plants (Basel). 2026 Jan 19;15(2):295. doi: 10.3390/plants15020295 (PMC12845481; doi:10.3390/plants15020295)
Supplement: Supplementary file 1 [file plants-15-00295-s001.zip › Sample 4_AnalysisReport.pdf]

Qualitative Analysis Report

**Data Filename**	Sample 5.D	**Sample Name**	Sample 5
**Sample Type**		**Position**	1
**Instrument Name**	3	**User Name**	
**Acq Method**	Scan DB-5MS Hydrogen 2024.M	**Acquired Time**	6/24/2024 6:54:58 PM
**IRM Calibration Status**	Not Applicable	**DA Method**	SignalToNoiseCheckout.m
**Comment**			

**Expected Barcode**		**Sample Amount**	
**Dual Inj Vol**	0.2	**TuneName**	ATUNE.U
**TunePath**	D:\MassHunter\GCMS\3\5977 \	**TuneDateStamp**	2024-06-23T14:01:57+02:00
**MSFirmwareVersion**	6.00.34	**OperatorName**	
**RunCompletedFlag**	True	**Acquisition SW Version**	MassHunter GC/MS Acquisition 10.0.368 14-Feb-2019 Copyright © 1989-2018 Agilent Technologies, Inc

User Chromatograms

Fragmentor Voltage **Collision Energy** 0 **Ionization Mode** EI

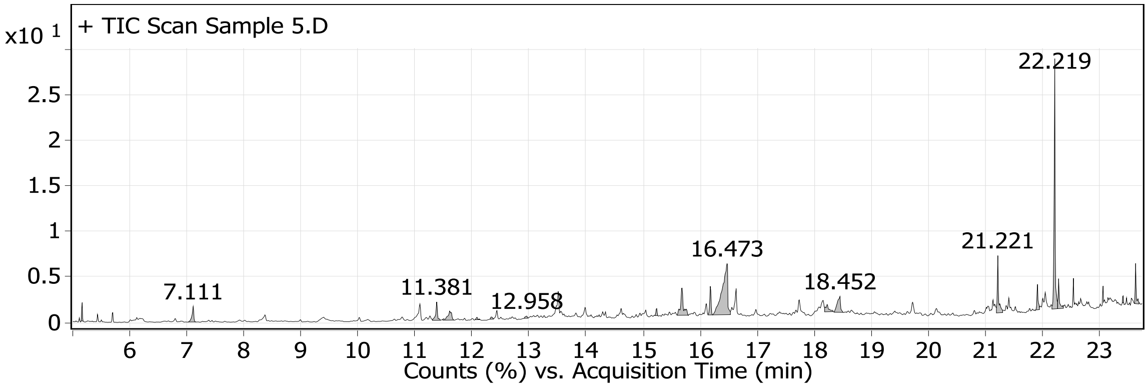

Integration Peak List

Peak	Start	RT	End	Height	Area	Area %
1	5.145	5.165	5.181	1451801.44	1297890.38	4.74
2	7.027	7.111	7.16	1208457.04	2608187.04	9.52
3	11.305	11.381	11.448	1455777.51	3130294.25	11.43
4	11.49	11.607	11.682	710402.56	3327003.81	12.15
5	12.052	12.085	12.143	189367.44	380921.81	1.39
6	12.313	12.337	12.379	172862.09	393804.21	1.44
7	12.909	12.958	12.983	171060.31	435793.4	1.59
8	15.206	15.24	15.259	551787.61	819506.74	2.99
9	15.6	15.684	15.79	2143186.29	7490201.51	27.34
10	16.137	16.179	16.219	2254384.32	3632179.09	13.26
11	16.222	16.473	16.531	4022992.05	27391913.6	100
12	18.184	18.226	18.334	577985.63	2252666.11	8.22
13	18.352	18.452	18.509	1246492.73	5048856.82	18.43
14	21.196	21.221	21.305	4515786.8	7000434.43	25.56
15	21.883	21.917	21.951	2008268.83	2794225.16	10.2
16	22.169	22.219	22.269	19741058.12	24639827.35	89.95
17	22.269	22.286	22.37	2360228.39	3531297.24	12.89
18	23.403	23.419	23.427	541487.43	393820.44	1.44
19	23.62	23.637	23.695	3183654.18	3690450.54	13.47

User Spectra

Spectrum Source	Collision Energy	Ionization Mode
Peak (1) in "+ TIC Scan"	0	EI

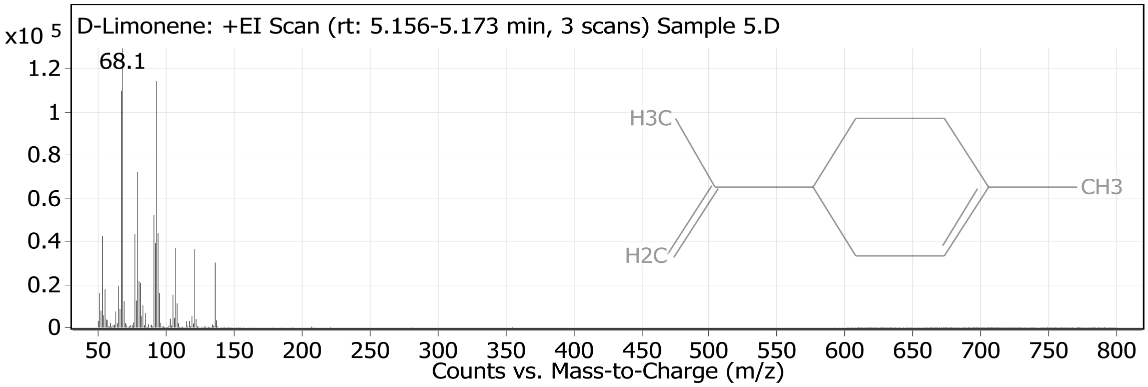

Library Spectrum

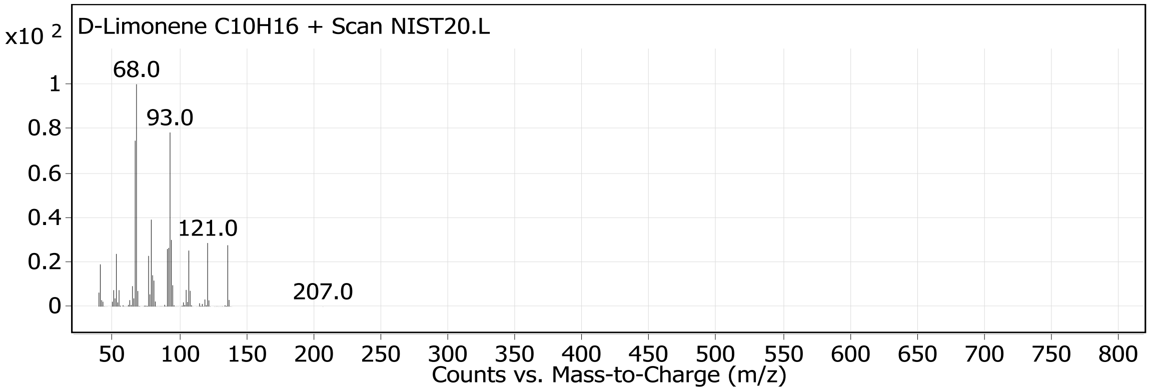

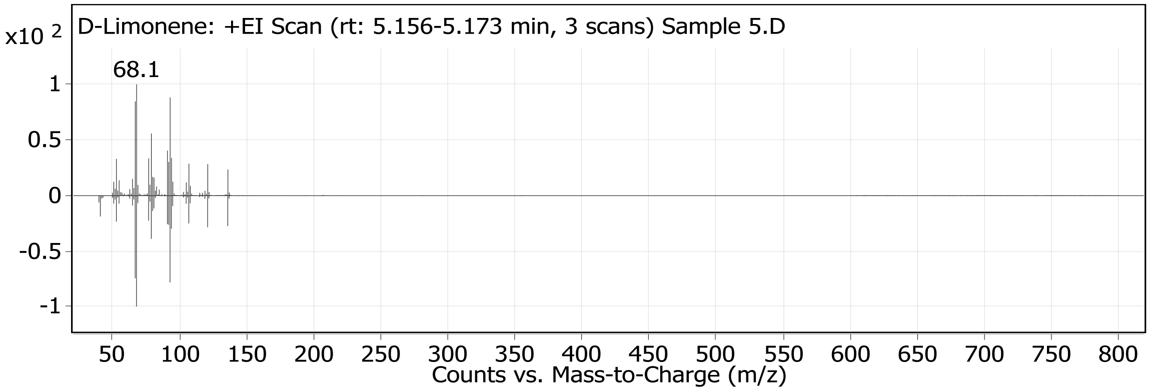

Spectrum Structure

D-Limonene

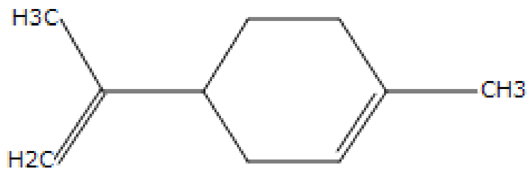

**Spectrum Source**	**Collision Energy**	**Ionization Mode**
Peak (2) in "+ TIC Scan"	0	EI

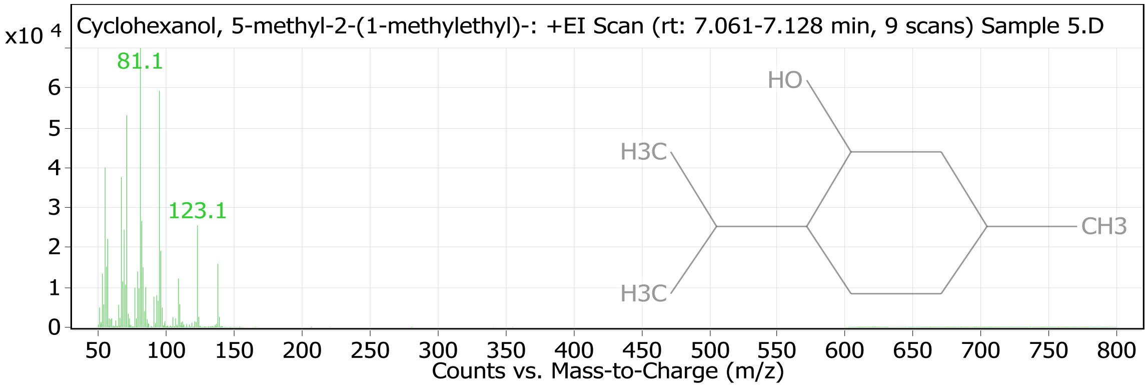

Library Spectrum

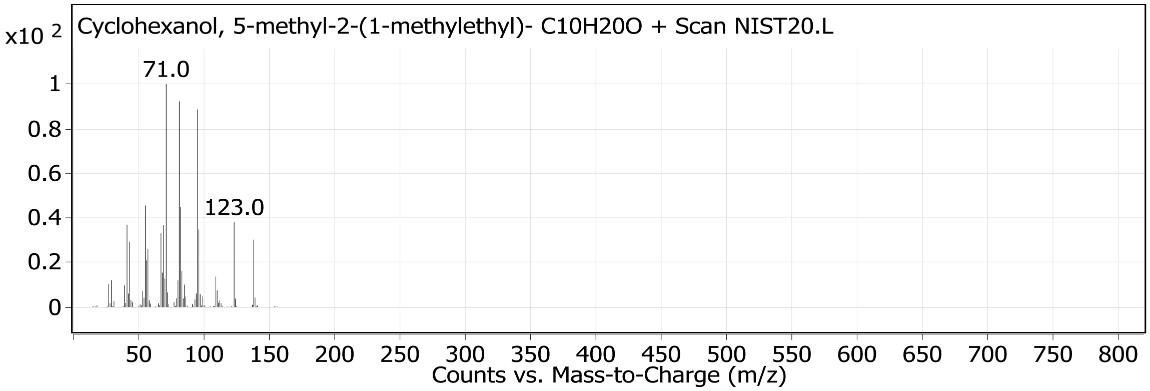

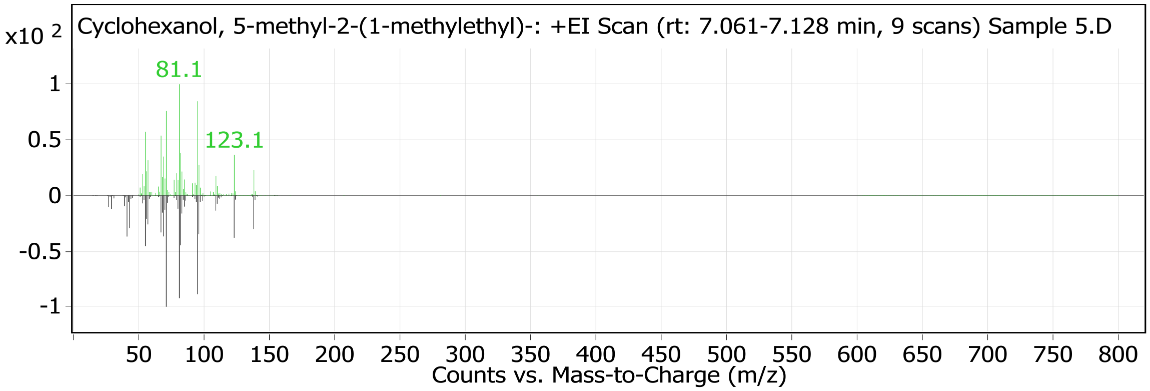

Spectrum Structure

Cyclohexanol, 5-methyl-2-(1-methylethyl)-

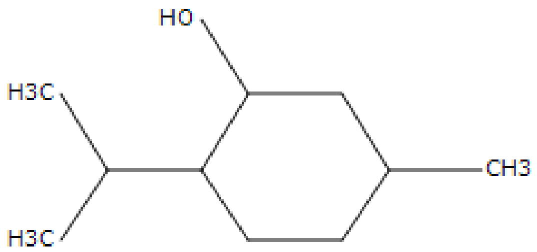

**Spectrum Source**	**Collision Energy**	**Ionization Mode**
Peak (3) in "+ TIC Scan"	0	EI

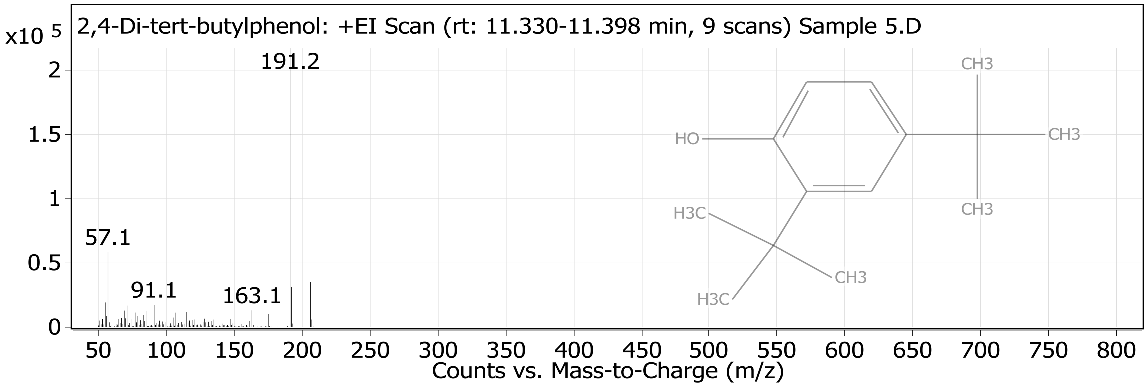

Library Spectrum

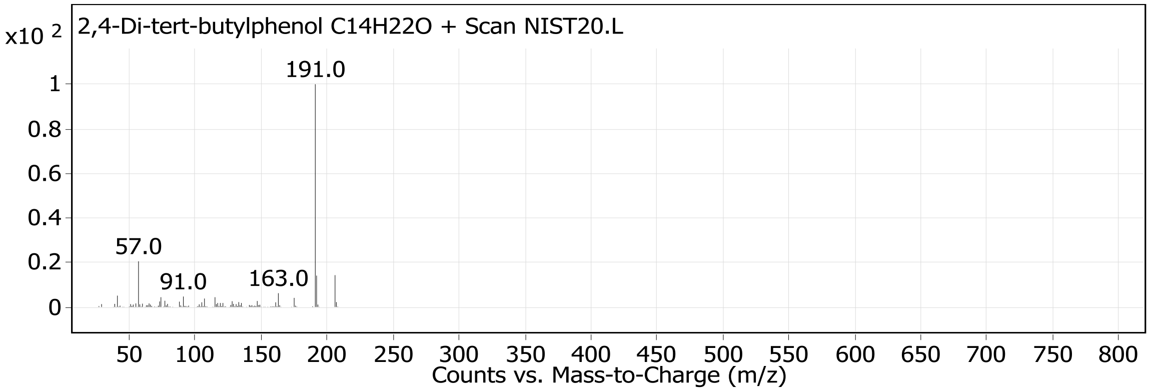

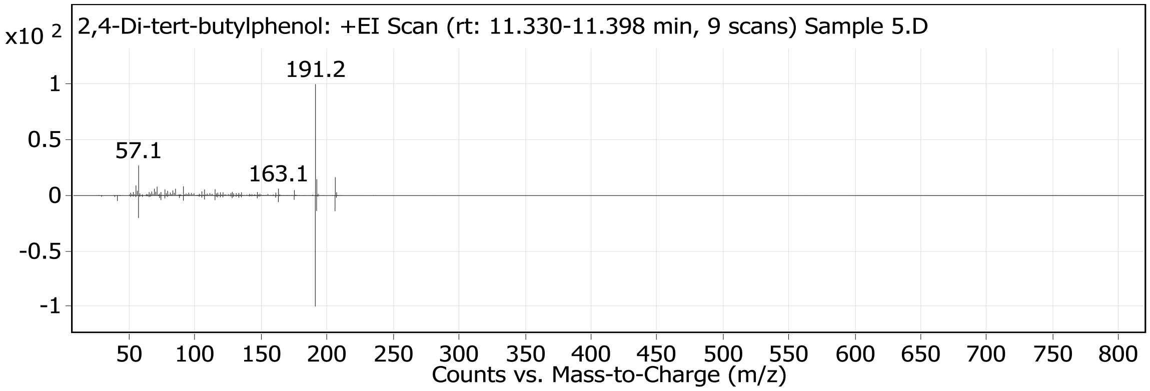

Spectrum Structure

2,4-Di-tert-butylphenol

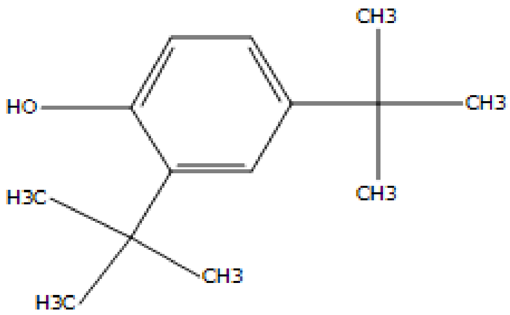

**Spectrum Source**	**Collision Energy**	**Ionization Mode**
Peak (4) in "+ TIC Scan"	0	EI

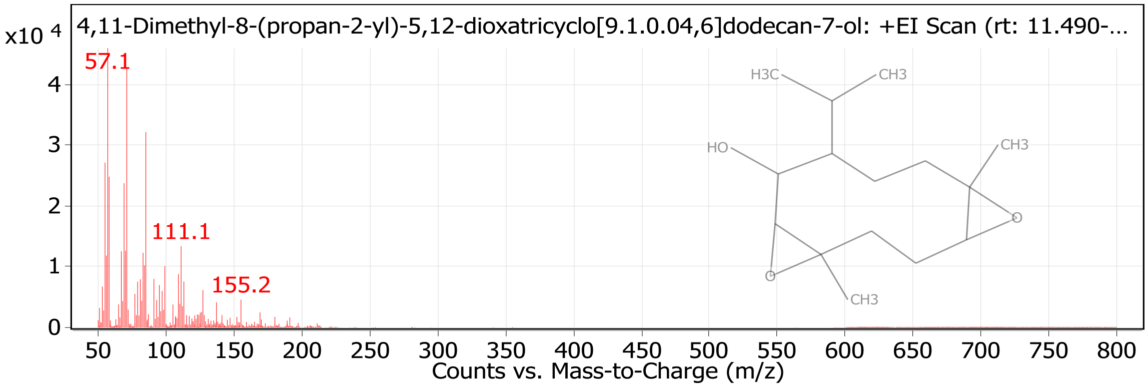

Library Spectrum

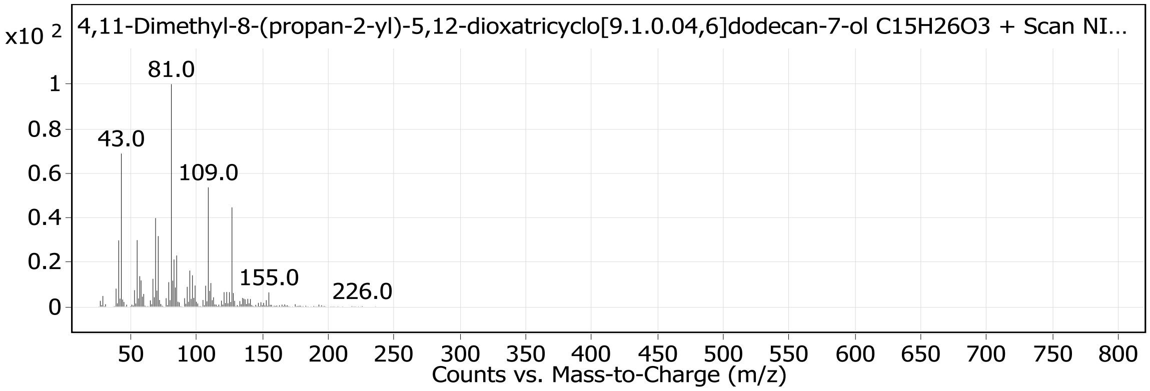

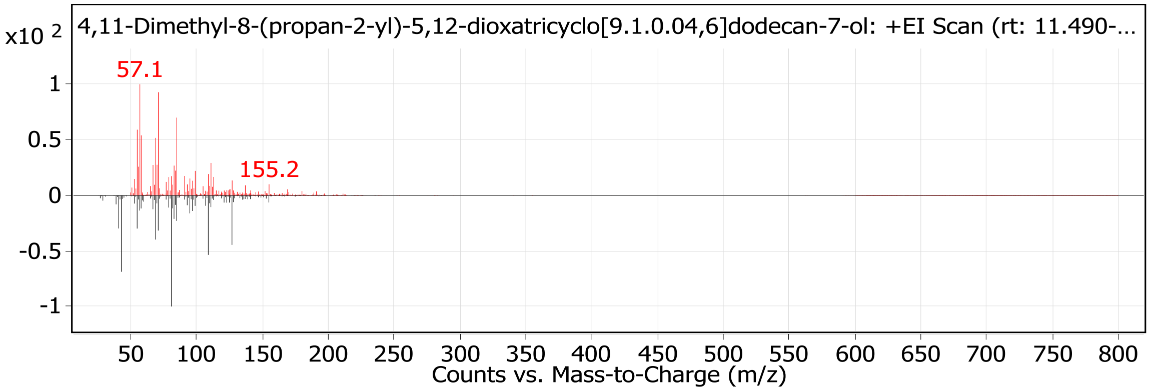

Spectrum Structure

4,11-Dimethyl-8-(propan-2-yl)-5,12-dioxatricyclo[9.1.0.04,6]dodecan-7-ol

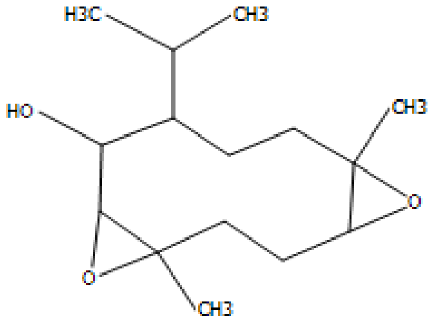

Spectrum Source	Collision Energy	Ionization Mode
Peak (5) in "+ TIC Scan"	0	EI

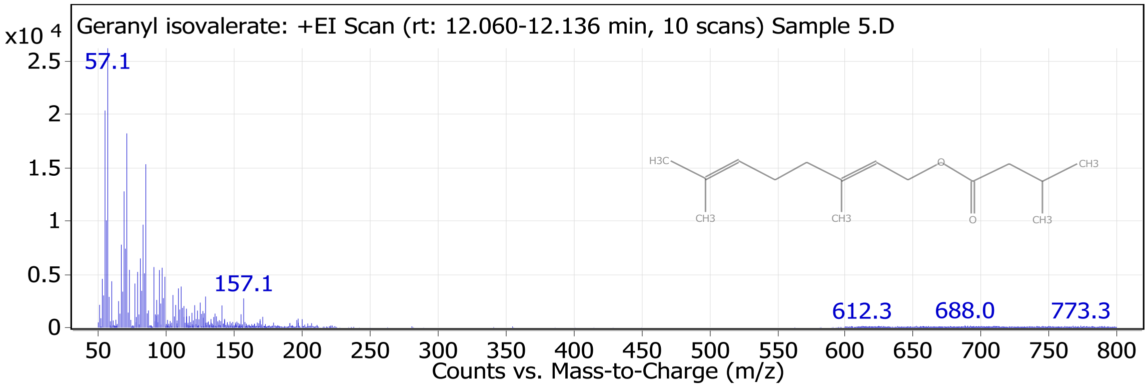

Library Spectrum

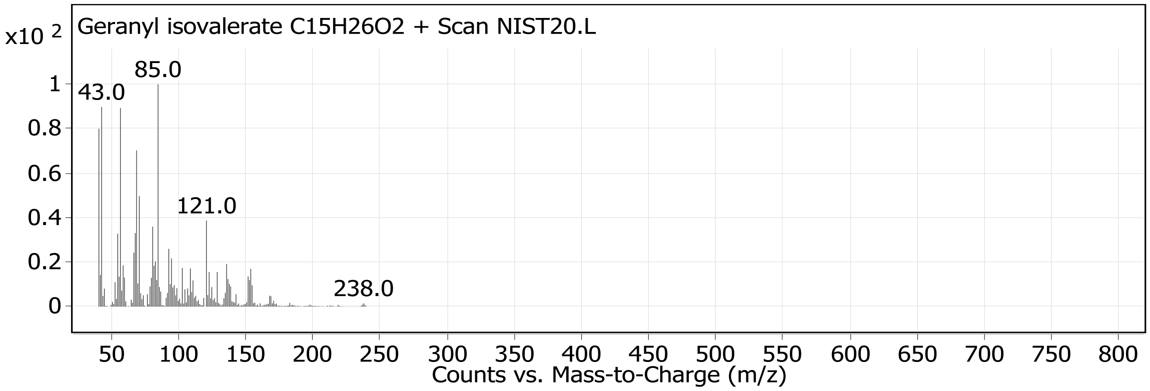

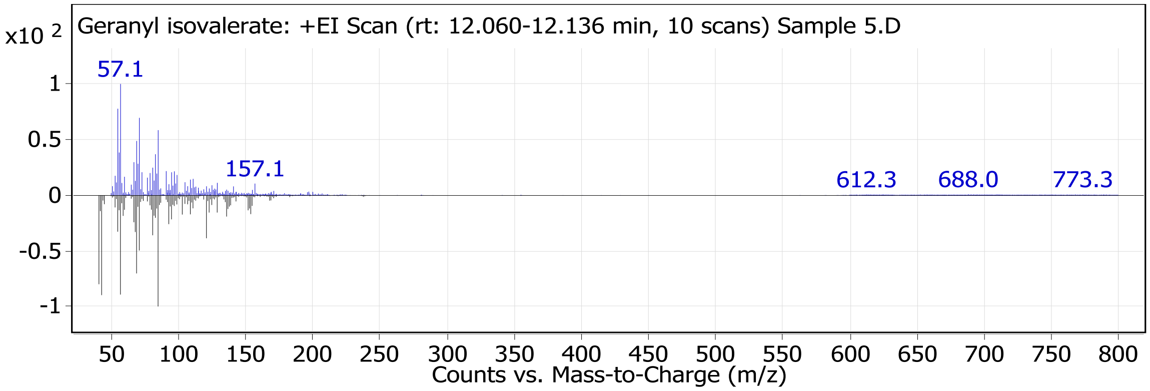

Spectrum Structure

Geranyl isovalerate

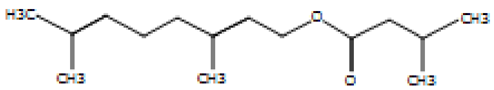

Spectrum Source	Collision Energy	Ionization Mode
Peak (6) in "+ TIC Scan"	0	EI

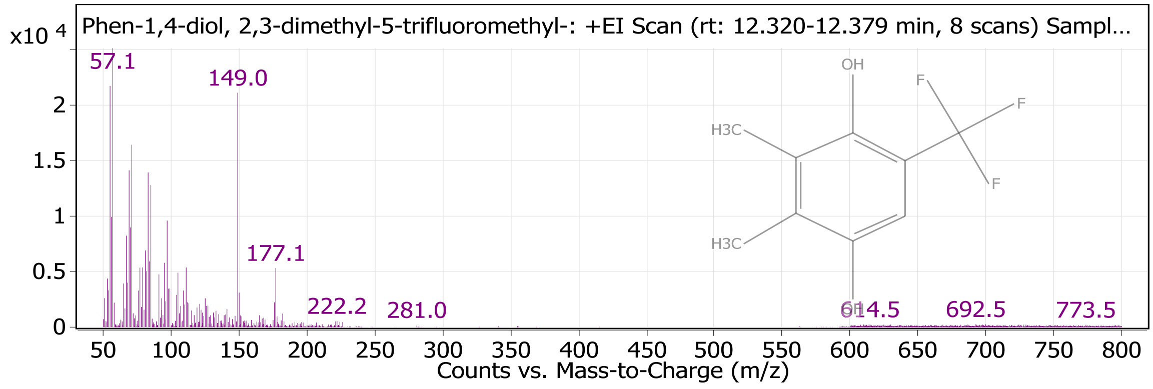

Library Spectrum

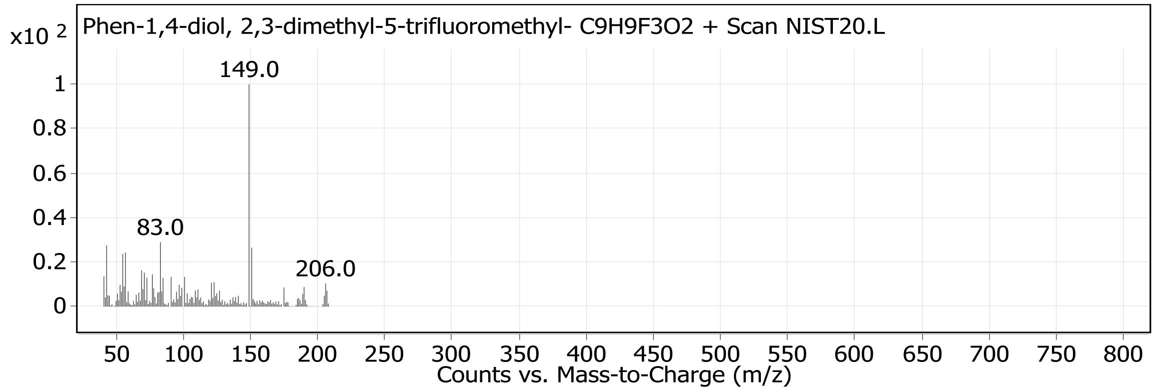

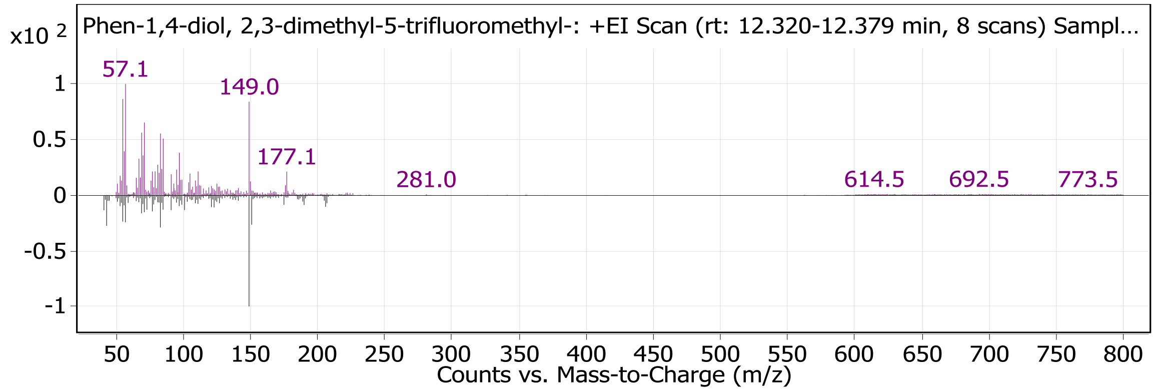

Spectrum Structure

Phen-1,4-diol, 2,3-dimethyl-5-trifluoromethyl-

Qualitative Analysis Report

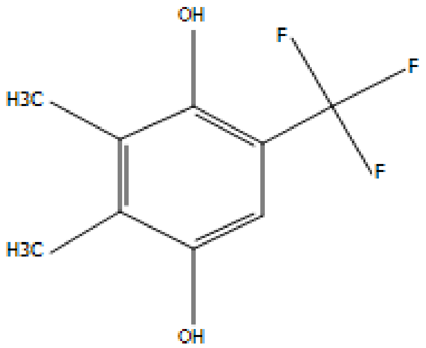

Spectrum Source	Collision Energy	Ionization Mode
Peak (7) in "+ TIC Scan"	0	EI

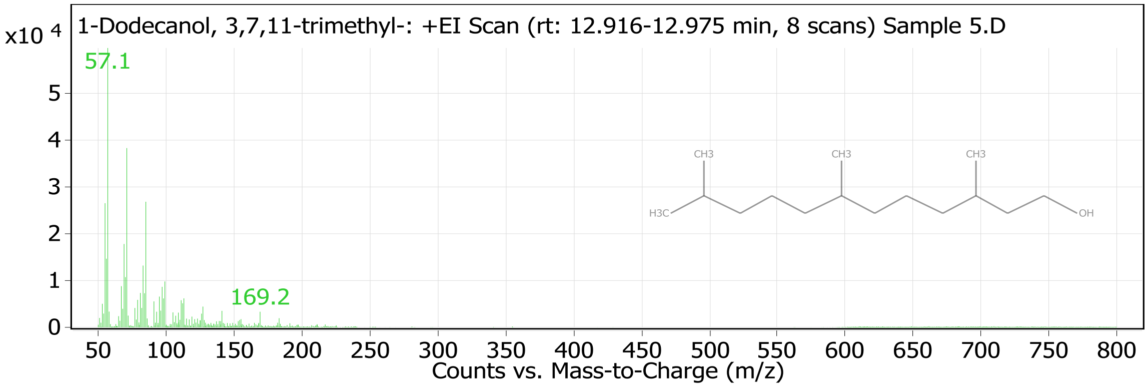

Library Spectrum

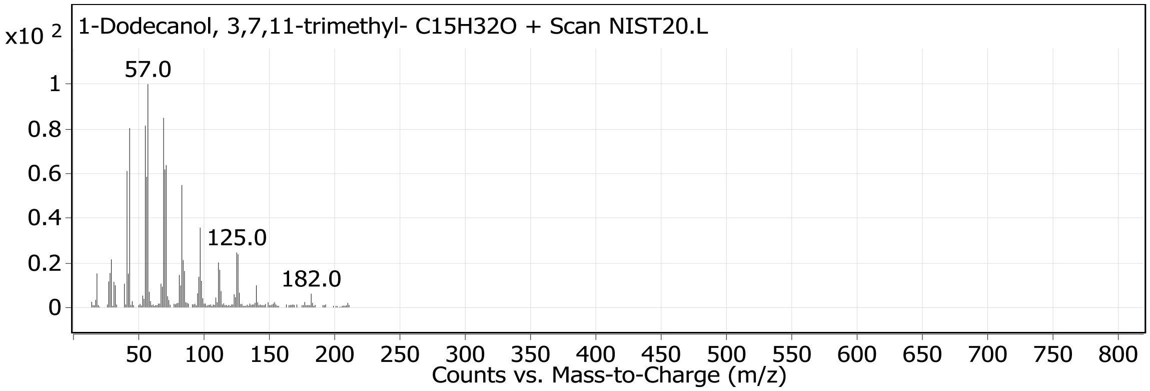

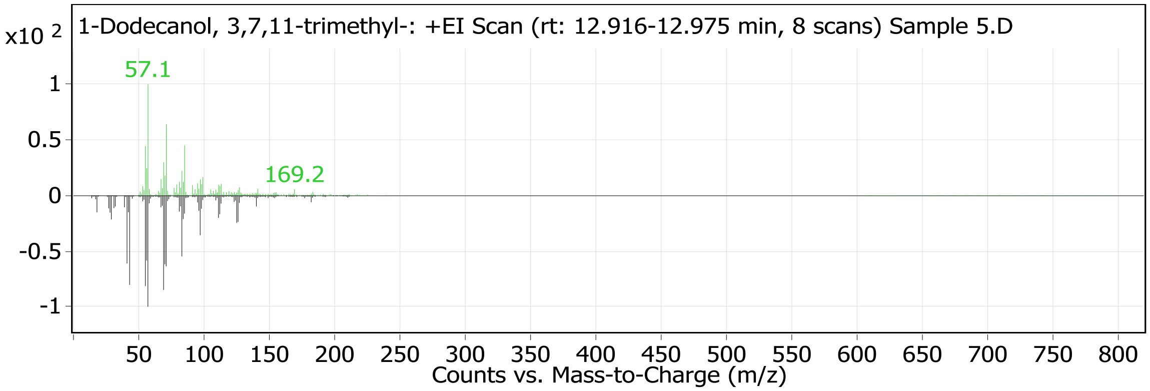

Spectrum Structure

1-Dodecanol, 3,7,11-trimethyl-

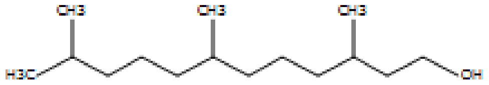

**Spectrum Source**	**Collision Energy**	**Ionization Mode**
Peak (8) in "+ TIC Scan"	0	EI

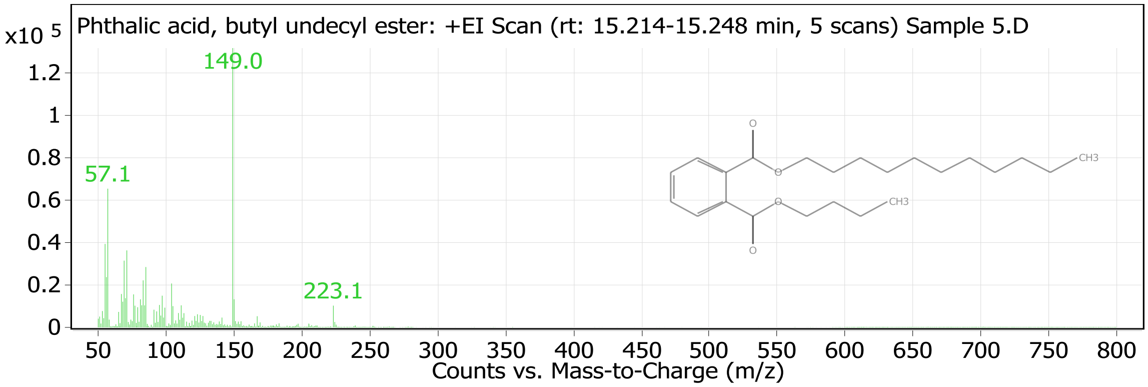

Library Spectrum

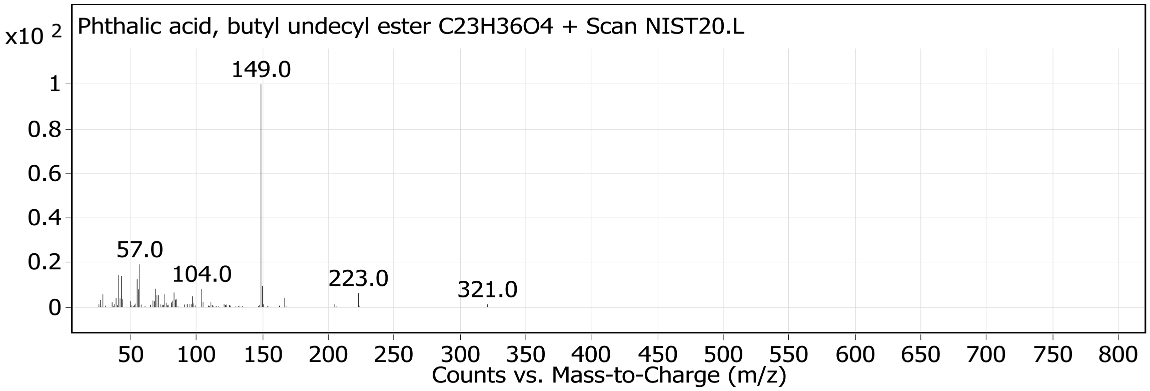

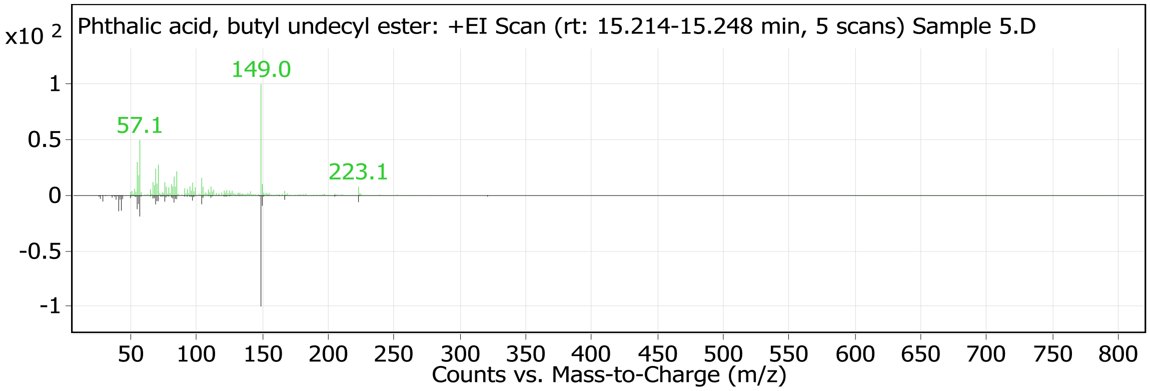

Spectrum Structure

Phthalic acid, butyl undecyl ester

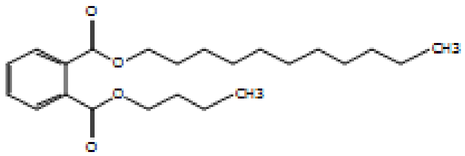

**Spectrum Source**	**Collision Energy**	**Ionization Mode**
Peak (9) in "+ TIC Scan"	0	EI

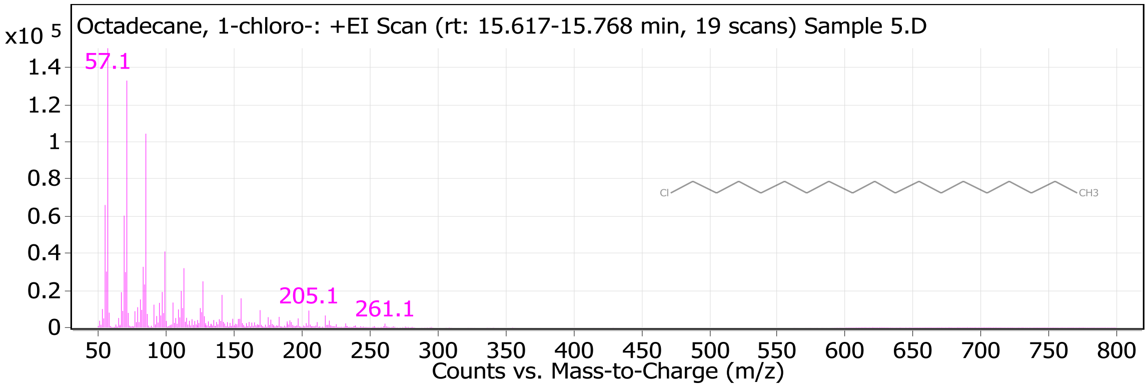

Library Spectrum

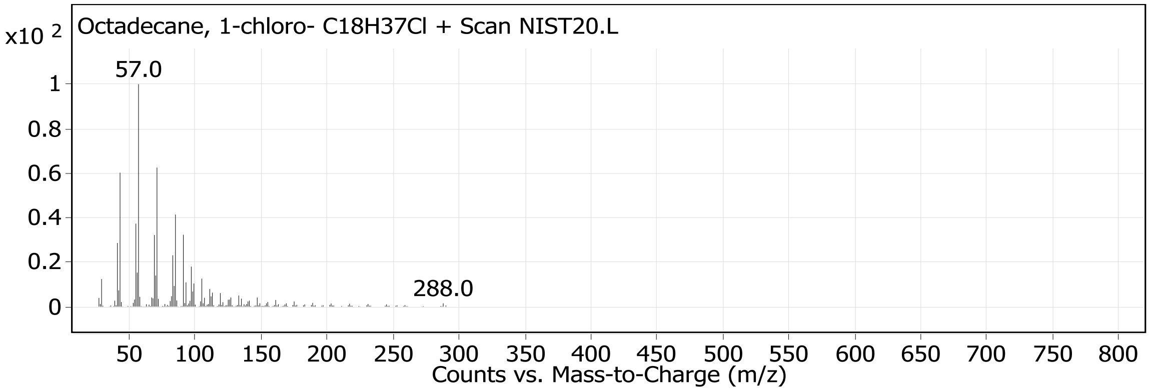

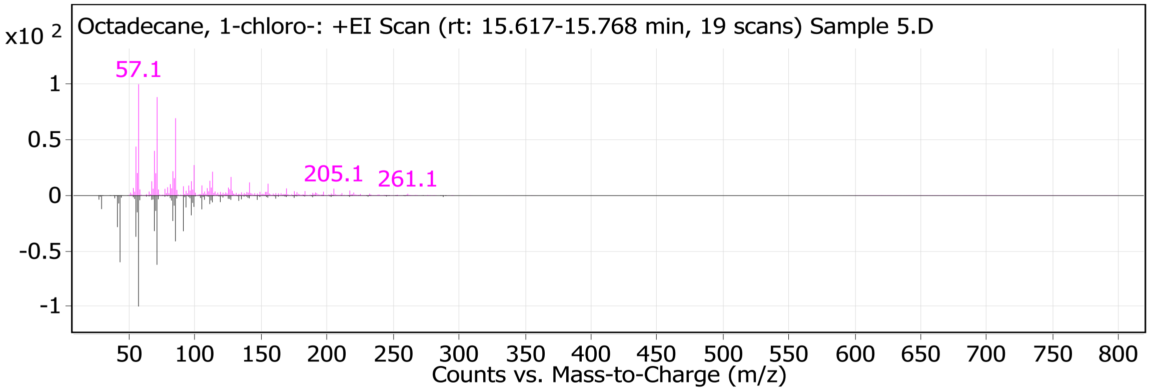

Spectrum Structure

Octadecane, 1-chloro-

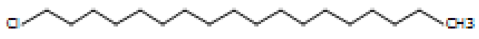

**Spectrum Source**	**Collision Energy**	**Ionization Mode**
Peak (10) in "+ TIC Scan"	0	EI

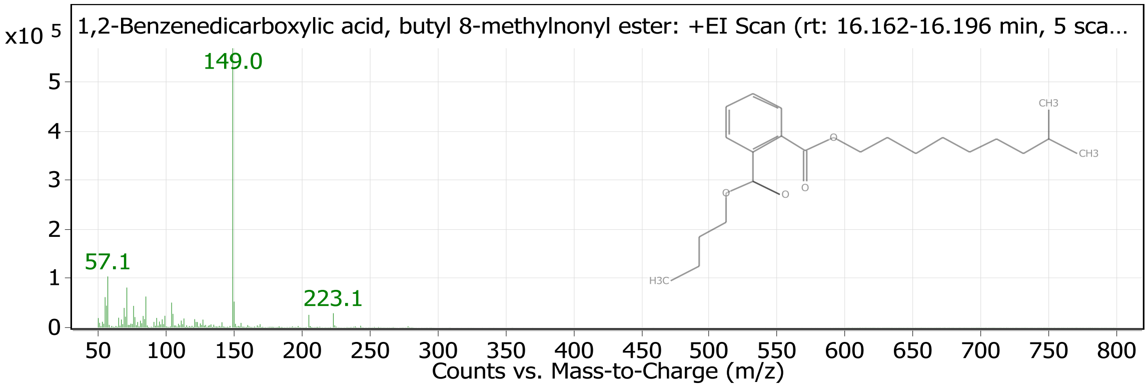

Library Spectrum

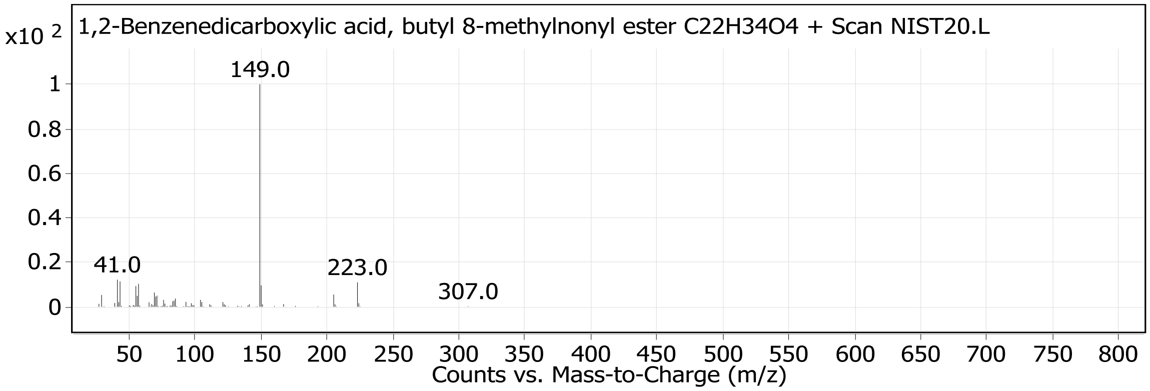

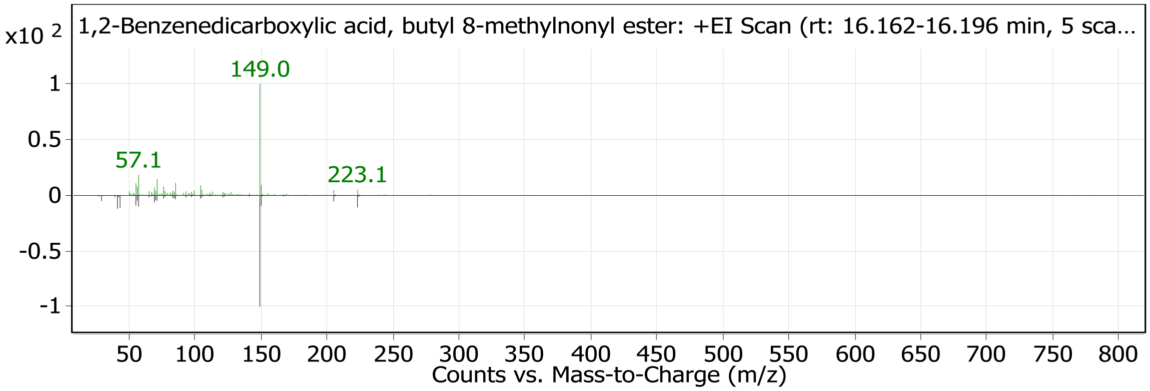

Spectrum Structure

1,2-Benzenedicarboxylic acid, butyl 8-methylnonyl ester

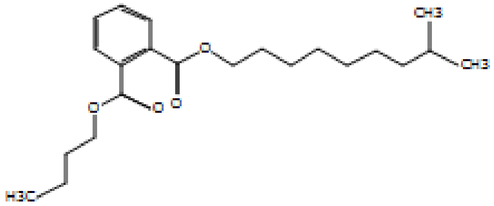

Spectrum Source	Collision Energy	Ionization Mode
Peak (11) in "+ TIC Scan"	0	EI

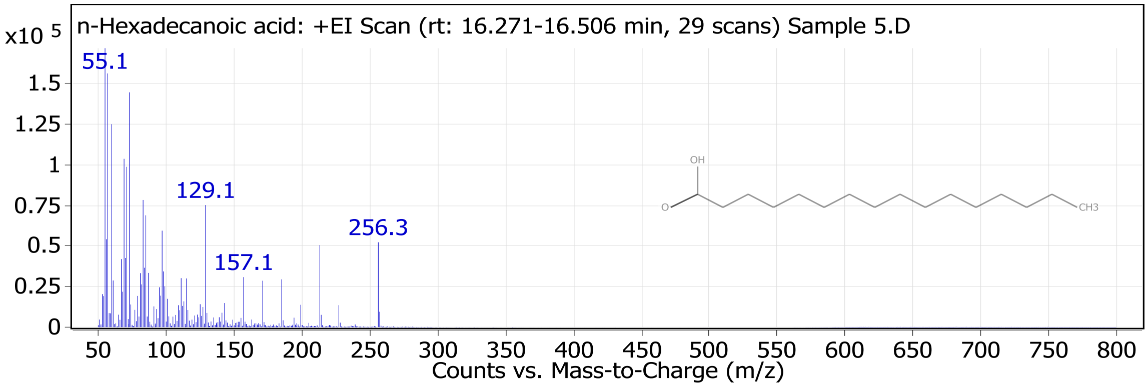

Library Spectrum

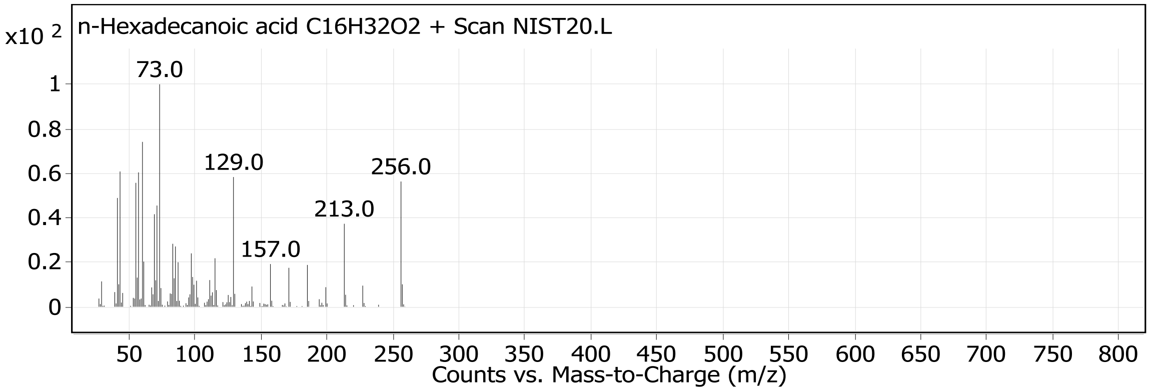

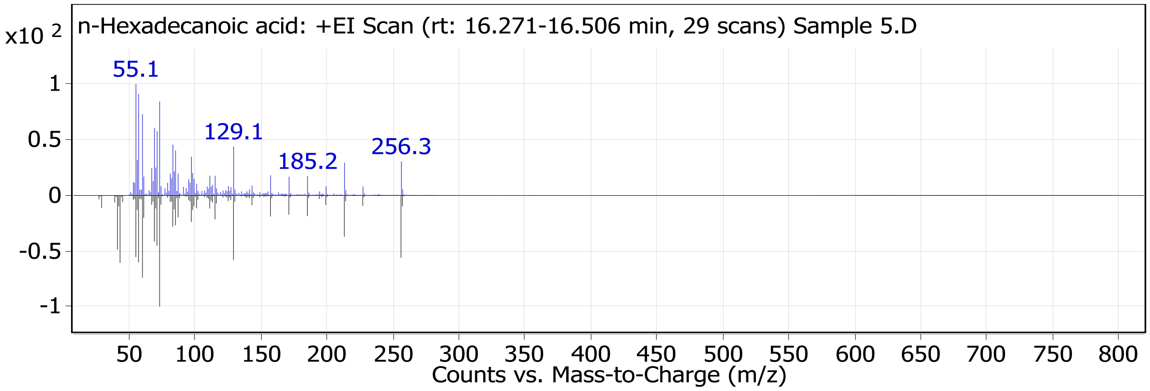

Spectrum Structure

n-Hexadecanoic acid

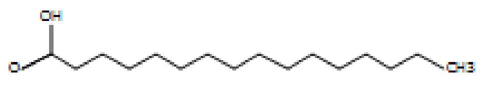

**Spectrum Source**	**Collision Energy**	**Ionization Mode**
Peak (12) in "+ TIC Scan"	0	EI

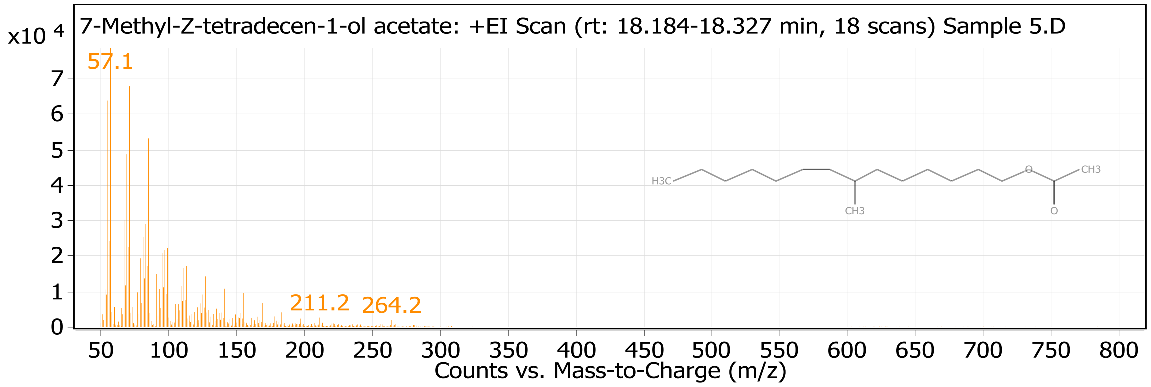

Library Spectrum

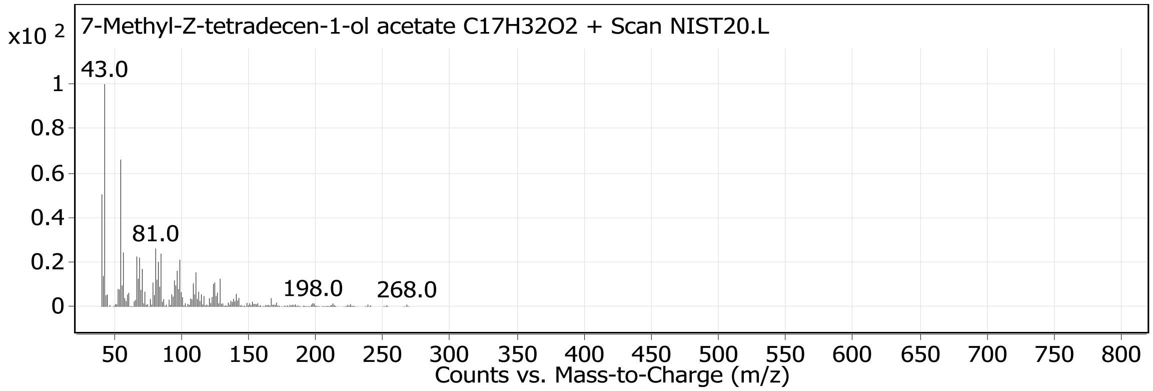

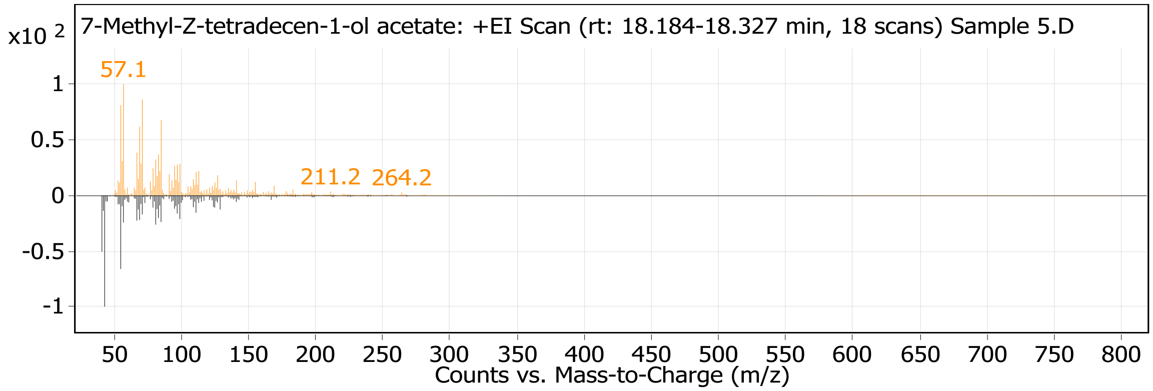

Spectrum Structure

7-Methyl-Z-tetradecen-1-ol acetate

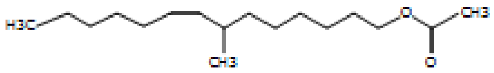

**Spectrum Source**	**Collision Energy**	**Ionization Mode**
Peak (13) in "+ TIC Scan"	0	EI

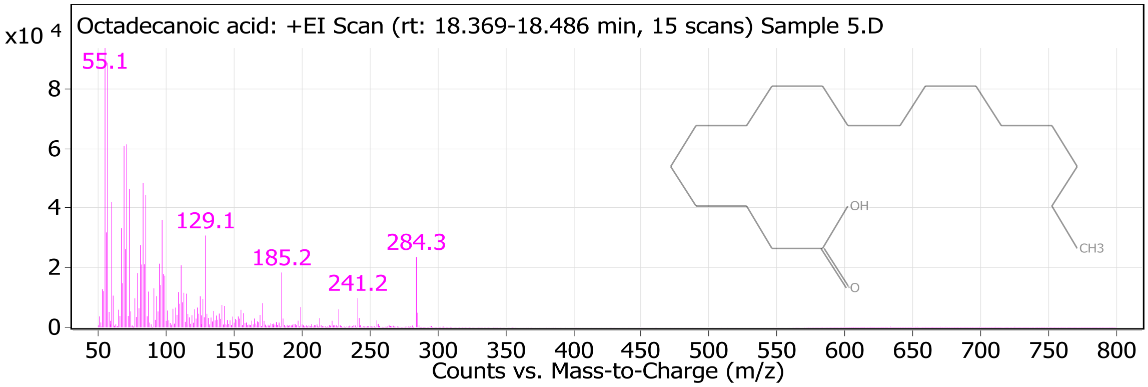

Library Spectrum

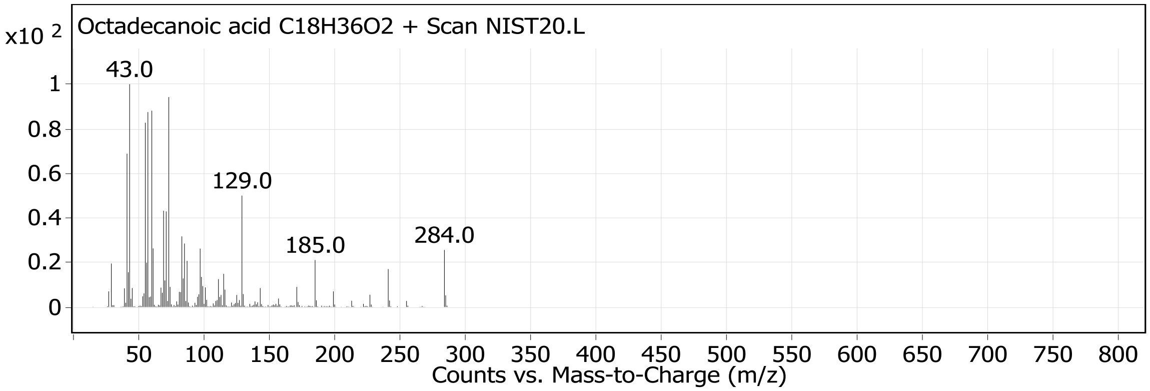

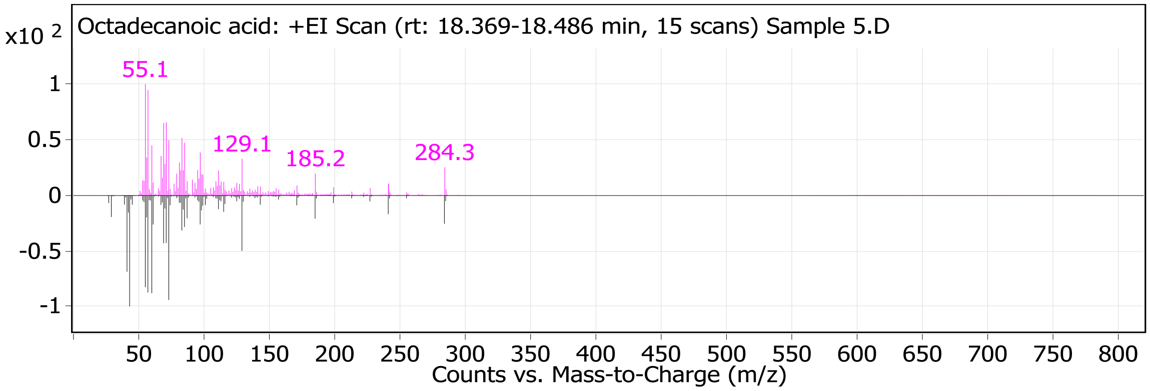

Spectrum Structure

Octadecanoic acid

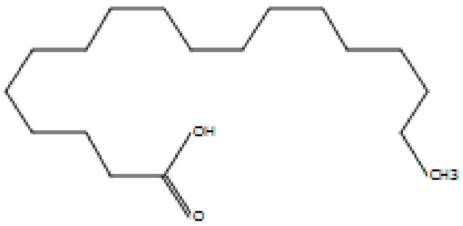

Spectrum Source	Collision Energy	Ionization Mode
Peak (14) in "+ TIC Scan"	0	EI

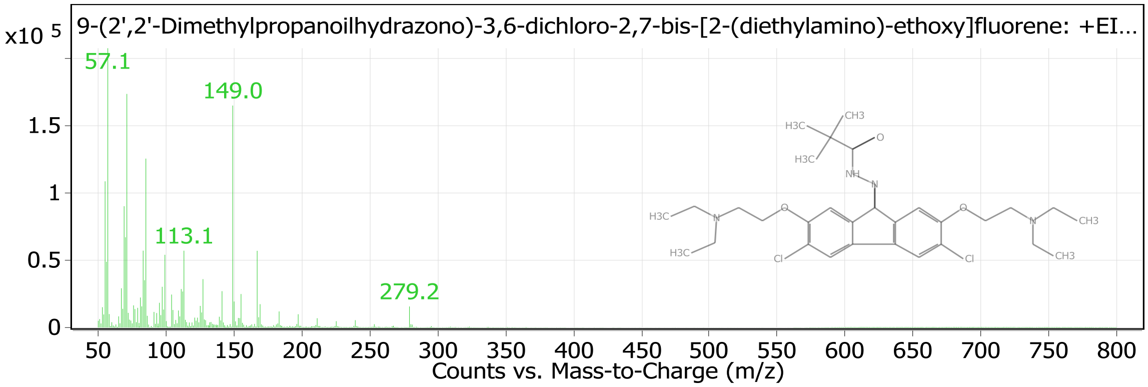

Library Spectrum

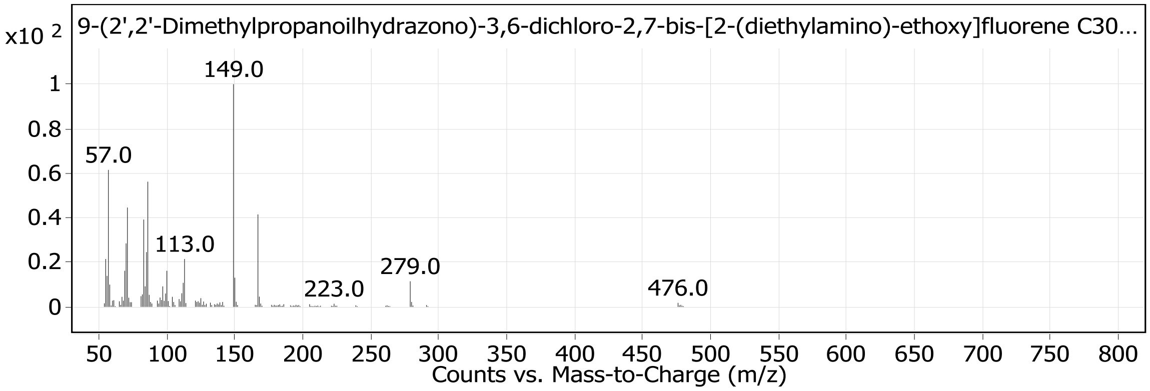

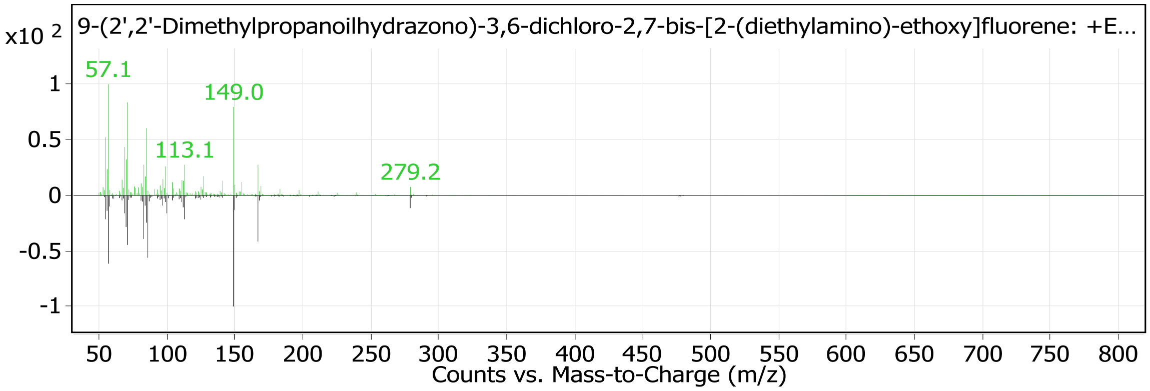

Spectrum Structure

9-(2',2'-Dimethylpropanoilhydrazono)-3,6-dichloro-2,7-bis-[2-(diethylamino)-ethoxy]fluorene

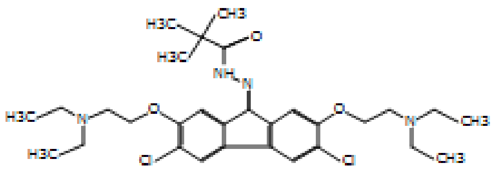

Spectrum Source	Collision Energy	Ionization Mode
Peak (15) in "+ TIC Scan"	0	EI

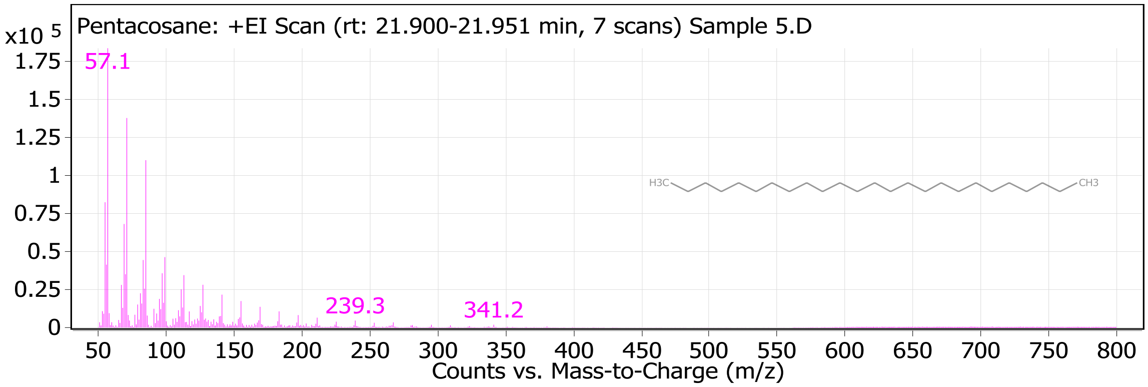

Library Spectrum

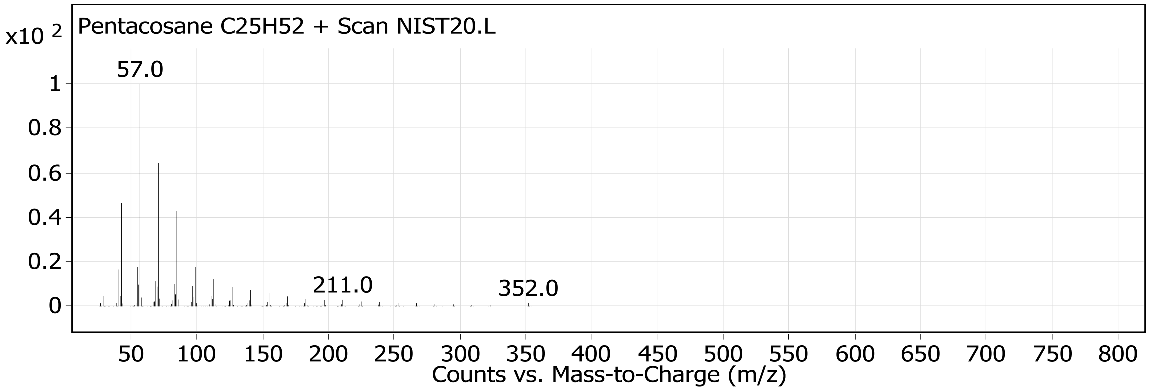

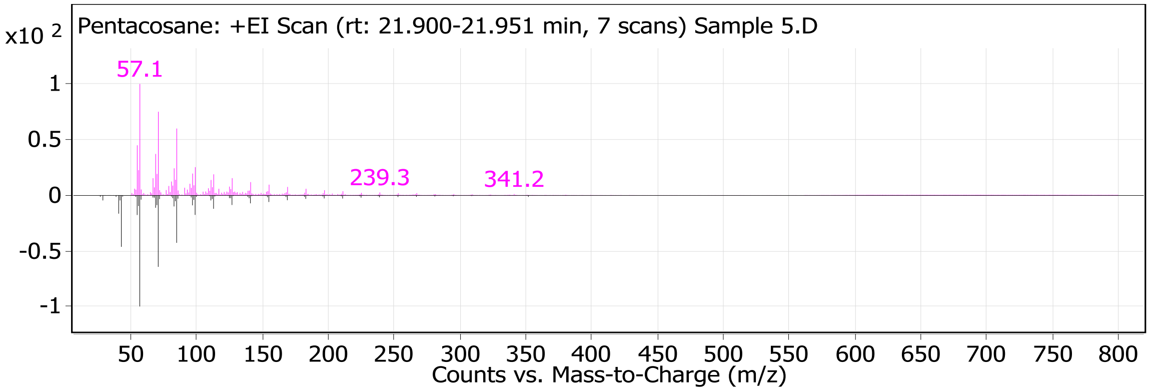

Spectrum Structure

Pentacosane

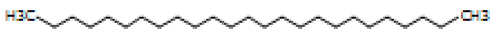

Spectrum Source	Collision Energy	Ionization Mode
Peak (16) in "+ TIC Scan"	0	EI

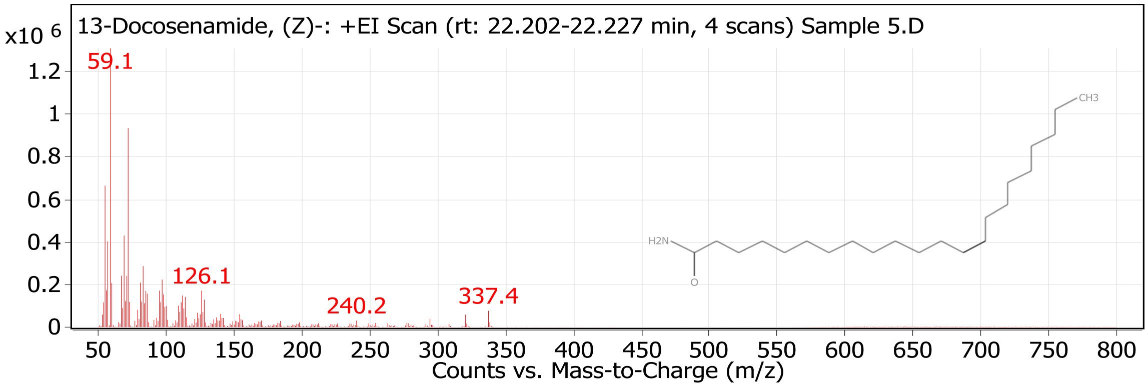

Library Spectrum

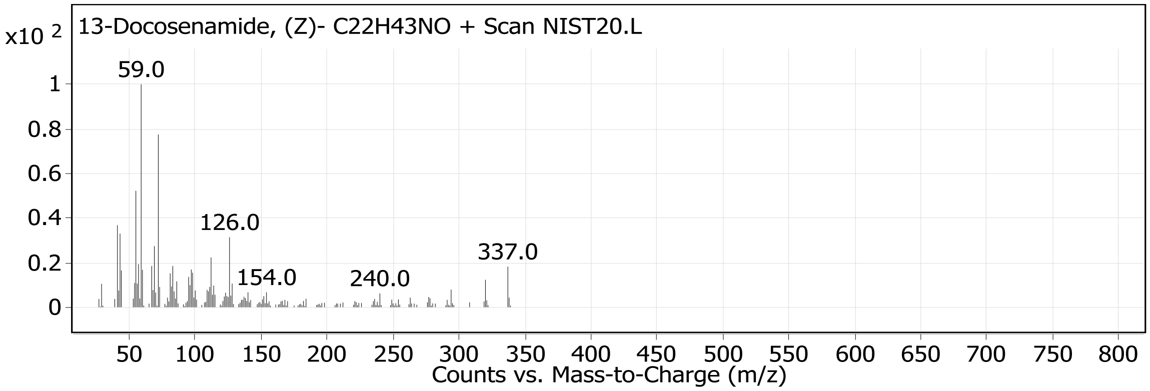

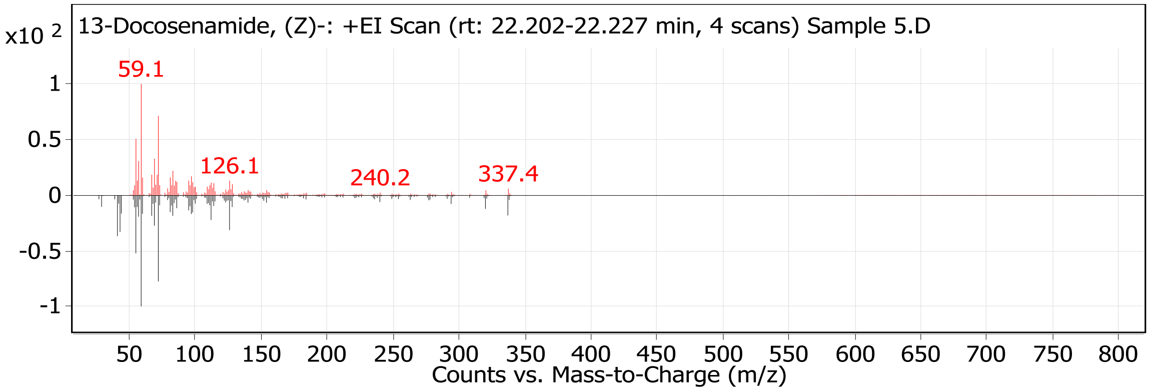

Spectrum Structure

13-Docosenamide, (Z)-

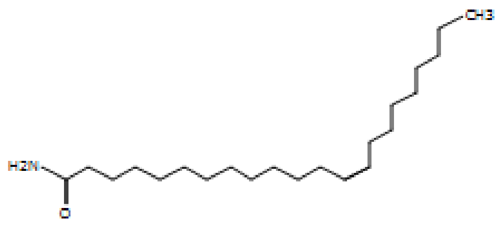

Spectrum Source	Collision Energy	Ionization Mode
Peak (17) in "+ TIC Scan"	0	EI

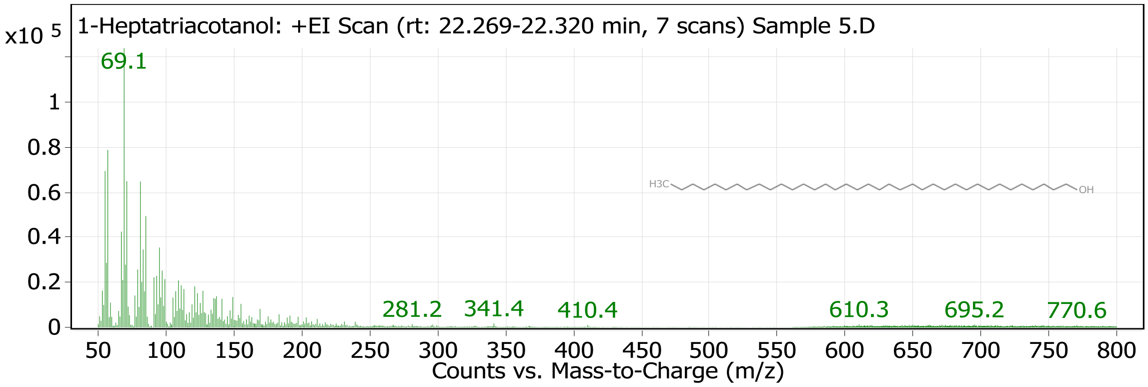

Library Spectrum

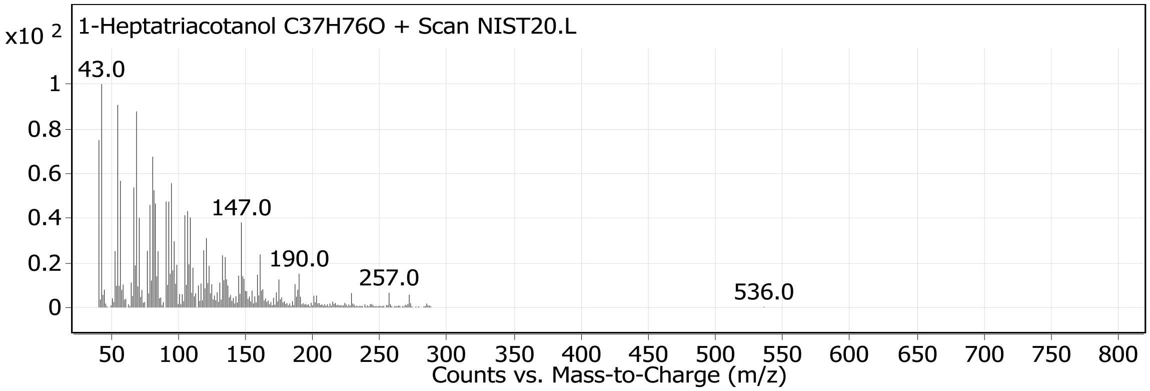

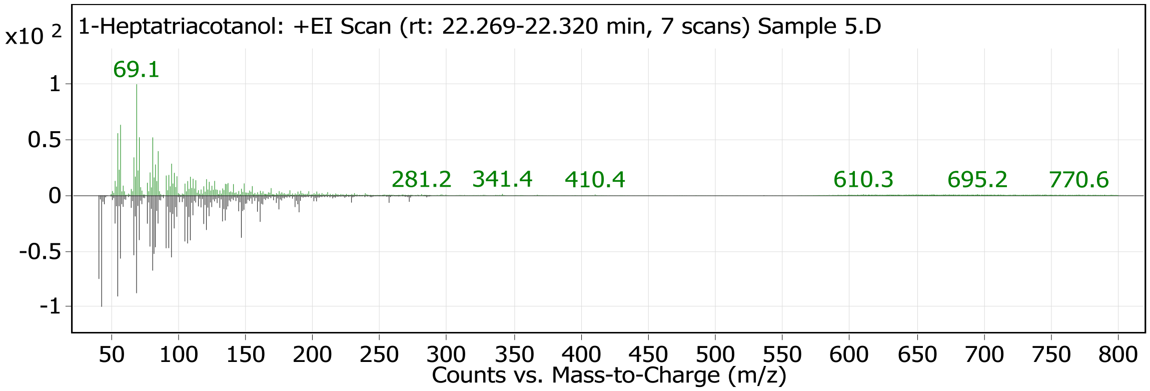

Spectrum Structure

1-Heptatriacotanol

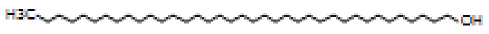

**Spectrum Source**	**Collision Energy**	**Ionization Mode**
Peak (18) in "+ TIC Scan"	0	EI

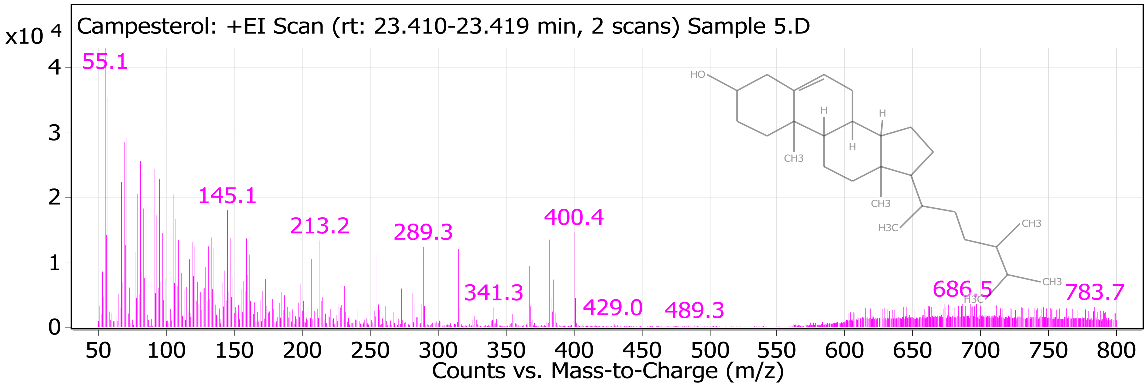

Library Spectrum

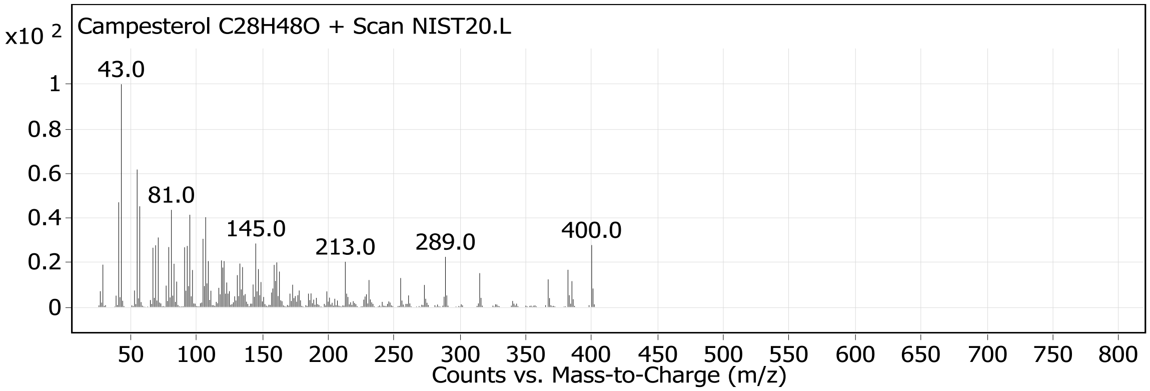

Qualitative Analysis Report

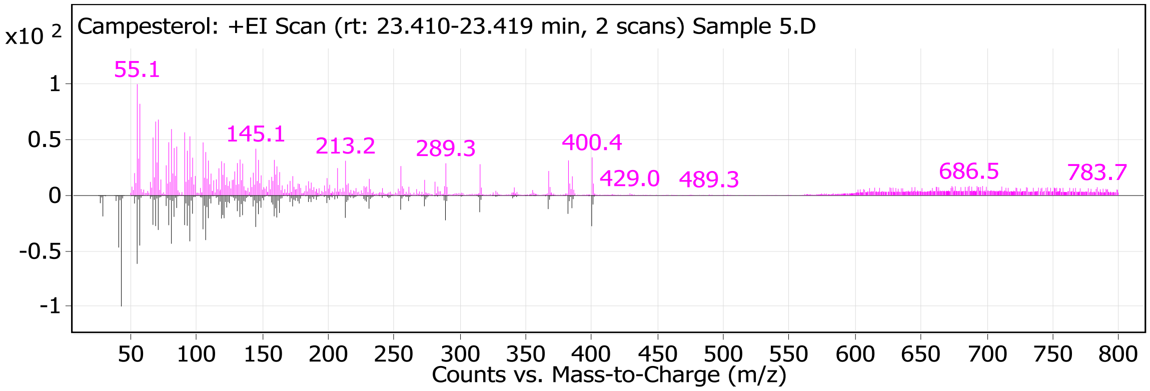

Spectrum Structure

Campesterol

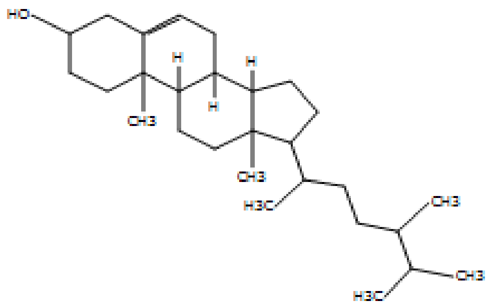

**Spectrum Source**	**Collision Energy**	**Ionization Mode**
Peak (19) in "+ TIC Scan"	0	EI

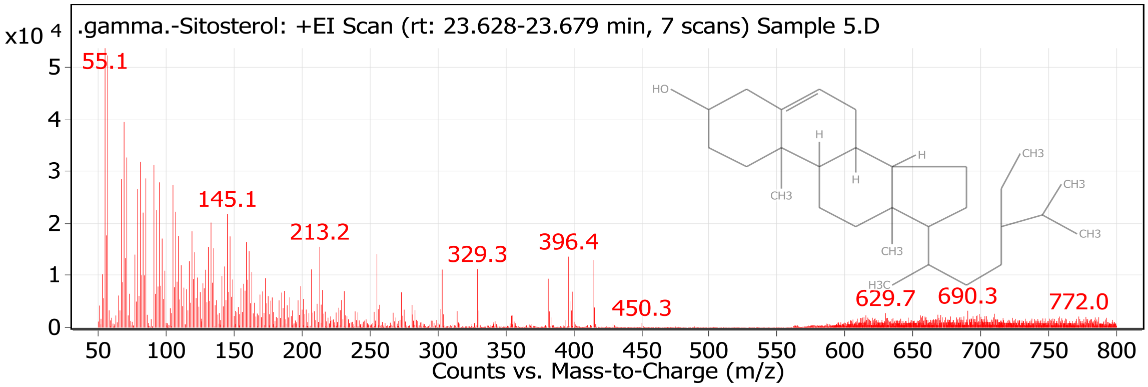

Library Spectrum

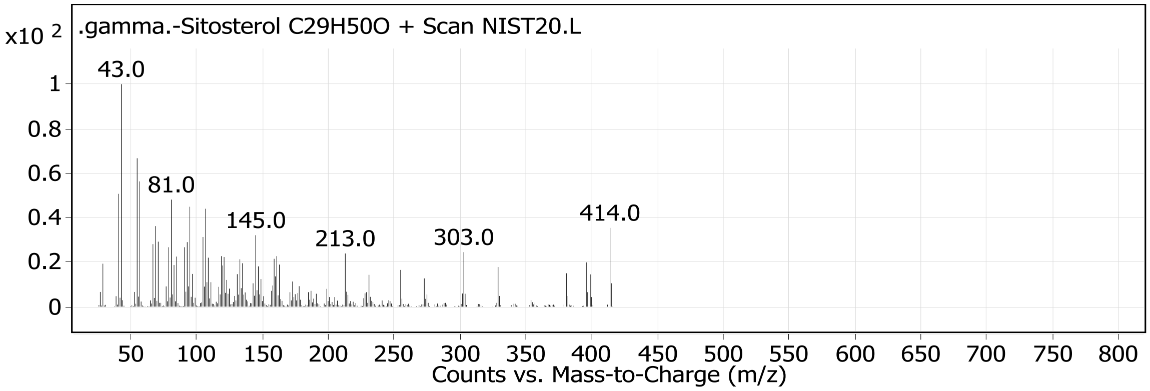

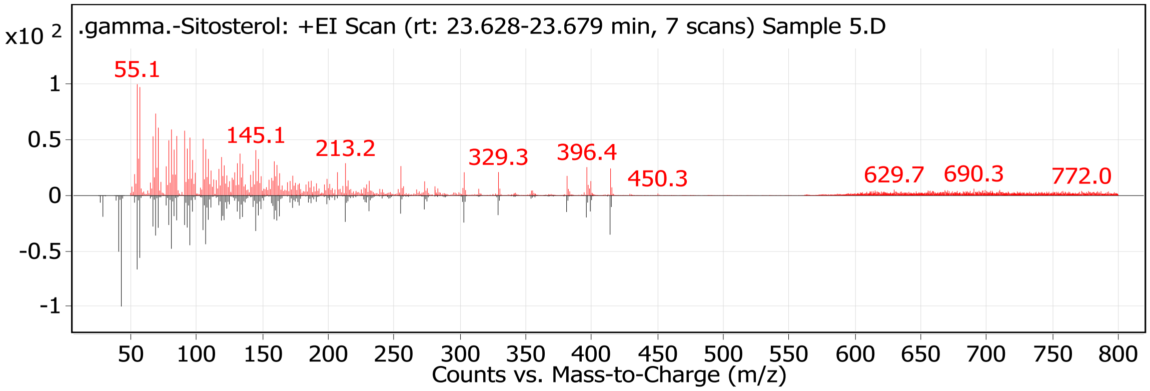

Spectrum Structure

.gamma.-Sitosterol

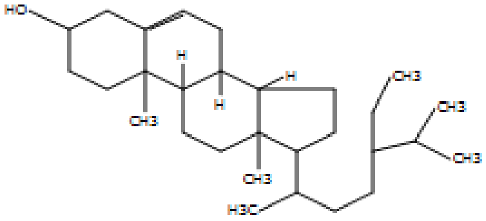

--- End Of Report ---
