# Supplementary material for: Chemical and Molecular Insights into the Arid Wild Plant Diversity of Saudi Arabia
Source: Plants (Basel). 2026 Jan 19;15(2):295. doi: 10.3390/plants15020295 (PMC12845481; doi:10.3390/plants15020295)
Supplement: Supplementary file 1 [file plants-15-00295-s001.zip › Sample 5_AnalysisReport.pdf]

Qualitative Analysis Report

**Data Filename**	Sample 6.D	**Sample Name**	Sample 6
**Sample Type**		**Position**	1
**Instrument Name**	3	**User Name**	
**Acq Method**	Scan DB-5MS Hydrogen 2024.M	**Acquired Time**	6/24/2024 7:42:31 PM
**IRM Calibration Status**	Not Applicable	**DA Method**	SignalToNoiseCheckout.m
**Comment**			

**Expected Barcode**		**Sample Amount**	
**Dual Inj Vol**	0.2	**TuneName**	ATUNE.U
**TunePath**	D:\MassHunter\GCMS\3\5977 \	**TuneDateStamp**	2024-06-23T14:01:57+02:00
**MSFirmwareVersion**	6.00.34	**OperatorName**	
**RunCompletedFlag**	True	**Acquisition SW Version**	MassHunter GC/MS Acquisition 10.0.368 14-Feb-2019 Copyright © 1989-2018 Agilent Technologies, Inc

User Chromatograms

Fragmentor Voltage **Collision Energy** 0 **Ionization Mode** EI

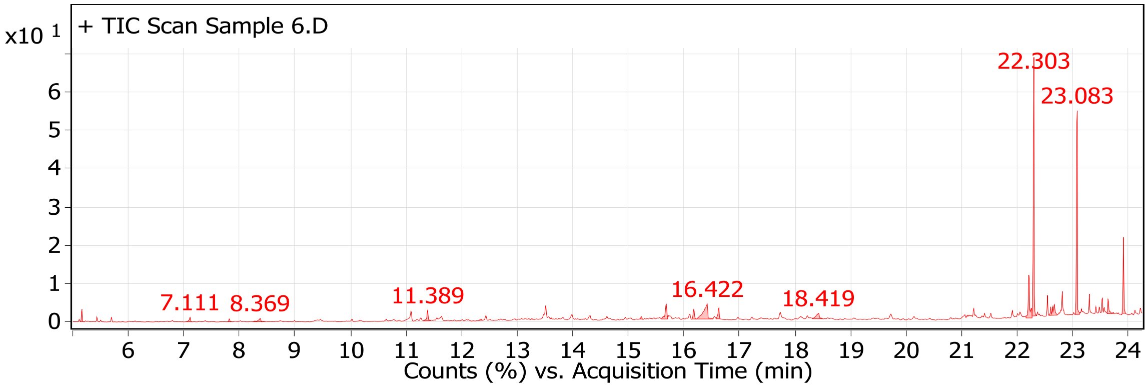

Integration Peak List

Peak	Start	RT	End	Height	Area	Area %
1	5.131	5.165	5.188	2156462.69	2256121.11	4.81
2	7.036	7.111	7.139	757801.28	1417388.23	3.02
3	7.782	7.816	7.865	486180.54	665336.67	1.42
4	8.241	8.369	8.462	581060.03	2064903.72	4.4
5	11.314	11.389	11.44	1794037.9	3181006.08	6.78
6	12.317	12.354	12.379	202894.49	454369.56	0.97
7	15.206	15.24	15.255	393096.28	552799.54	1.18
8	15.592	15.684	15.709	2549135.71	5886741.88	12.54
9	16.144	16.179	16.214	1646134.24	2721615.07	5.8
10	16.229	16.422	16.523	2608604.12	14356190.23	30.58
11	16.582	16.632	16.656	1882535.75	3201561.3	6.82
12	18.318	18.419	18.499	933024.28	4109534.9	8.75
13	22.16	22.211	22.269	7538411.8	14465683.58	30.81
14	22.269	22.303	22.353	45328338.22	46944503.89	100
15	22.571	22.672	22.722	1847239.44	6647480.41	14.16
16	23.041	23.083	23.125	35717337.2	43776648.84	93.25
17	23.62	23.637	23.746	2466516.85	4014934.35	8.55
18	23.889	23.914	23.953	13312913.71	13373398.58	28.49

User Spectra

Qualitative Analysis Report

Spectrum Source	Collision Energy	Ionization Mode
Peak (1) in "+ TIC Scan"	0	EI

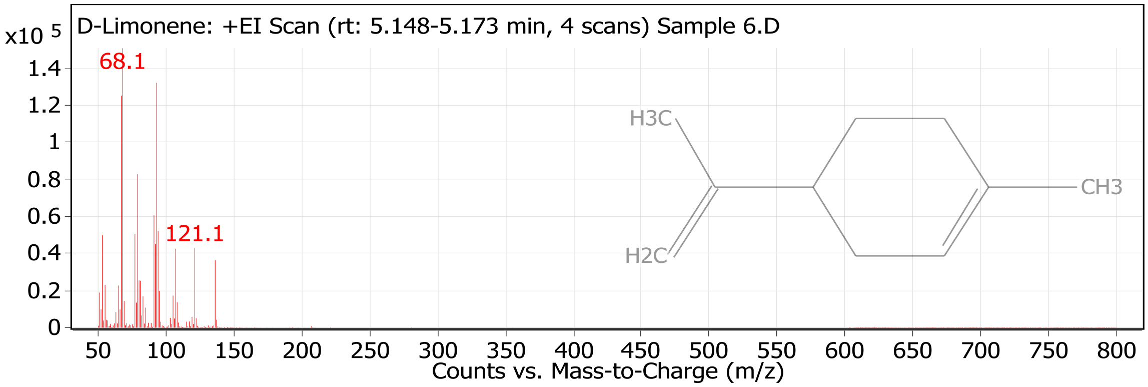

Library Spectrum

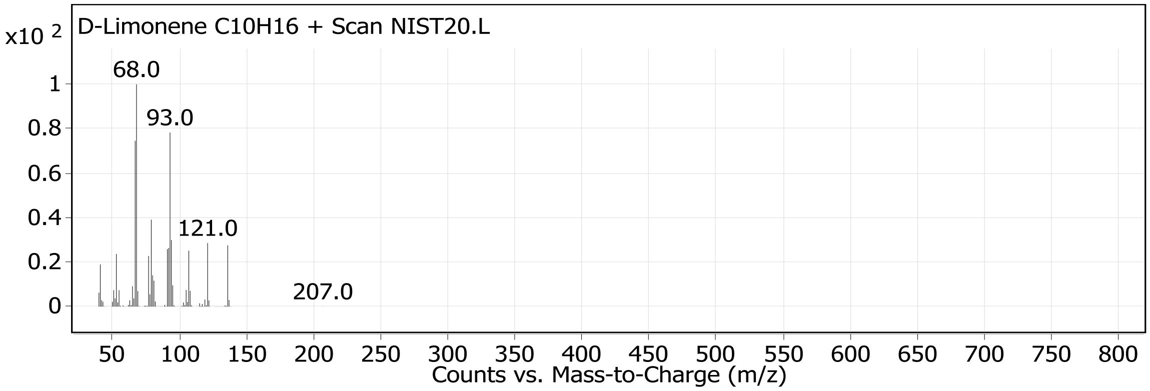

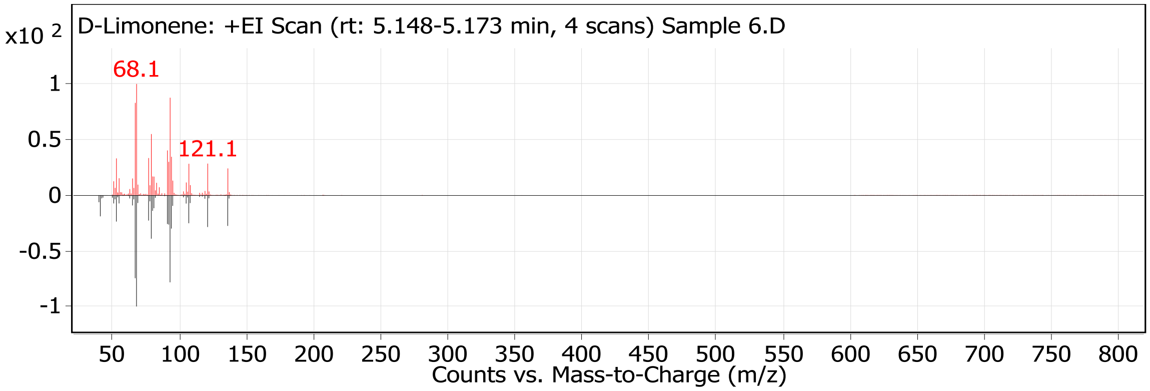

Spectrum Structure

D-Limonene

Qualitative Analysis Report

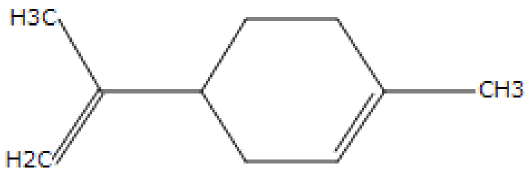

Spectrum Source	Collision Energy	Ionization Mode
Peak (2) in "+ TIC Scan"	0	EI

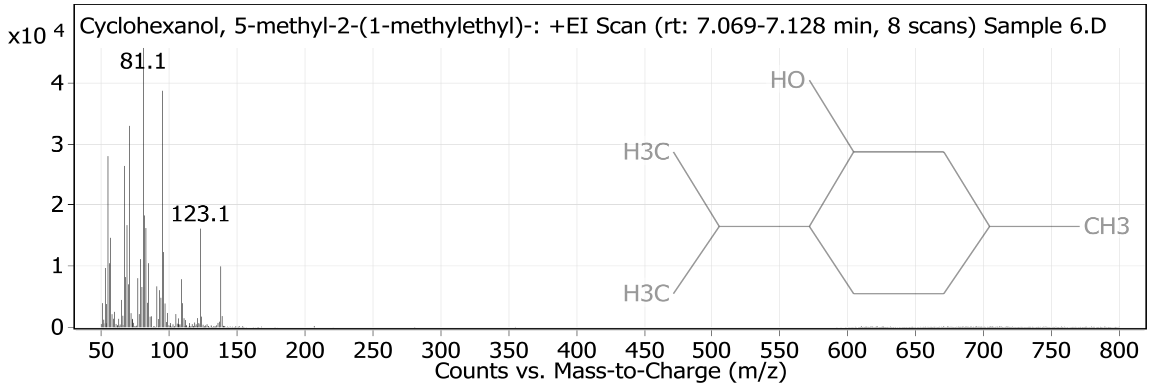

Library Spectrum

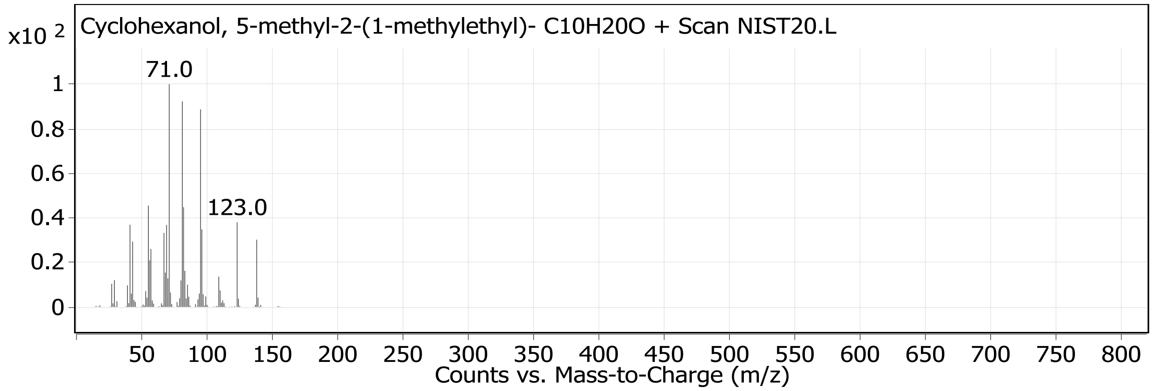

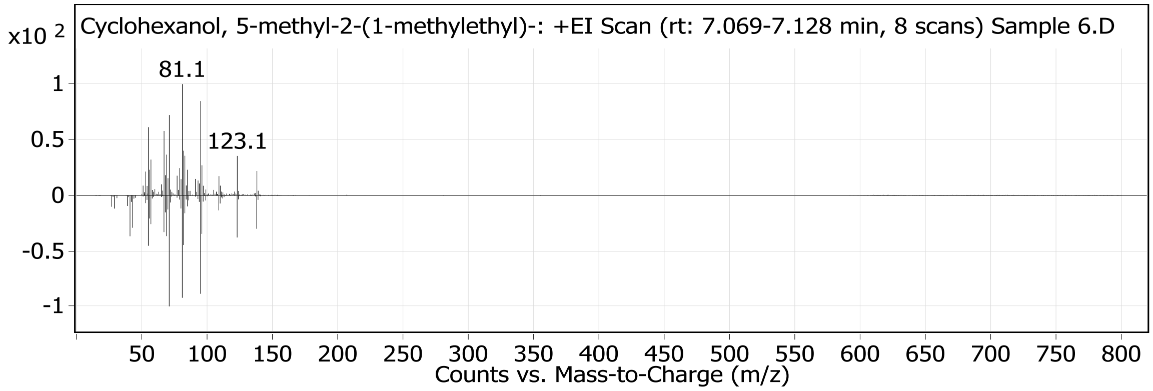

Spectrum Structure

Cyclohexanol, 5-methyl-2-(1-methylethyl)-

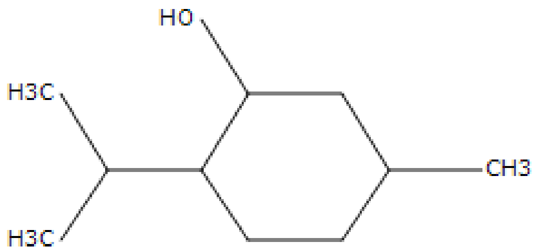

Spectrum Source	Collision Energy	Ionization Mode
Peak (3) in "+ TIC Scan"	0	EI

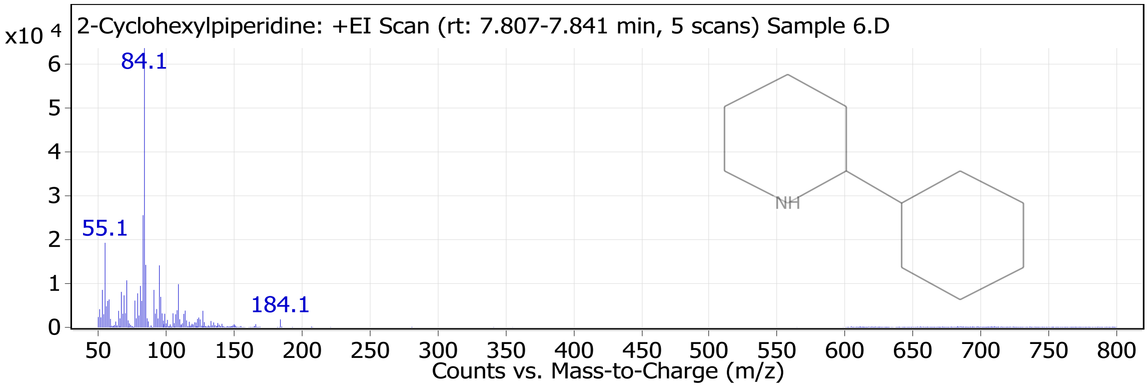

Library Spectrum

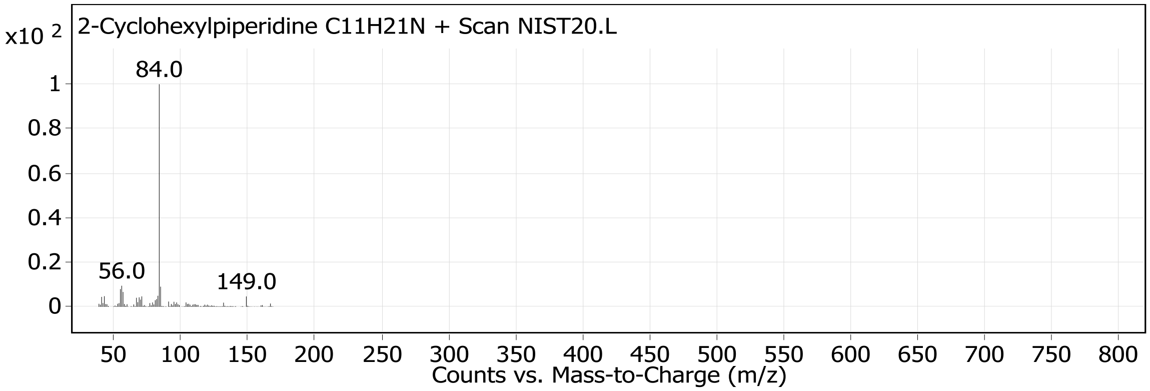

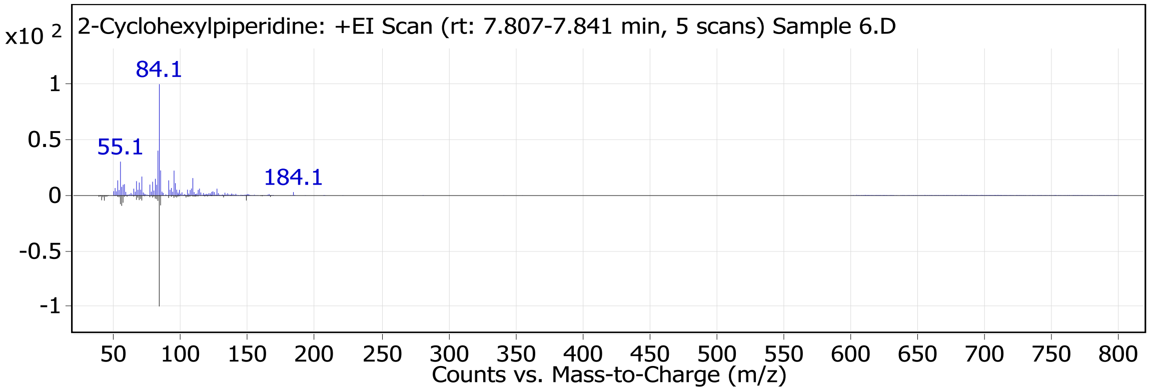

Spectrum Structure

2-Cyclohexylpiperidine

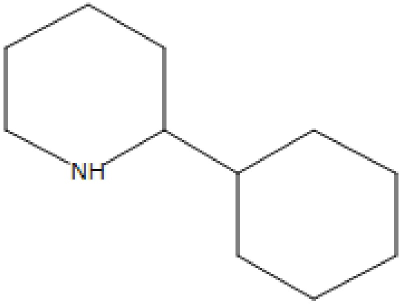

Spectrum Source	Collision Energy	Ionization Mode
Peak (4) in "+ TIC Scan"	0	EI

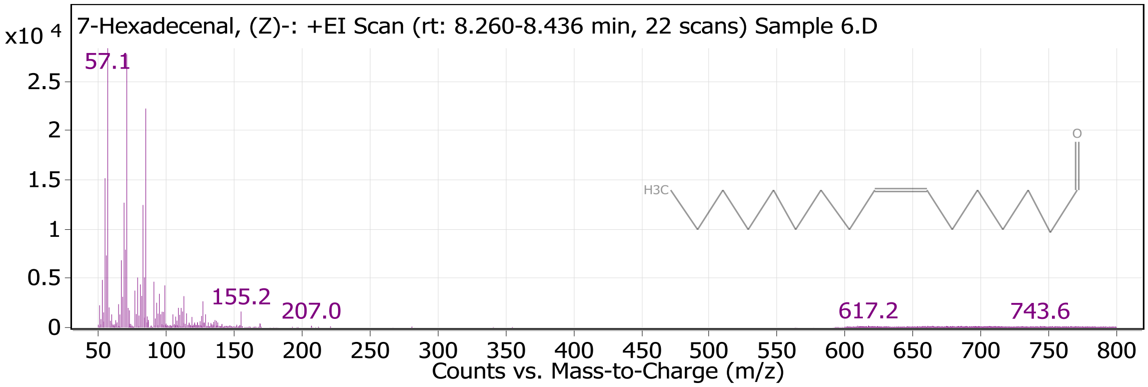

Library Spectrum

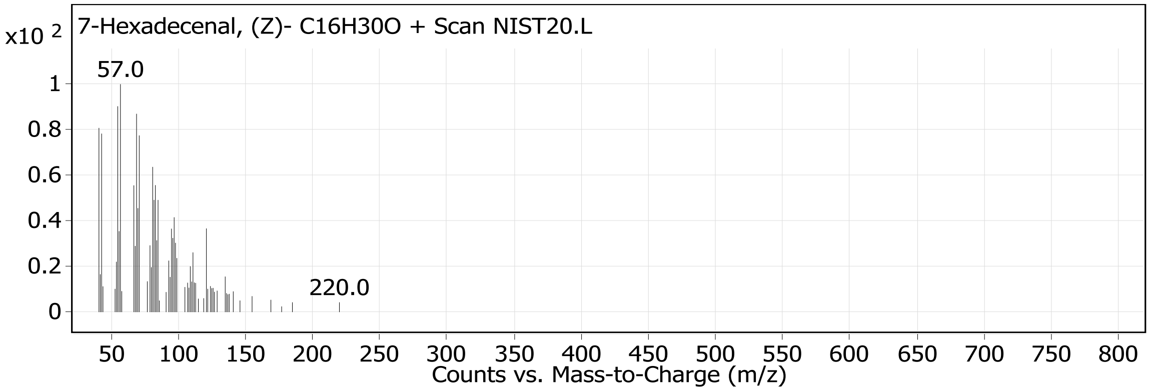

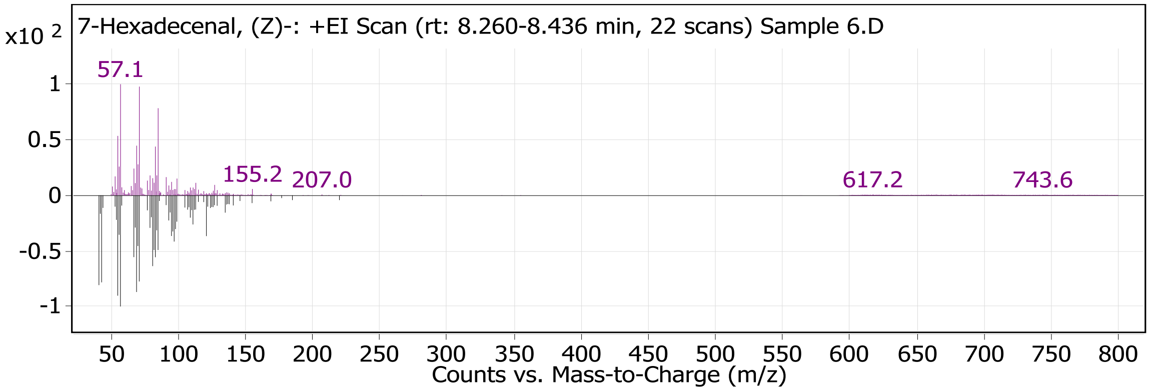

Spectrum Structure

7-Hexadecenal, (Z)-

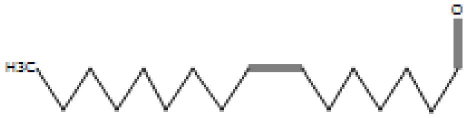

**Spectrum Source**	**Collision Energy**	**Ionization Mode**
Peak (5) in "+ TIC Scan"	0	EI

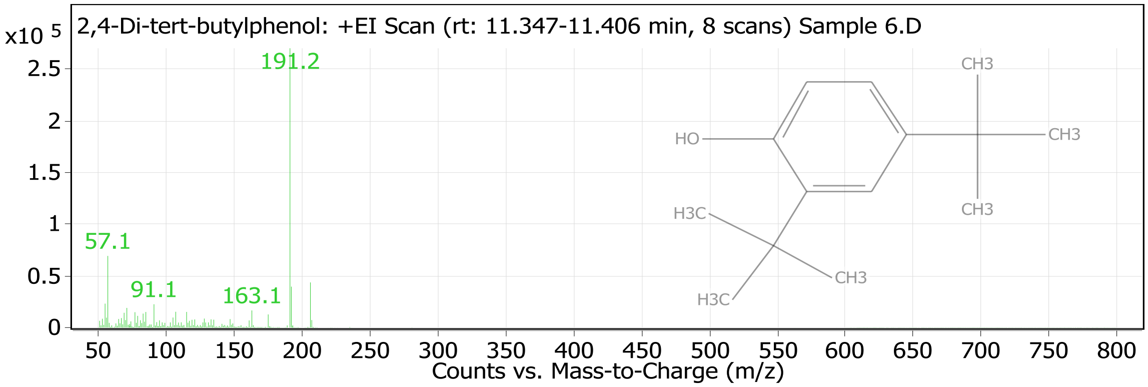

Library Spectrum

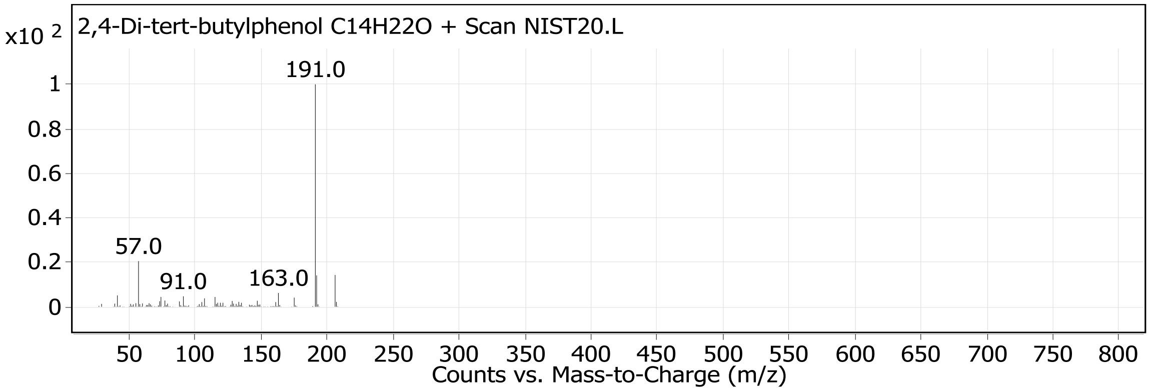

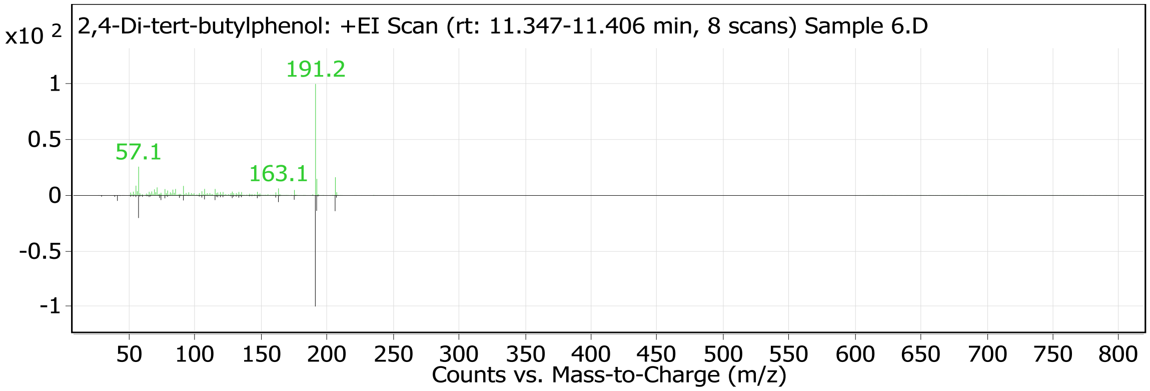

Spectrum Structure

2,4-Di-tert-butylphenol

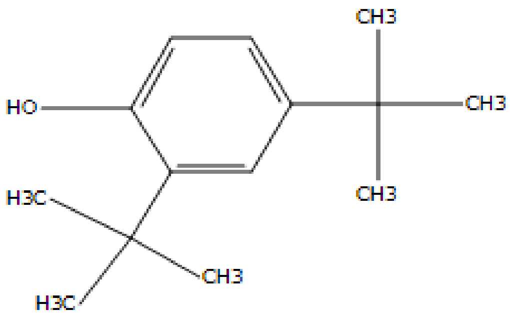

Qualitative Analysis Report

Spectrum Source	Collision Energy	Ionization Mode
Peak (6) in "+ TIC Scan"	0	EI

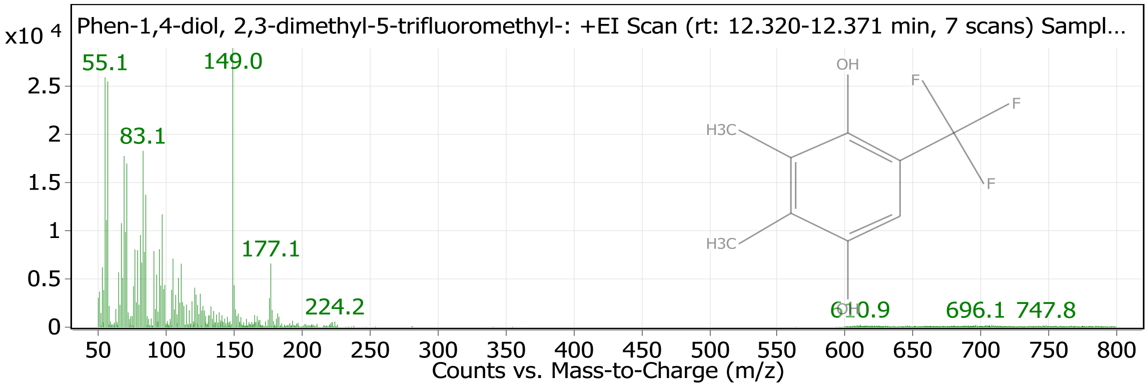

Library Spectrum

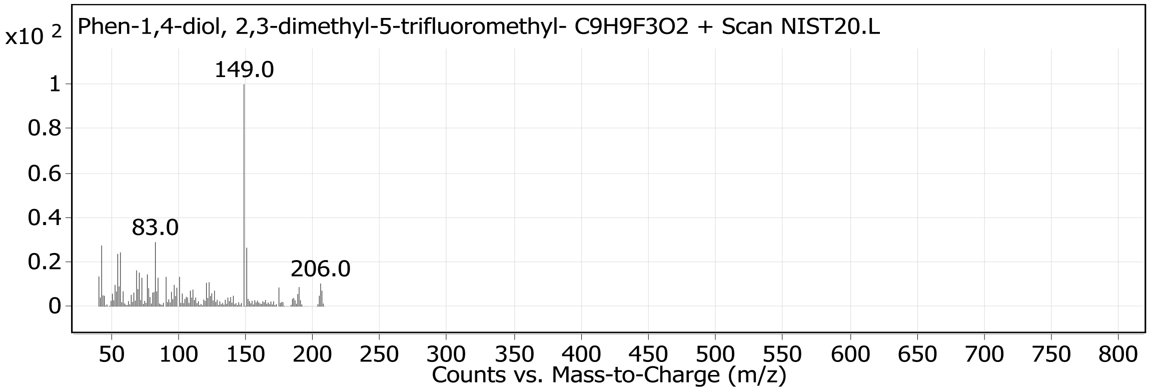

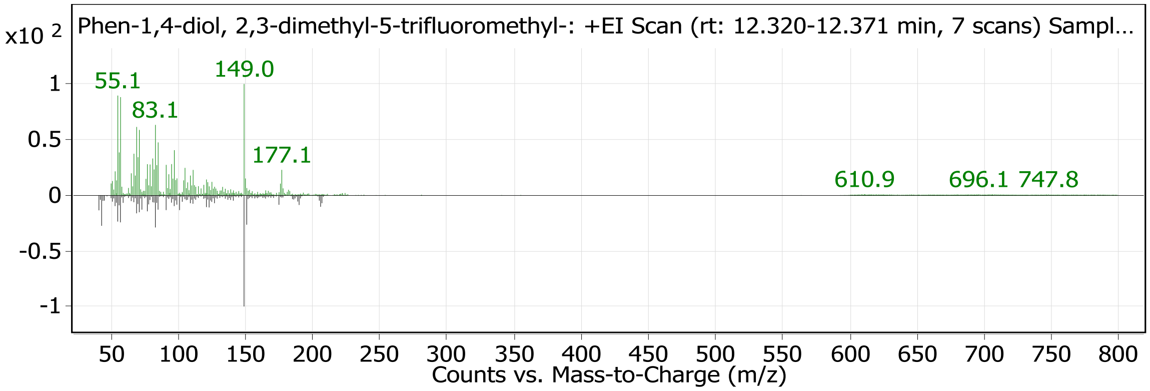

Spectrum Structure

Phen-1,4-diol, 2,3-dimethyl-5-trifluoromethyl-

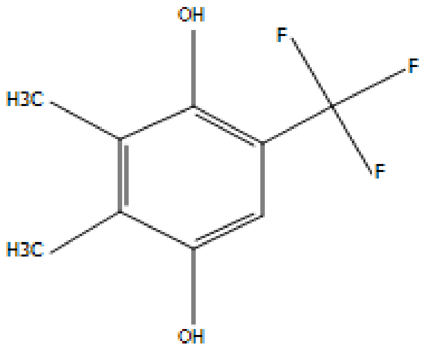

Spectrum Source	Collision Energy	Ionization Mode
Peak (7) in "+ TIC Scan"	0	EI

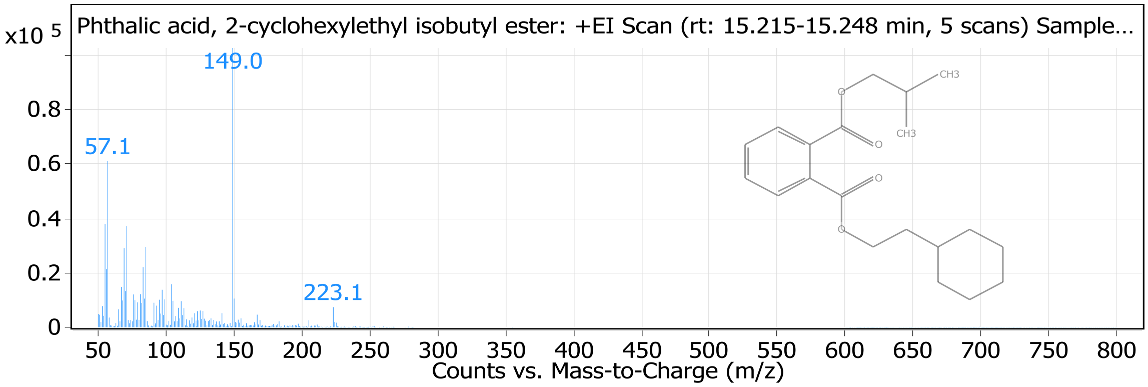

Library Spectrum

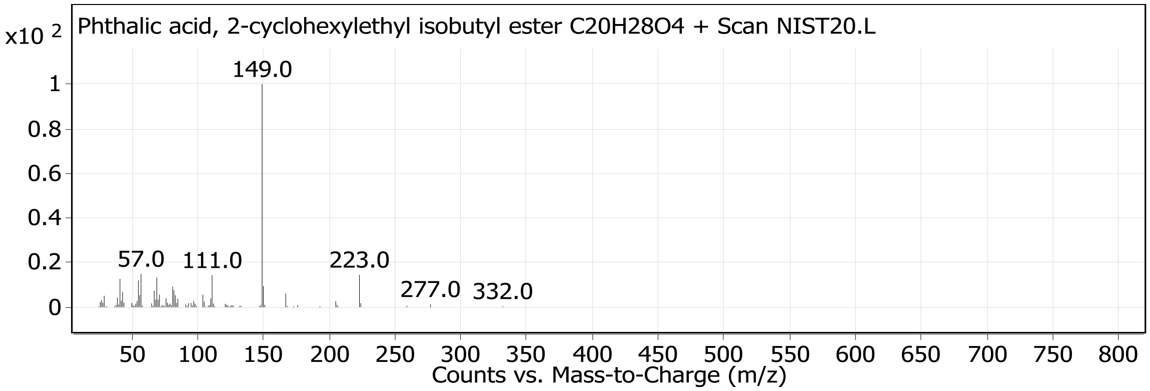

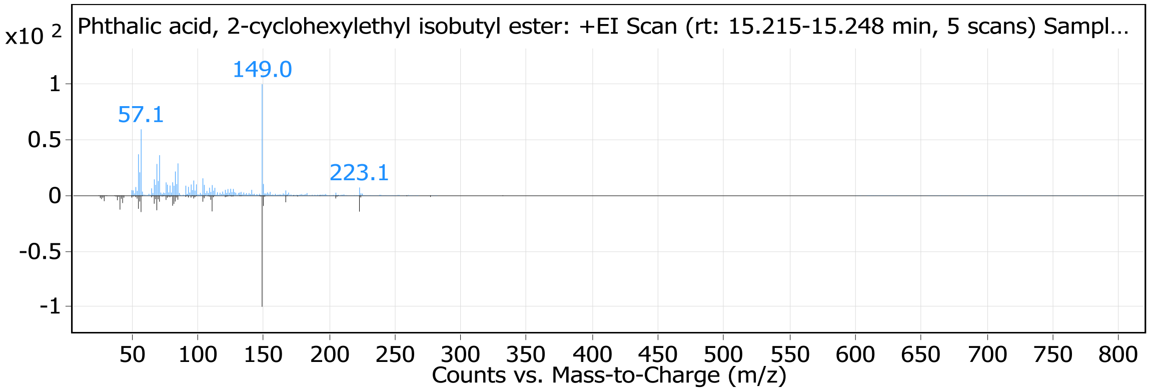

Spectrum Structure

Phthalic acid, 2-cyclohexylethyl isobutyl ester

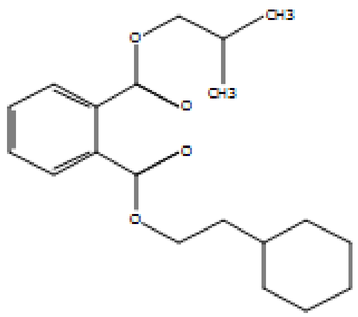

**Spectrum Source**	**Collision Energy**	**Ionization Mode**
Peak (8) in "+ TIC Scan"	0	EI

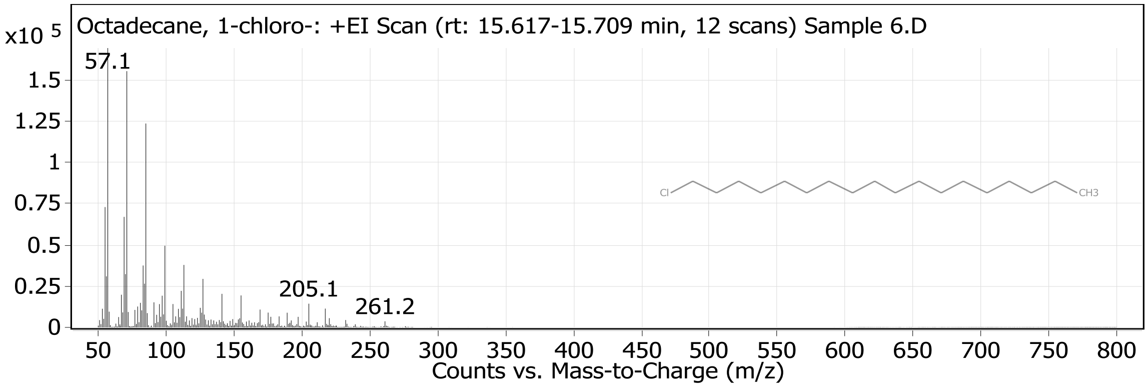

Library Spectrum

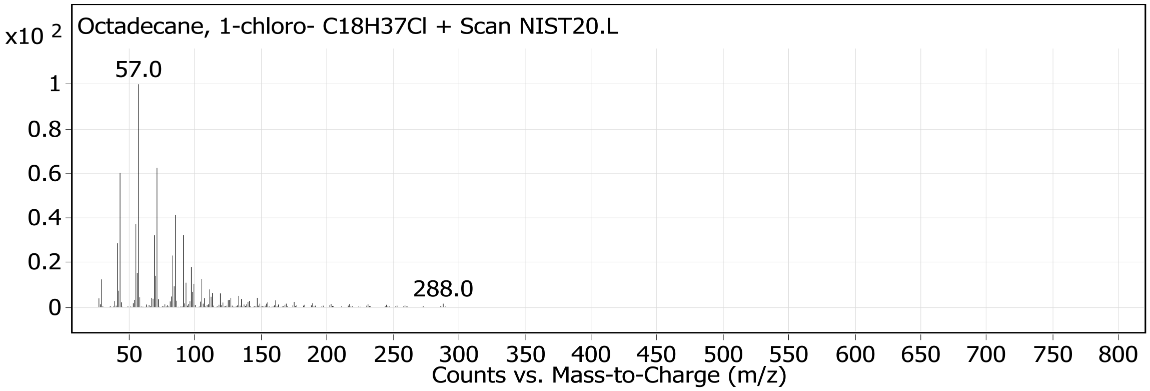

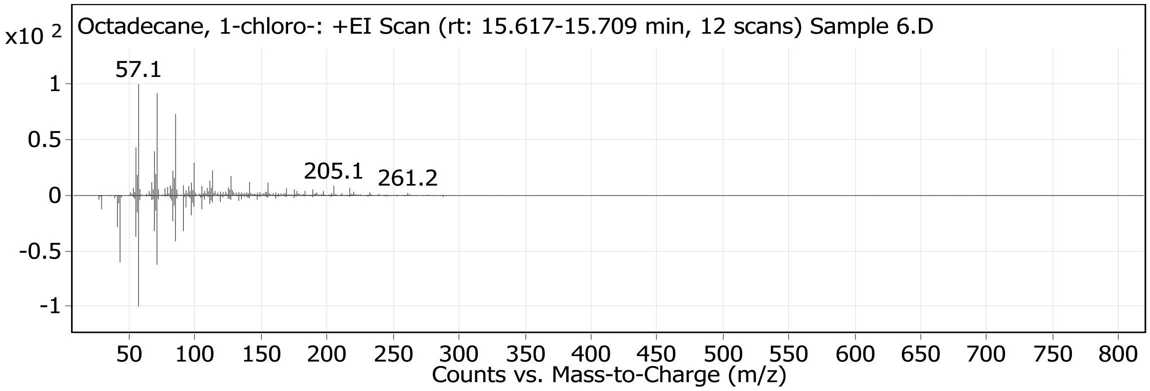

Spectrum Structure

Octadecane, 1-chloro-

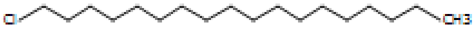

Spectrum Source	Collision Energy	Ionization Mode
Peak (9) in "+ TIC Scan"	0	EI

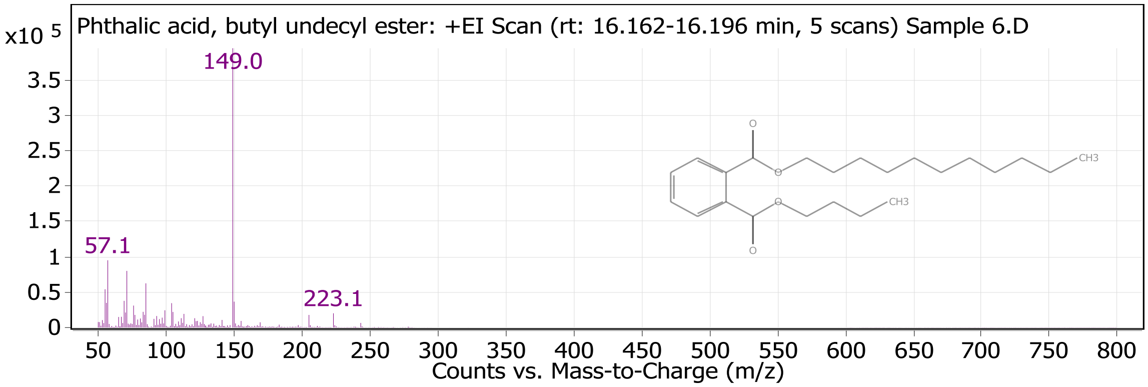

Library Spectrum

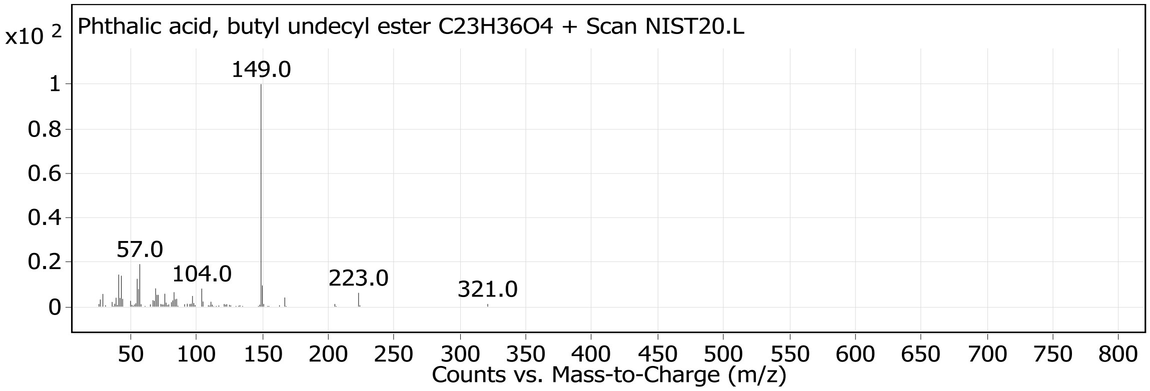

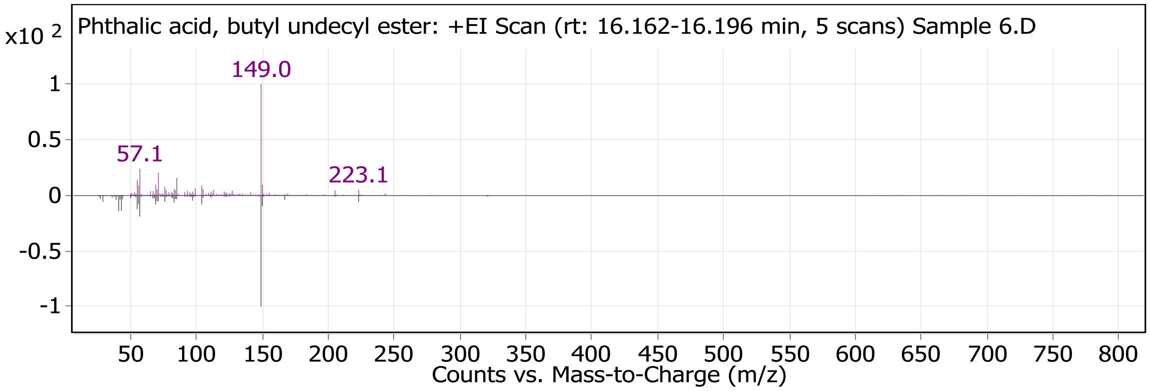

Spectrum Structure

Phthalic acid, butyl undecyl ester

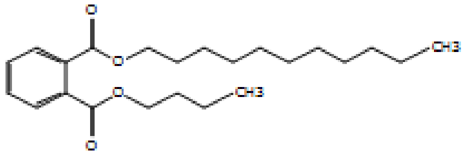

Spectrum Source	Collision Energy	Ionization Mode
Peak (10) in "+ TIC Scan"	0	EI

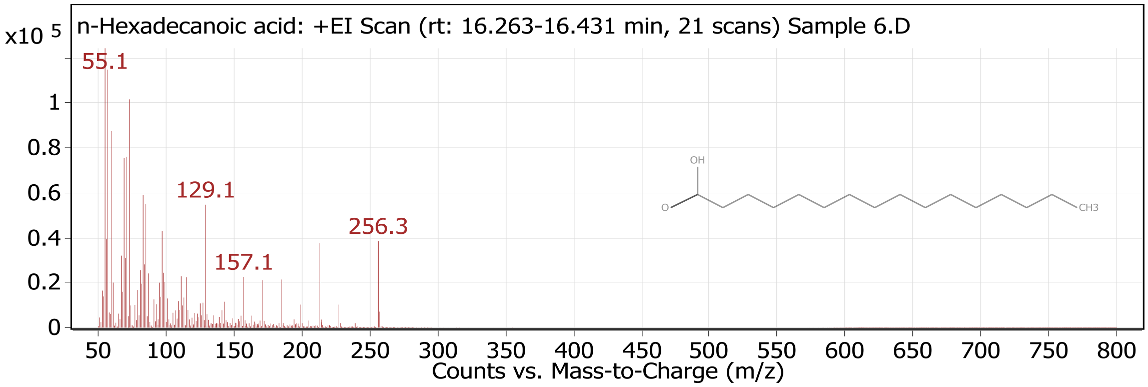

Library Spectrum

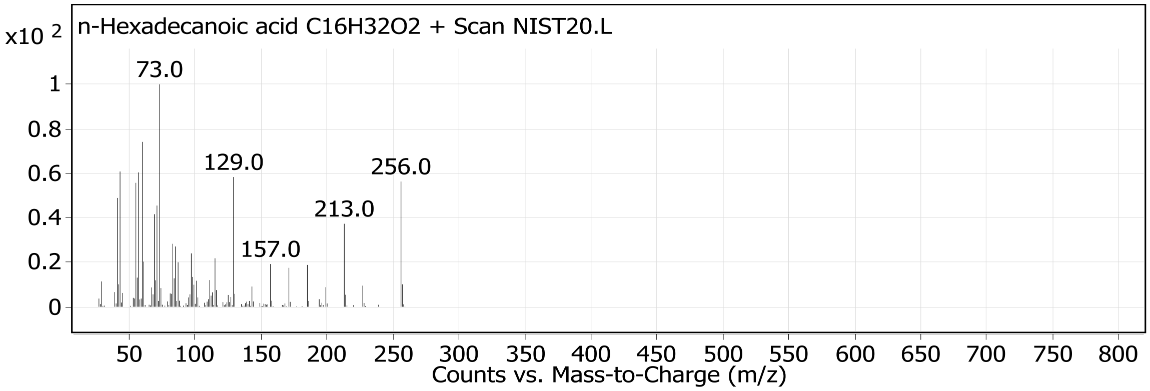

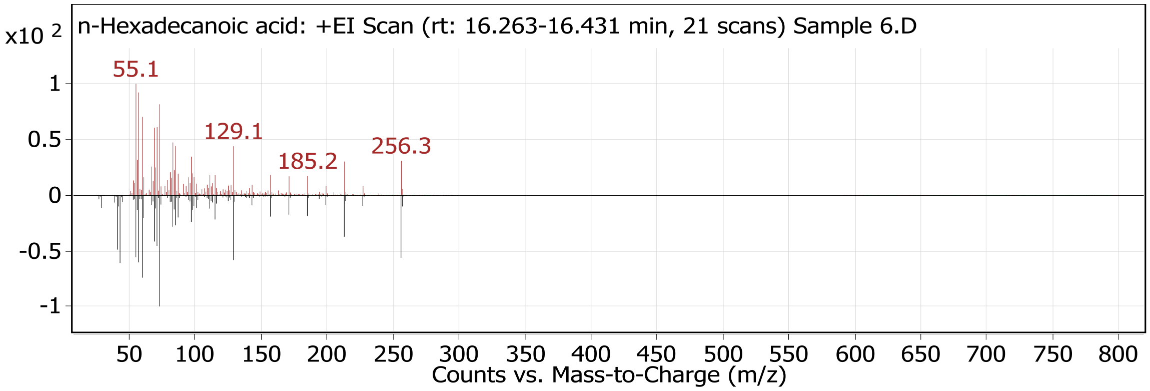

Spectrum Structure

n-Hexadecanoic acid

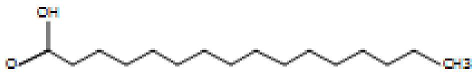

Qualitative Analysis Report

Spectrum Source	Collision Energy	Ionization Mode
Peak (11) in "+ TIC Scan"	0	EI

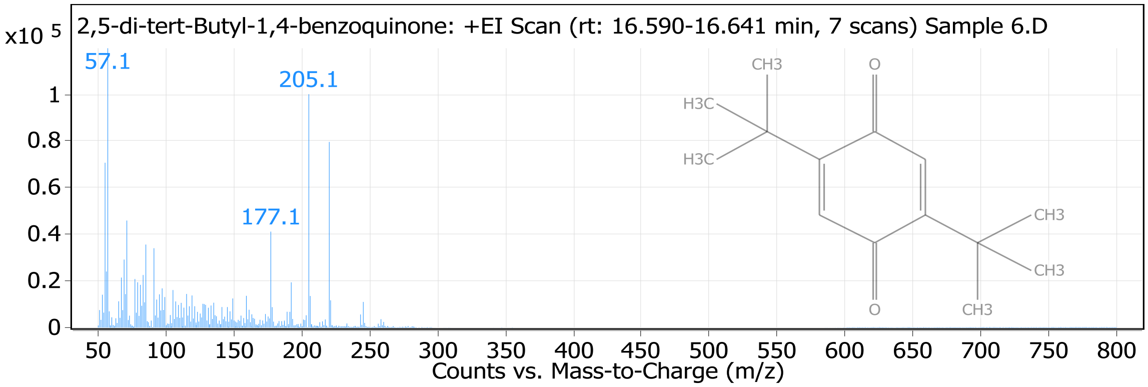

Library Spectrum

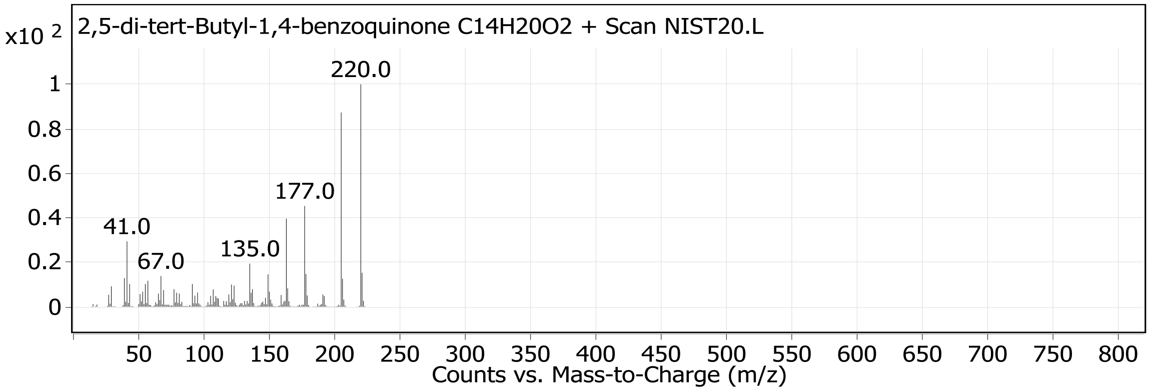

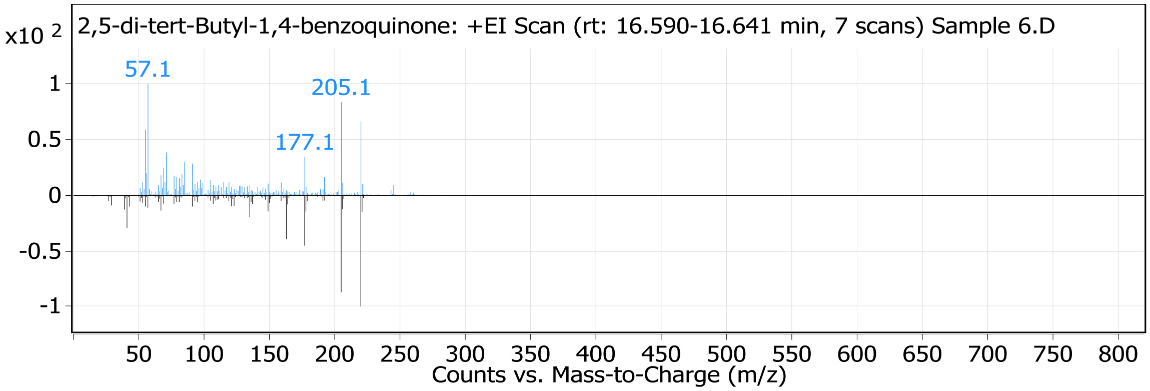

Spectrum Structure

2,5-di-tert-Butyl-1,4-benzoquinone

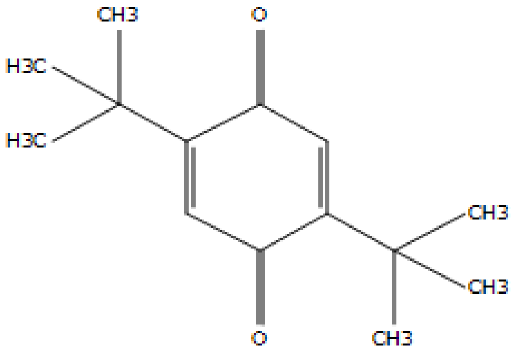

**Spectrum Source**	**Collision Energy**	**Ionization Mode**
Peak (12) in "+ TIC Scan"	0	EI

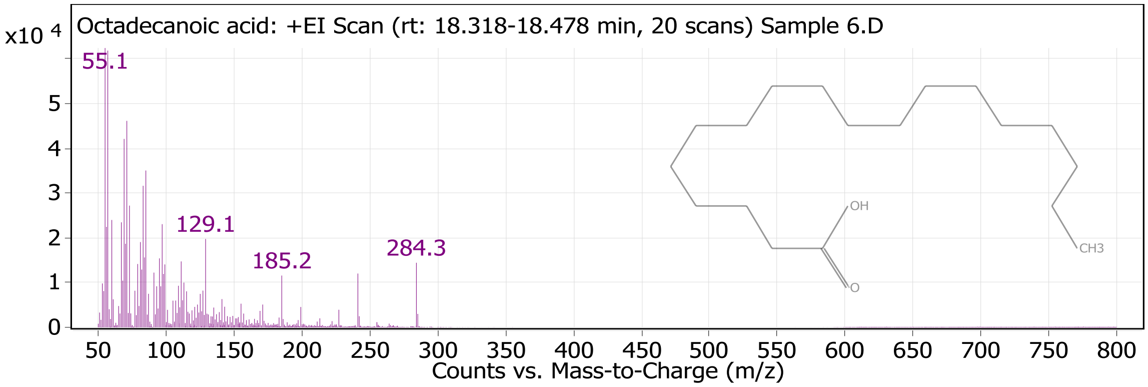

Library Spectrum

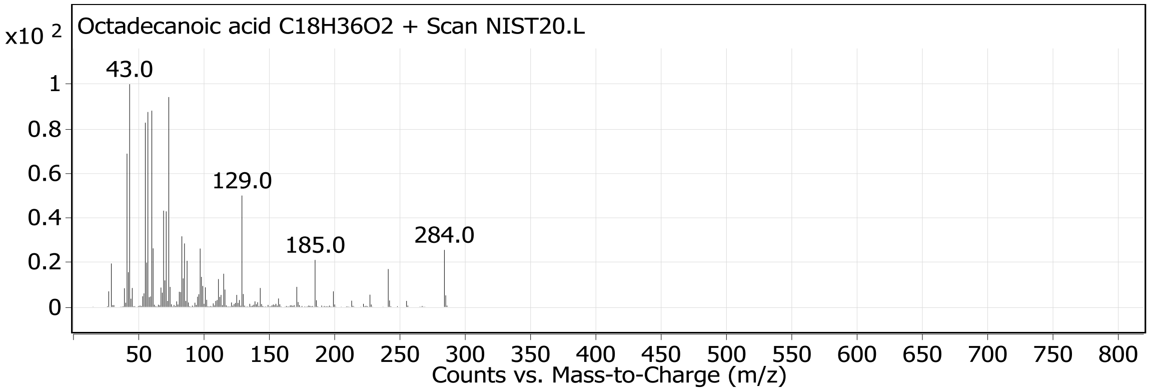

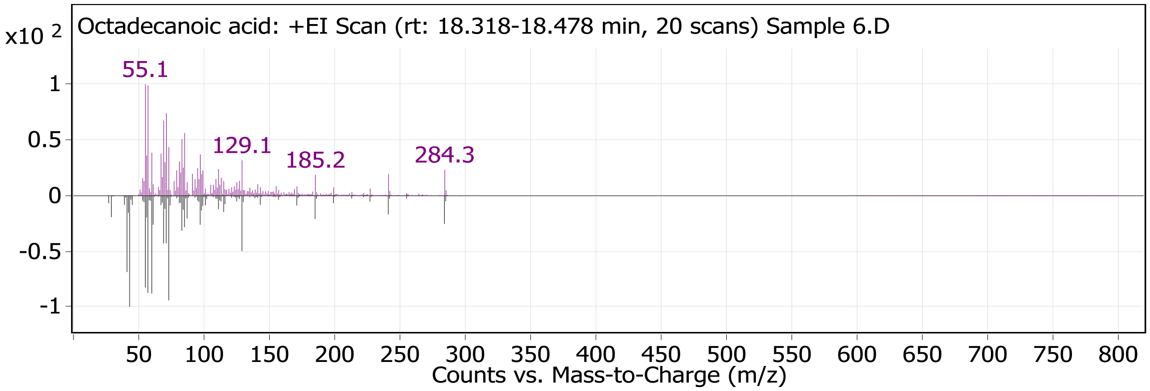

Spectrum Structure

Octadecanoic acid

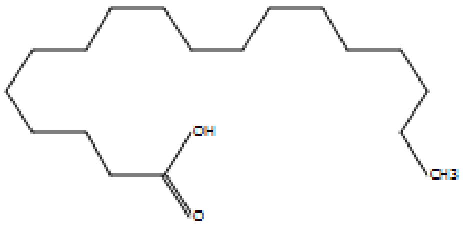

Spectrum Source	Collision Energy	Ionization Mode
Peak (13) in "+ TIC Scan"	0	EI

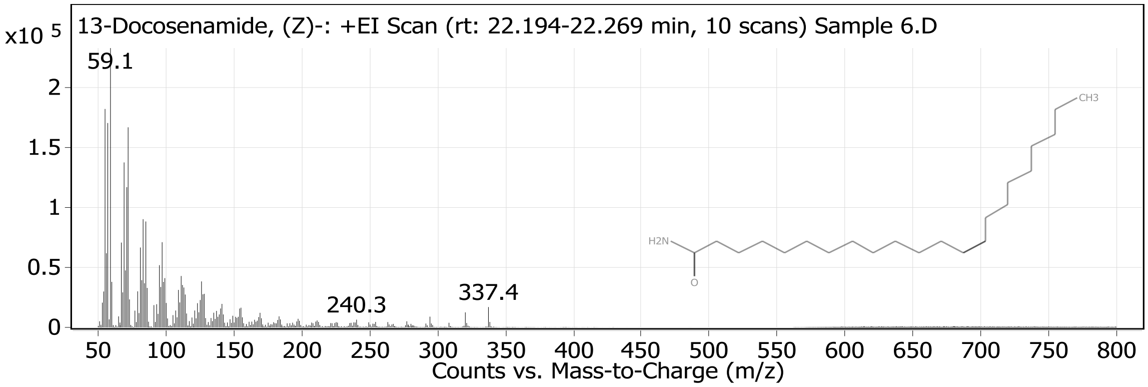

Library Spectrum

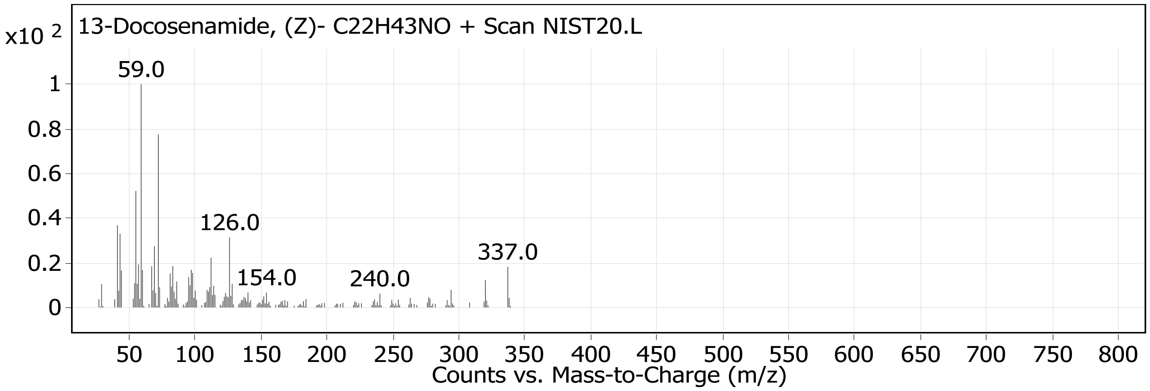

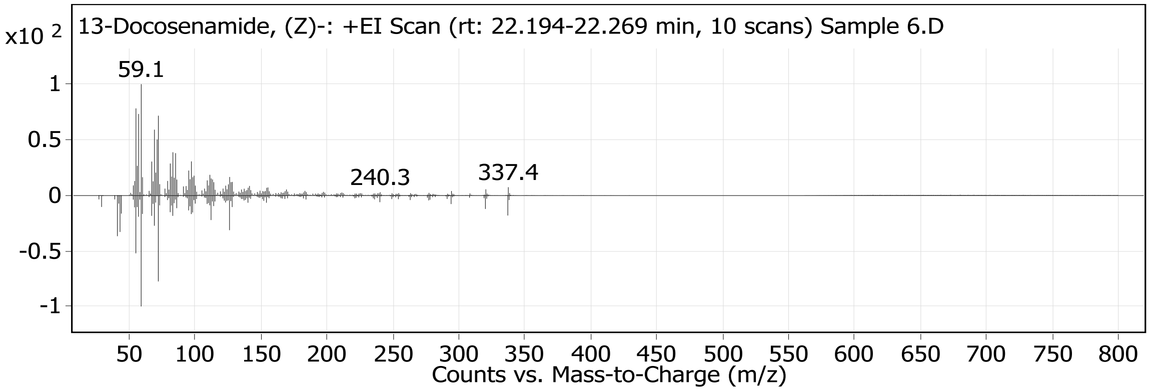

Spectrum Structure

13-Docosenamide, (Z)-

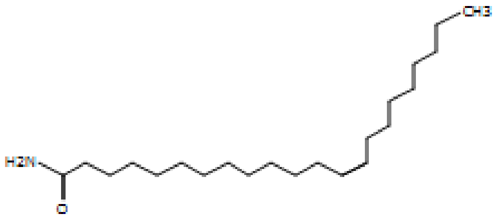

Spectrum Source	Collision Energy	Ionization Mode
Peak (14) in "+ TIC Scan"	0	EI

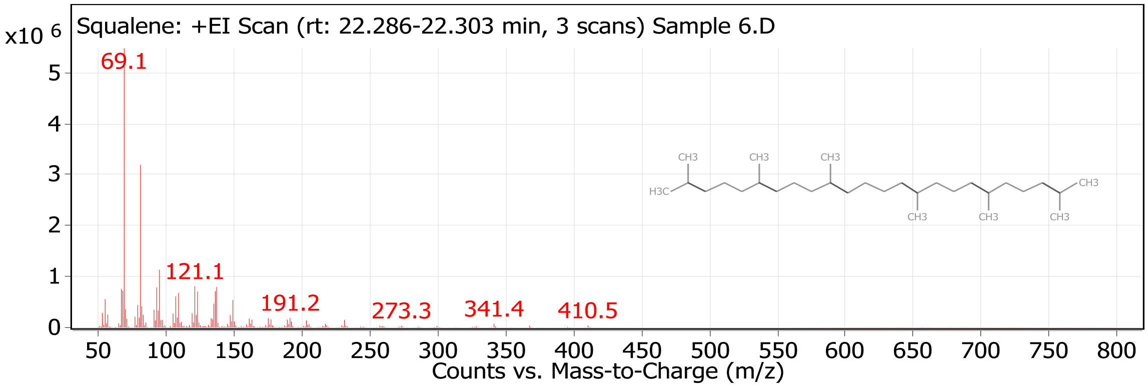

Library Spectrum

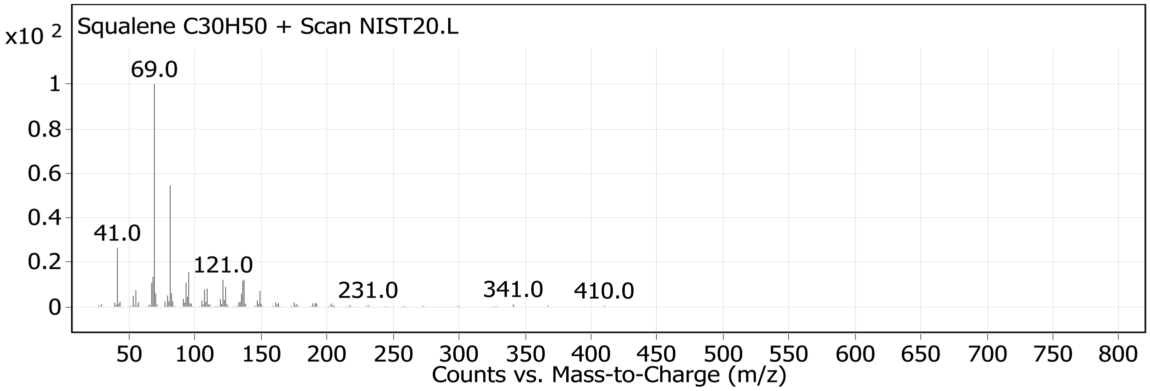

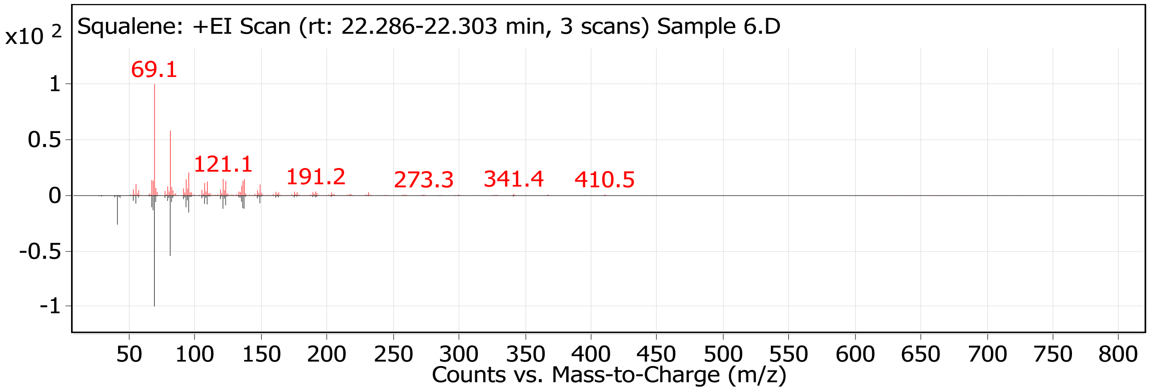

Spectrum Structure

Squalene

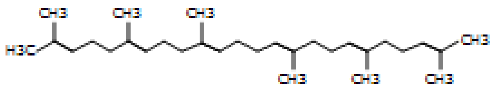

Spectrum Source	Collision Energy	Ionization Mode
Peak (15) in "+ TIC Scan"	0	EI

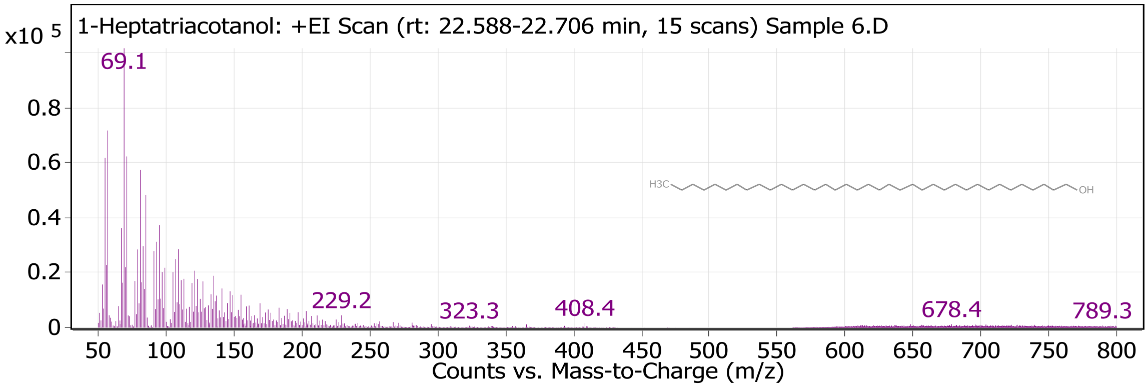

Library Spectrum

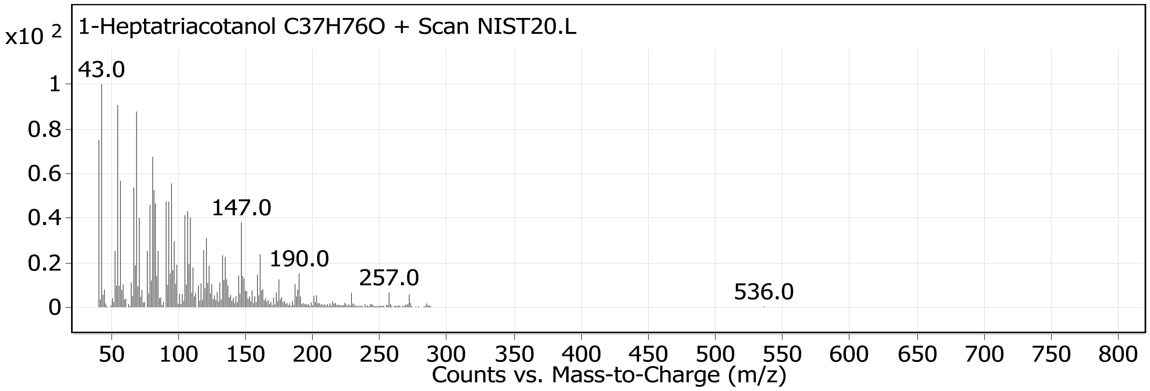

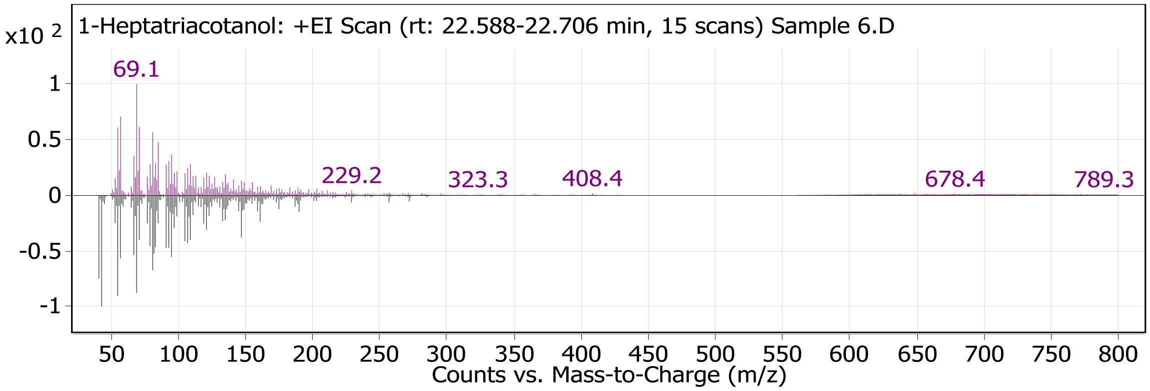

Spectrum Structure

1-Heptatriacotanol

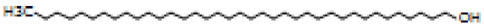

Qualitative Analysis Report

Spectrum Source	Collision Energy	Ionization Mode
Peak (16) in "+ TIC Scan"	0	EI

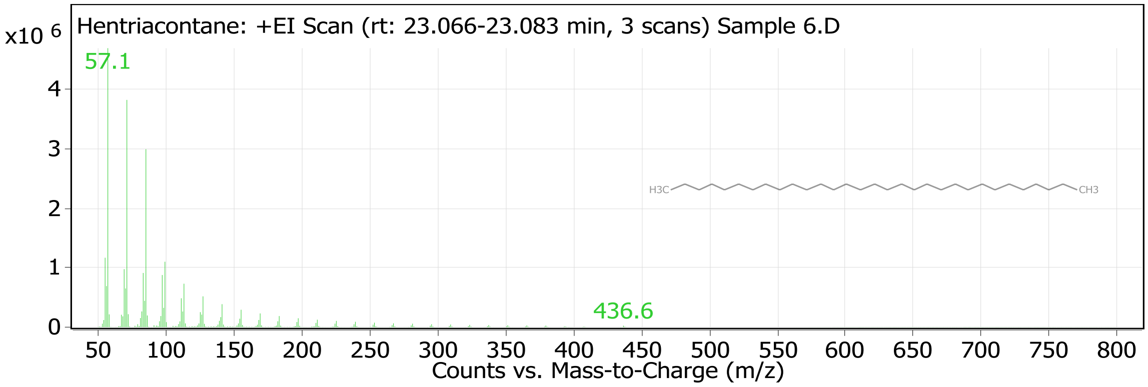

Library Spectrum

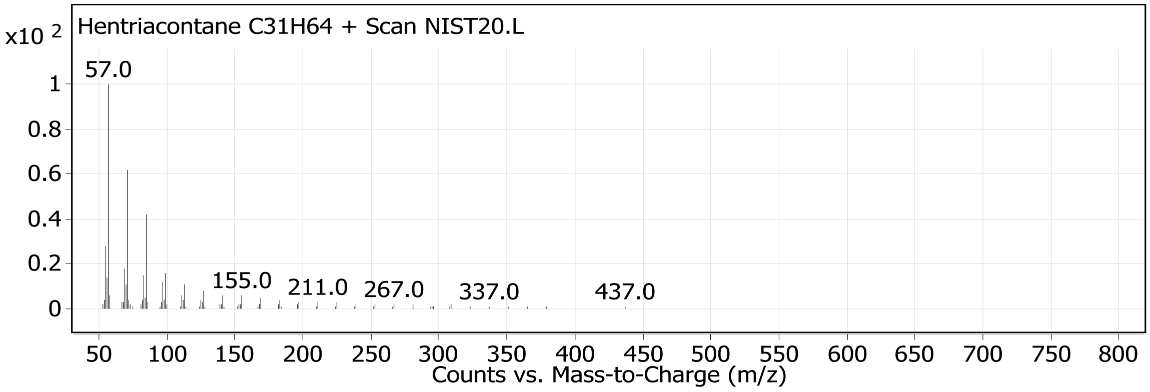

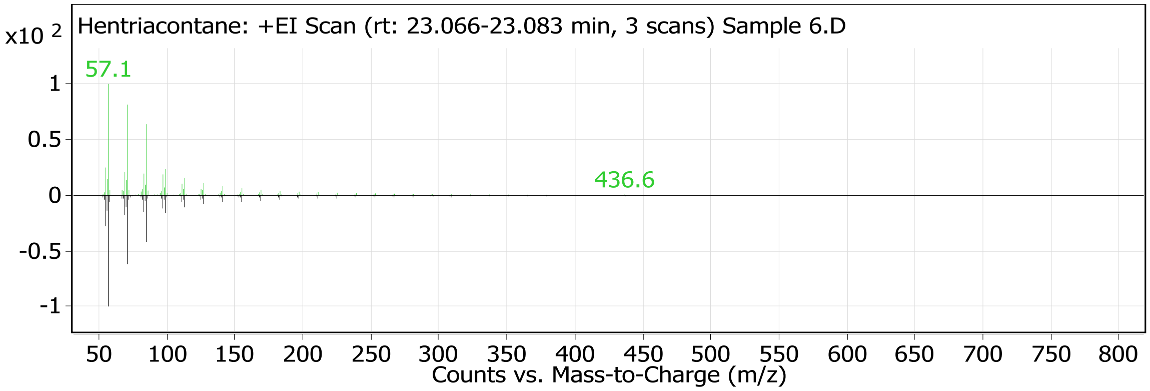

Spectrum Structure

Hentriacontane

Spectrum Source	Collision Energy	Ionization Mode
Peak (17) in "+ TIC Scan"	0	EI

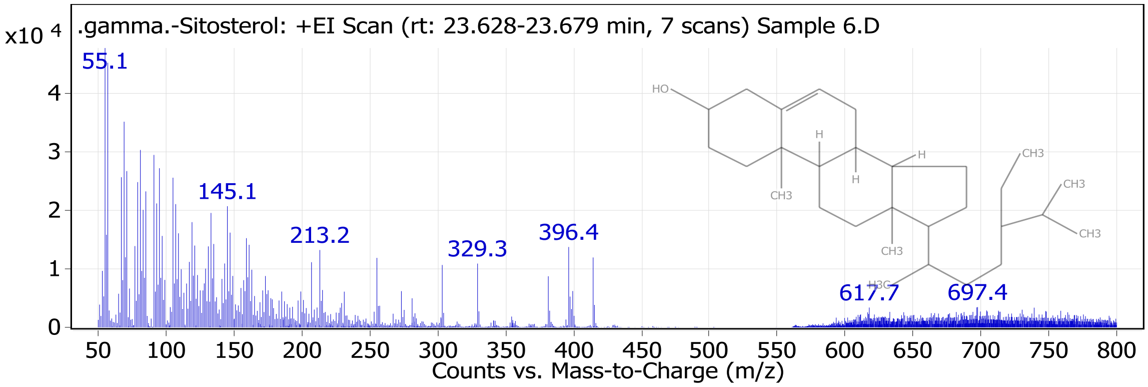

Library Spectrum

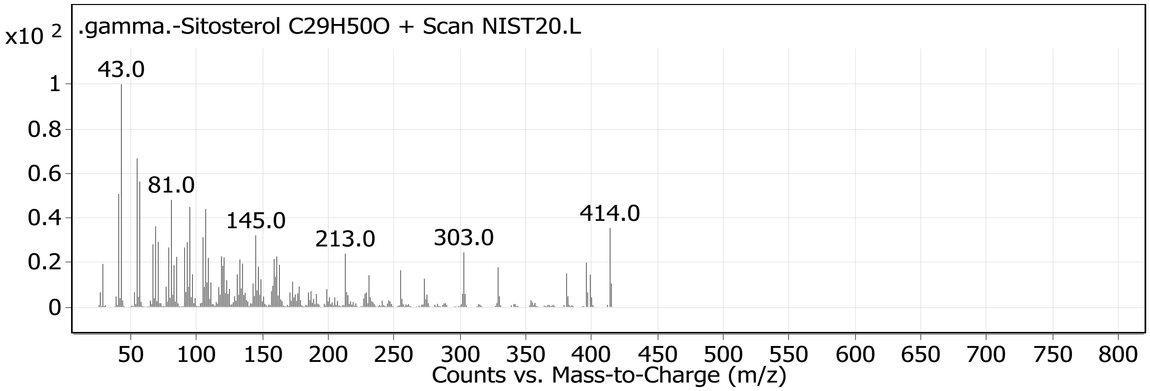

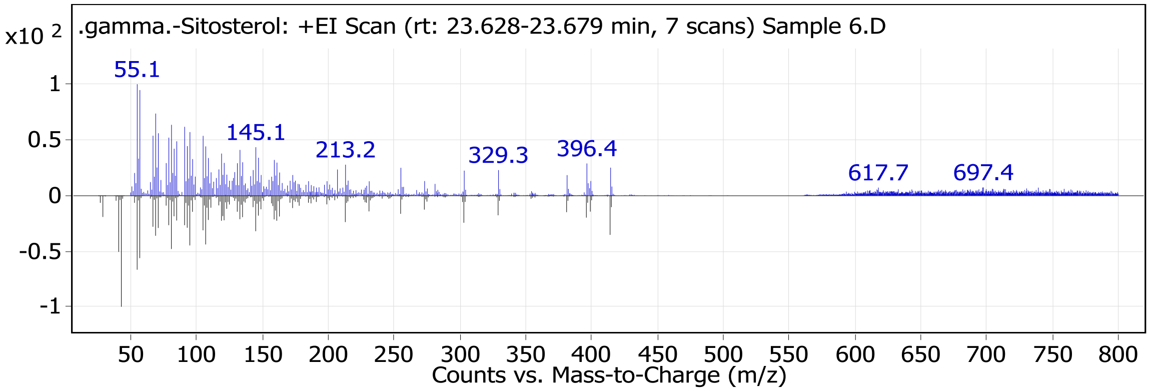

Spectrum Structure

.gamma.-Sitosterol

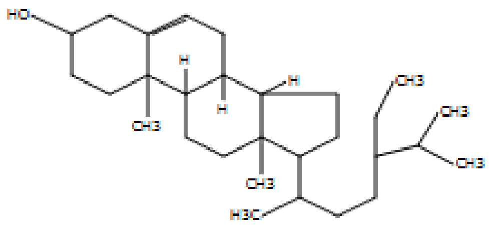

Spectrum Source	Collision Energy	Ionization Mode
Peak (18) in "+ TIC Scan"	0	EI

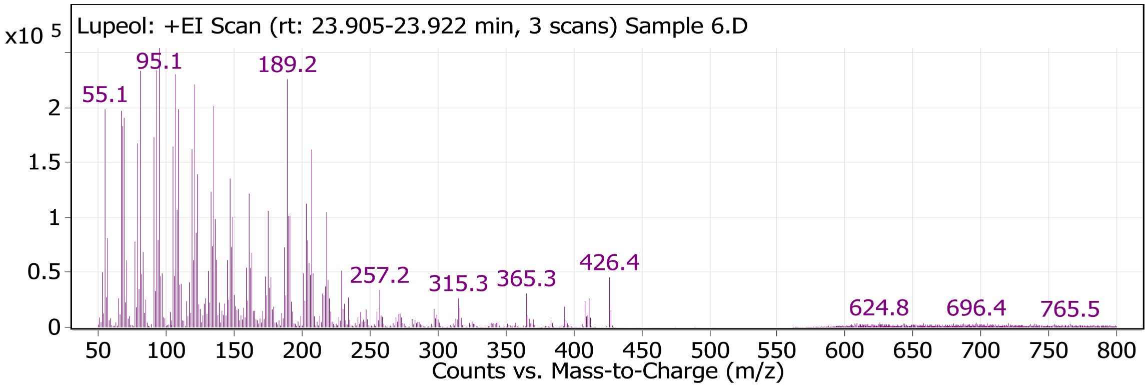

Library Spectrum

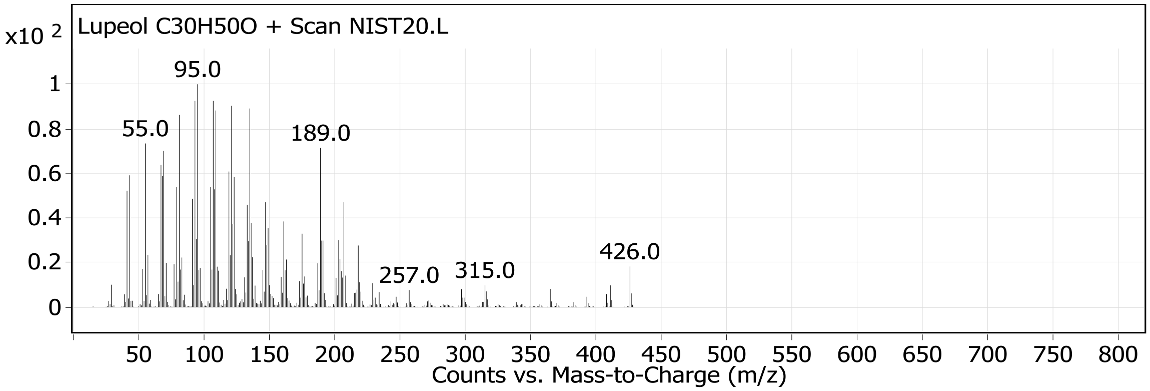

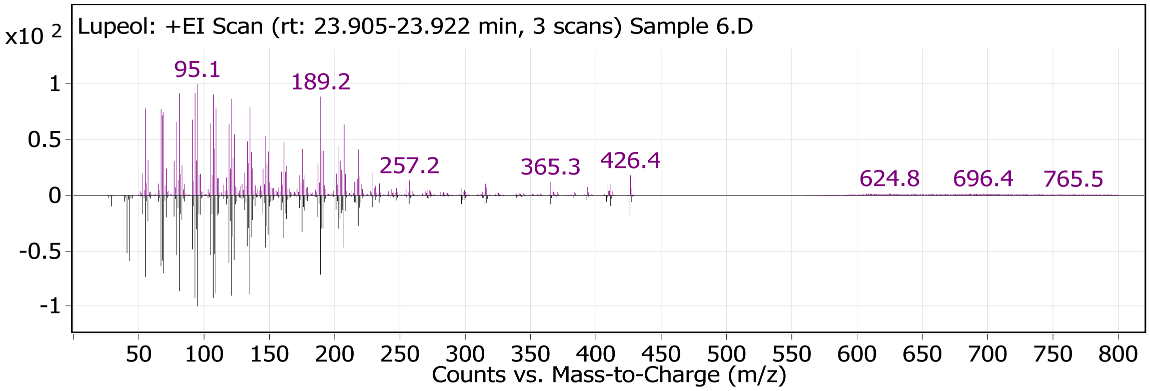

Spectrum Structure

Lupeol

--- End Of Report ---
