# Supplementary material for: Chemical and Molecular Insights into the Arid Wild Plant Diversity of Saudi Arabia
Source: Plants (Basel). 2026 Jan 19;15(2):295. doi: 10.3390/plants15020295 (PMC12845481; doi:10.3390/plants15020295)
Supplement: Supplementary file 1 [file plants-15-00295-s001.zip › Sample 6_AnalysisReport.pdf]

Qualitative Analysis Report

**Data Filename**	Sample 8.D	**Sample Name**	Sample 8
**Sample Type**		**Position**	1
**Instrument Name**	3	**User Name**	
**Acq Method**	Scan DB-5MS Hydrogen 2024.M	**Acquired Time**	6/25/2024 12:18:02 PM
**IRM Calibration Status**	Not Applicable	**DA Method**	SignalToNoiseCheckout.m
**Comment**			

**Expected Barcode**		**Sample Amount**	
**Dual Inj Vol**	0.2	**TuneName**	ATUNE.U
**TunePath**	D:\MassHunter\GCMS\3\5977 \	**TuneDateStamp**	2024-06-23T14:01:57+02:00
**MSFirmwareVersion**	6.00.34	**OperatorName**	
**RunCompletedFlag**	True	**Acquisition SW Version**	MassHunter GC/MS Acquisition 10.0.368 14-Feb-2019 Copyright © 1989-2018 Agilent Technologies, Inc

User Chromatograms

Fragmentor Voltage **Collision Energy** 0 **Ionization Mode** EI

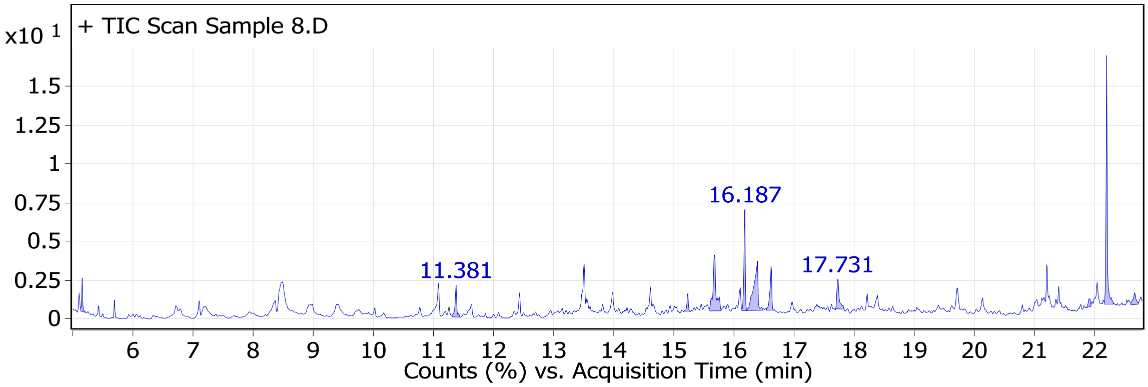

Integration Peak List

Peak	Start	RT	End	Height	Area	Area %
1	5.131	5.156	5.215	1315566.67	1413605.53	10.35
2	11.314	11.381	11.446	1241584.69	2497660.23	18.29
3	15.181	15.239	15.268	684243.82	1107756.4	8.11
4	15.592	15.684	15.793	2190001.61	8183307.67	59.94
5	16.137	16.187	16.221	3951937.6	5878141.86	43.05
6	16.221	16.397	16.489	1938783.3	9501020.1	69.59
7	16.548	16.624	16.685	1739144.75	3713816.85	27.2
8	17.7	17.731	17.839	1148942.65	3272022.05	23.97
9	21.879	21.917	21.95	316871.52	789261.93	5.78
10	22.169	22.21	22.32	9740782.69	13652863.56	100
11	22.613	22.672	22.739	459759.89	1589139.83	11.64

User Spectra

**Spectrum Source**	**Collision Energy**	**Ionization Mode**
Peak (1) in "+ TIC Scan"	0	EI

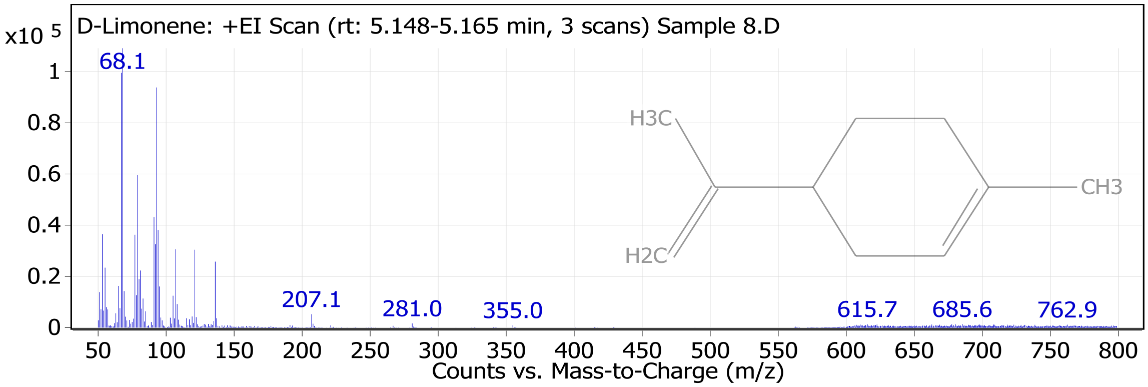

Library Spectrum

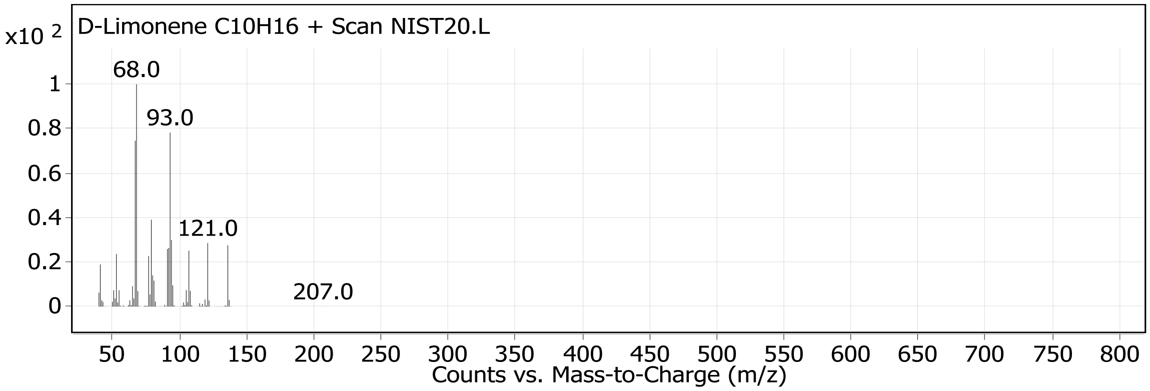

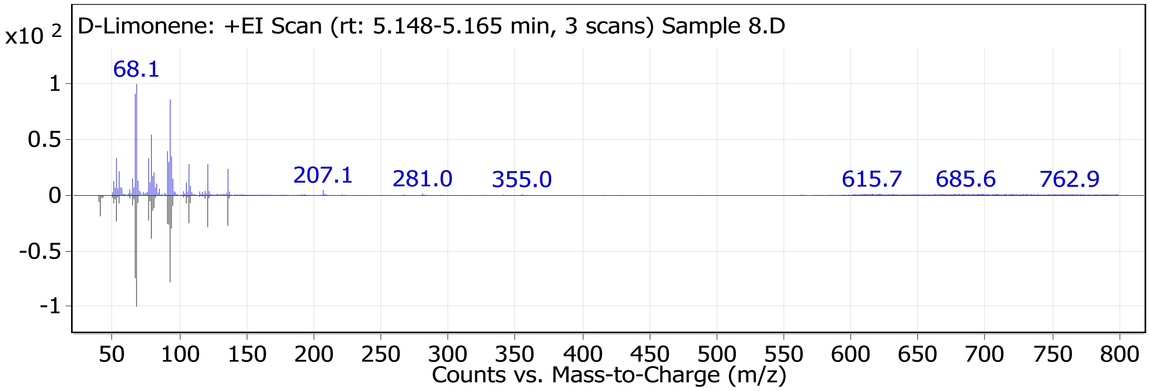

Spectrum Structure

D-Limonene

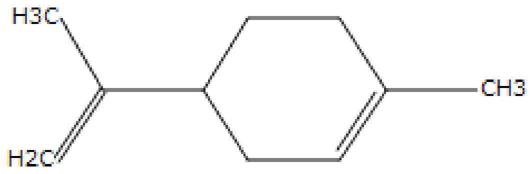

Spectrum Source	Collision Energy	Ionization Mode
Peak (2) in "+ TIC Scan"	0	EI

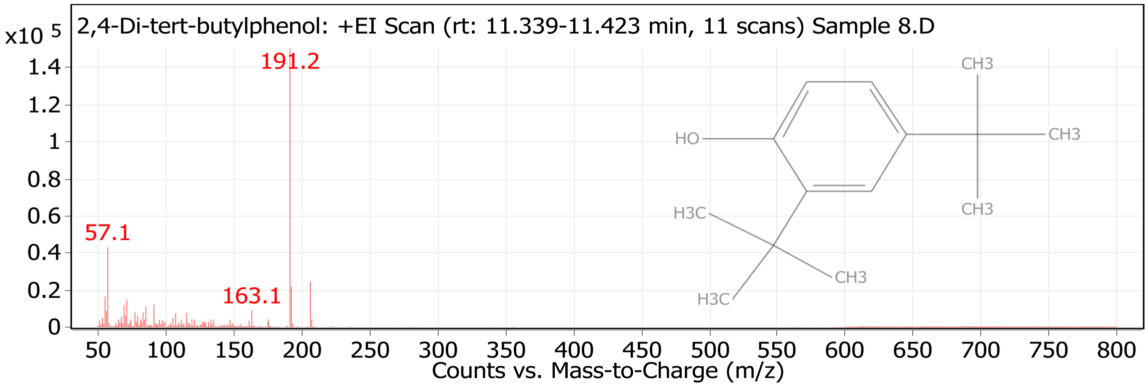

Library Spectrum

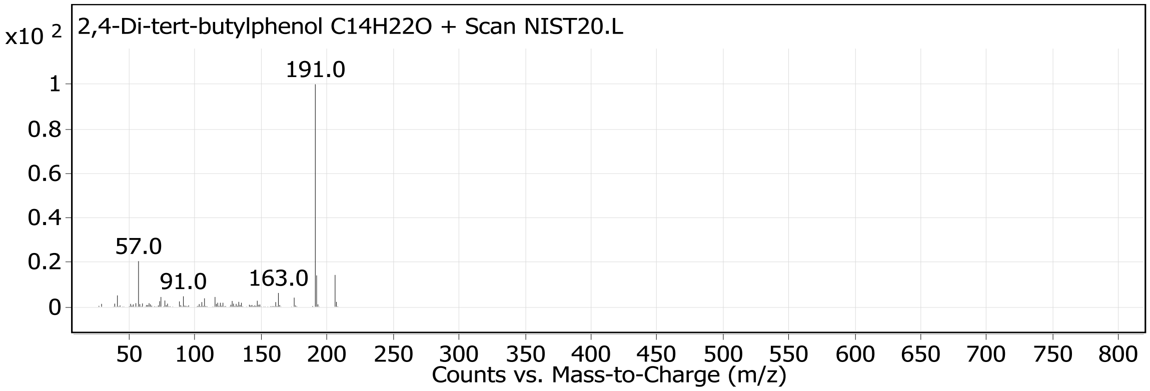

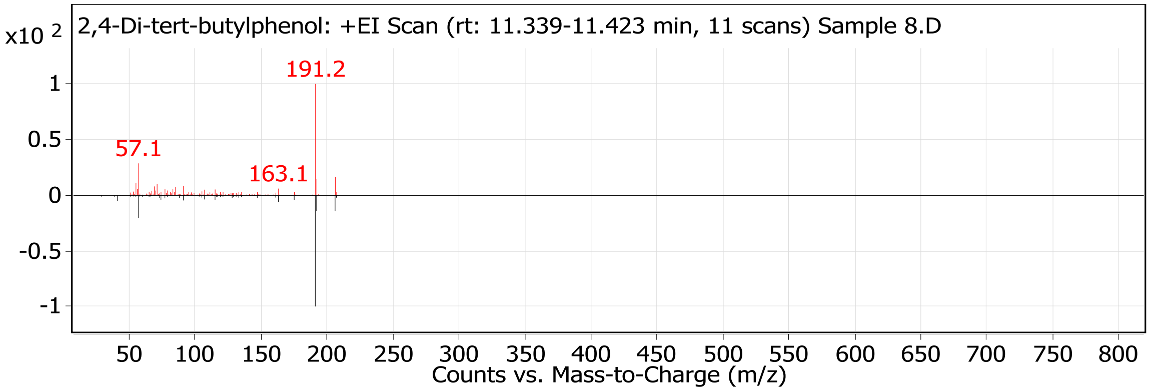

Spectrum Structure

2,4-Di-tert-butylphenol

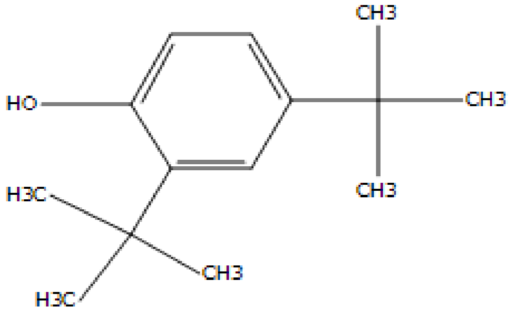

**Spectrum Source**	**Collision Energy**	**Ionization Mode**
Peak (3) in "+ TIC Scan"	0	EI

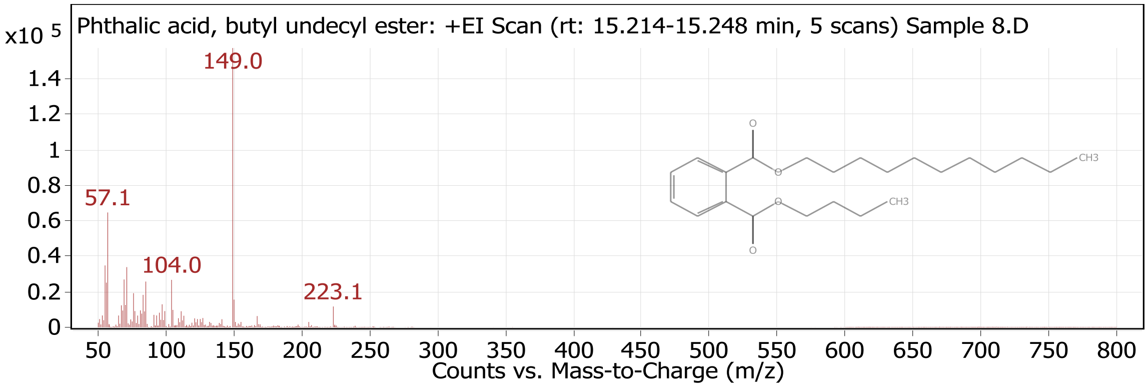

Library Spectrum

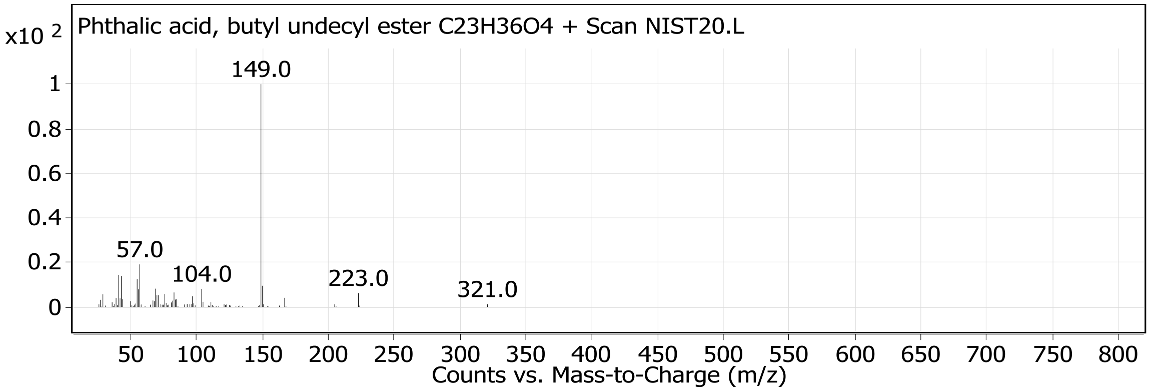

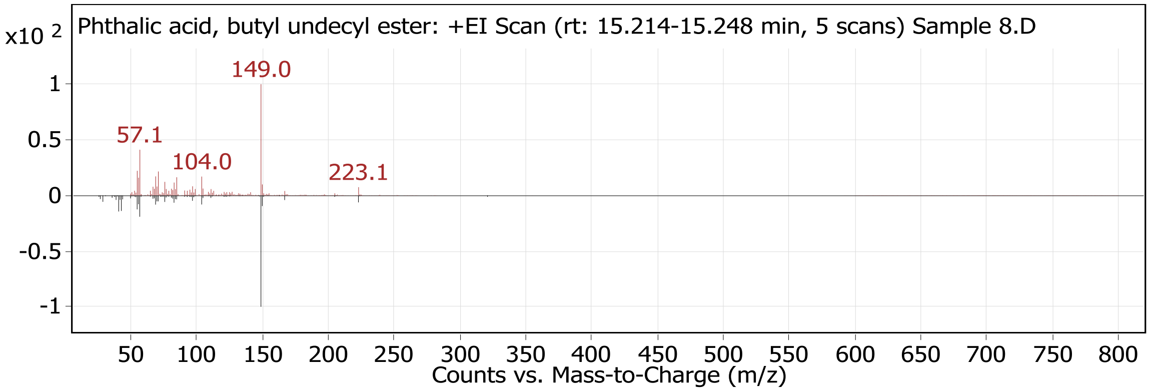

Spectrum Structure

Phthalic acid, butyl undecyl ester

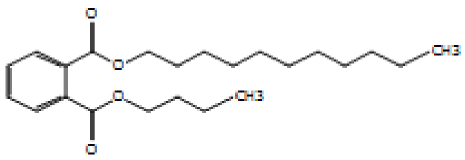

**Spectrum Source**	**Collision Energy**	**Ionization Mode**
Peak (4) in "+ TIC Scan"	0	EI

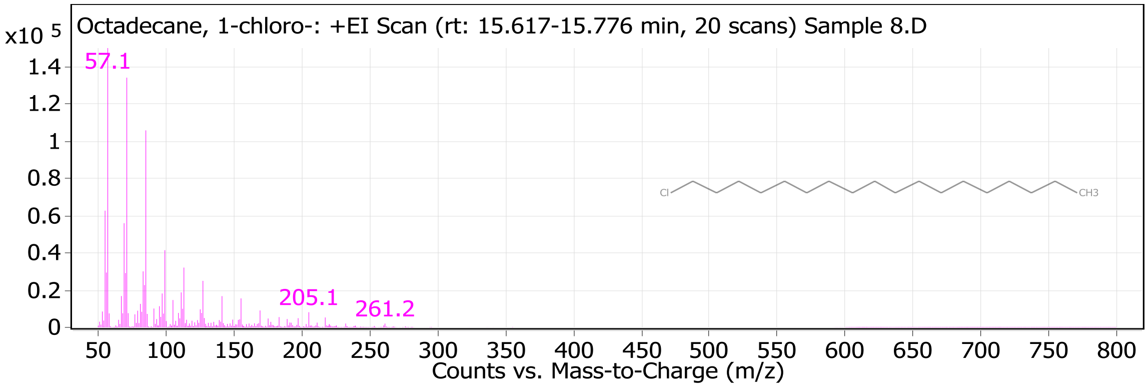

Library Spectrum

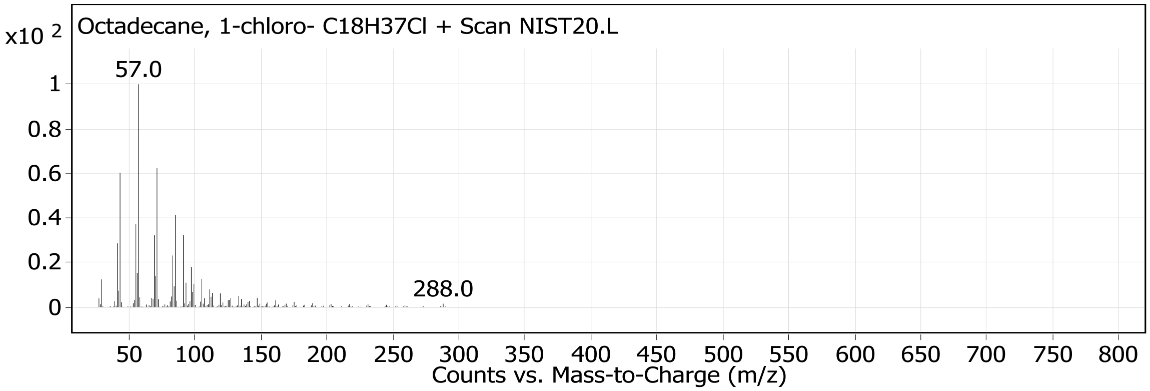

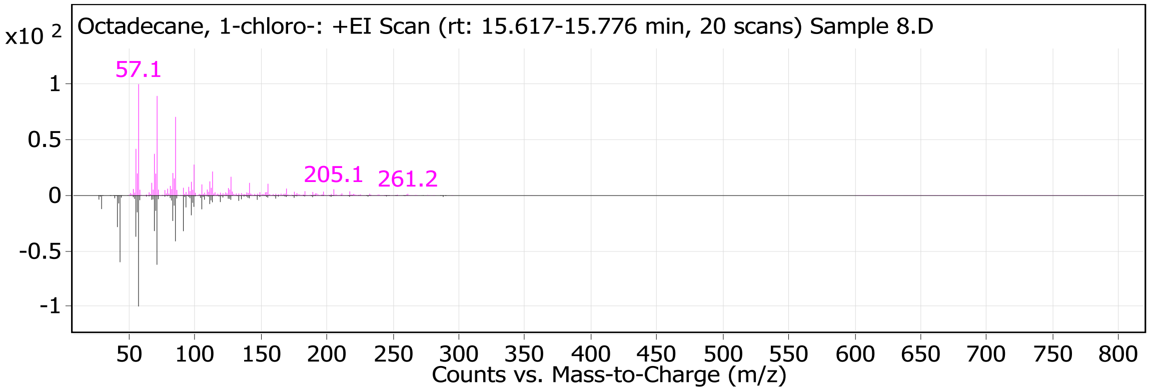

Spectrum Structure

Octadecane, 1-chloro-

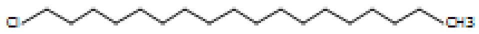

Spectrum Source	Collision Energy	Ionization Mode
Peak (5) in "+ TIC Scan"	0	EI

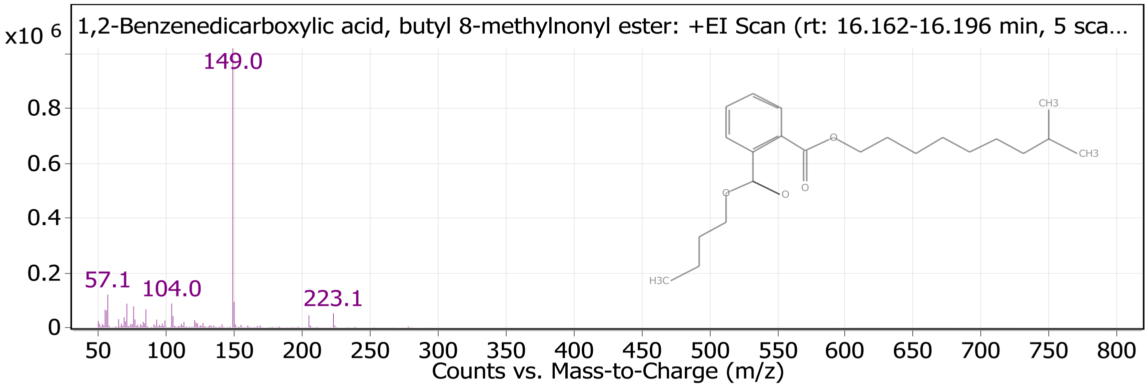

Library Spectrum

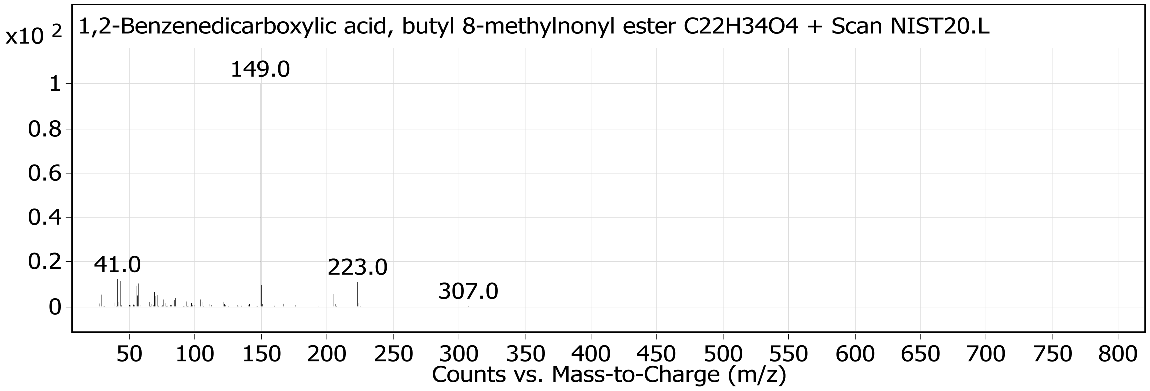

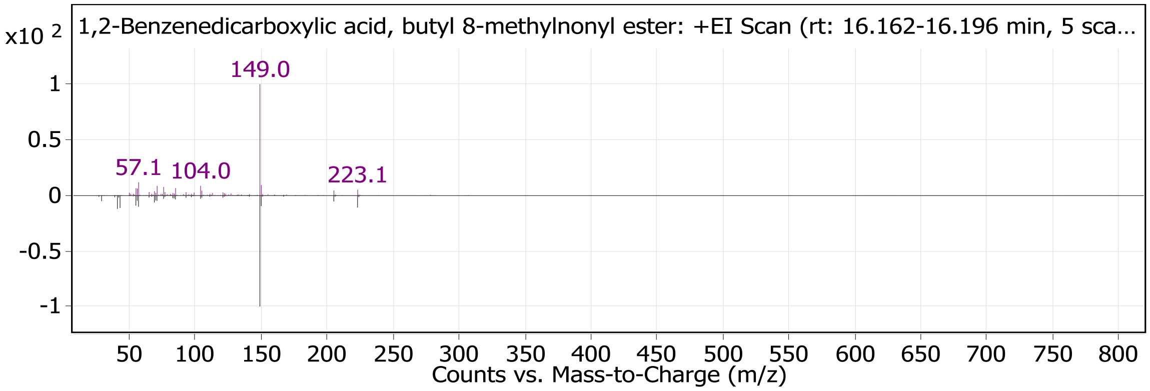

Spectrum Structure

1,2-Benzenedicarboxylic acid, butyl 8-methylnonyl ester

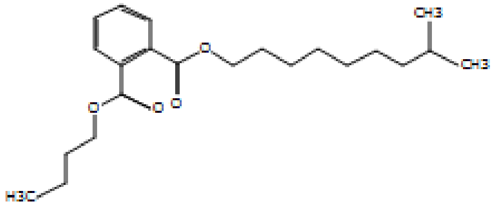

Spectrum Source	Collision Energy	Ionization Mode
Peak (6) in "+ TIC Scan"	0	EI

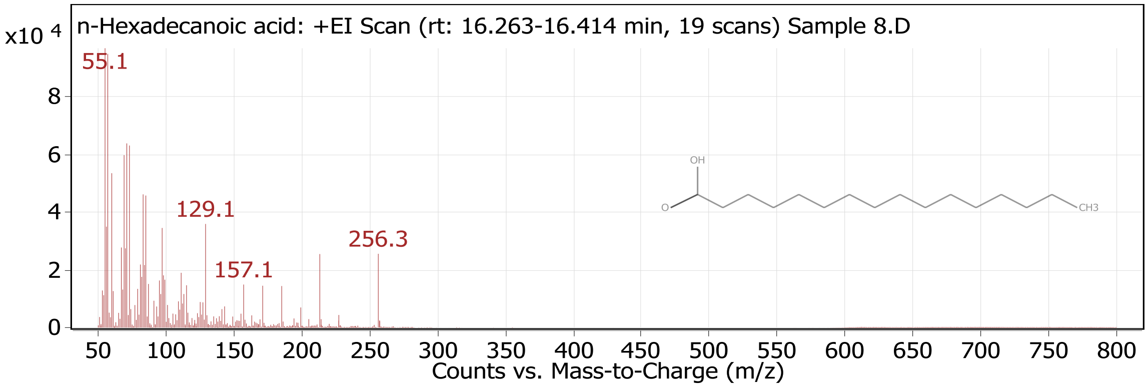

Library Spectrum

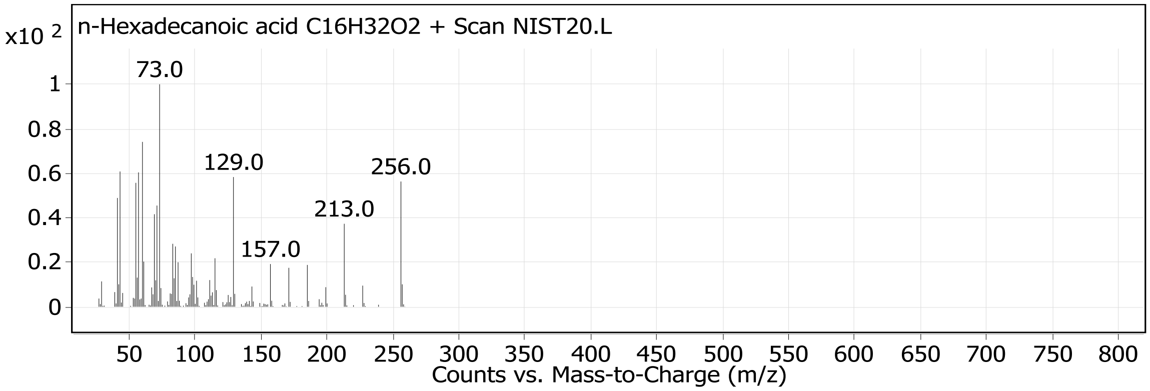

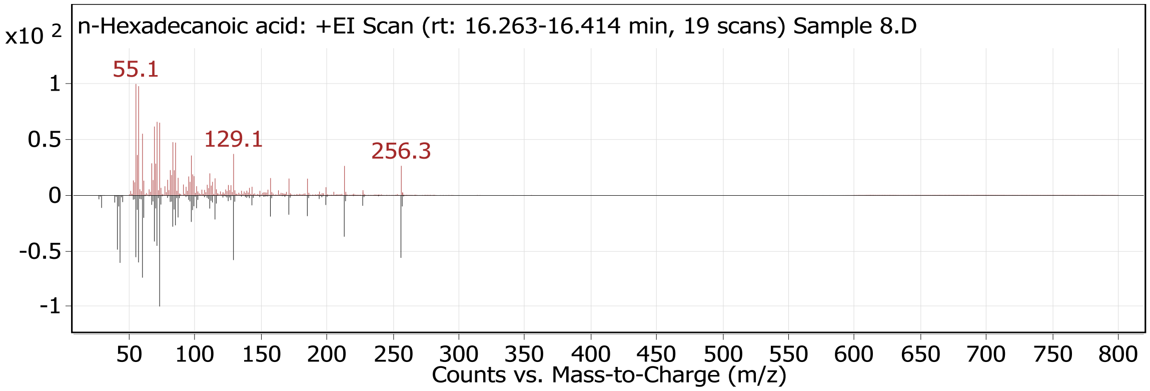

Spectrum Structure

n-Hexadecanoic acid

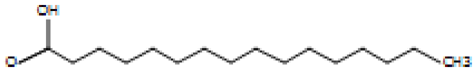

Spectrum Source	Collision Energy	Ionization Mode
Peak (7) in "+ TIC Scan"	0	EI

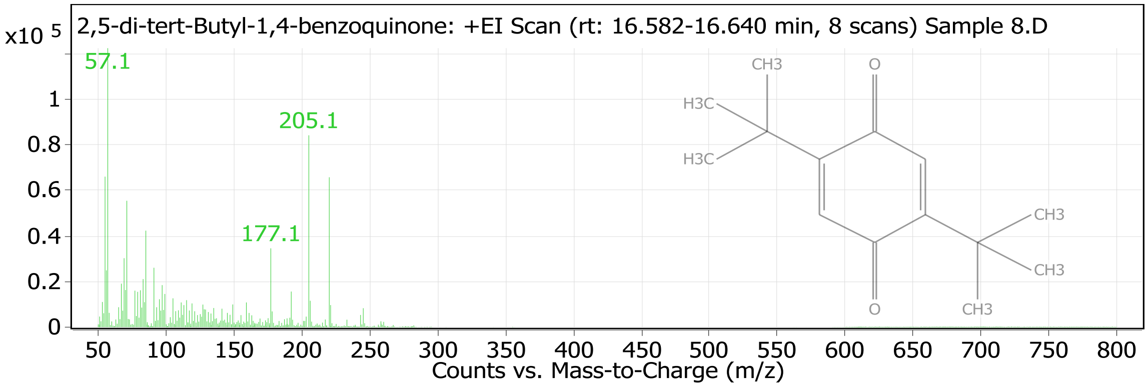

Library Spectrum

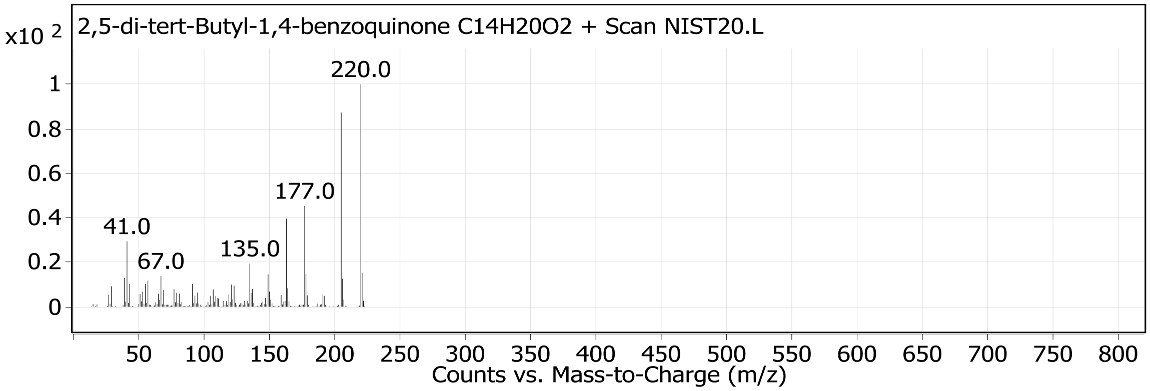

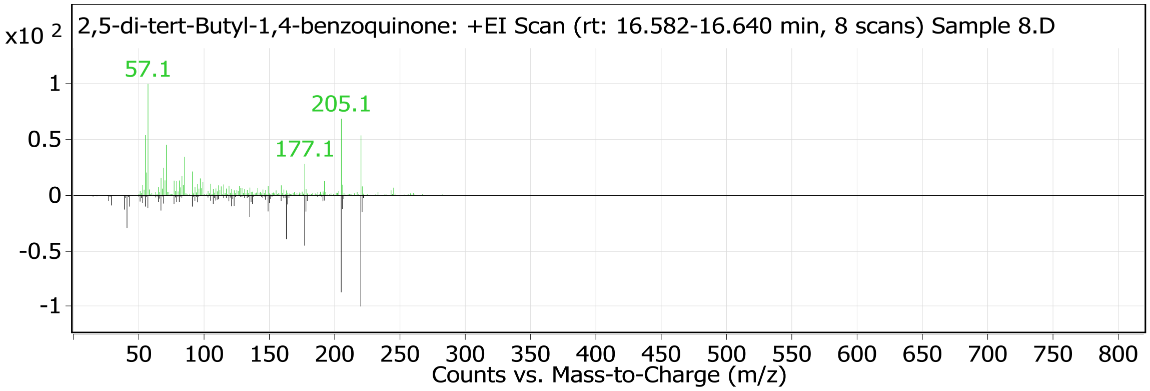

Spectrum Structure

2,5-di-tert-Butyl-1,4-benzoquinone

Qualitative Analysis Report

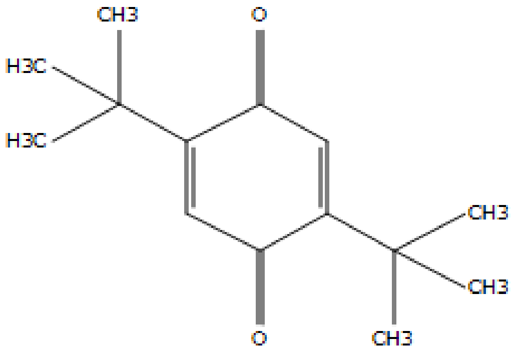

Spectrum Source	Collision Energy	Ionization Mode
Peak (8) in "+ TIC Scan"	0	EI

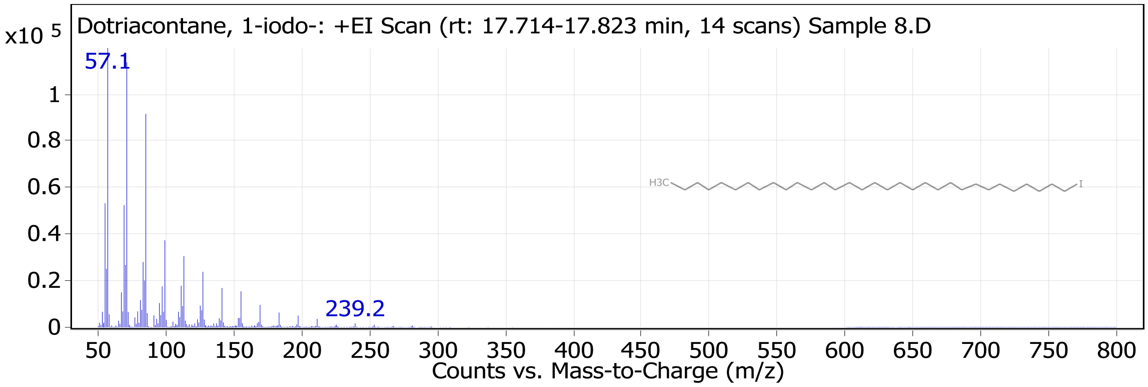

Library Spectrum

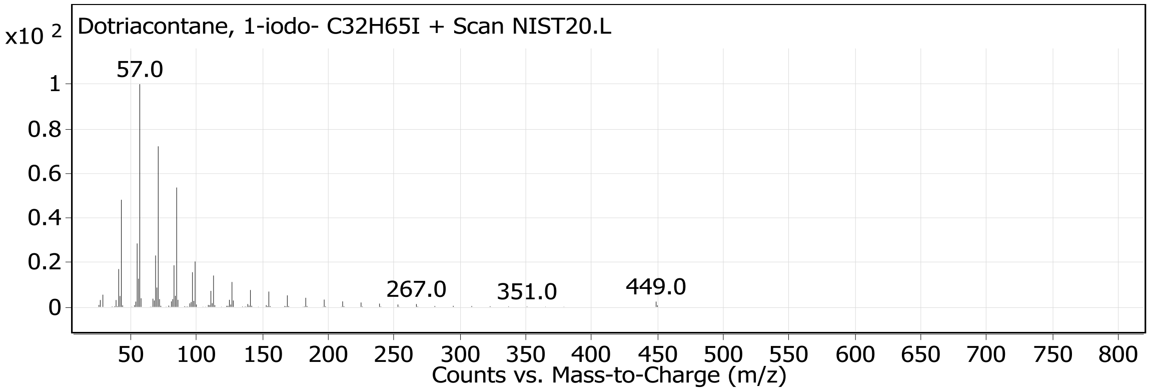

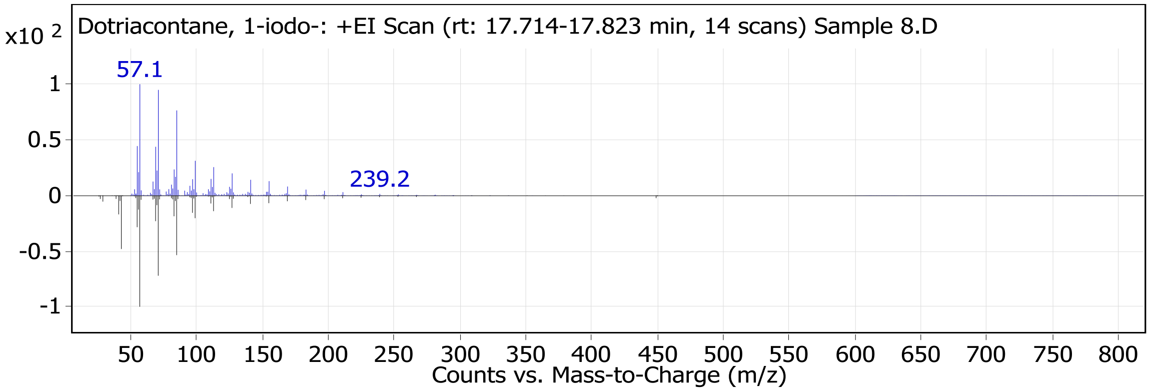

Spectrum Structure

Dotriacontane, 1-iodo-

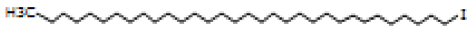

Spectrum Source	Collision Energy	Ionization Mode
Peak (9) in "+ TIC Scan"	0	EI

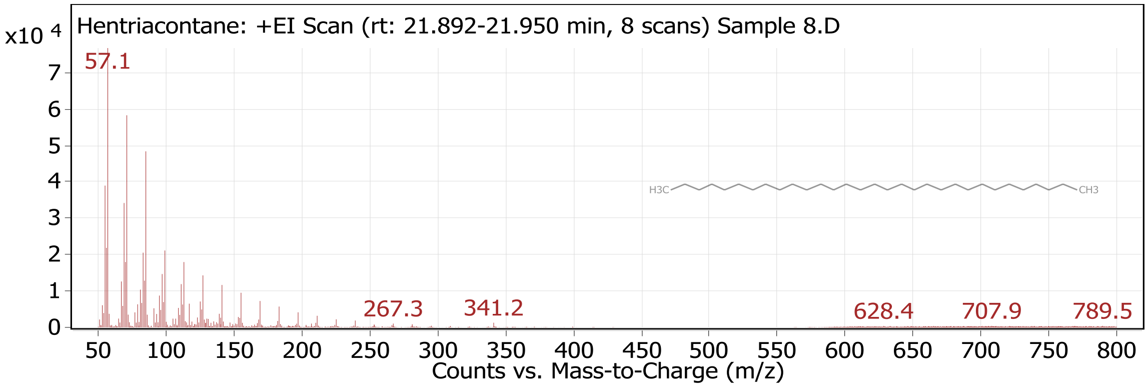

Library Spectrum

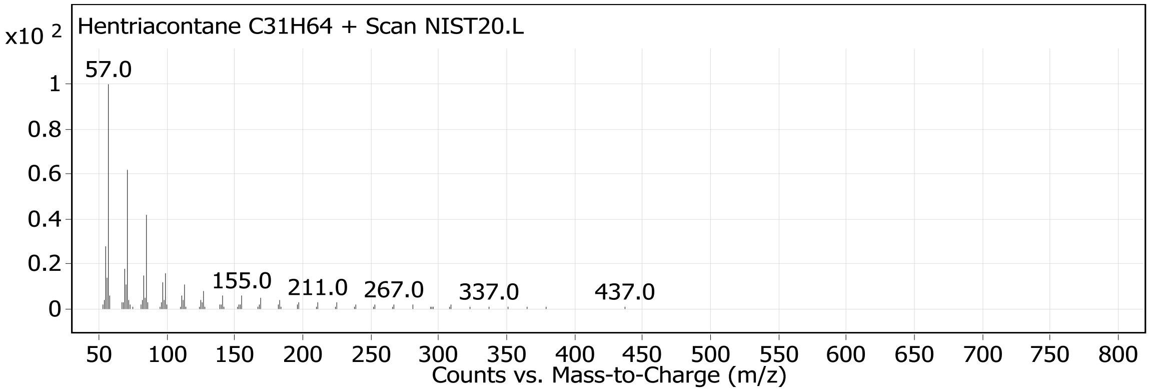

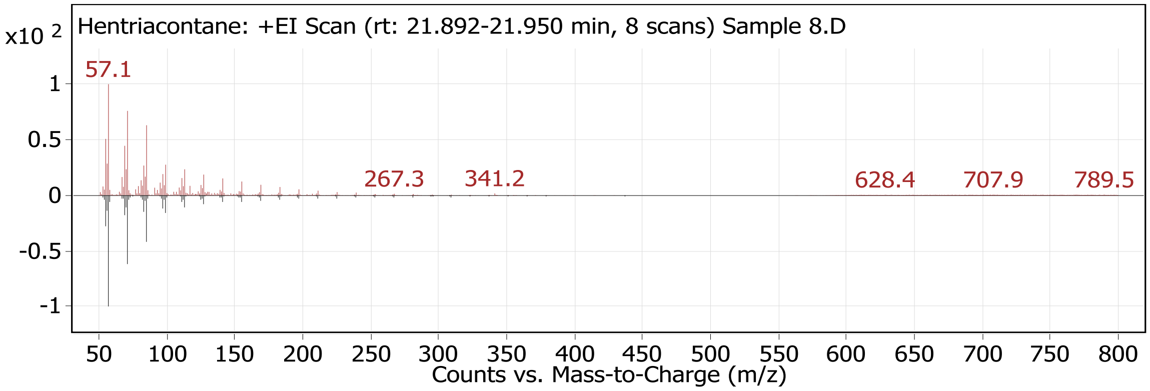
Hentriacontane: +EI Scan (rt: 21.892-21.950 min, 8 scans) Sample 8.D
x10 2
1
0.5
0
-0.5
-1
57.1
267.3
341.2
628.4
707.9
789.5
50 100 150 200 250 300 350 400 450 500 550 600 650 700 750 800
Counts vs. Mass-to-Charge (m/z)

Spectrum Structure

Hentriacontane

Spectrum Source	Collision Energy	Ionization Mode
Peak (10) in "+ TIC Scan"	0	EI

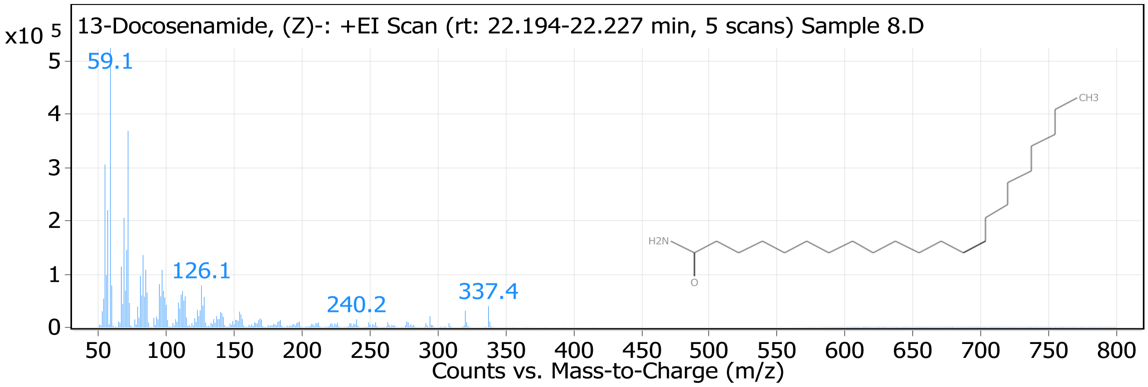

Library Spectrum

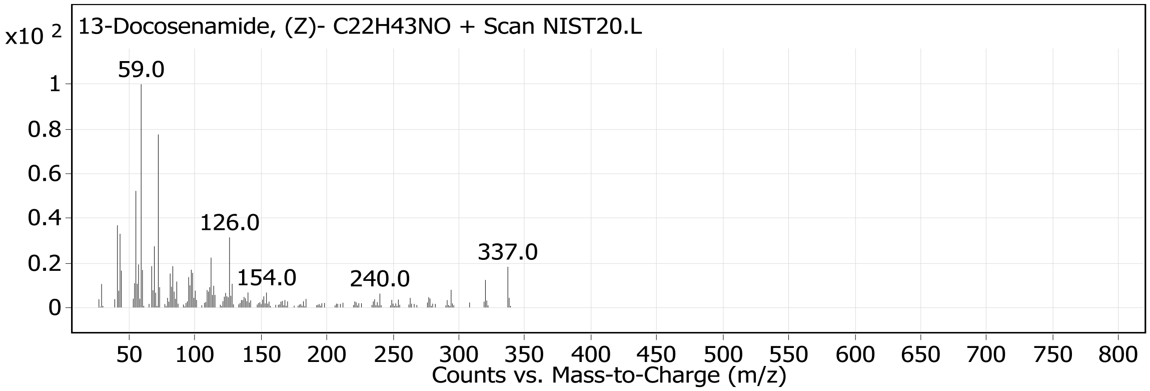

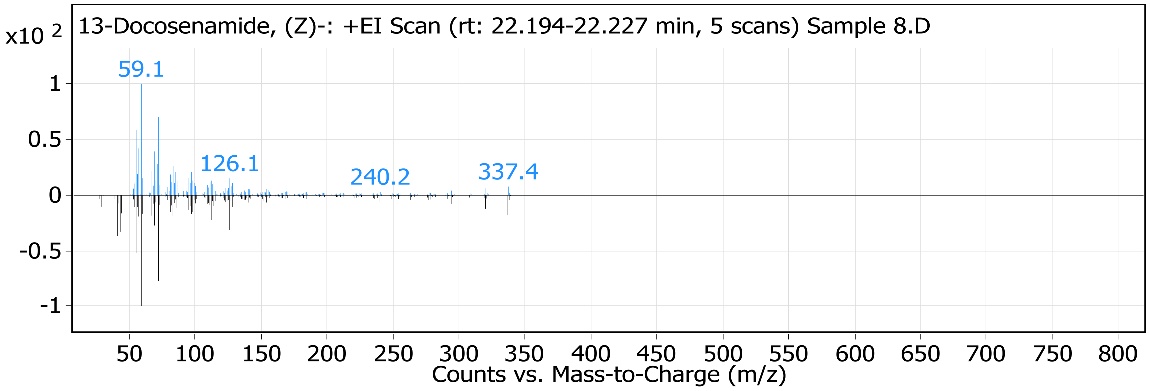

Spectrum Structure

13-Docosenamide, (Z)-

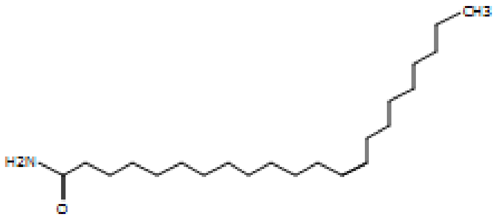

Spectrum Source	Collision Energy	Ionization Mode
Peak (11) in "+ TIC Scan"	0	EI

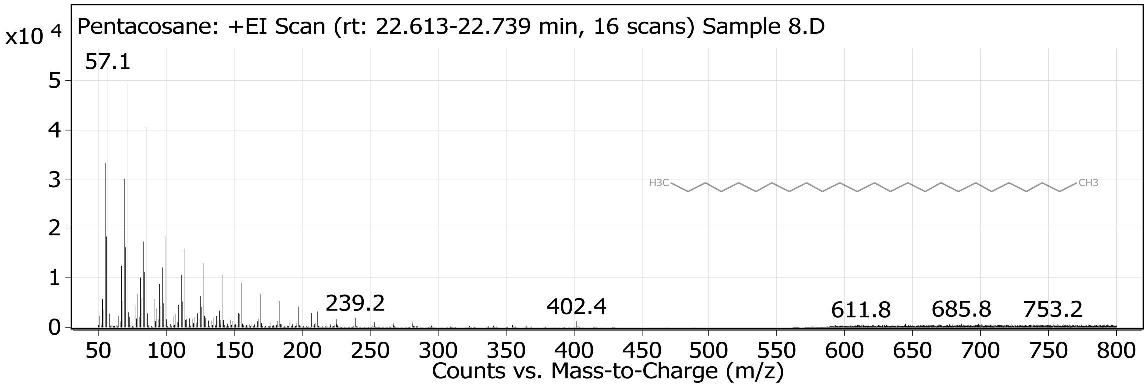

Library Spectrum

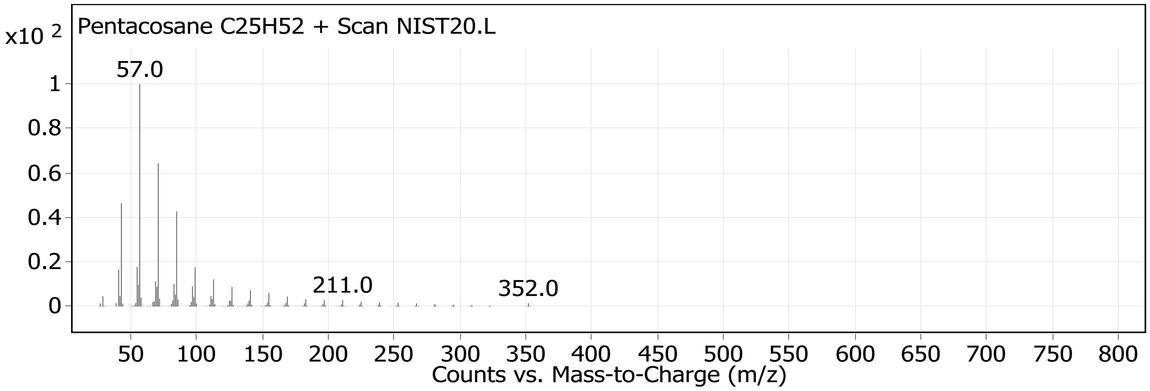

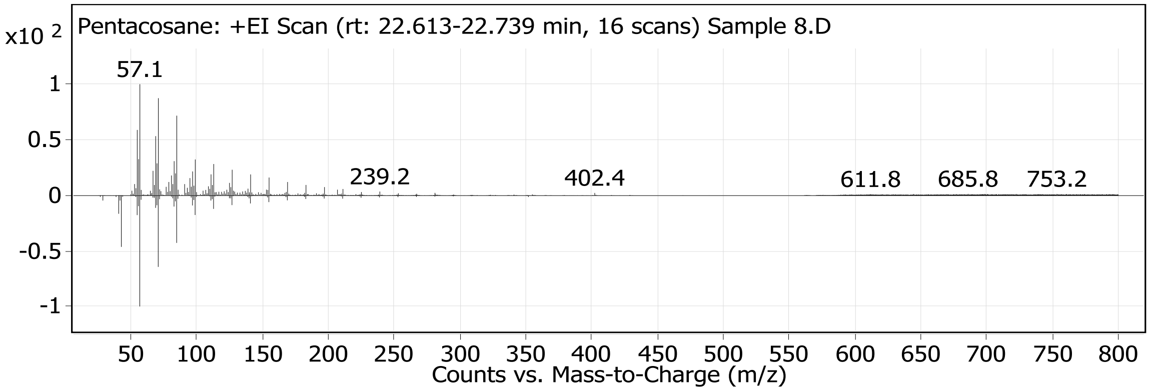

Spectrum Structure

Pentacosane

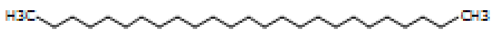

--- End Of Report ---
