# Supplementary material for: Chemical and Molecular Insights into the Arid Wild Plant Diversity of Saudi Arabia
Source: Plants (Basel). 2026 Jan 19;15(2):295. doi: 10.3390/plants15020295 (PMC12845481; doi:10.3390/plants15020295)
Supplement: Supplementary file 1 [file plants-15-00295-s001.zip › Sample 7_AnalysisReport.pdf]

Qualitative Analysis Report

**Data Filename**	Sample 14.D	**Sample Name**	Sample 14
**Sample Type**		**Position**	1
**Instrument Name**	3	**User Name**	
**Acq Method**	Scan DB-5MS Hydrogen 2024.M	**Acquired Time**	6/25/2024 4:56:01 PM
**IRM Calibration Status**	Not Applicable	**DA Method**	SignalToNoiseCheckout.m
**Comment**			

**Expected Barcode**		**Sample Amount**	
**Dual Inj Vol**	0.2	**TuneName**	ATUNE.U
**TunePath**	D:\MassHunter\GCMS\3\5977 \	**TuneDateStamp**	2024-06-23T14:01:57+02:00
**MSFirmwareVersion**	6.00.34	**OperatorName**	
**RunCompletedFlag**	True	**Acquisition SW Version**	MassHunter GC/MS Acquisition 10.0.368 14-Feb-2019 Copyright © 1989-2018 Agilent Technologies, Inc

User Chromatograms

Fragmentor Voltage **Collision Energy** 0 **Ionization Mode** EI

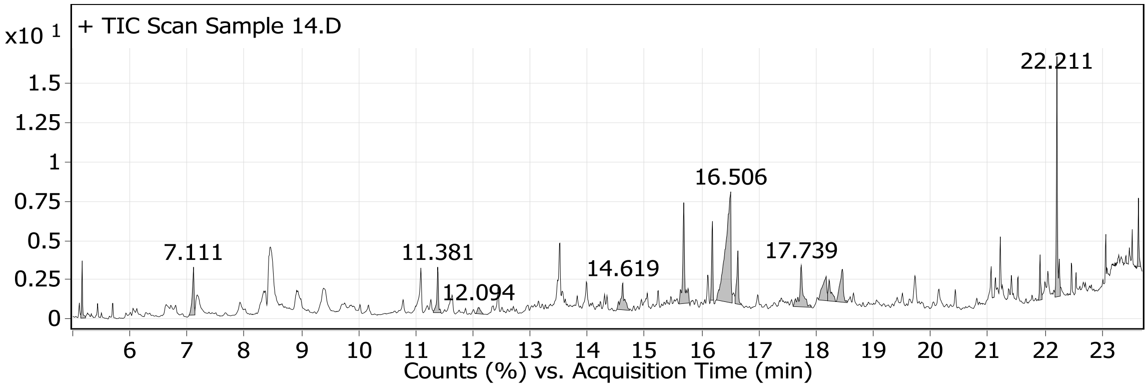

Integration Peak List

Peak	Start	RT	End	Height	Area	Area %
1	5.139	5.165	5.223	2431250.77	2665301.9	8.68
2	7.033	7.111	7.136	2033094.44	4313572.24	14.04
3	11.305	11.381	11.439	1935100.18	3812234.07	12.41
4	12.044	12.094	12.182	270475.59	1059595.25	3.45
5	14.514	14.619	14.736	1125792.72	4837050.47	15.75
6	15.6	15.684	15.784	4280286.66	10707398.09	34.86
7	16.15	16.187	16.208	3349849.5	4922563.02	16.03
8	16.256	16.506	16.531	4711595.73	30713298.57	100
9	16.582	16.632	16.7	2268080.36	4811552.68	15.67
10	17.597	17.739	17.907	1761025.94	7654382.82	24.92
11	18.041	18.176	18.201	1030747.83	5436247.67	17.7
12	18.201	18.226	18.343	893155.75	3198877.22	10.42
13	18.345	18.461	18.545	1396384.09	6070859.71	19.77
14	21.883	21.917	21.951	1922033.2	2680979.86	8.73
15	22.177	22.211	22.269	10200555.08	14057493.35	45.77
16	23.614	23.637	23.678	2901811.12	2997285.74	9.76

User Spectra

Spectrum Source **Collision Energy** **Ionization Mode**

Peak (1) in "+ TIC Scan" 0 EI

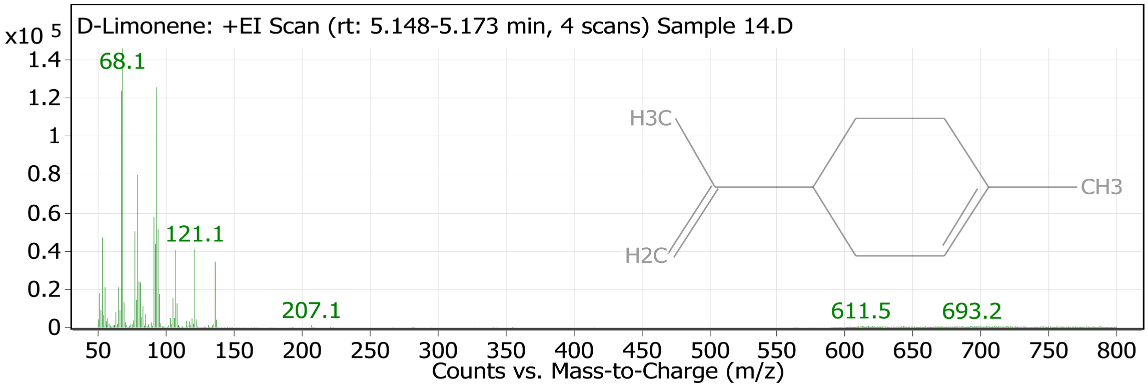

Library Spectrum

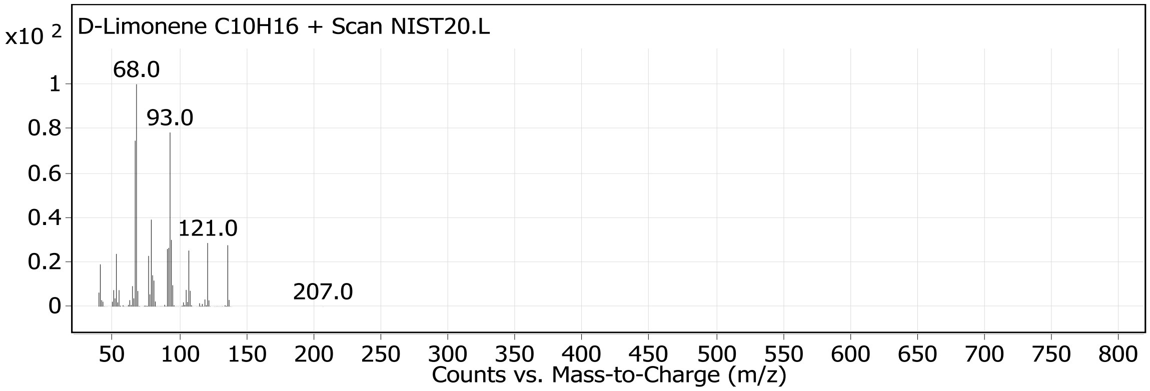

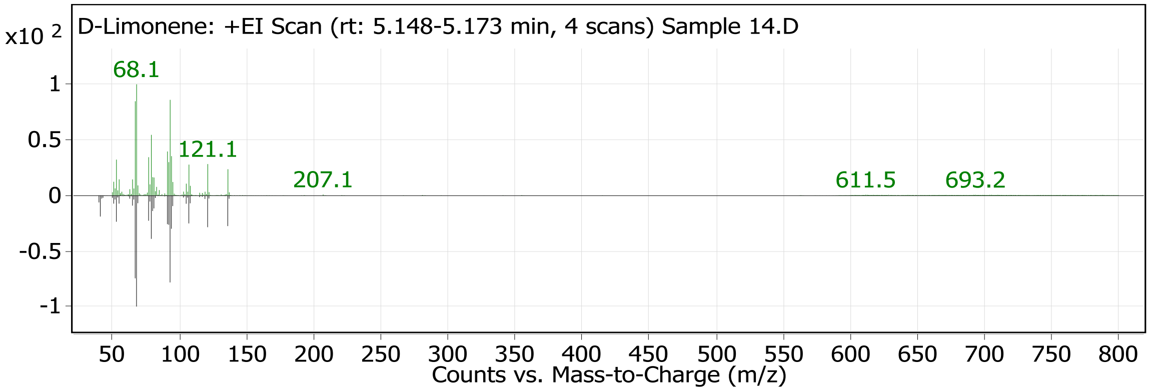

Spectrum Structure

D-Limonene

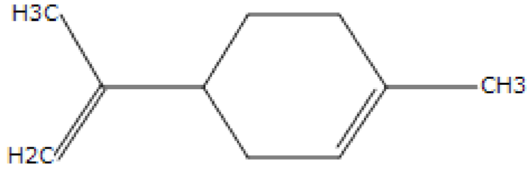

Spectrum Source	Collision Energy	Ionization Mode
Peak (2) in "+ TIC Scan"	0	EI

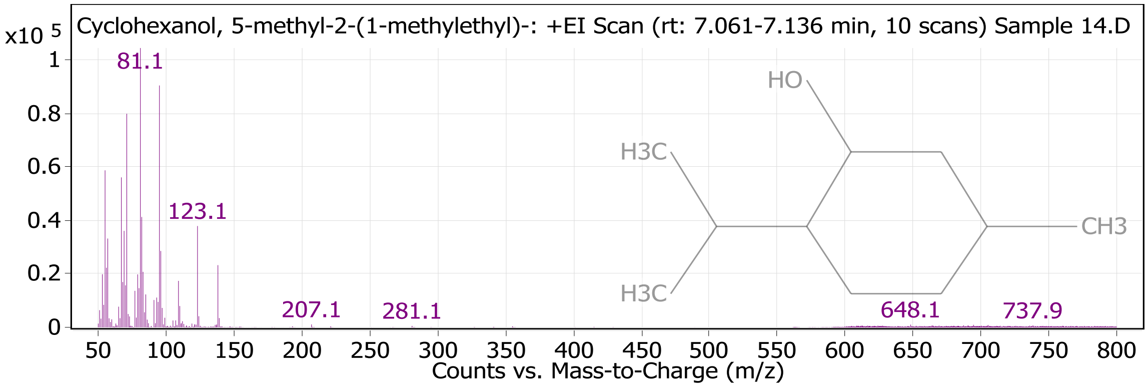

Library Spectrum

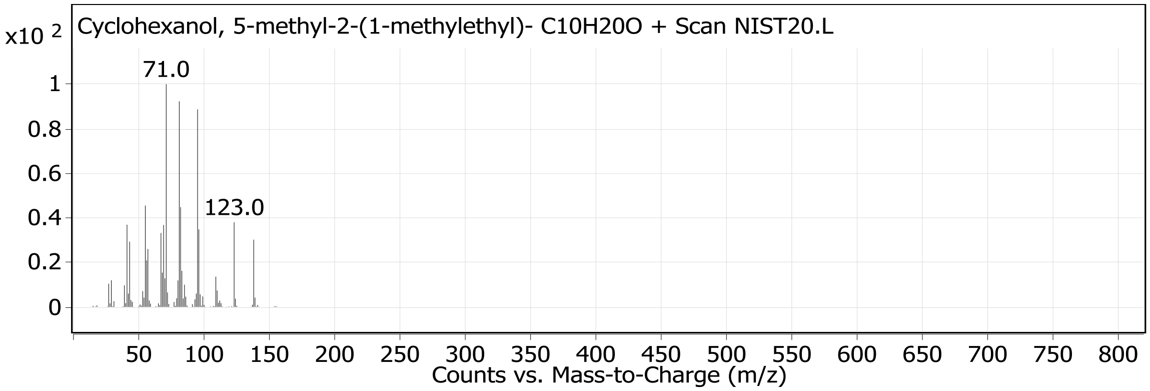

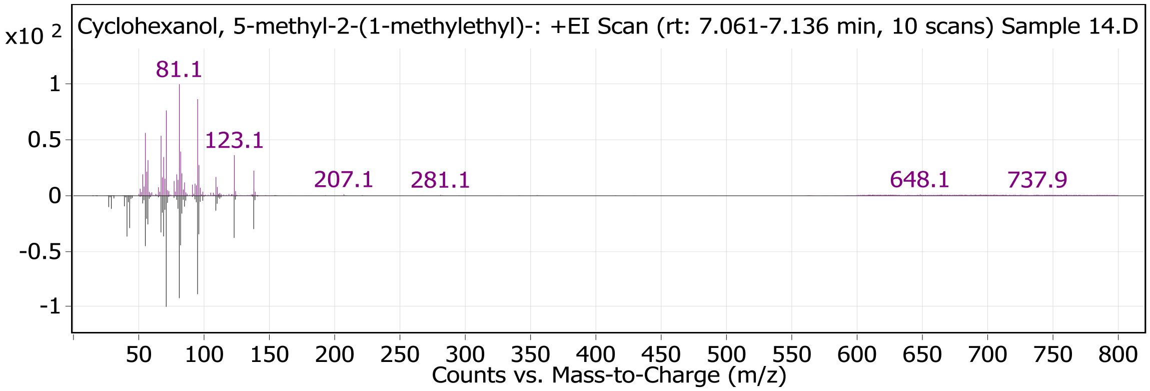

Spectrum Structure

Cyclohexanol, 5-methyl-2-(1-methylethyl)-

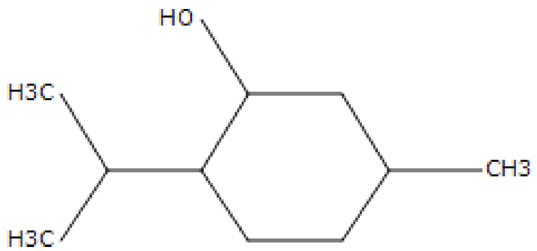

**Spectrum Source**	**Collision Energy**	**Ionization Mode**
Peak (3) in "+ TIC Scan"	0	EI

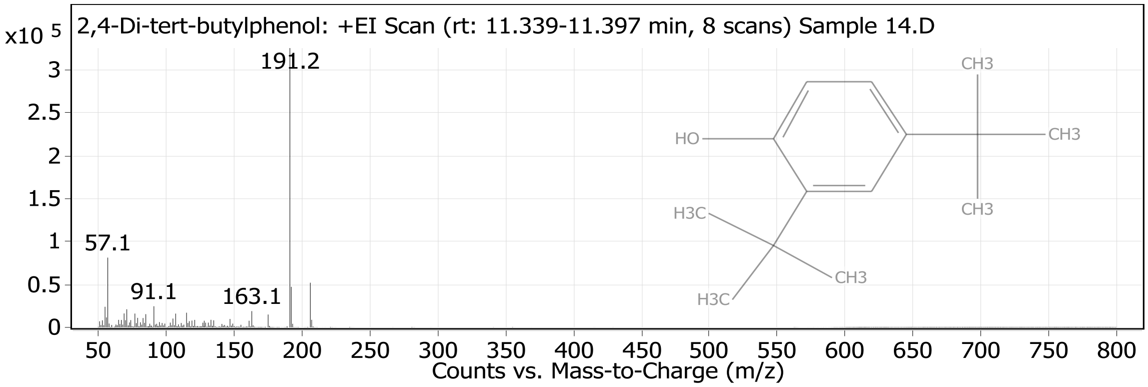

Library Spectrum

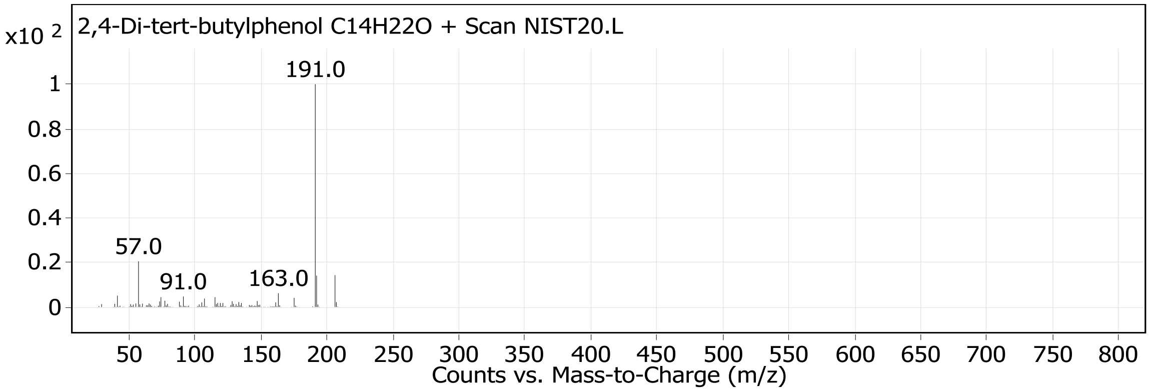

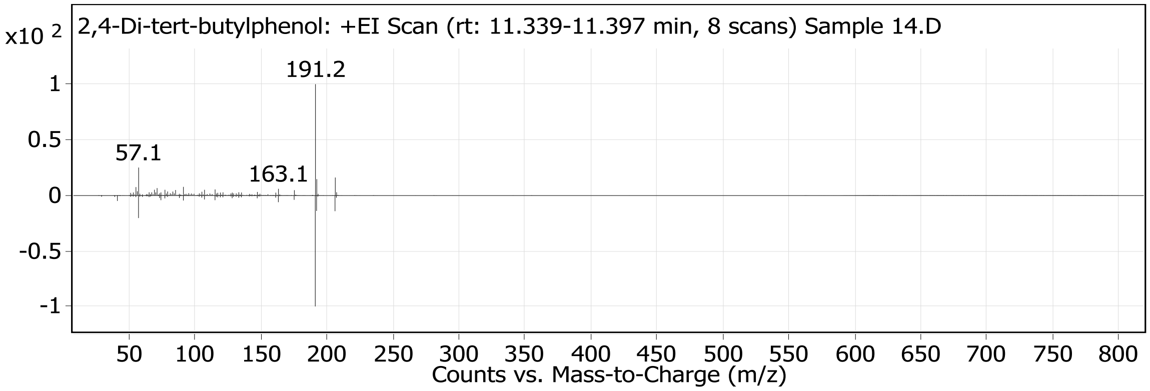

Spectrum Structure

2,4-Di-tert-butylphenol

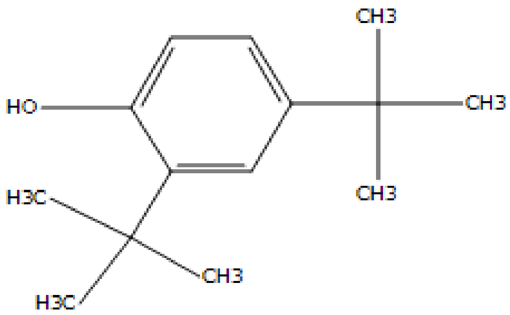

**Spectrum Source**	**Collision Energy**	**Ionization Mode**
Peak (4) in "+ TIC Scan"	0	EI

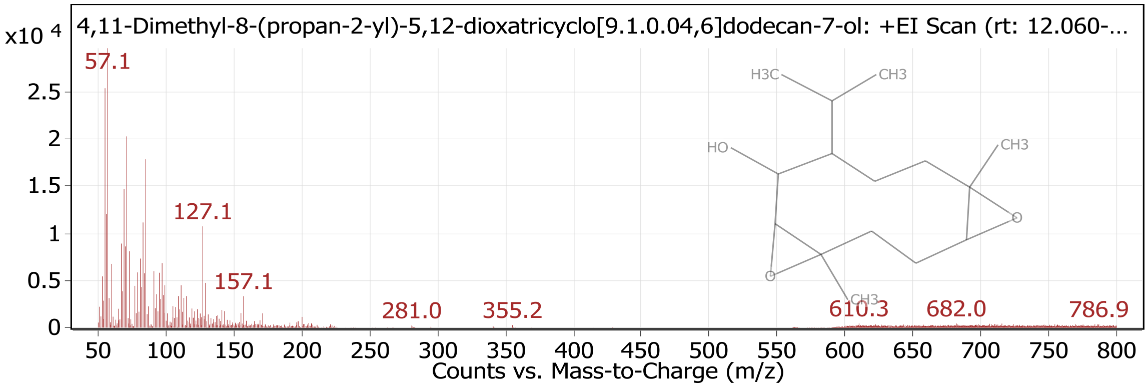

Library Spectrum

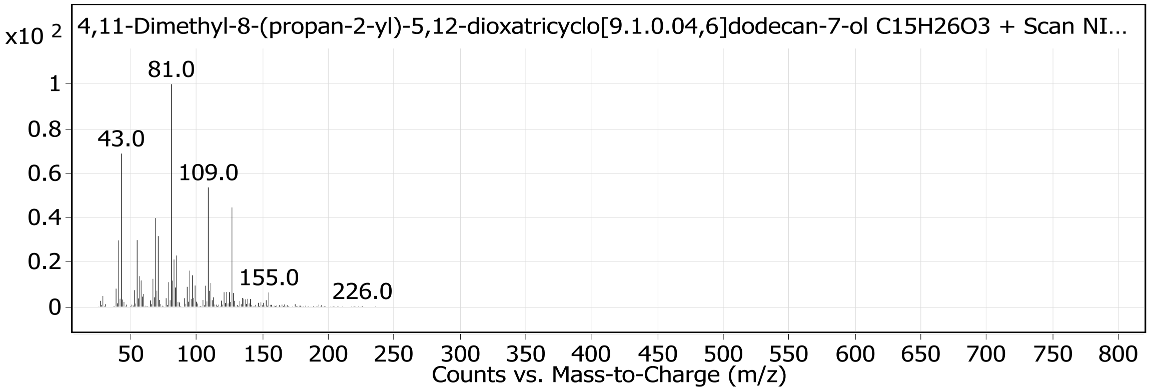

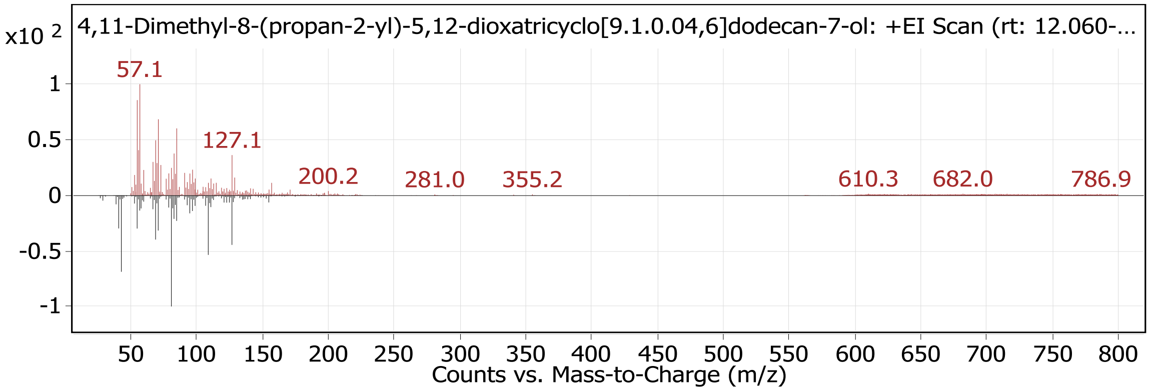

Spectrum Structure

4,11-Dimethyl-8-(propan-2-yl)-5,12-dioxatricyclo[9.1.0.04,6]dodecan-7-ol

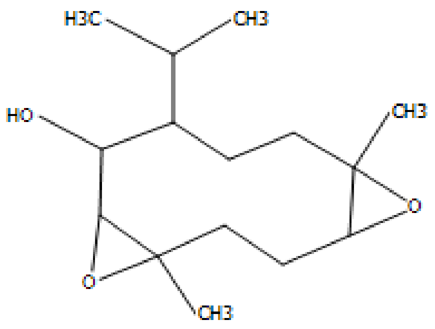

Spectrum Source	Collision Energy	Ionization Mode
Peak (5) in "+ TIC Scan"	0	EI

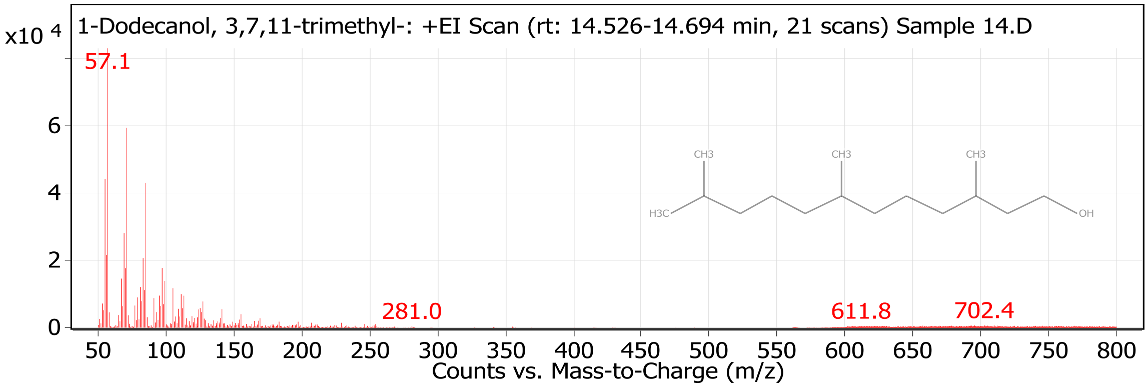

Library Spectrum

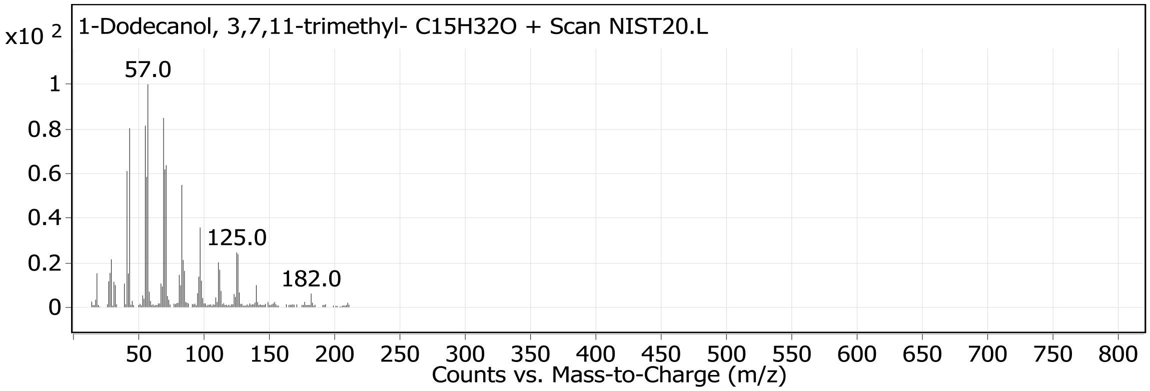

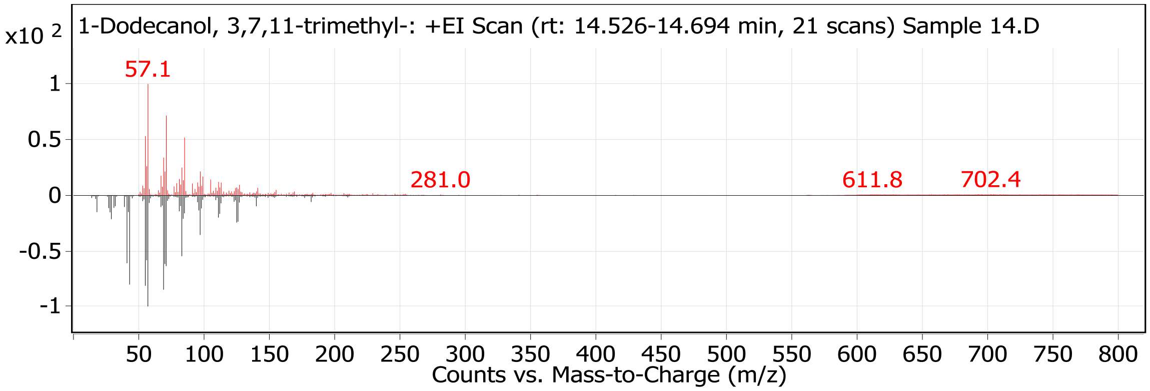

Spectrum Structure

1-Dodecanol, 3,7,11-trimethyl-

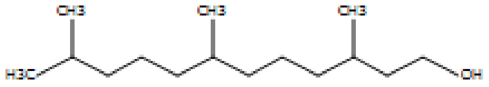

Qualitative Analysis Report

Spectrum Source	Collision Energy	Ionization Mode
Peak (6) in "+ TIC Scan"	0	EI

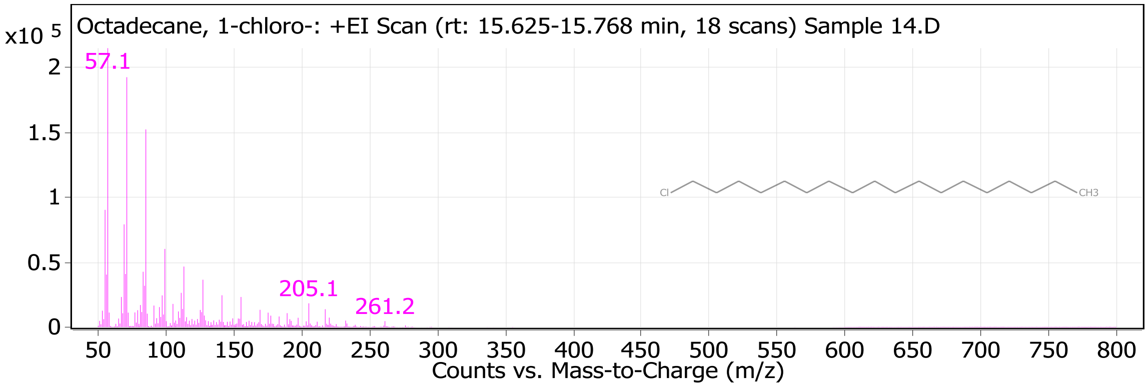

Library Spectrum

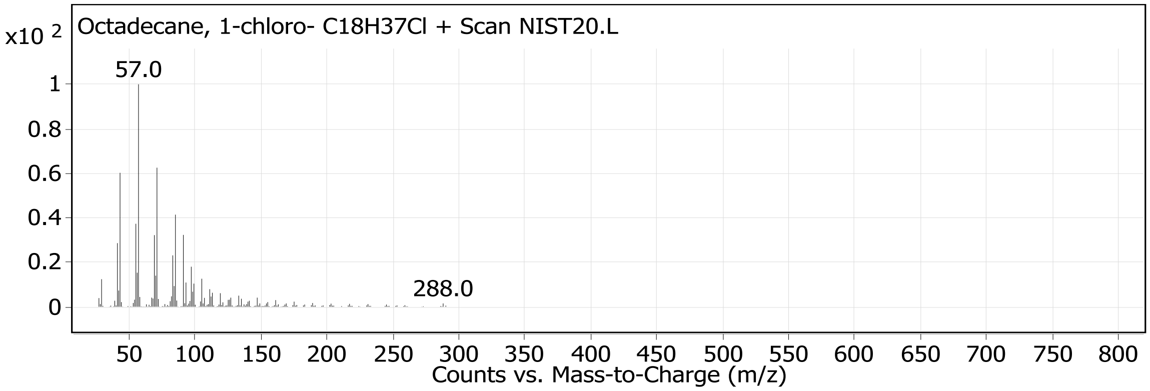

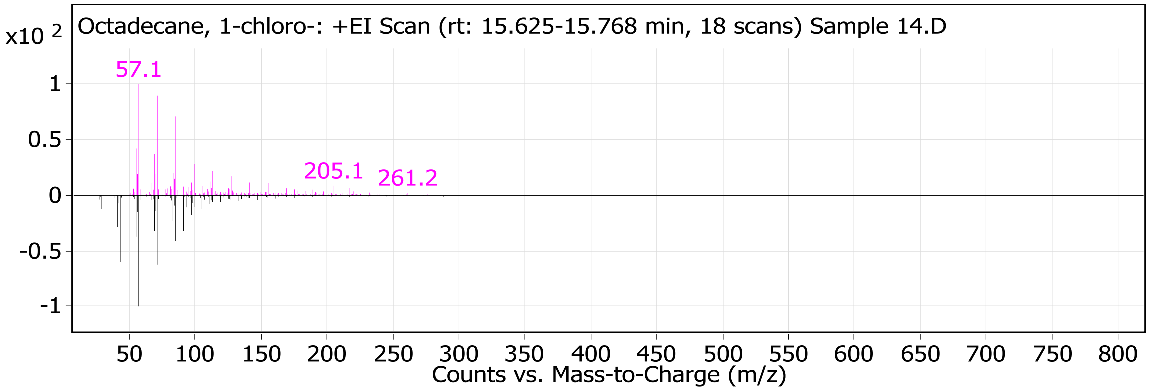

Spectrum Structure

Octadecane, 1-chloro-

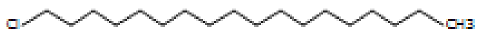

Spectrum Source	Collision Energy	Ionization Mode
Peak (7) in "+ TIC Scan"	0	EI

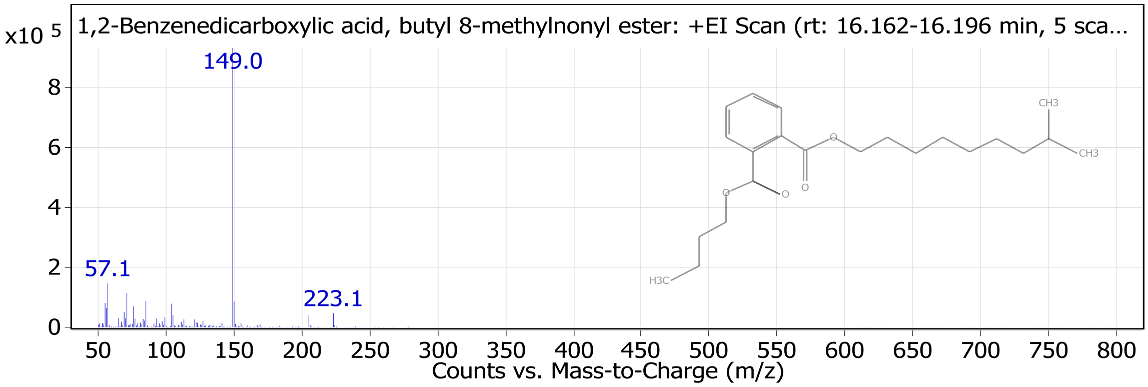

Library Spectrum

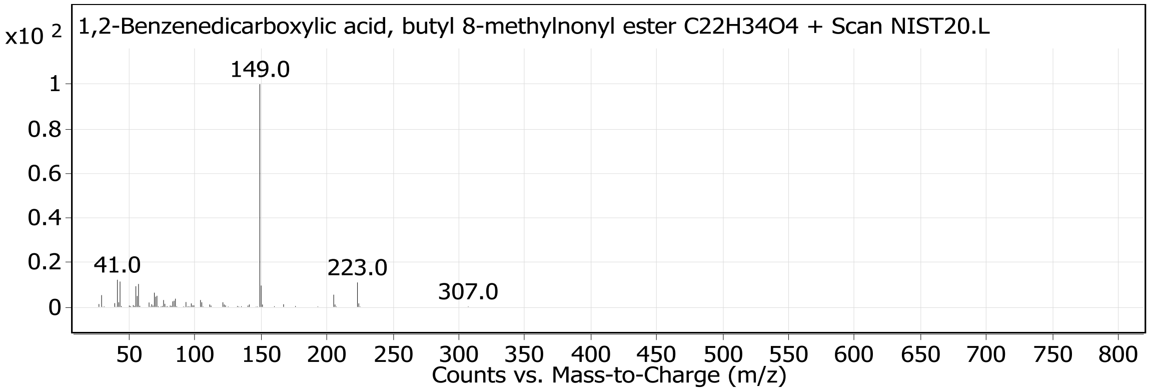

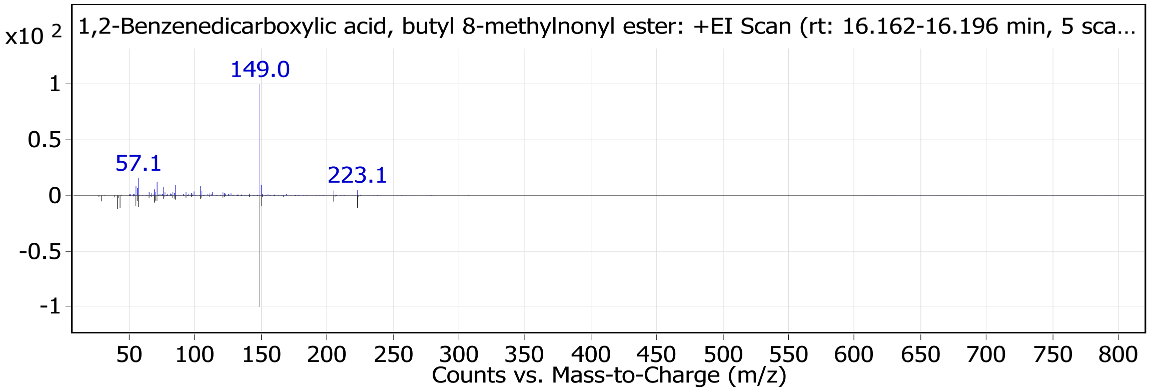

Spectrum Structure

1,2-Benzenedicarboxylic acid, butyl 8-methylnonyl ester

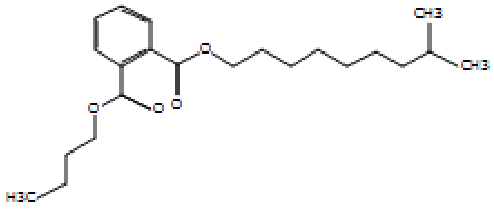

**Spectrum Source**	**Collision Energy**	**Ionization Mode**
Peak (8) in "+ TIC Scan"	0	EI

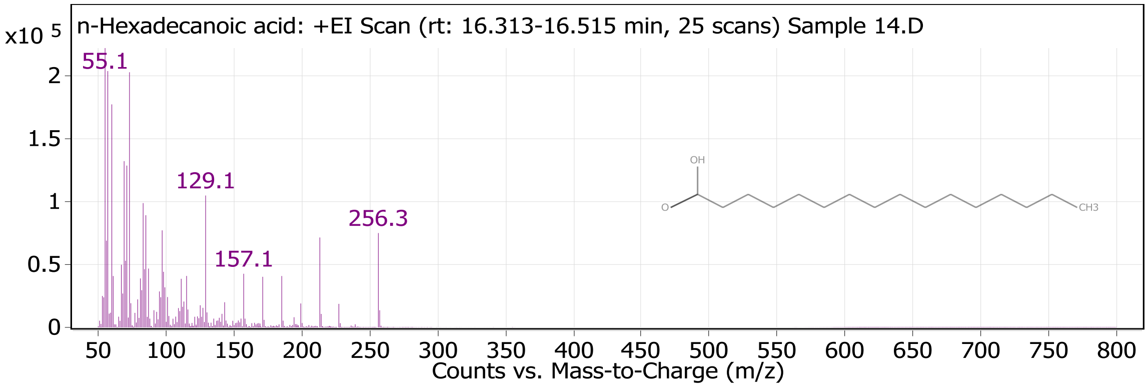

Library Spectrum

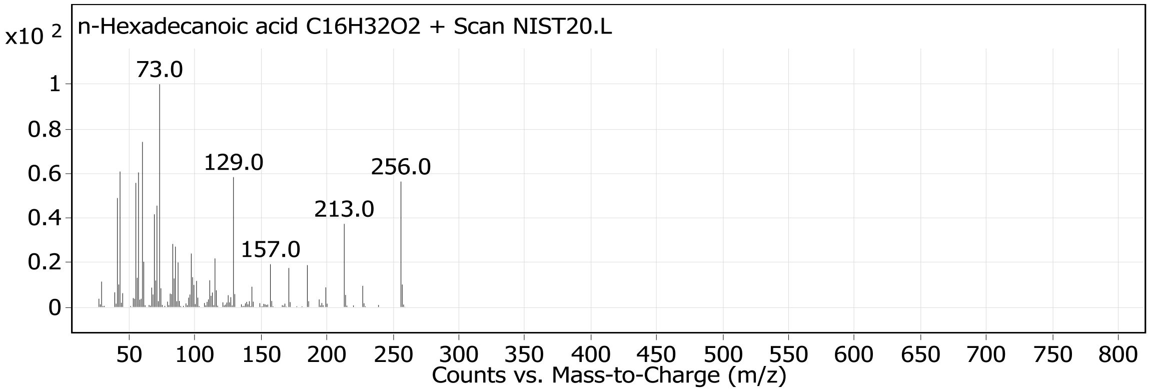

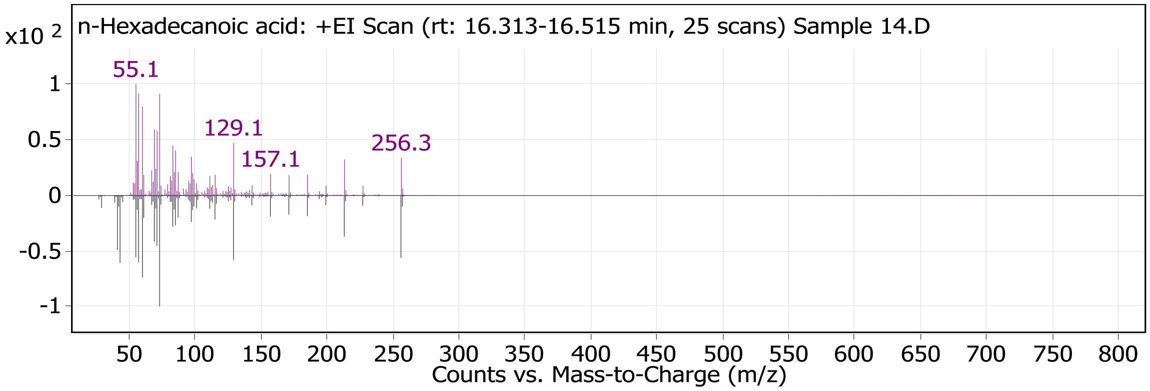

Spectrum Structure

n-Hexadecanoic acid

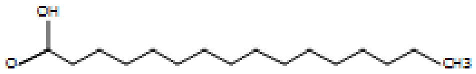

**Spectrum Source**	**Collision Energy**	**Ionization Mode**
Peak (9) in "+ TIC Scan"	0	EI

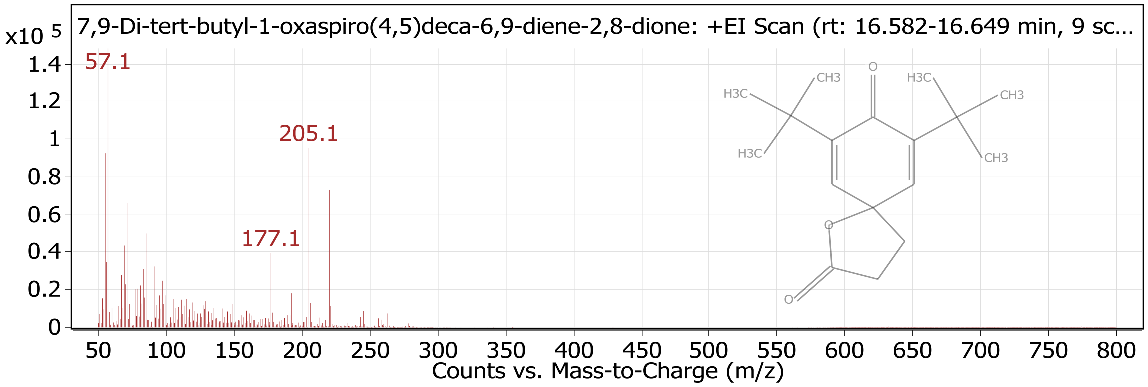

Library Spectrum

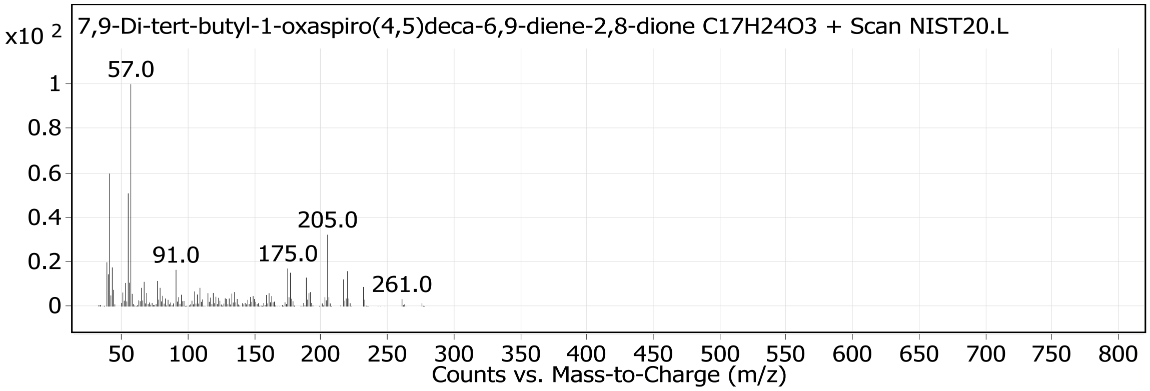
x10 2
7,9-Di-tert-butyl-1-oxaspiro(4,5)deca-6,9-diene-2,8-dione C17H24O3 + Scan NIST20.L
57.0
205.0
91.0
175.0
261.0
Counts vs. Mass-to-Charge (m/z)

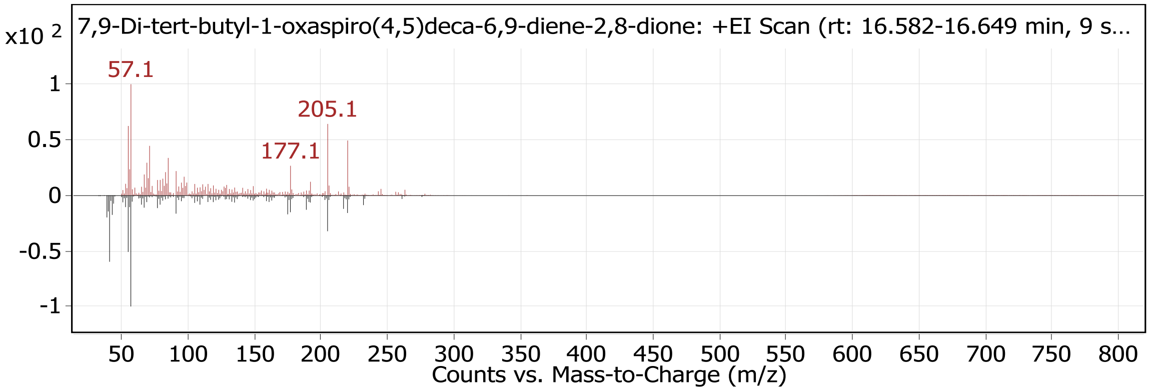
x10 2
7,9-Di-tert-butyl-1-oxaspiro(4,5)deca-6,9-diene-2,8-dione: +EI Scan (rt: 16.582-16.649 min, 9 s...
57.1
205.1
177.1
Counts vs. Mass-to-Charge (m/z)

Spectrum Structure

7,9-Di-tert-butyl-1-oxaspiro(4,5)deca-6,9-diene-2,8-dione

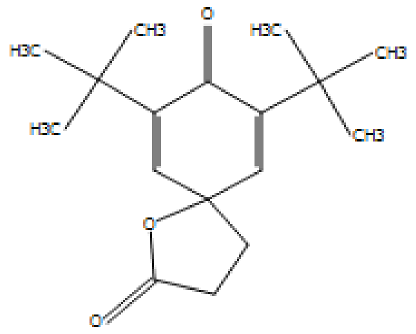

Spectrum Source	Collision Energy	Ionization Mode
Peak (10) in "+ TIC Scan"	0	EI

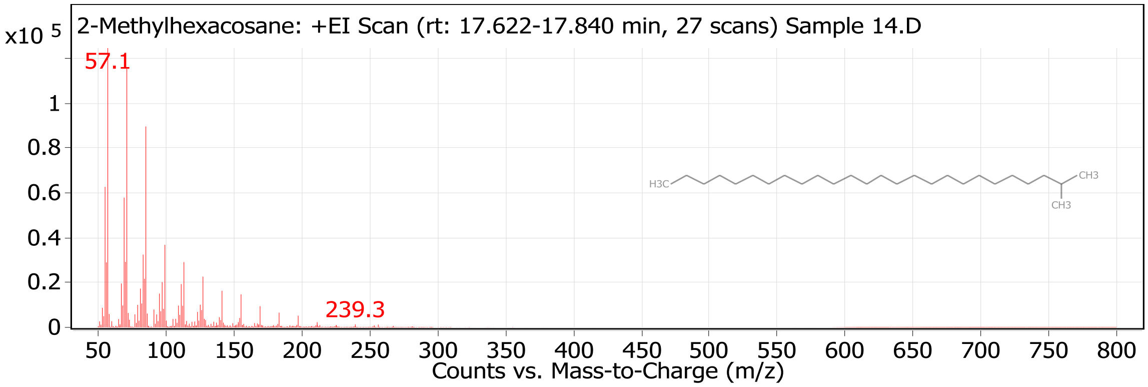

Library Spectrum

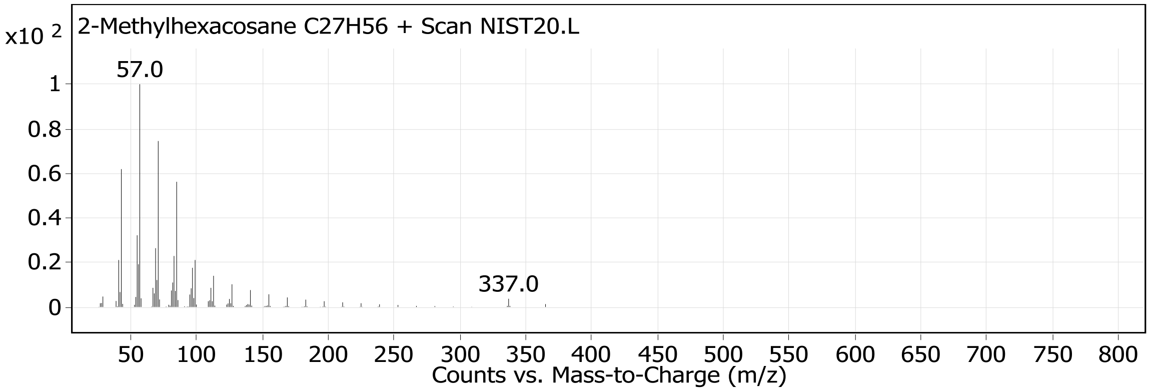

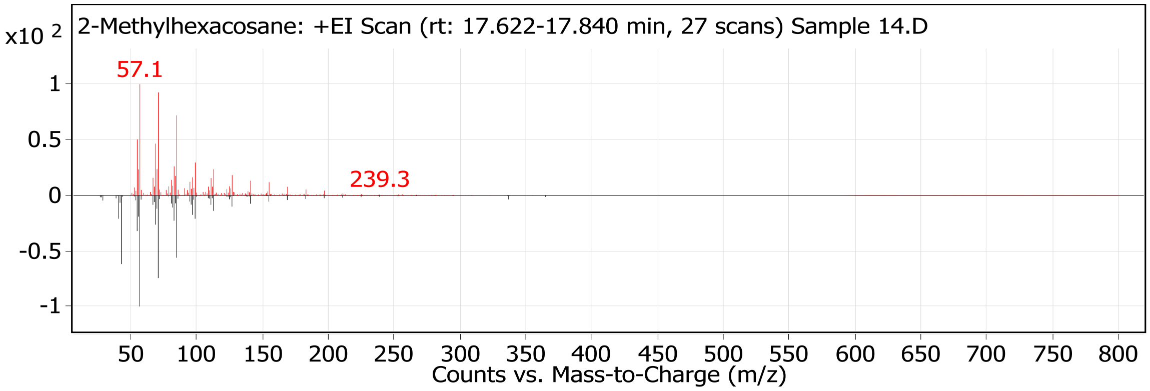

Spectrum Structure

2-Methylhexacosane

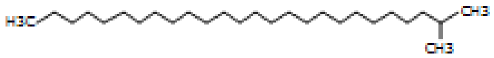

**Spectrum Source**	**Collision Energy**	**Ionization Mode**
Peak (11) in "+ TIC Scan"	0	EI

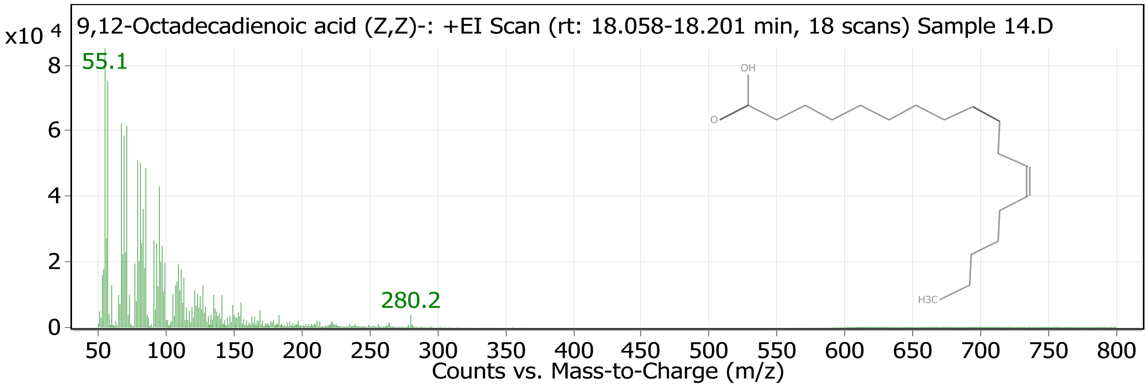

Library Spectrum

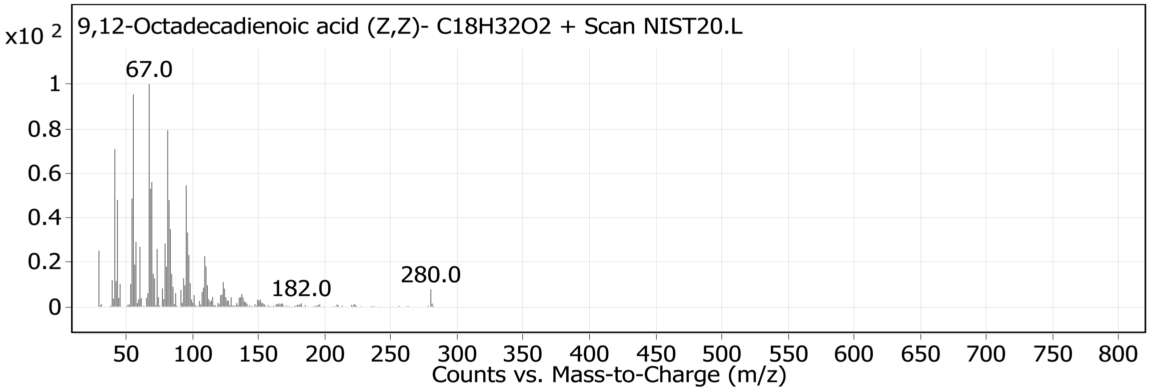

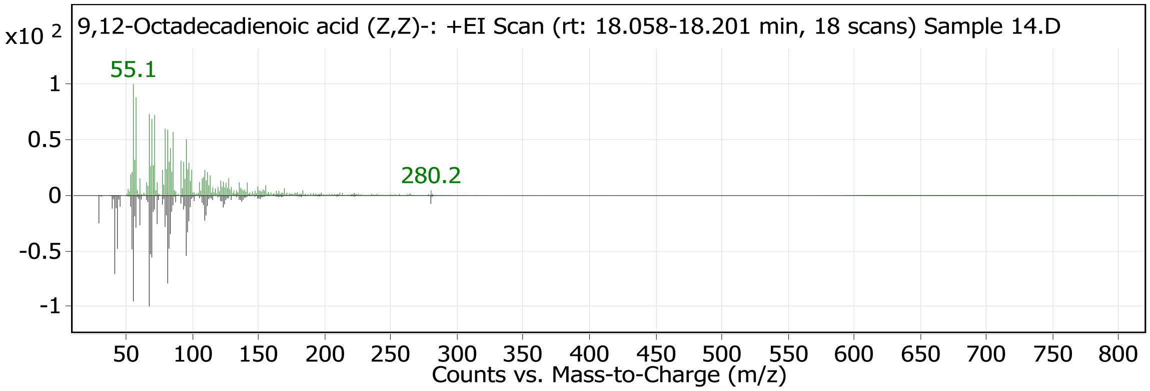

Spectrum Structure

9,12-Octadecadienoic acid (Z,Z)-

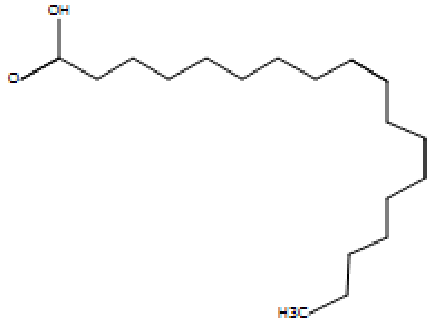

Spectrum Source	Collision Energy	Ionization Mode
Peak (12) in "+ TIC Scan"	0	EI

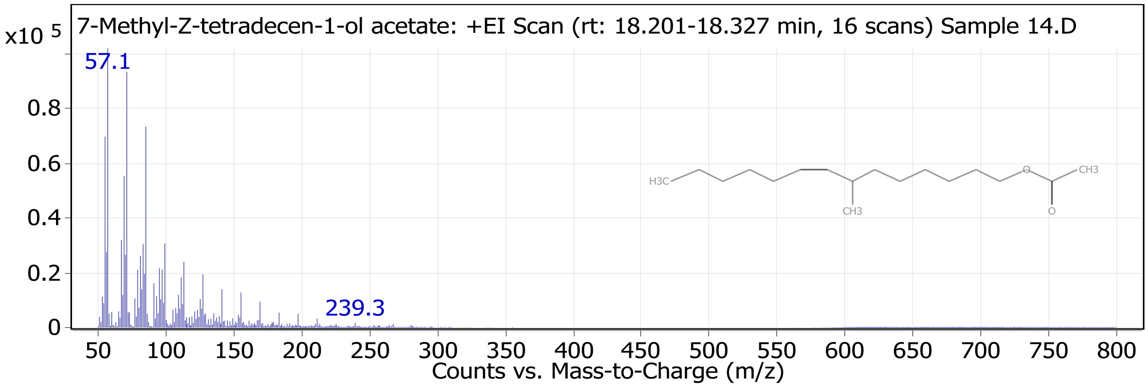

Library Spectrum

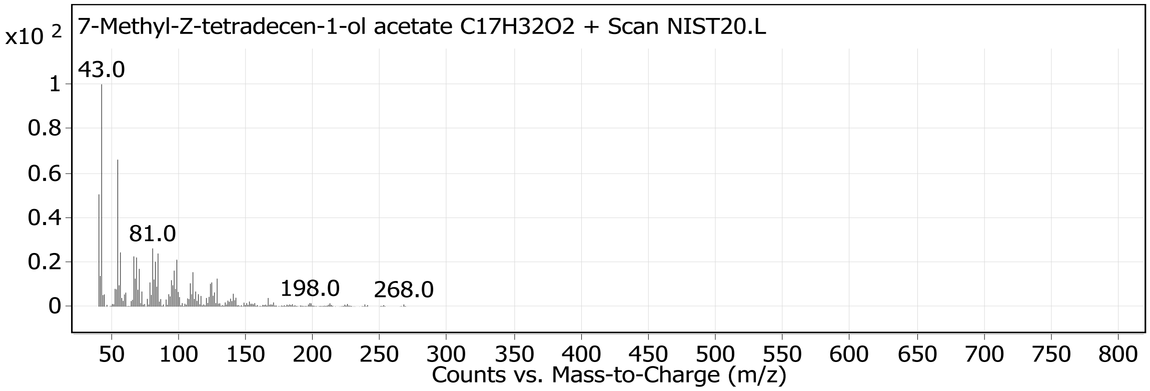

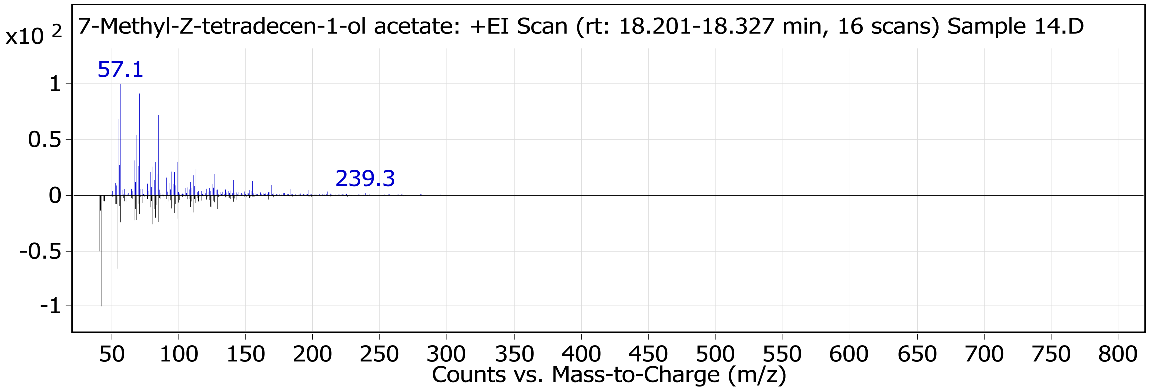

Spectrum Structure

7-Methyl-Z-tetradecen-1-ol acetate

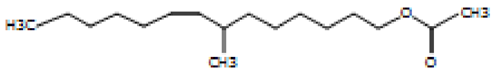

Spectrum Source	Collision Energy	Ionization Mode
Peak (13) in "+ TIC Scan"	0	EI

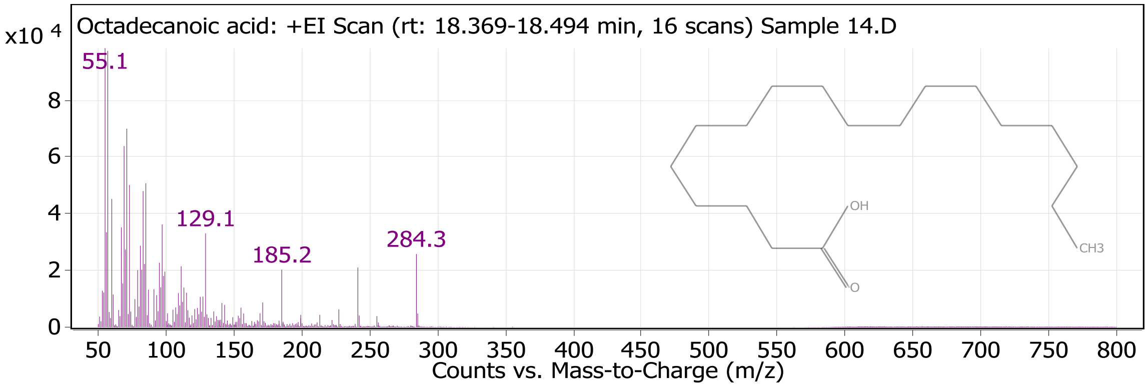

Library Spectrum

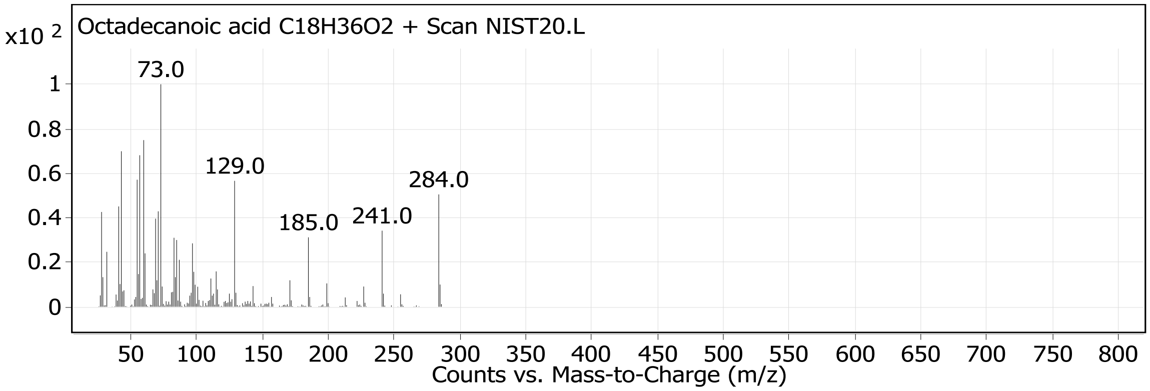

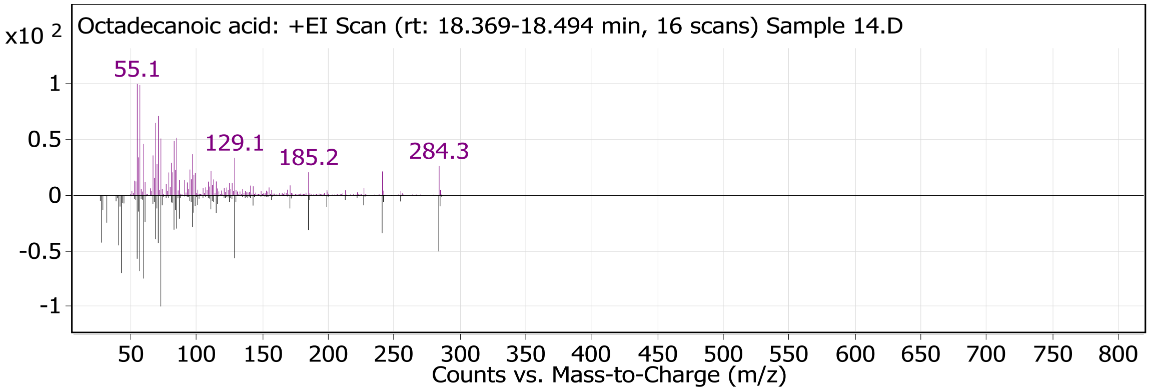

Spectrum Structure

Octadecanoic acid

Qualitative Analysis Report

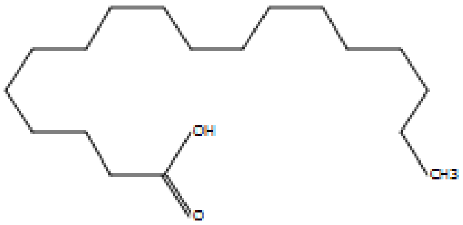

Spectrum Source	Collision Energy	Ionization Mode
Peak (14) in "+ TIC Scan"	0	EI

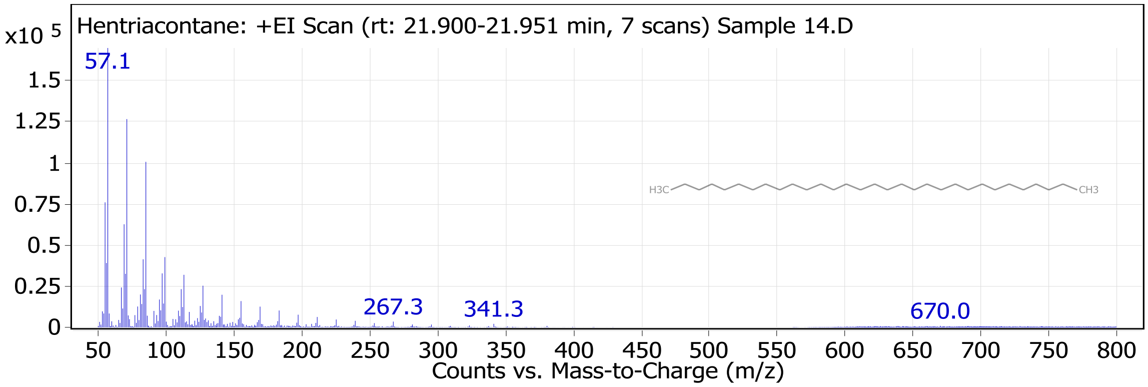

Library Spectrum

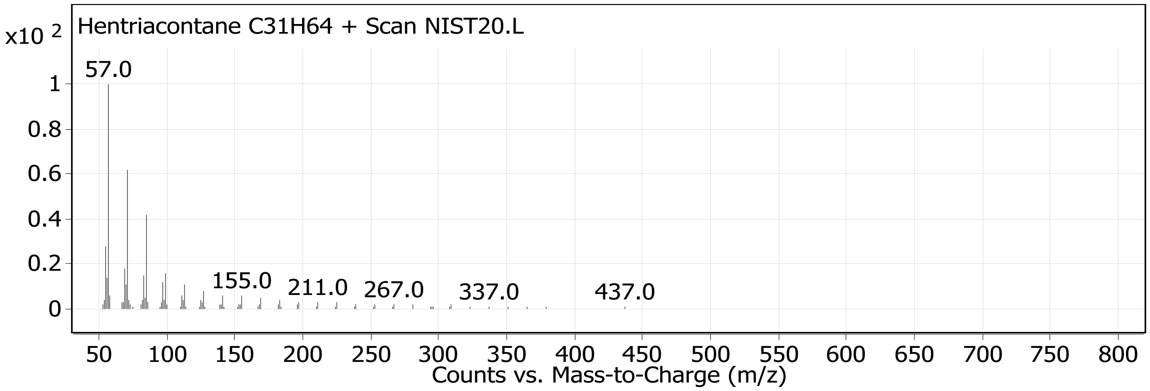

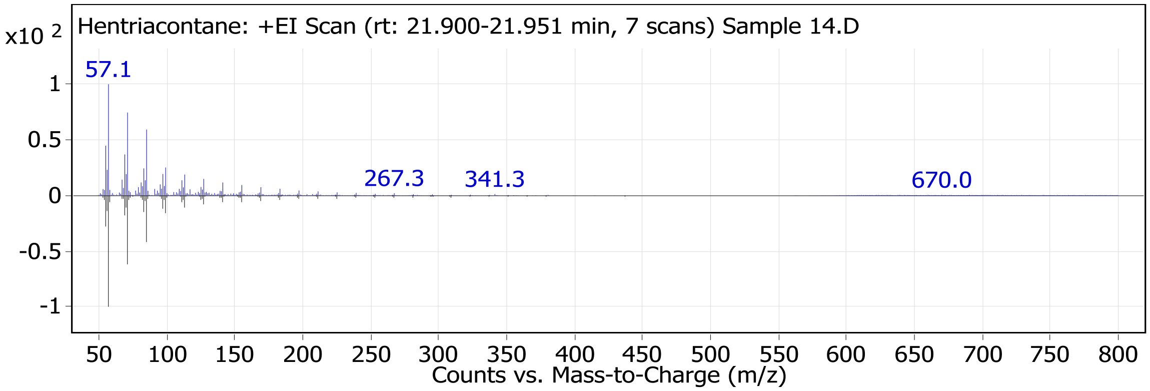

Qualitative Analysis Report

Spectrum Structure

Hentriacontane

Spectrum Source	Collision Energy	Ionization Mode
Peak (15) in "+ TIC Scan"	0	EI

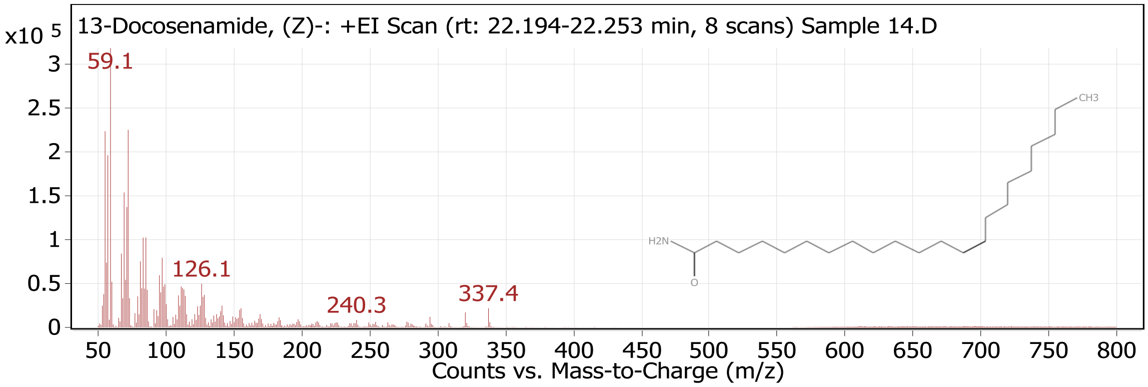

Library Spectrum

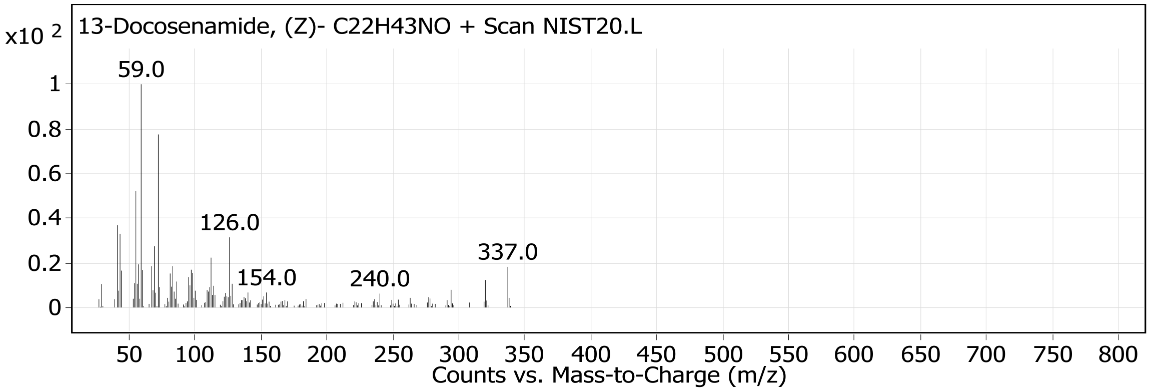

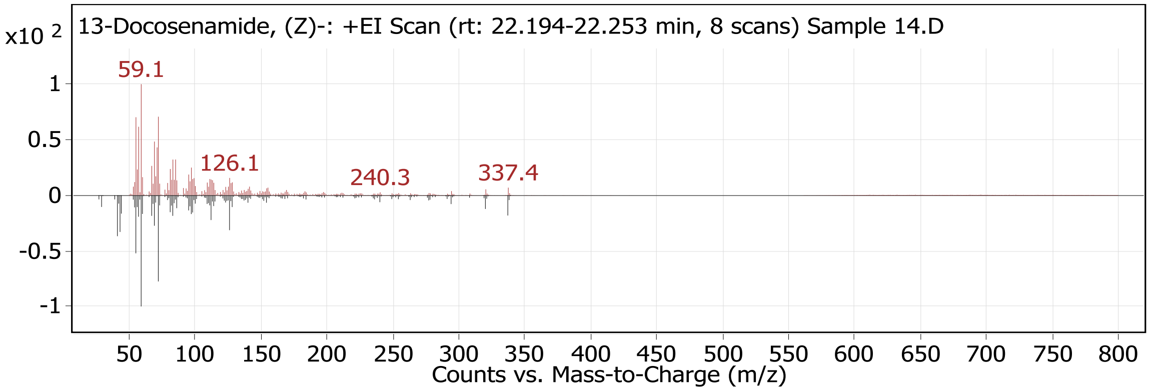

Spectrum Structure

13-Docosenamide, (Z)-

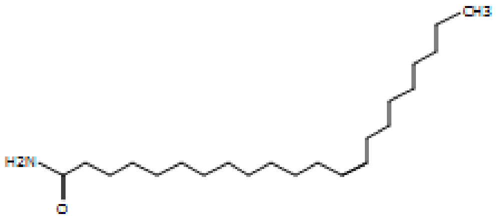

**Spectrum Source**	**Collision Energy**	**Ionization Mode**
Peak (16) in "+ TIC Scan"	0	EI

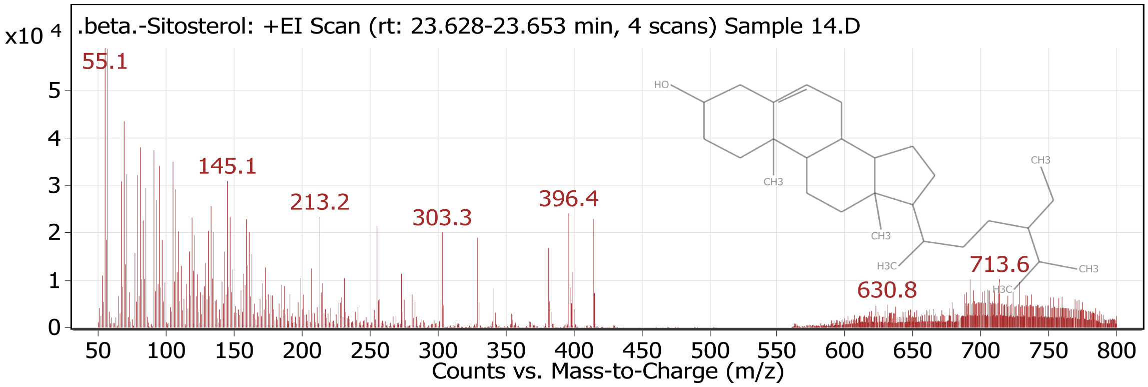

Library Spectrum

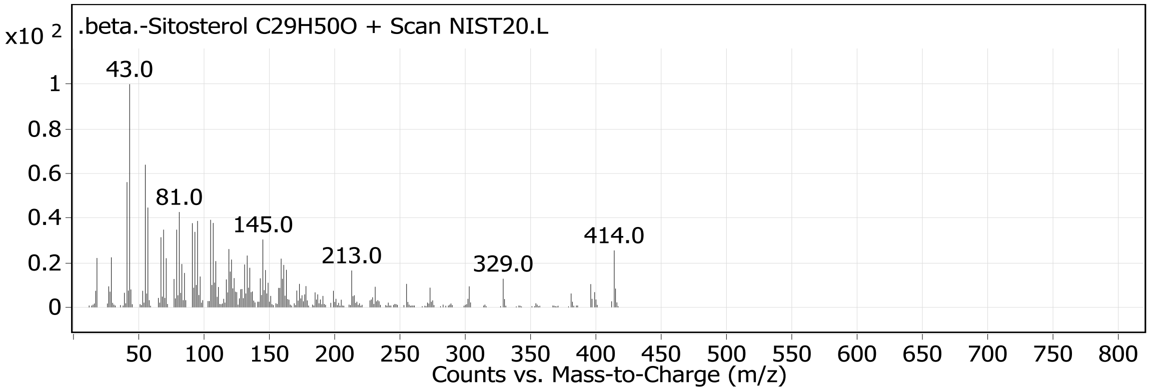

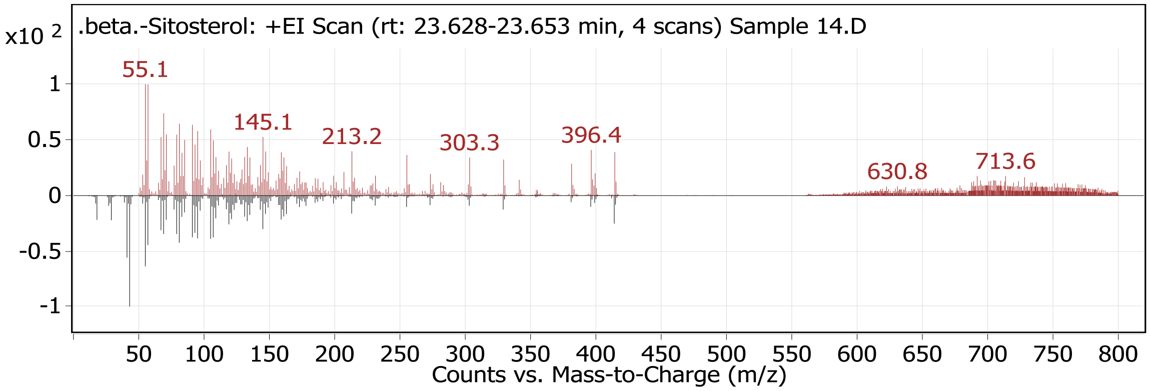

Spectrum Structure

.beta.-Sitosterol

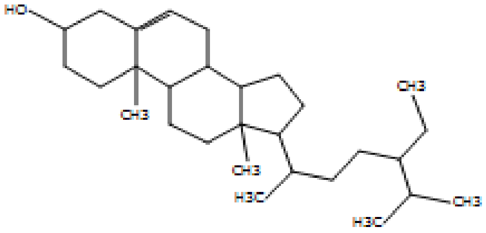

--- End Of Report ---
