# Supplementary material for: Chemical and Molecular Insights into the Arid Wild Plant Diversity of Saudi Arabia
Source: Plants (Basel). 2026 Jan 19;15(2):295. doi: 10.3390/plants15020295 (PMC12845481; doi:10.3390/plants15020295)
Supplement: Supplementary file 1 [file plants-15-00295-s001.zip › Sample 8_AnalysisReport.pdf]

Qualitative Analysis Report

**Data Filename**	Sample 15.D	**Sample Name**	Sample 15
**Sample Type**		**Position**	1
**Instrument Name**	3	**User Name**	
**Acq Method**	Scan DB-5MS Hydrogen 2024.M	**Acquired Time**	6/25/2024 5:38:57 PM
**IRM Calibration Status**	Not Applicable	**DA Method**	SignalToNoiseCheckout.m
**Comment**			

**Expected Barcode**		**Sample Amount**	
**Dual Inj Vol**	0.2	**TuneName**	ATUNE.U
**TunePath**	D:\MassHunter\GCMS\3\5977 \	**TuneDateStamp**	2024-06-23T14:01:57+02:00
**MSFirmwareVersion**	6.00.34	**OperatorName**	
**RunCompletedFlag**	True	**Acquisition SW Version**	MassHunter GC/MS Acquisition 10.0.368 14-Feb-2019 Copyright © 1989-2018 Agilent Technologies, Inc

User Chromatograms

Fragmentor Voltage **Collision Energy** 0 **Ionization Mode** EI

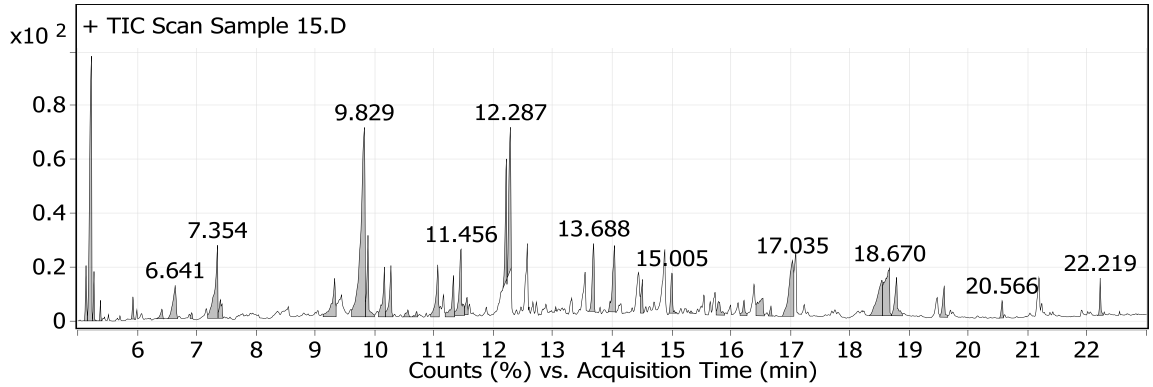

Integration Peak List

Peak	Start	RT	End	Height	Area	Area %
1	5.106	5.139	5.165	15272664.02	20033004.19	7.78
2	5.165	5.232	5.248	73149228.06	171941224.9	66.81
3	5.248	5.274	5.324	13643984.59	13292376.18	5.16
4	5.358	5.383	5.408	5677808.12	5156375.58	2
5	5.898	5.928	5.952	6116499.98	7910713.17	3.07
6	6.314	6.415	6.445	2640436.33	6935509.25	2.69
7	6.515	6.641	6.683	9192205.41	34851097.42	13.54
8	6.893	6.918	6.937	1575425.4	2687801.94	1.04
9	7.186	7.354	7.362	20183099.41	68809945.28	26.74
10	7.362	7.404	7.446	5088512.32	15143991.29	5.88
11	9.132	9.325	9.351	10616777.48	41198966.21	16.01
12	9.611	9.829	9.846	52314249.86	257376869.2	100
13	9.846	9.887	9.997	22506639.88	44582147.67	17.32
14	10.064	10.164	10.181	13780464.9	33848608.25	13.15
15	10.181	10.273	10.307	14181084.2	33556035.7	13.04
16	10.483	10.559	10.609	1914334.98	5785530.48	2.25
17	10.626	10.71	10.726	1467560	3255090.47	1.26
18	10.944	11.062	11.087	14366108	40105546.16	15.58
19	11.188	11.33	11.347	11455728	30468947.64	11.84

20	11.347	11.456	11.515	18320381.94	54280547.11	21.09
21	11.515	11.557	11.574	4746583	11160815.89	4.34
22	12.16	12.22	12.228	33385848.73	45349818.04	17.62
23	12.233	12.287	12.302	39420661.52	84598637.45	32.87
24	13.035	13.05	13.064	1068219.55	770127.41	0.3
25	13.604	13.688	13.711	18768510.68	42204935.02	16.4
26	13.934	14.04	14.057	18330797.85	49445601.75	19.21
27	14.476	14.51	14.526	9300578.78	16557072.98	6.43
28	14.957	15.005	15.093	11030481.63	24789522.82	9.63
29	15.307	15.323	15.362	602859.97	1032279.23	0.4
30	15.751	15.793	15.894	3759256.13	15285867.8	5.94
31	16.154	16.221	16.271	4153223.03	10602055.49	4.12
32	16.422	16.54	16.565	4853039.36	30325802.19	11.78
33	16.632	16.674	16.699	2671584.44	4797281.05	1.86
34	16.86	17.035	17.06	15405743.33	81468103.61	31.65
35	18.328	18.545	18.553	9758930.55	58106253.3	22.58
36	18.553	18.67	18.704	13069779.74	77528918.81	30.12
37	18.704	18.788	18.889	10620537.3	33436481.87	12.99
38	19.518	19.593	19.652	8662662.09	21751612.63	8.45
39	20.524	20.566	20.591	4746327.48	8328545.33	3.24
40	22.169	22.219	22.269	10286815.65	13561422.16	5.27

User Spectra

Spectrum Source	Collision Energy	Ionization Mode
Peak (1) in "+ TIC Scan"	0	EI

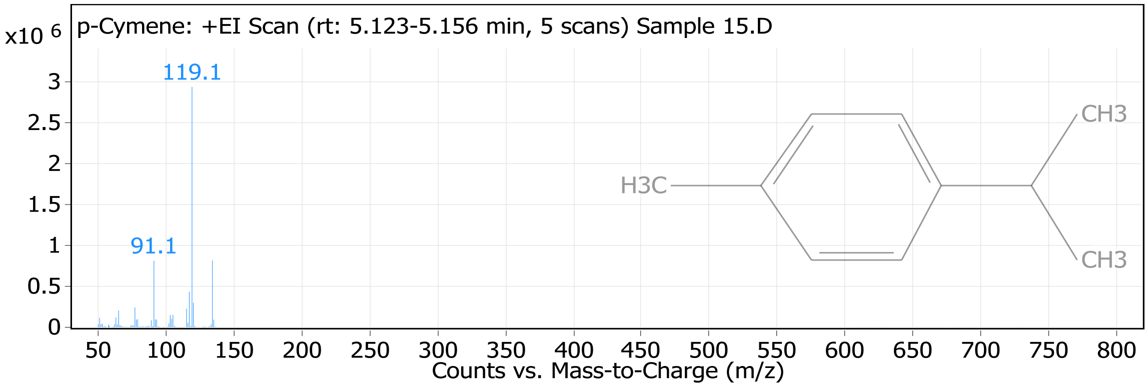

Library Spectrum

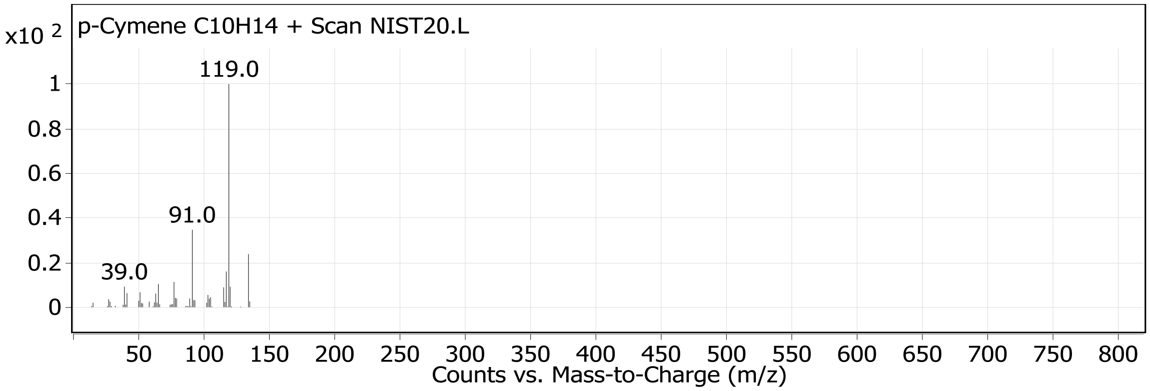

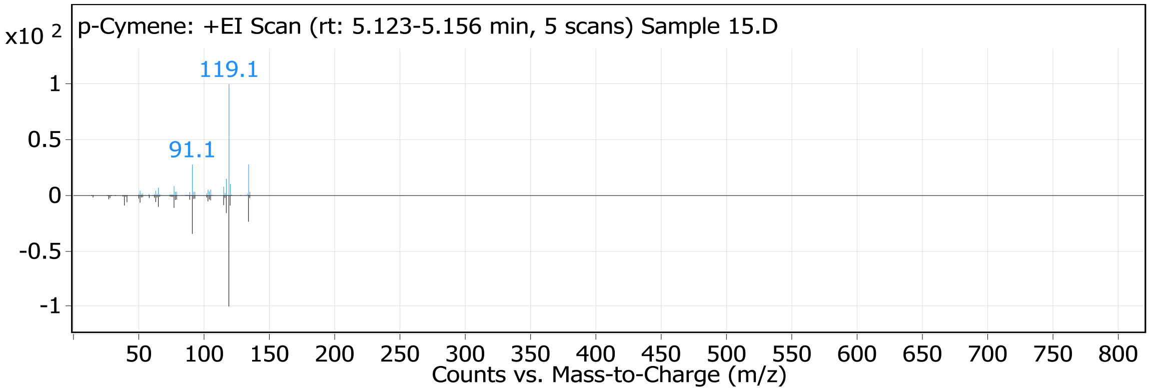

Spectrum Structure

p-Cymene

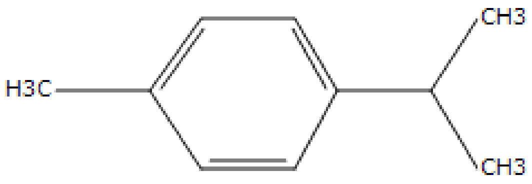

**Spectrum Source**	**Collision Energy**	**Ionization Mode**
Peak (2) in "+ TIC Scan"	0	EI

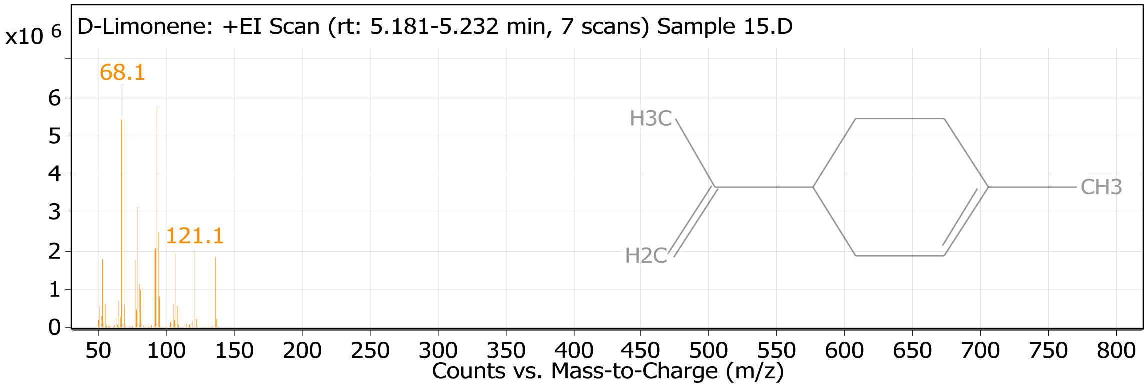

Library Spectrum

Qualitative Analysis Report

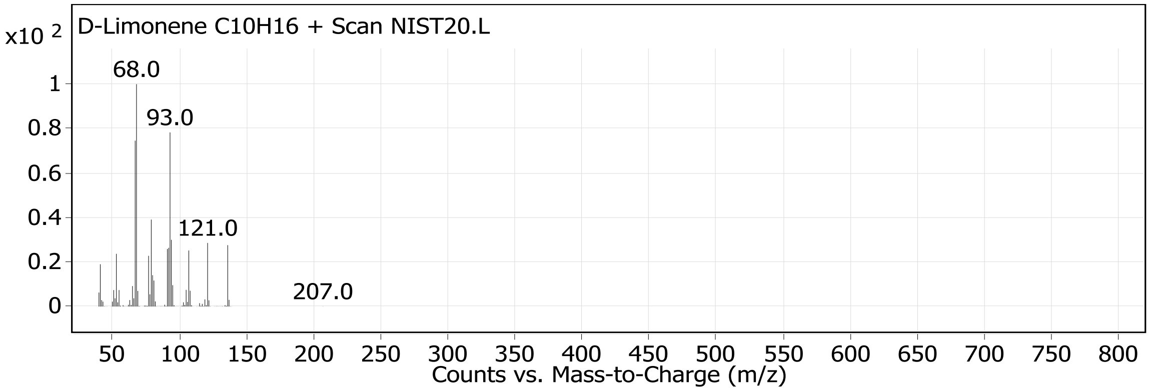

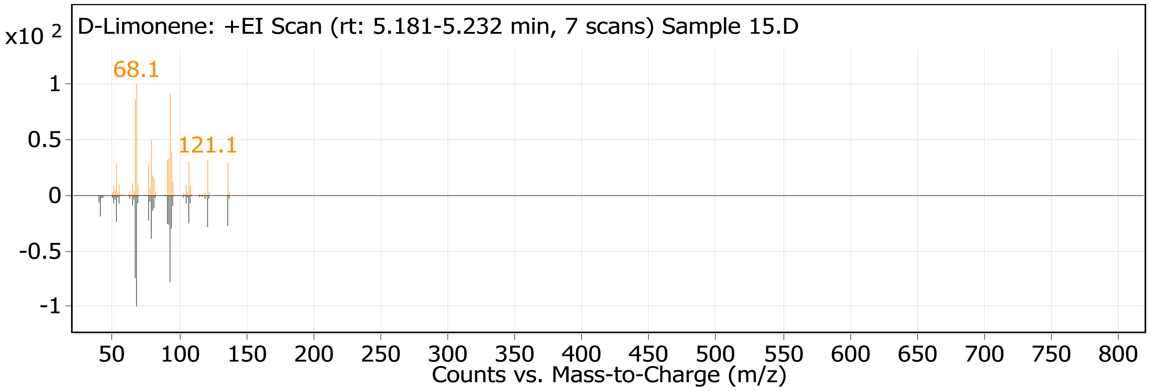

Spectrum Structure

D-Limonene

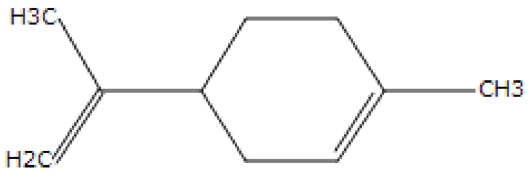

**Spectrum Source**	**Collision Energy**	**Ionization Mode**
Peak (3) in "+ TIC Scan"	0	EI

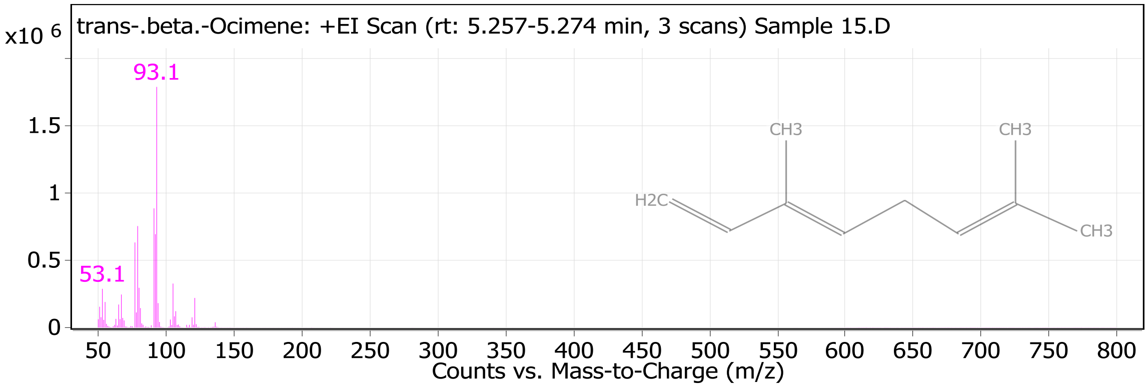

Library Spectrum

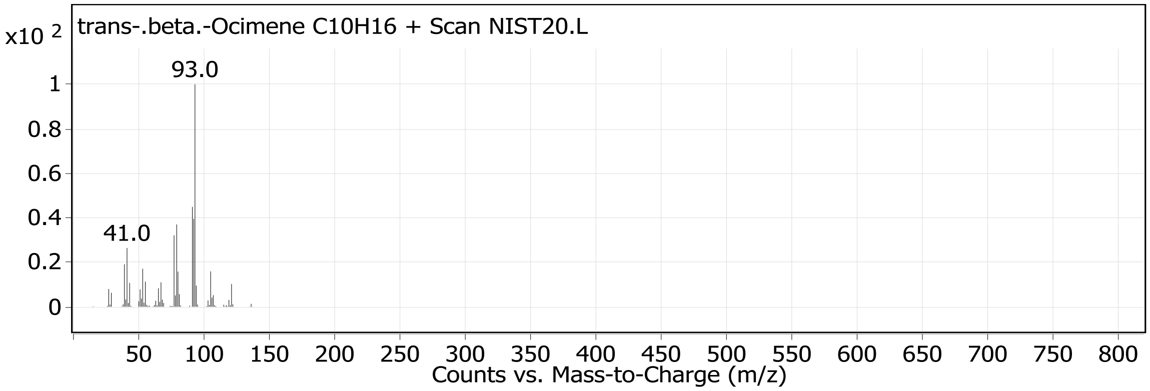

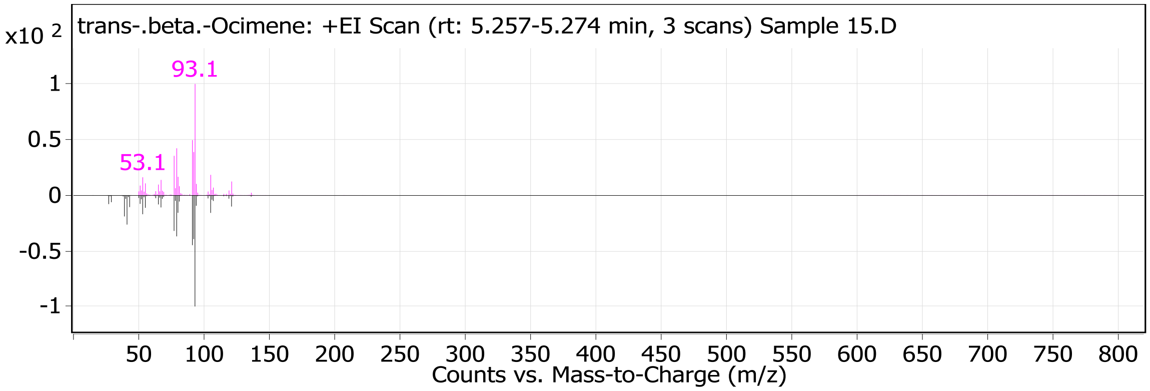

Spectrum Structure

trans-.beta.-Ocimene

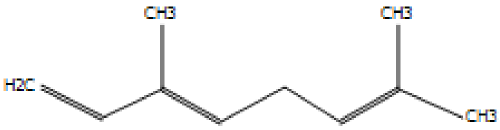

Qualitative Analysis Report

Spectrum Source	Collision Energy	Ionization Mode
Peak (4) in "+ TIC Scan"	0	EI

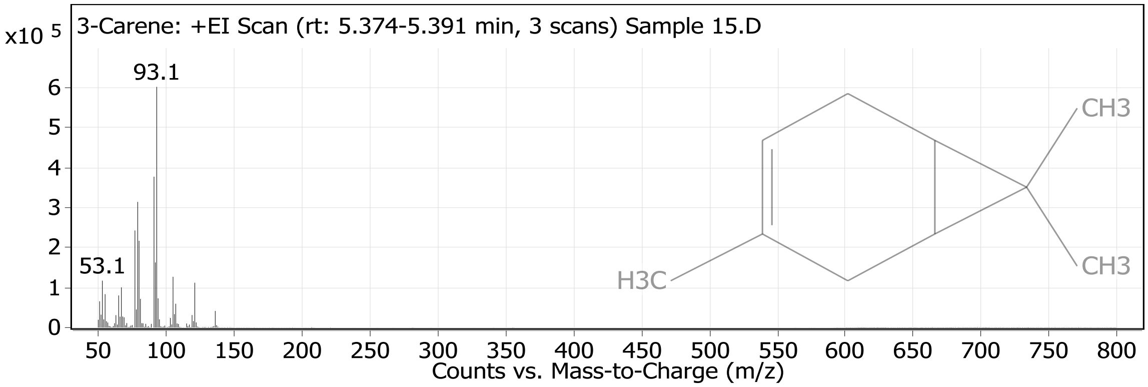

Library Spectrum

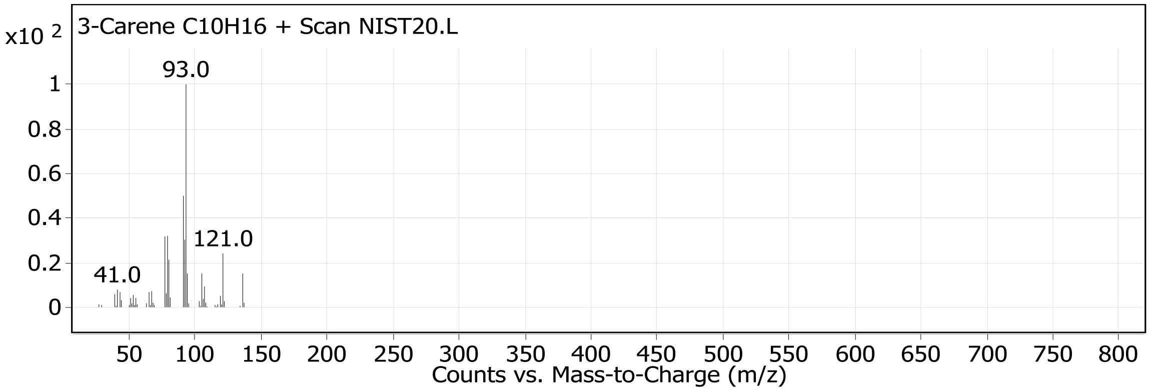

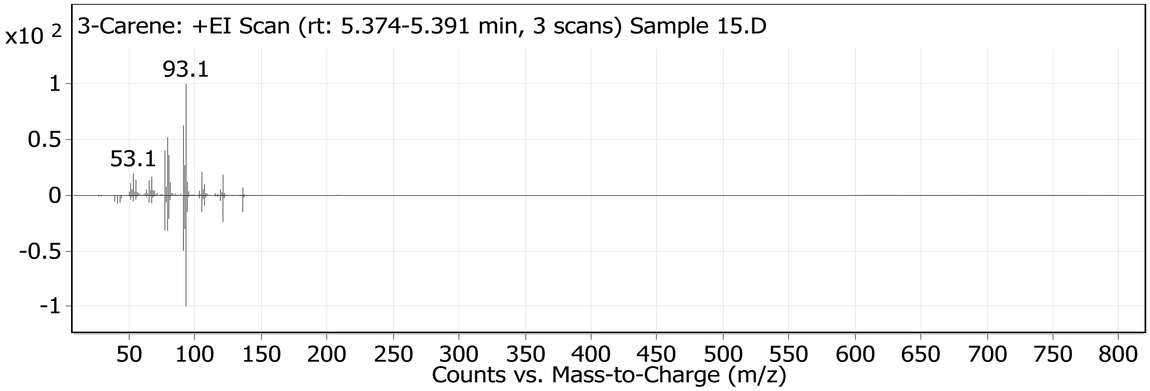

Spectrum Structure

3-Carene

Qualitative Analysis Report

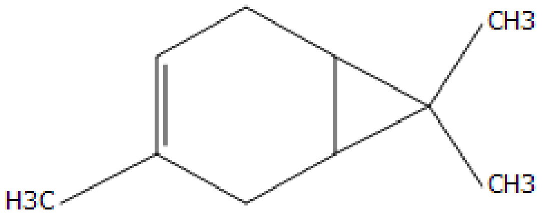

Spectrum Source	Collision Energy	Ionization Mode
Peak (5) in "+ TIC Scan"	0	EI

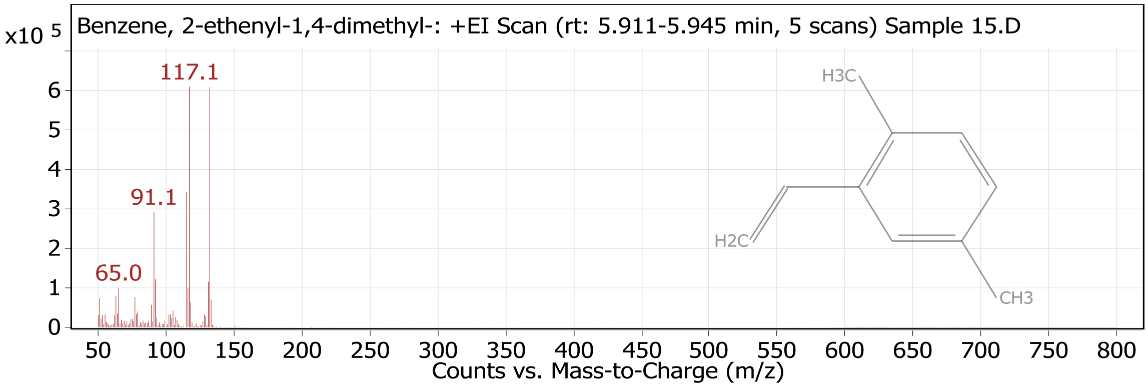

Library Spectrum

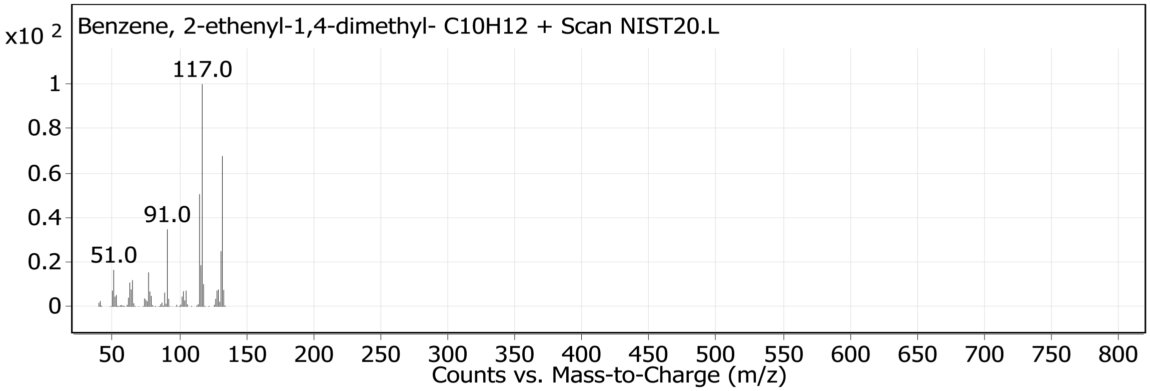

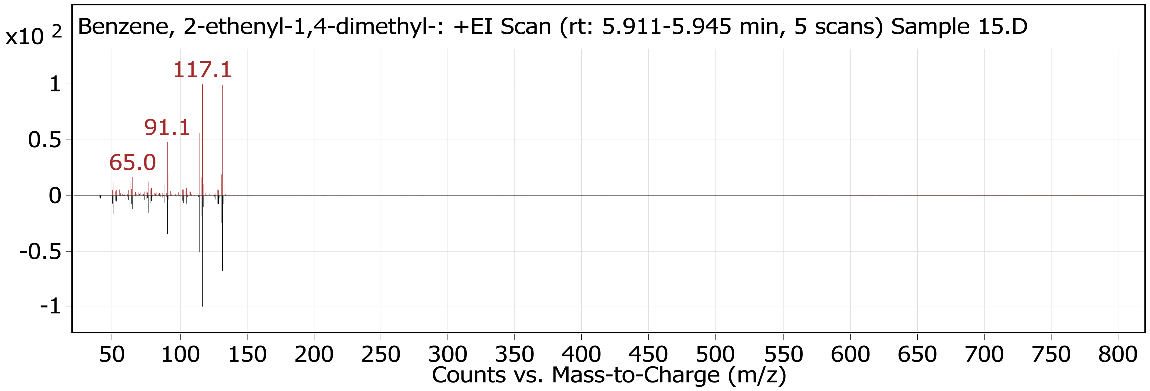

Spectrum Structure

Benzene, 2-ethenyl-1,4-dimethyl-

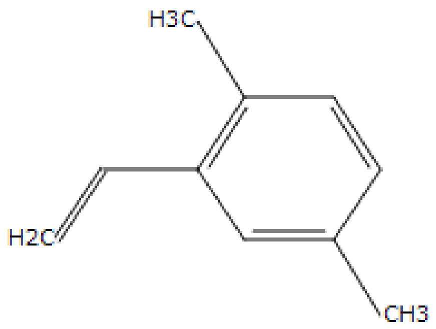

Spectrum Source	Collision Energy	Ionization Mode
Peak (6) in "+ TIC Scan"	0	EI

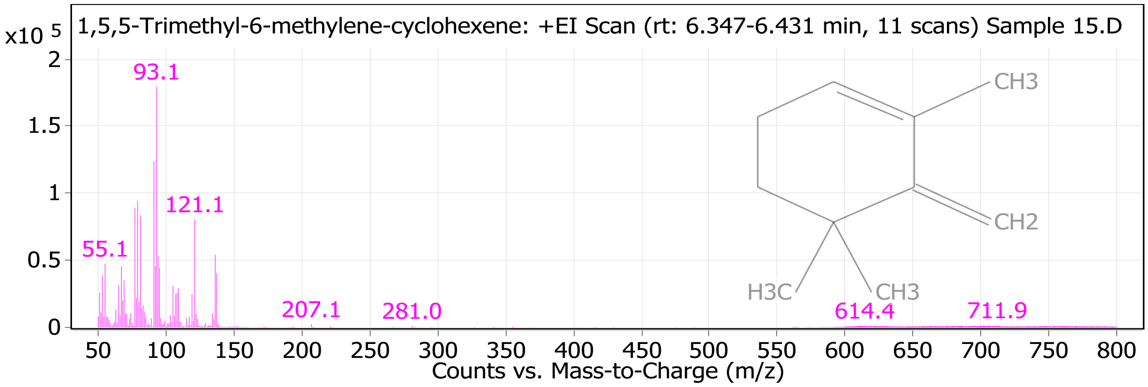

Library Spectrum

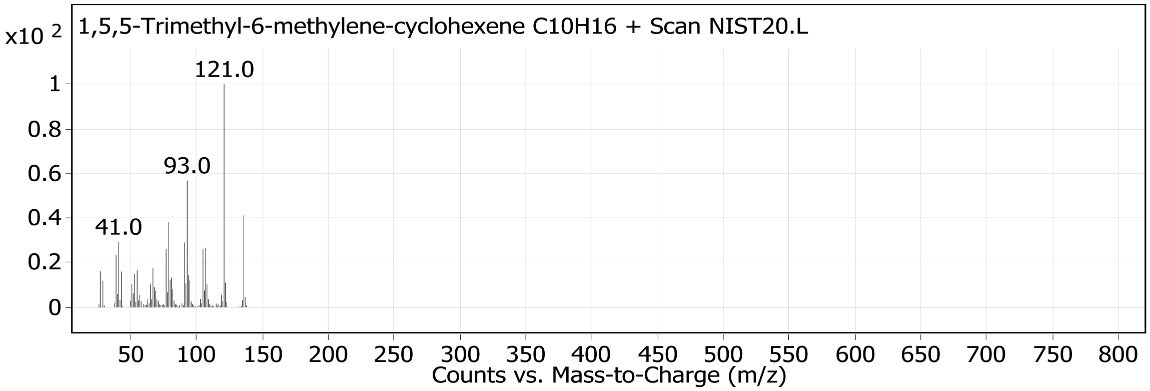

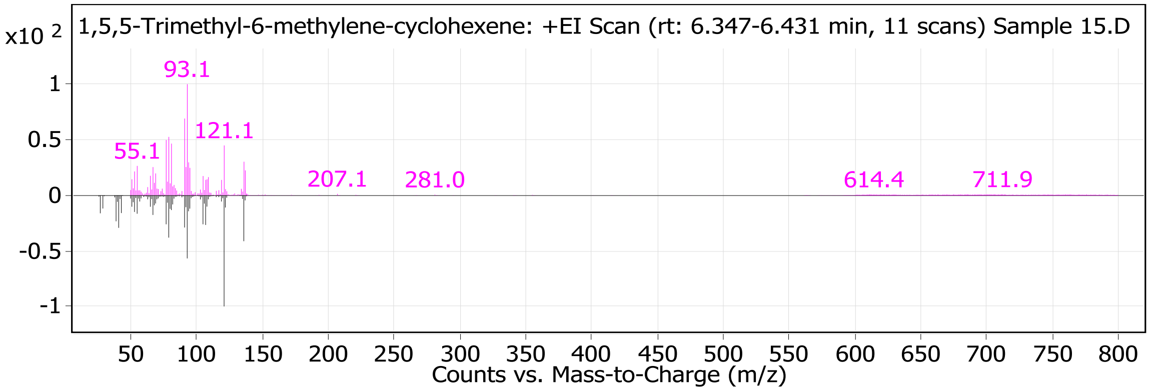

Spectrum Structure

1,5,5-Trimethyl-6-methylene-cyclohexene

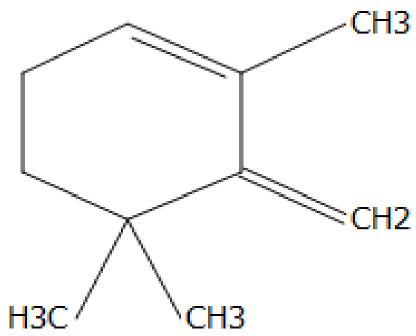

**Spectrum Source**	**Collision Energy**	**Ionization Mode**
Peak (7) in "+ TIC Scan"	0	EI

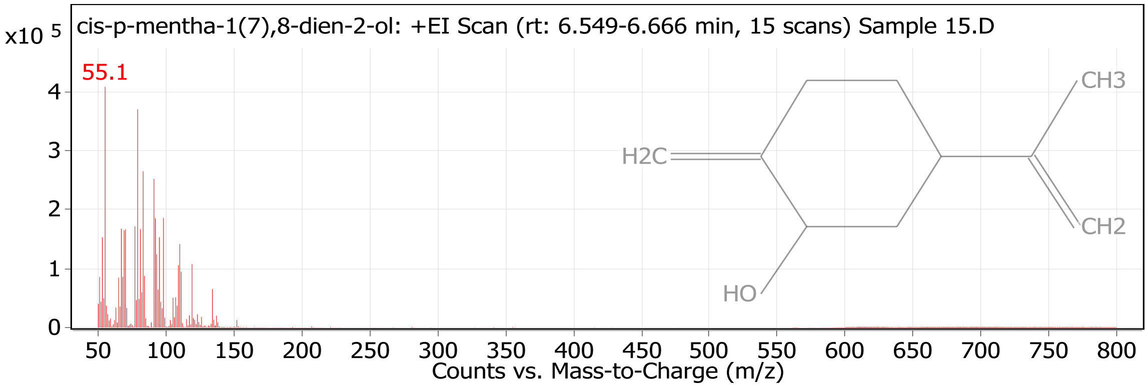

Library Spectrum

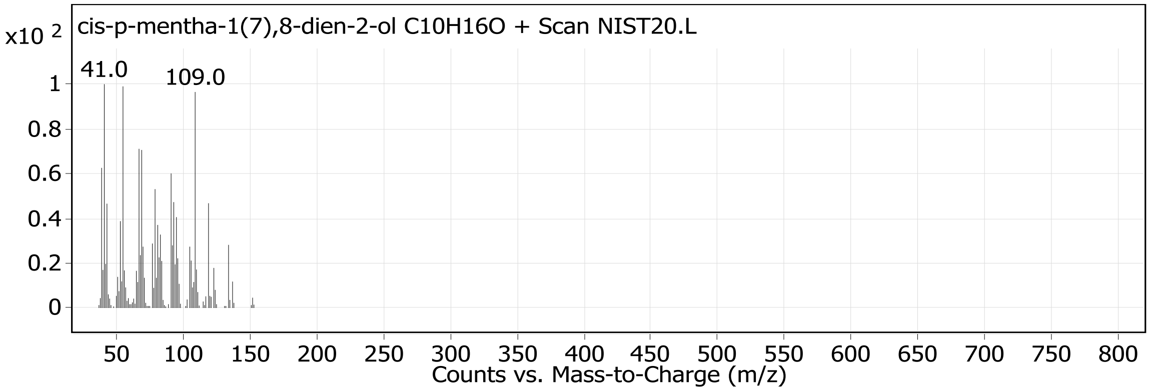

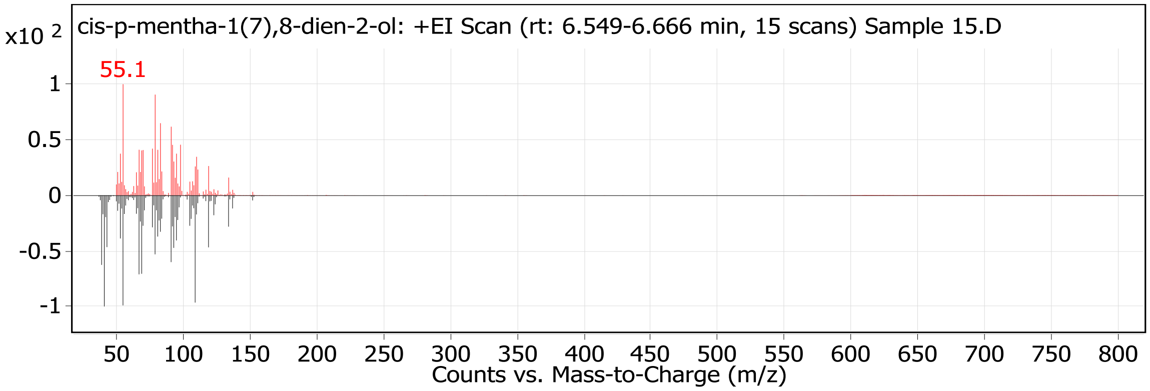

Spectrum Structure

cis-p-mentha-1(7),8-dien-2-ol

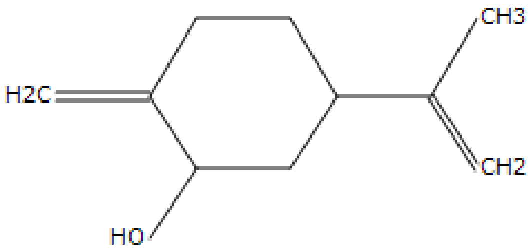

Spectrum Source	Collision Energy	Ionization Mode
Peak (8) in "+ TIC Scan"	0	EI

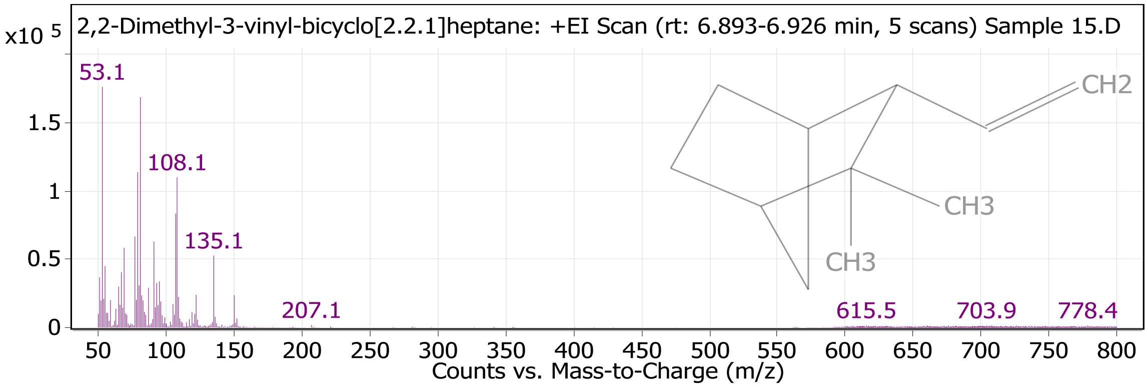

Library Spectrum

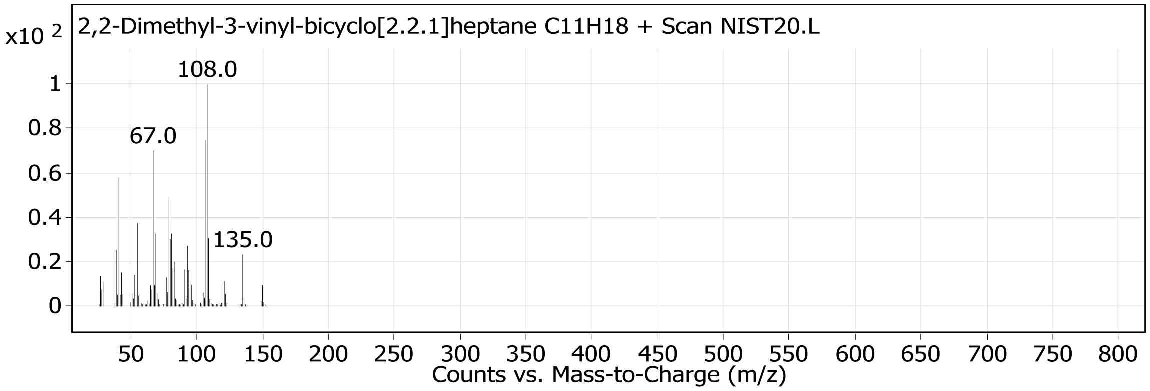

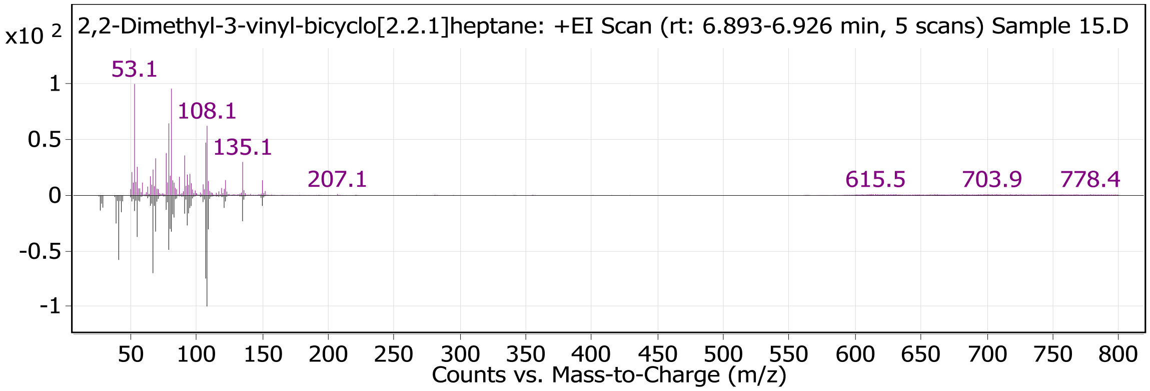

Spectrum Structure

2,2-Dimethyl-3-vinyl-bicyclo[2.2.1]heptane

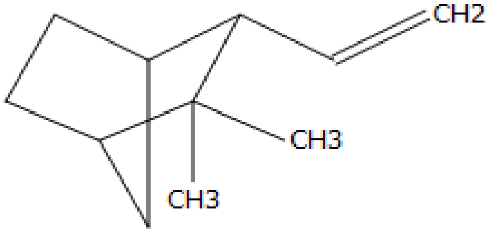

Qualitative Analysis Report

Spectrum Source	Collision Energy	Ionization Mode
Peak (9) in "+ TIC Scan"	0	EI

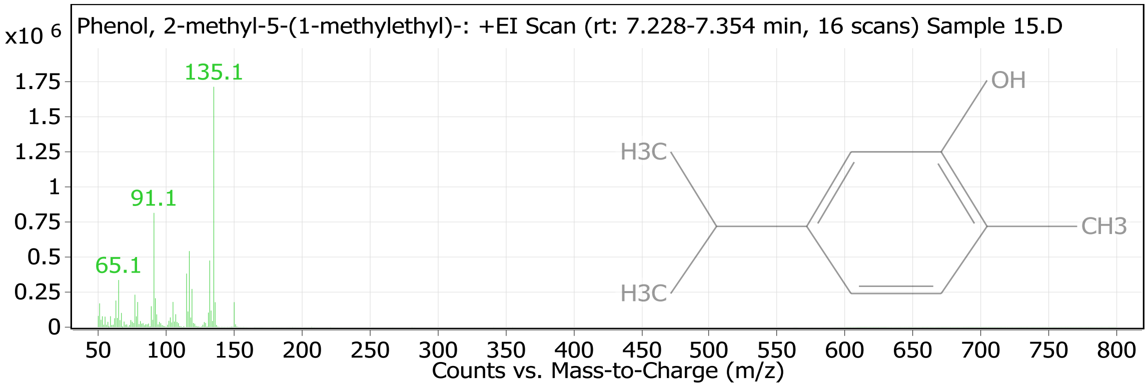

Library Spectrum

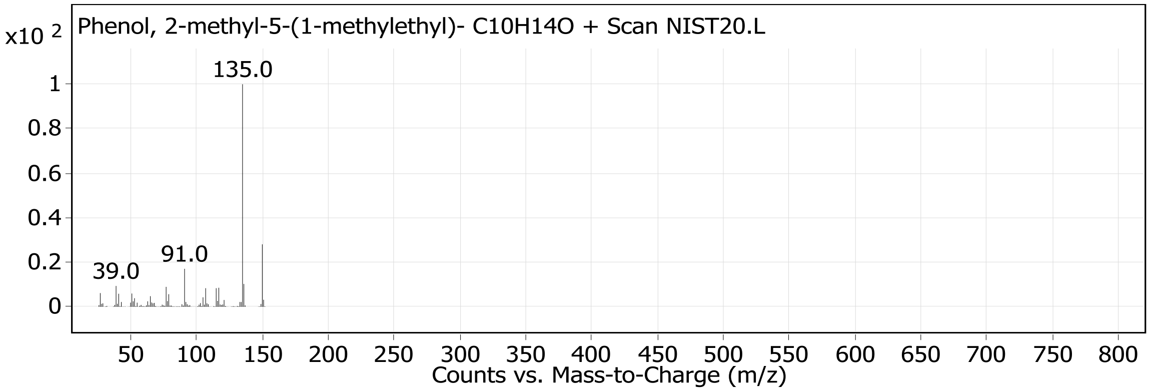

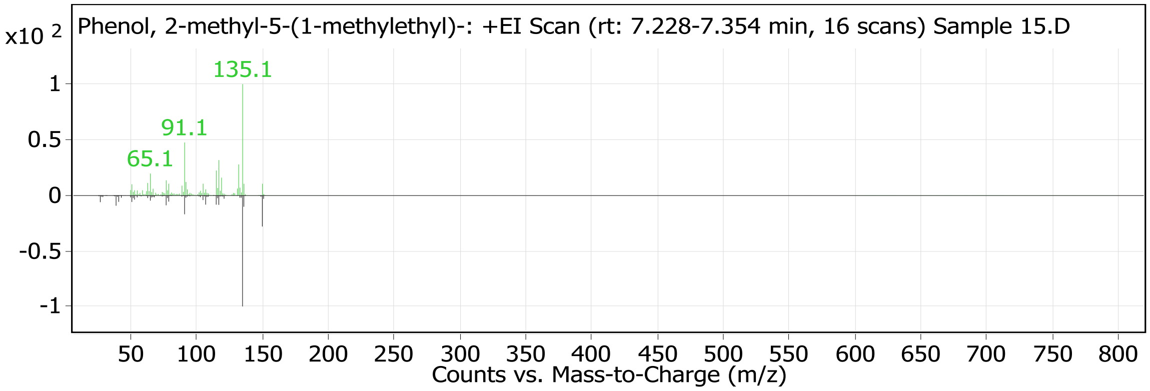

Spectrum Structure

Phenol, 2-methyl-5-(1-methylethyl)-

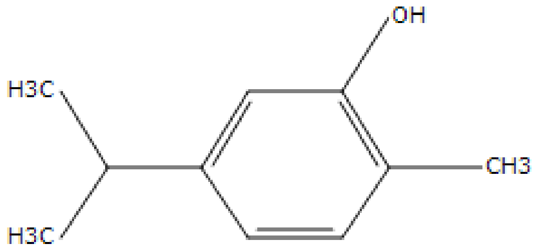

**Spectrum Source**	**Collision Energy**	**Ionization Mode**
Peak (10) in "+ TIC Scan"	0	EI

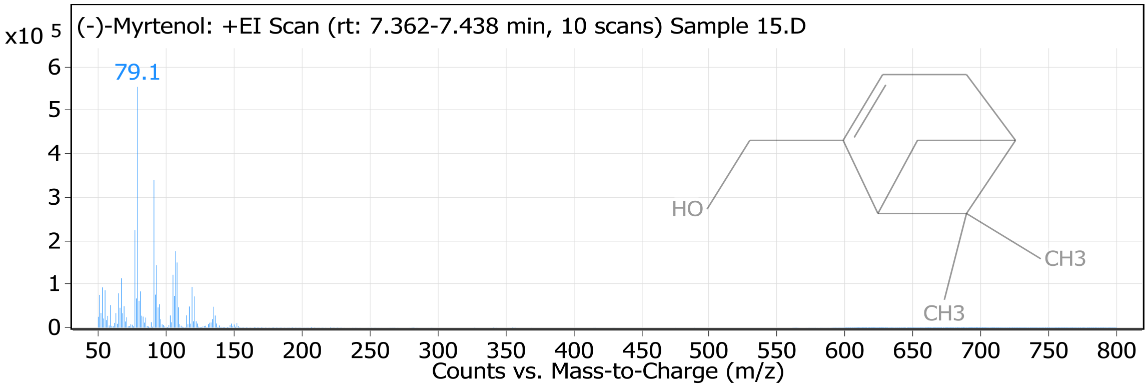

Library Spectrum

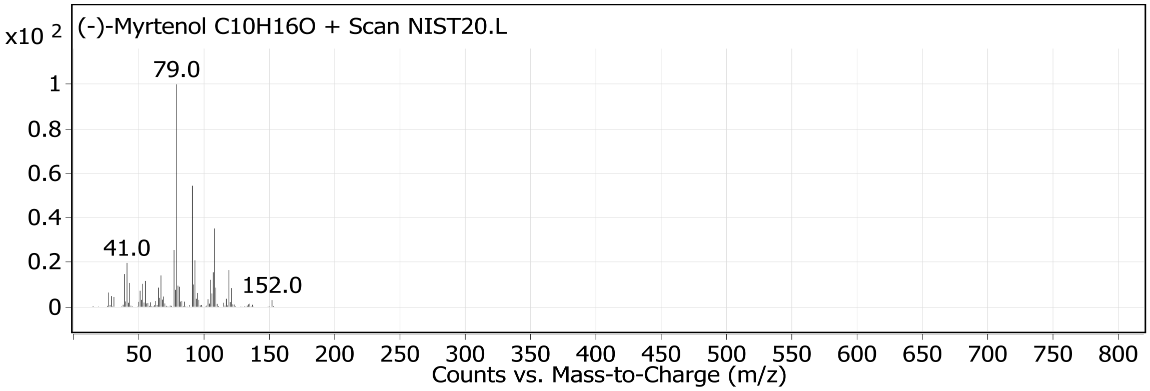

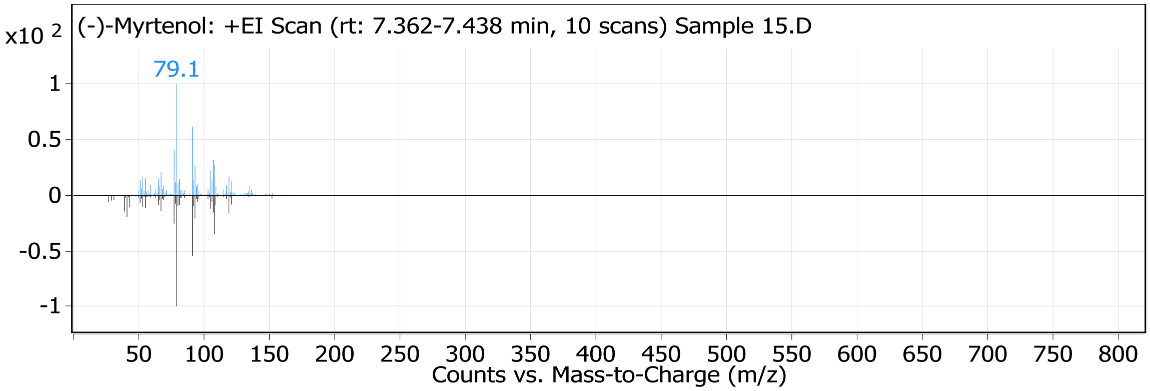

Spectrum Structure

(-)-Myrtenol

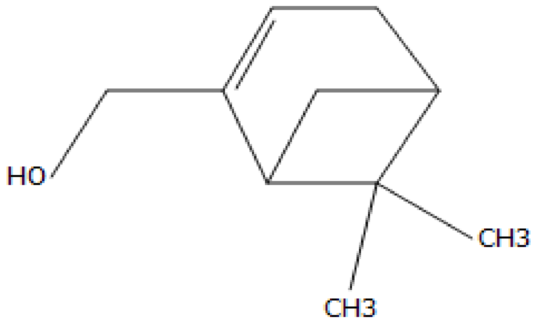

Qualitative Analysis Report

**Spectrum Source**	**Collision Energy**	**Ionization Mode**
Peak (11) in "+ TIC Scan"	0	EI

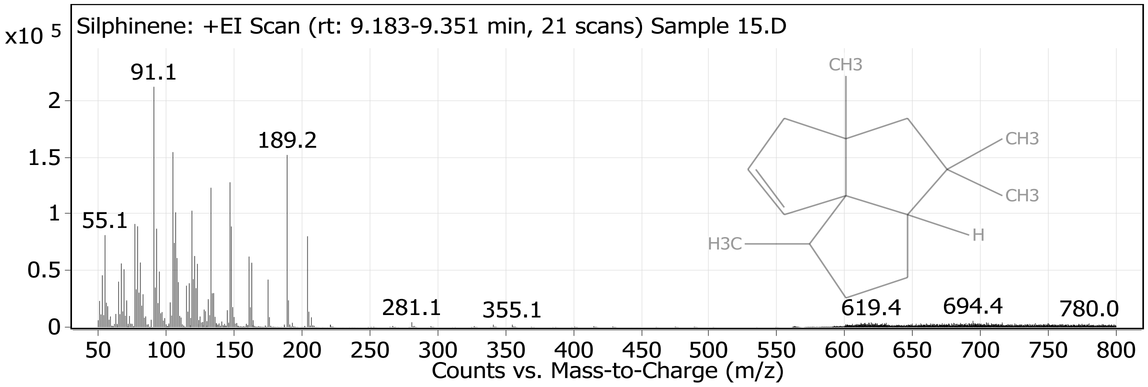

Library Spectrum

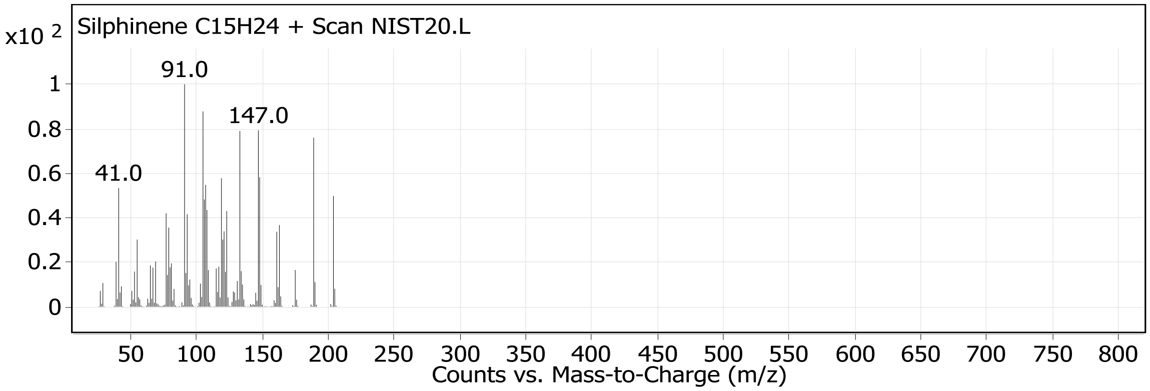

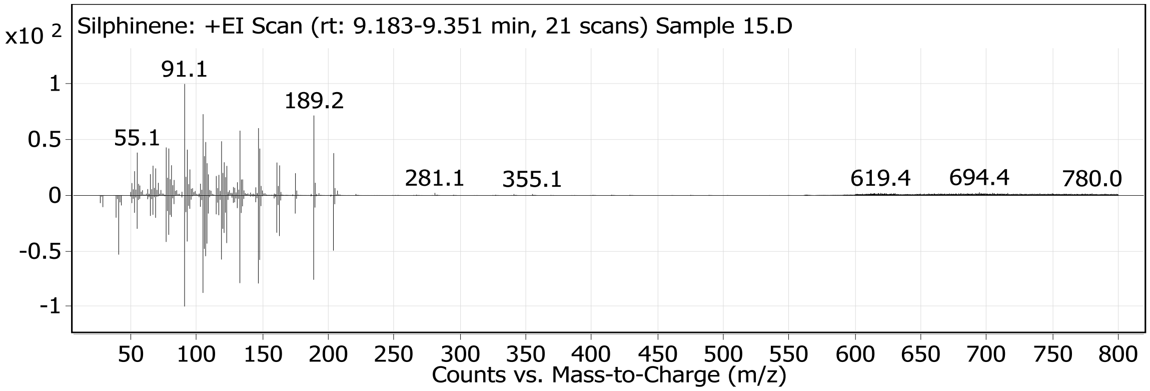

Spectrum Structure

Silphinene

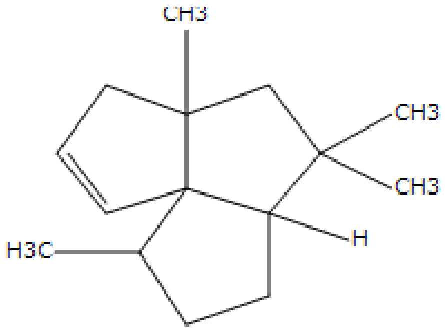

Spectrum Source	Collision Energy	Ionization Mode
Peak (12) in "+ TIC Scan"	0	EI

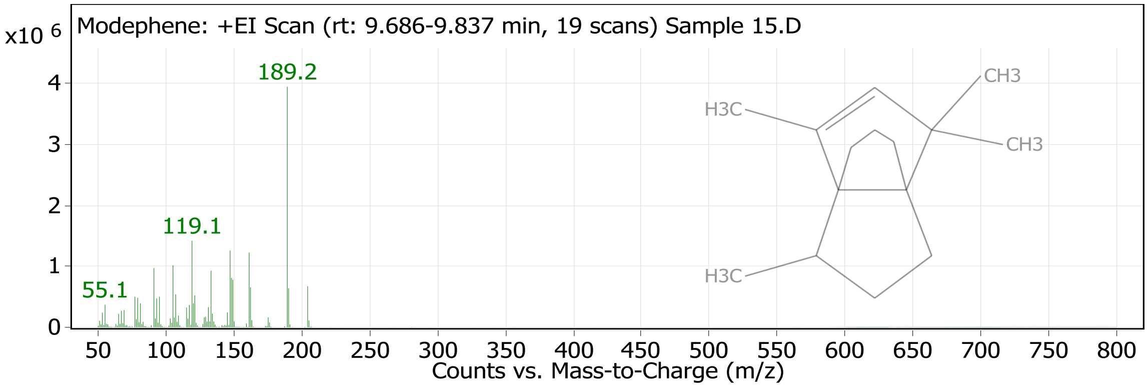

Library Spectrum

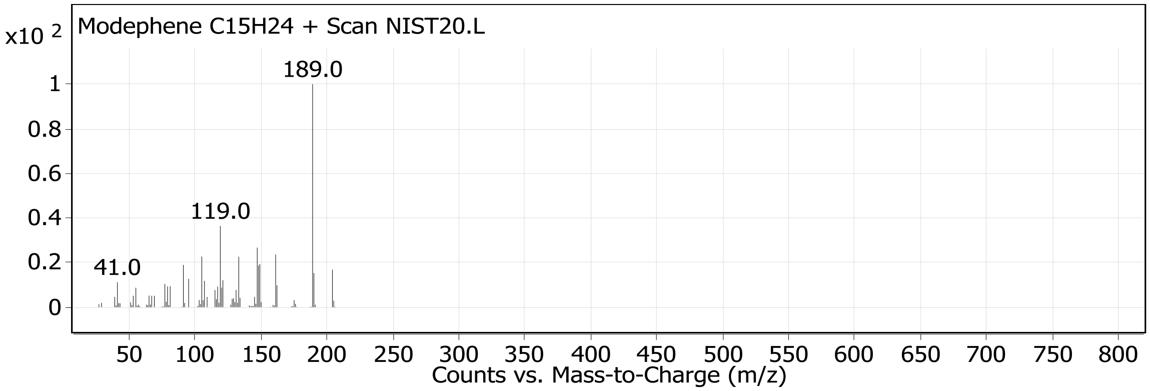

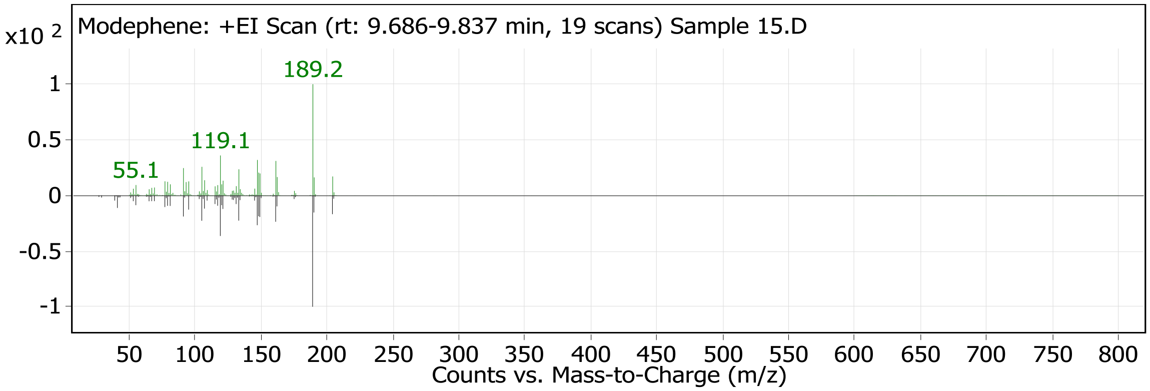

Spectrum Structure

Modephene

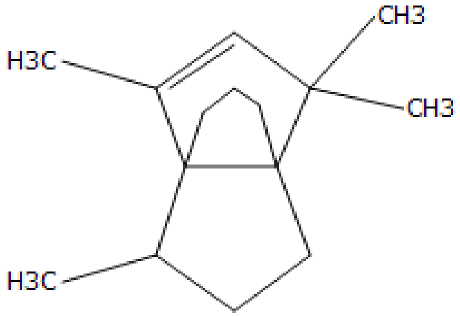

**Spectrum Source**	**Collision Energy**	**Ionization Mode**
Peak (13) in "+ TIC Scan"	0	EI

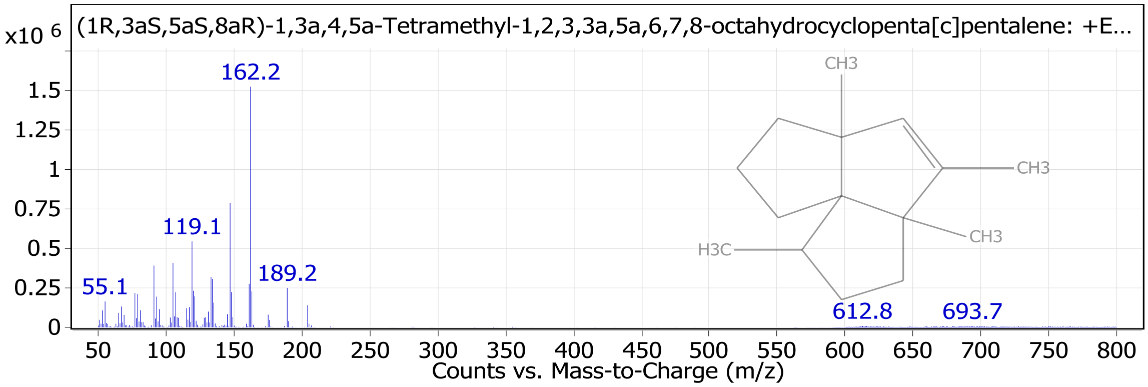

Library Spectrum

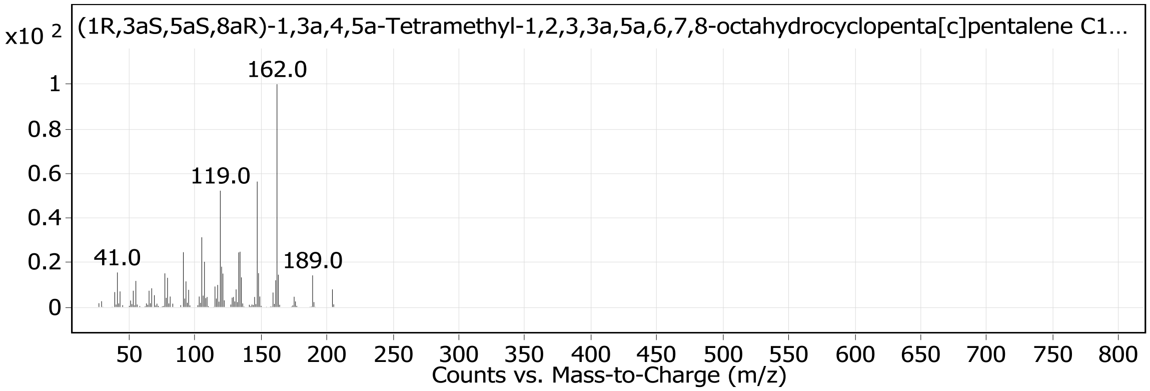

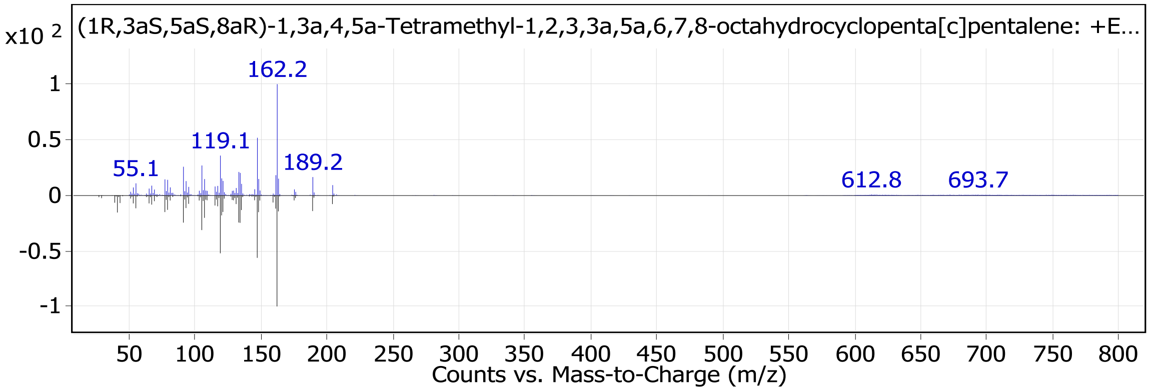

Spectrum Structure

(1R,3aS,5aS,8aR)-1,3a,4,5a-Tetramethyl-1,2,3,3a,5a,6,7,8-octahydrocyclopenta[c]pentalene

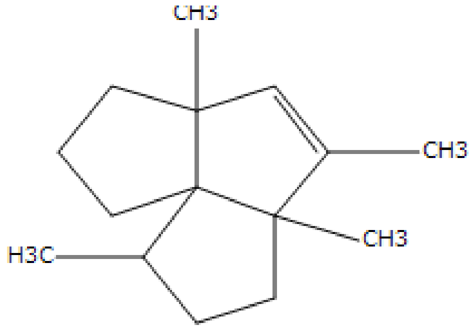

**Spectrum Source**	**Collision Energy**	**Ionization Mode**
Peak (14) in "+ TIC Scan"	0	EI

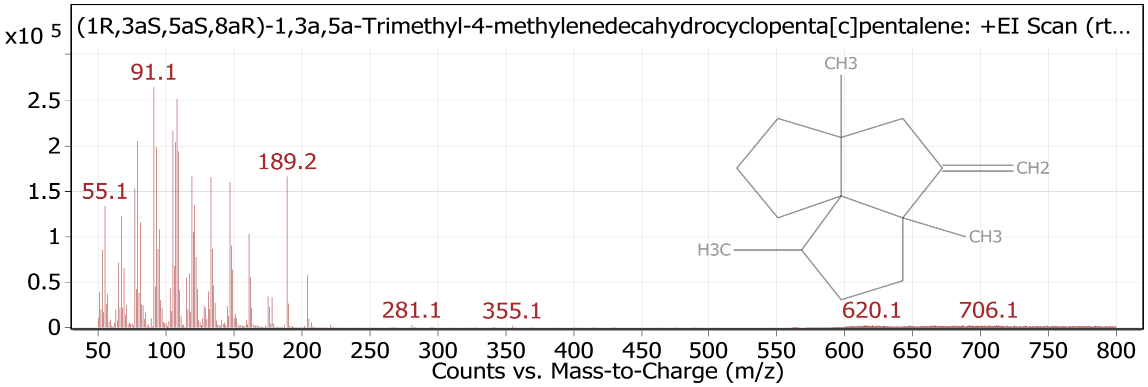

Library Spectrum

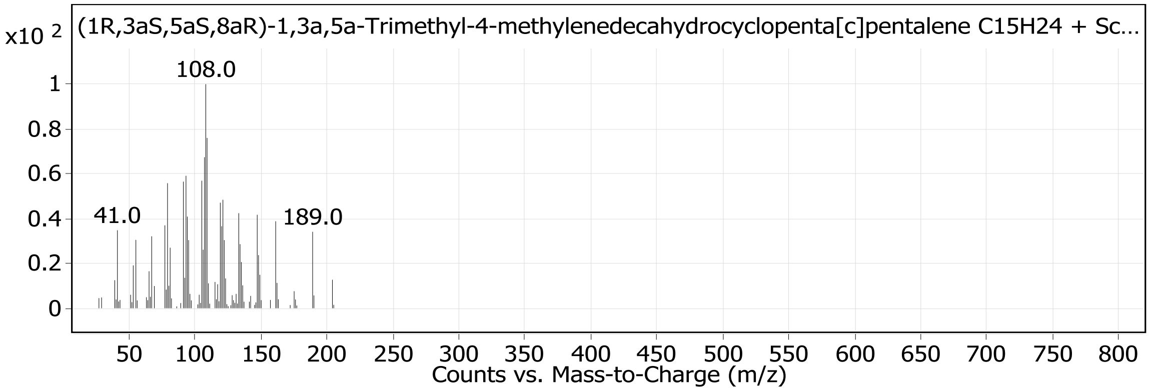

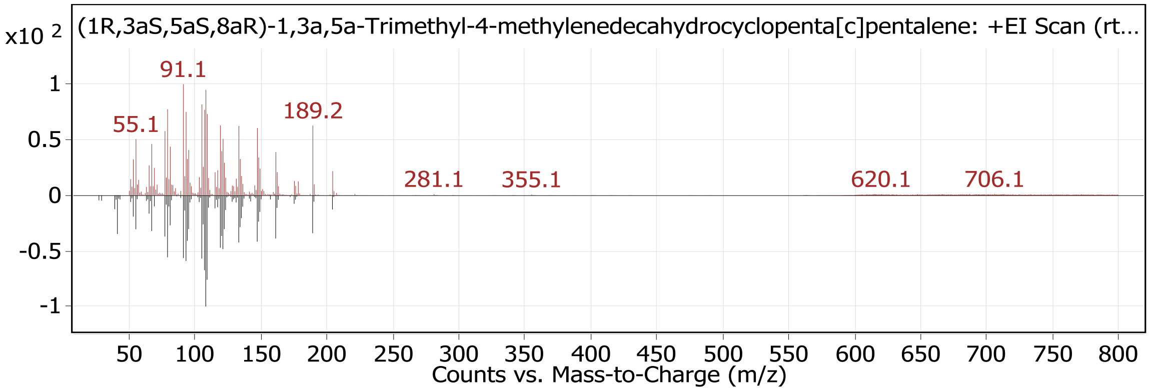

Spectrum Structure

(1R,3aS,5aS,8aR)-1,3a,5a-Trimethyl-4-methylenedecahydrocyclopenta[c]pentalene

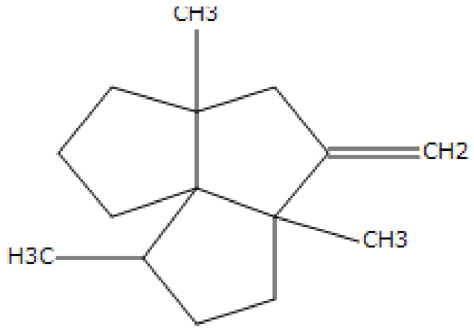

Spectrum Source	Collision Energy	Ionization Mode
Peak (15) in "+ TIC Scan"	0	EI

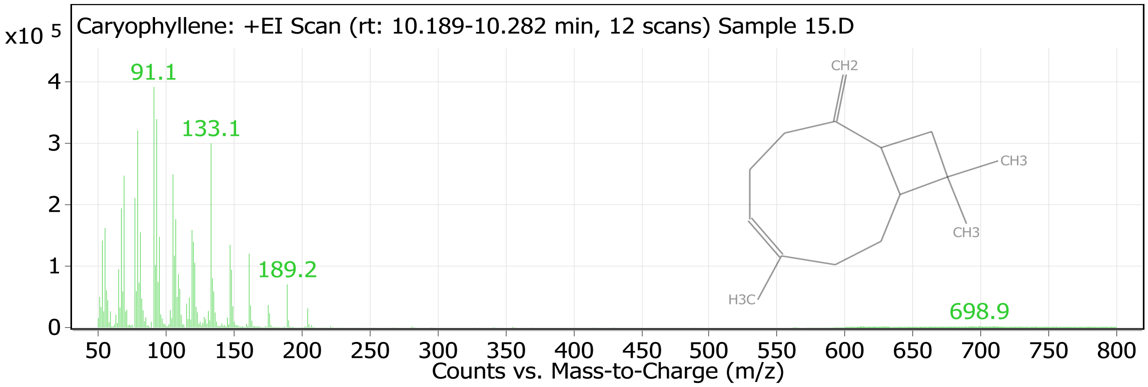

Library Spectrum

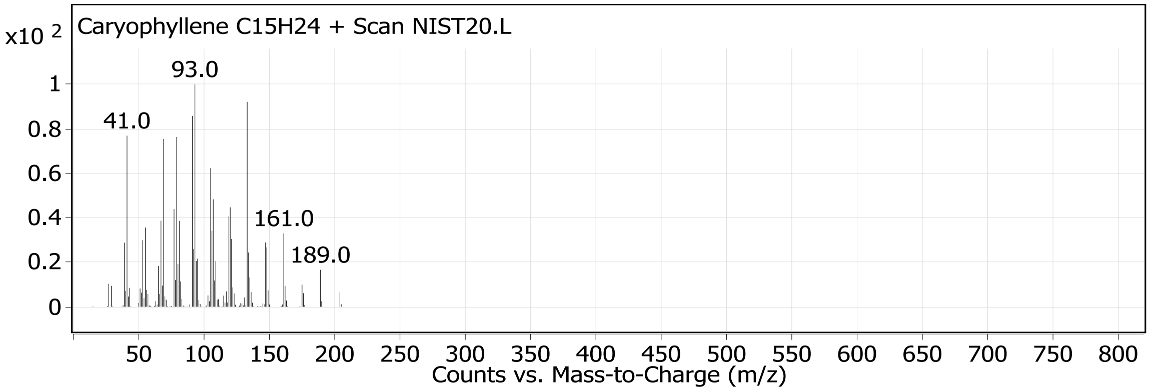

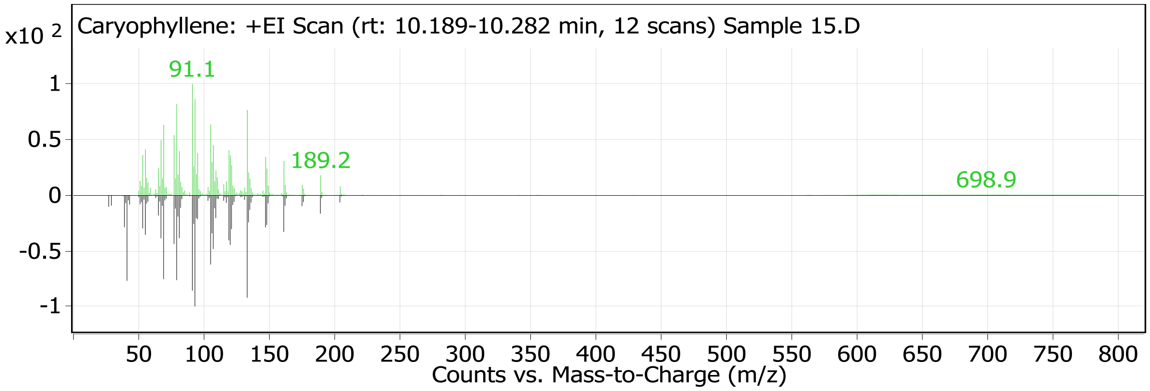

Spectrum Structure

Caryophyllene

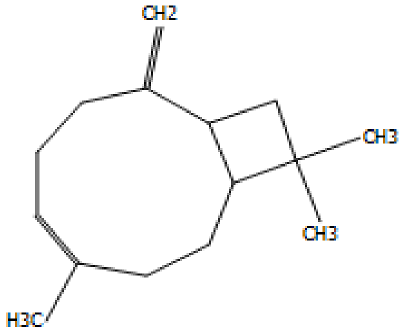

Qualitative Analysis Report

Spectrum Source	Collision Energy	Ionization Mode
Peak (16) in "+ TIC Scan"	0	EI

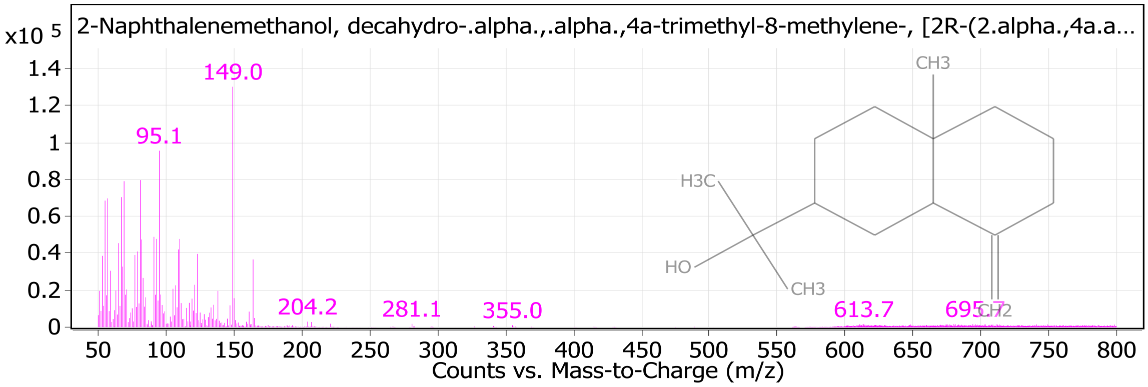

Library Spectrum

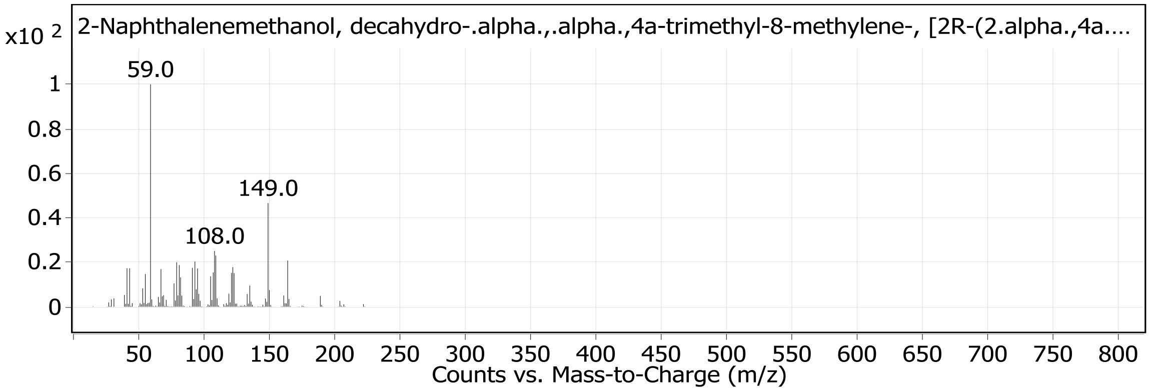

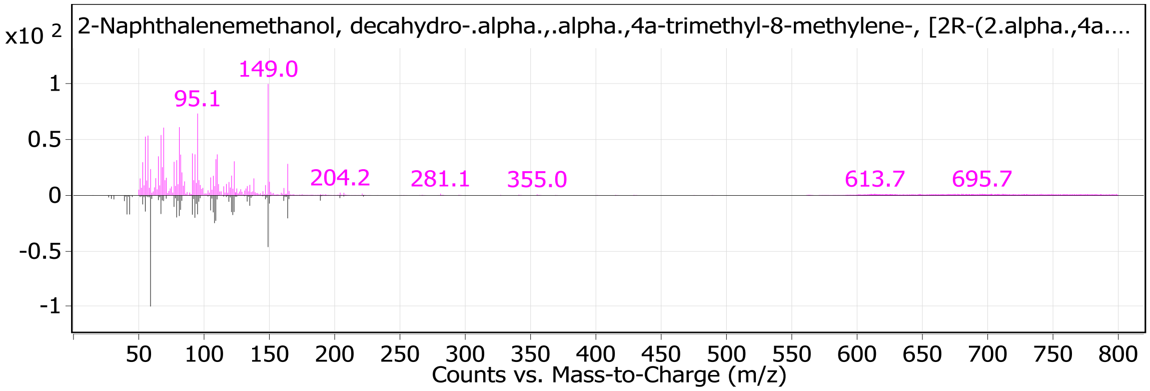

Spectrum Structure

2-Naphthalenemethanol, decahydro-.alpha.,.alpha.,4a-trimethyl-8-methylene-, [2R-(2.alpha.,4a.alpha.,8a.beta.)]-

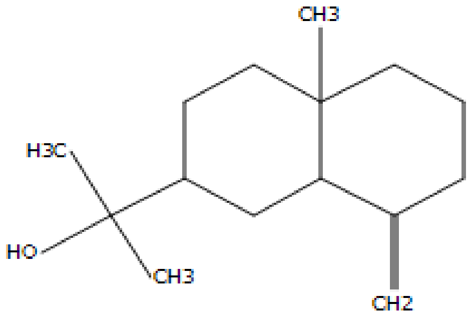

Spectrum Source	Collision Energy	Ionization Mode
Peak (17) in "+ TIC Scan"	0	EI

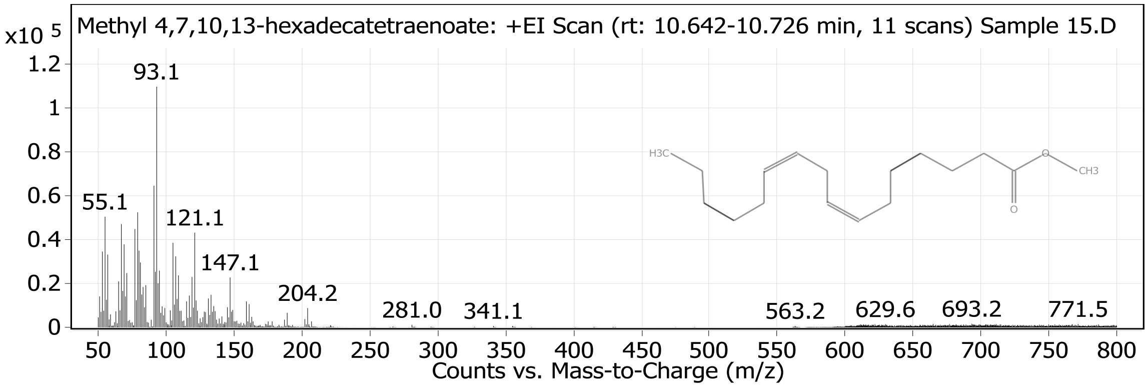

Library Spectrum

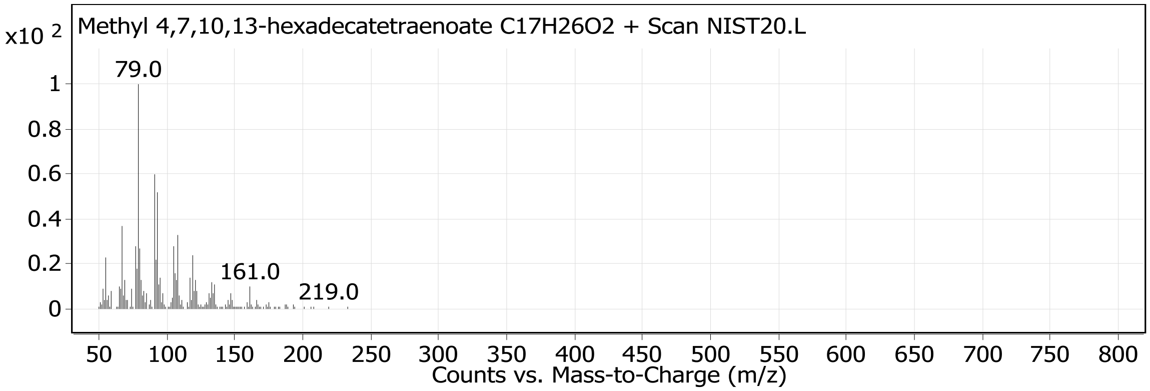

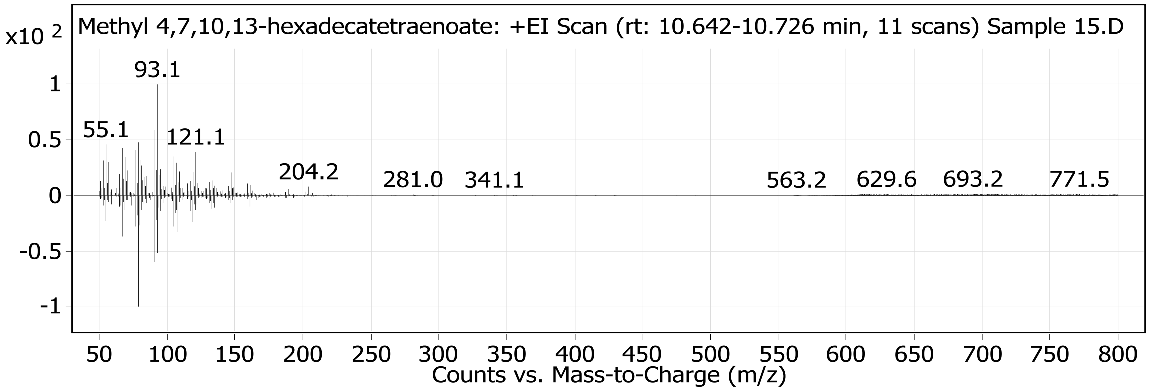

Spectrum Structure

Methyl 4,7,10,13-hexadecatetraenoate

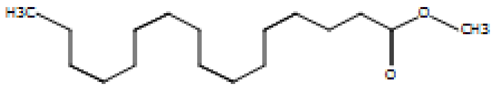

**Spectrum Source**	**Collision Energy**	**Ionization Mode**
Peak (18) in "+ TIC Scan"	0	EI

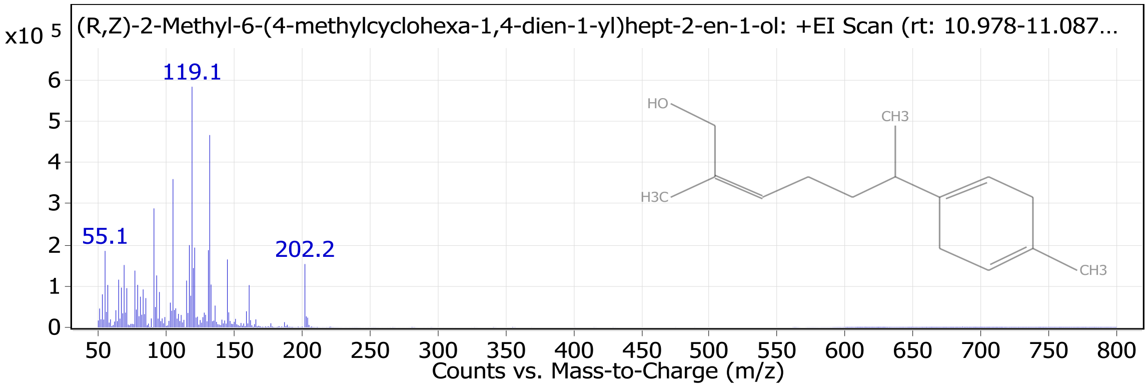

Library Spectrum

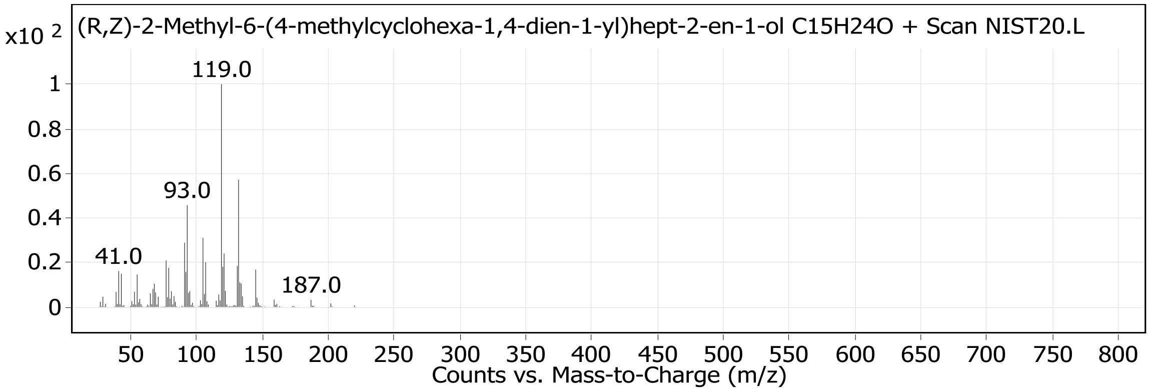

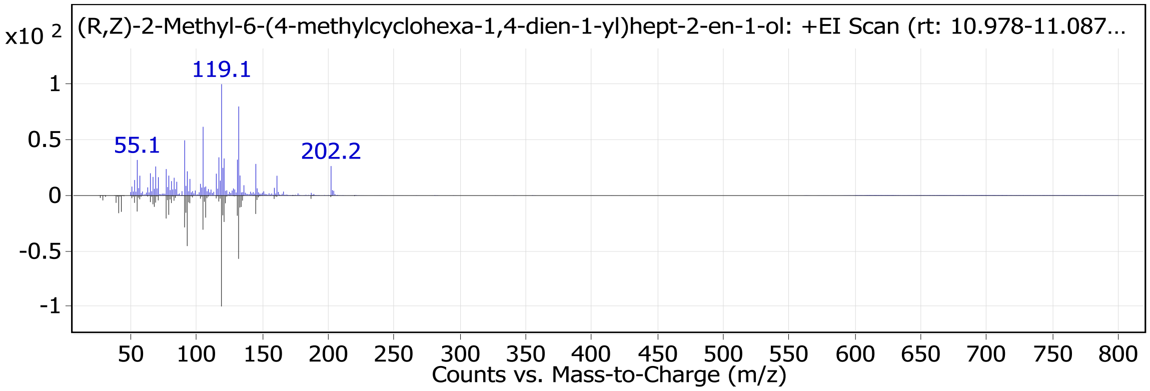
x10 2
(R,Z)-2-Methyl-6-(4-methylcyclohexa-1,4-dien-1-yl)hept-2-en-1-ol: +EI Scan (rt: 10.978-11.087...
119.1
55.1
202.2
1
0.5
0
-0.5
-1
50 100 150 200 250 300 350 400 450 500 550 600 650 700 750 800
Counts vs. Mass-to-Charge (m/z)

Spectrum Structure

(R,Z)-2-Methyl-6-(4-methylcyclohexa-1,4-dien-1-yl)hept-2-en-1-ol

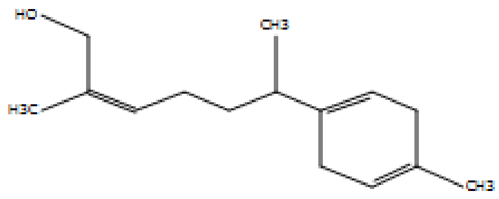

Spectrum Source	Collision Energy	Ionization Mode
Peak (19) in "+ TIC Scan"	0	EI

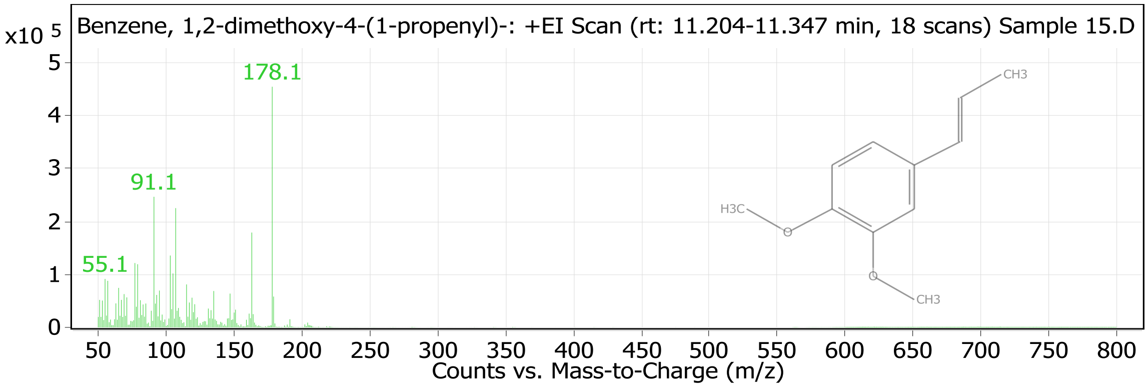

Library Spectrum

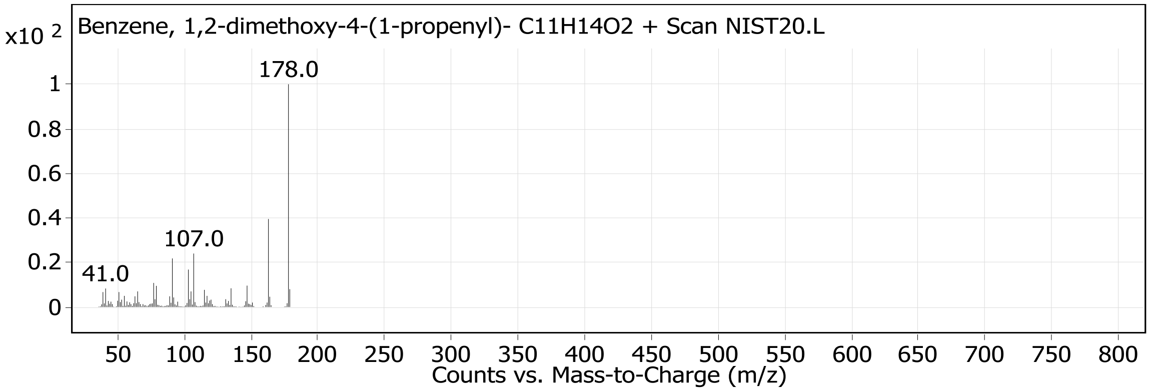

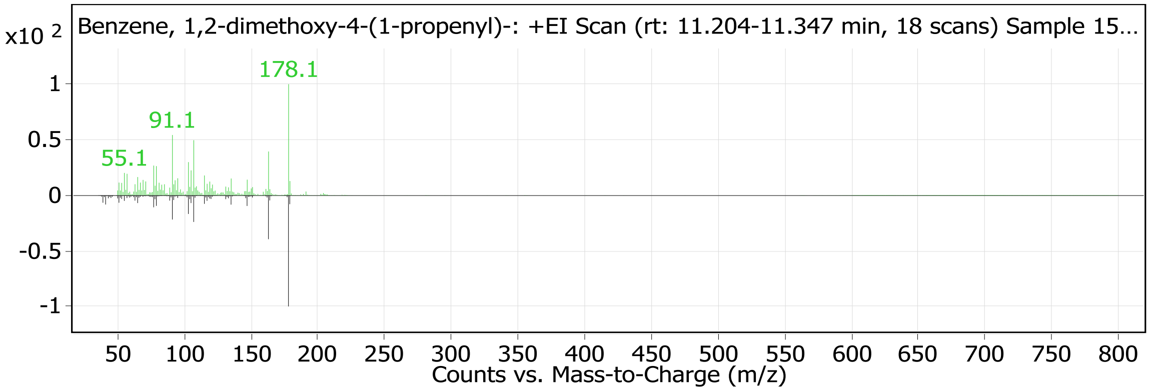

Spectrum Structure

Benzene, 1,2-dimethoxy-4-(1-propenyl)-

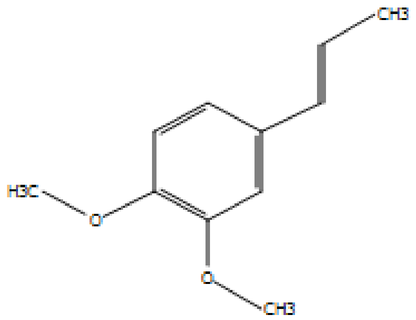

**Spectrum Source**	**Collision Energy**	**Ionization Mode**
Peak (20) in "+ TIC Scan"	0	EI

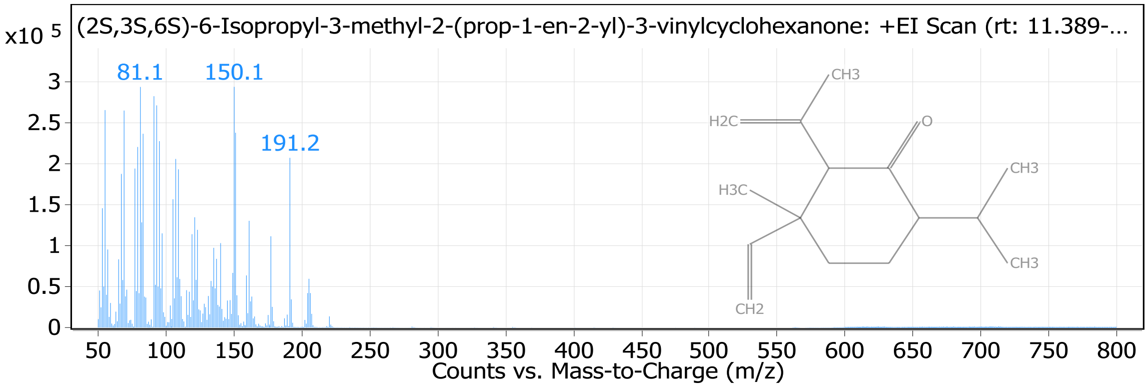

Library Spectrum

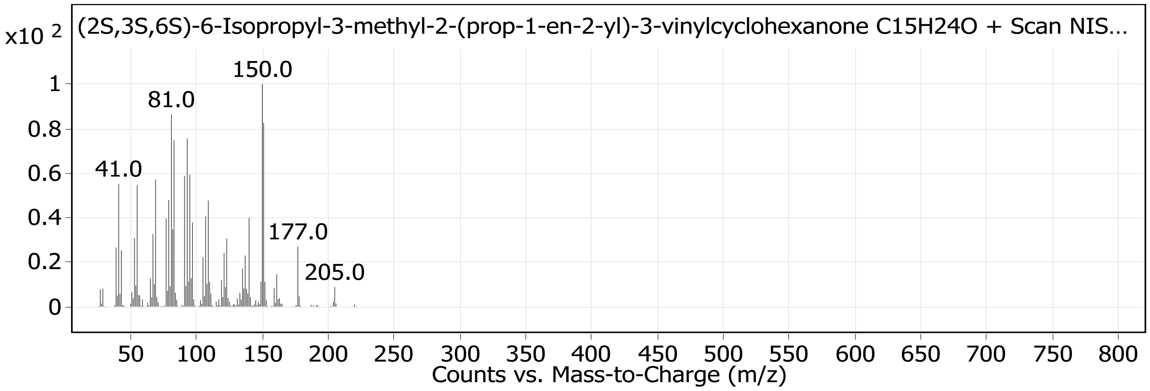

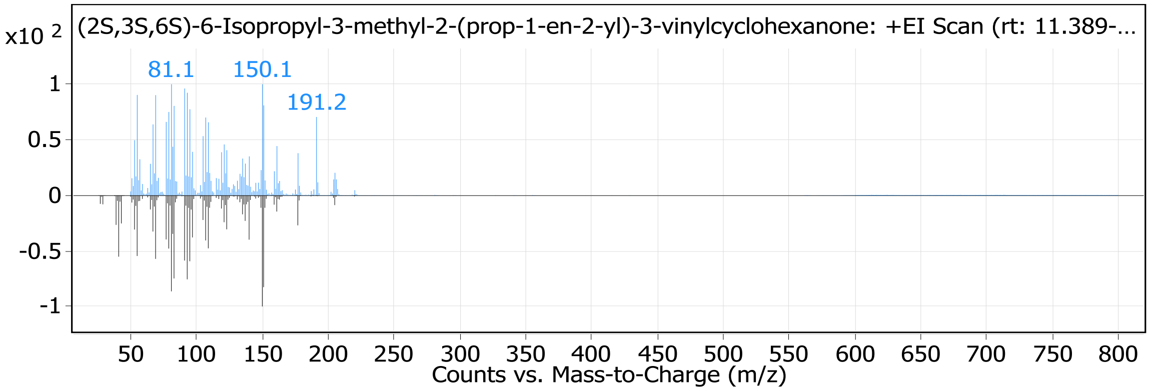

Spectrum Structure

(2S,3S,6S)-6-Isopropyl-3-methyl-2-(prop-1-en-2-yl)-3-vinylcyclohexanone

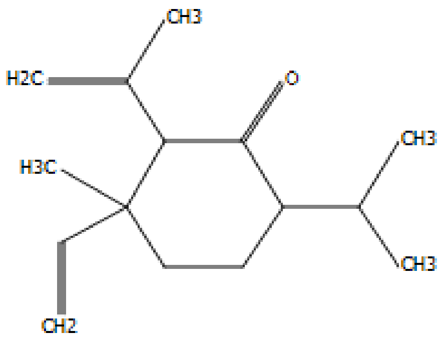

Spectrum Source	Collision Energy	Ionization Mode
Peak (21) in "+ TIC Scan"	0	EI

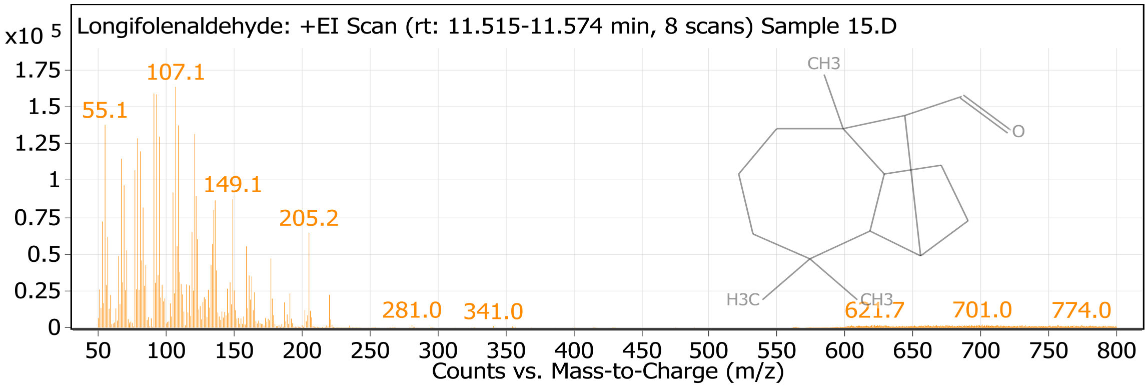

Library Spectrum

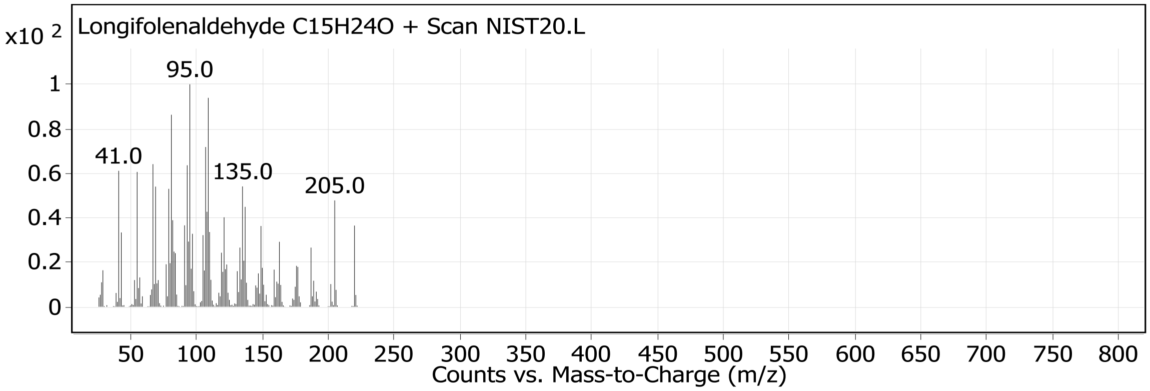

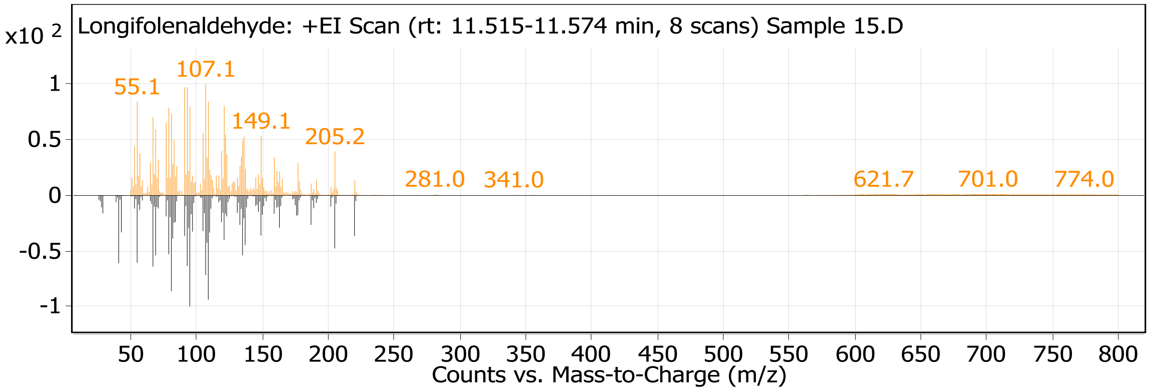

Spectrum Structure

Longifolenaldehyde

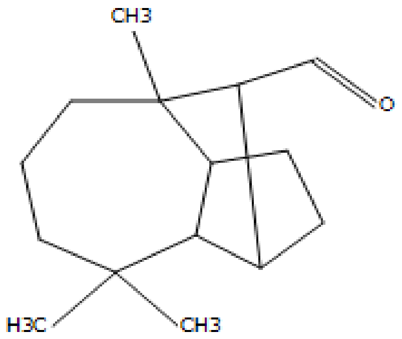

Spectrum Source	Collision Energy	Ionization Mode
Peak (22) in "+ TIC Scan"	0	EI

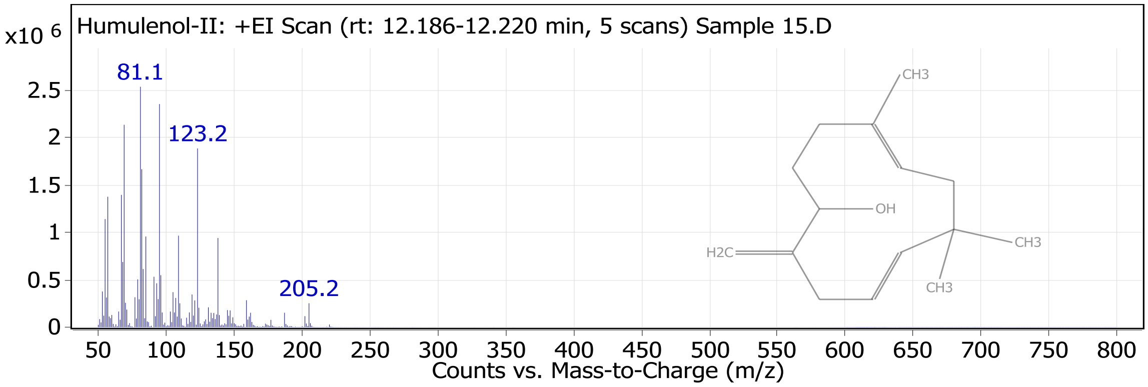

Library Spectrum

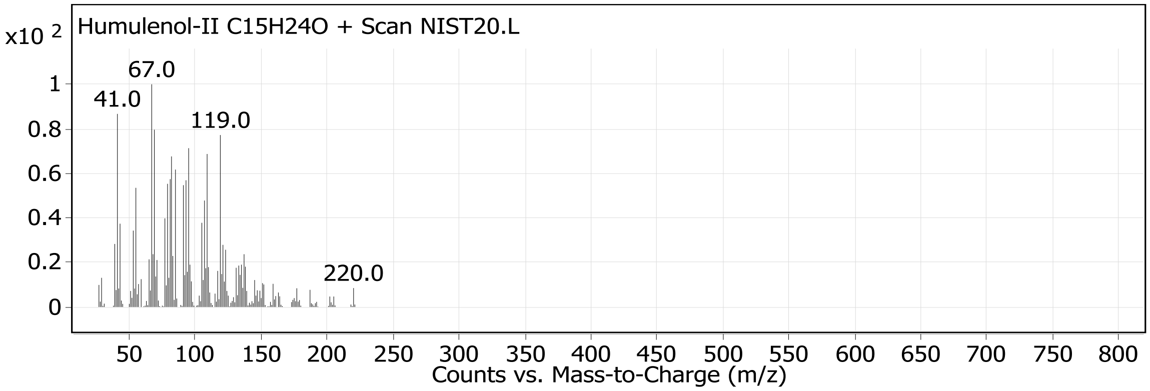

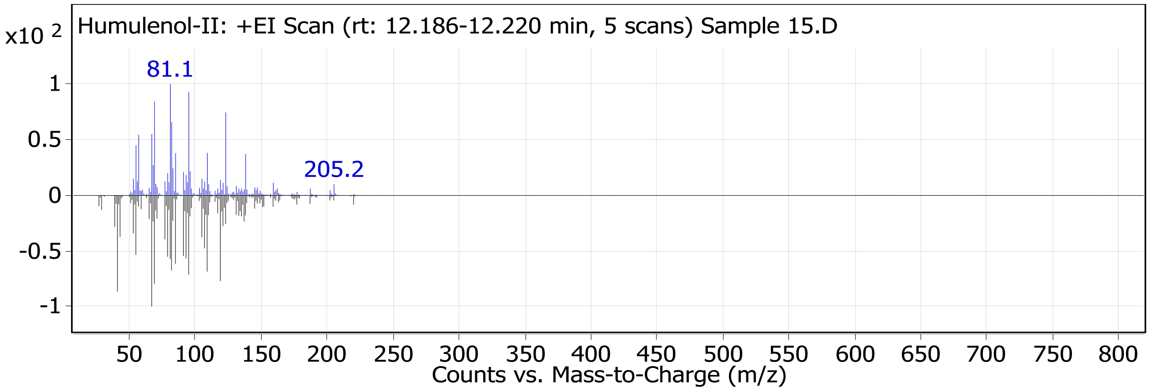

Spectrum Structure

Humulenol-II

Qualitative Analysis Report

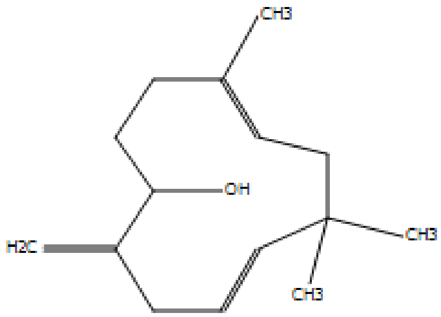

Spectrum Source	Collision Energy	Ionization Mode
Peak (23) in "+ TIC Scan"	0	EI

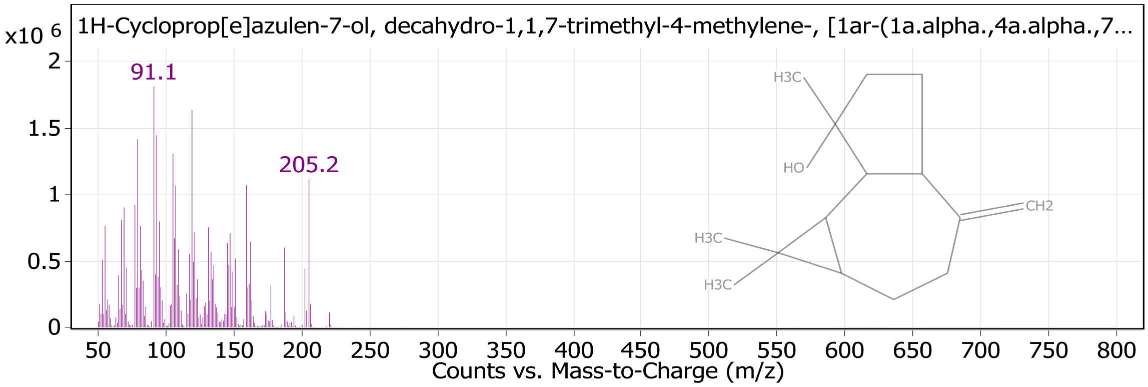

Library Spectrum

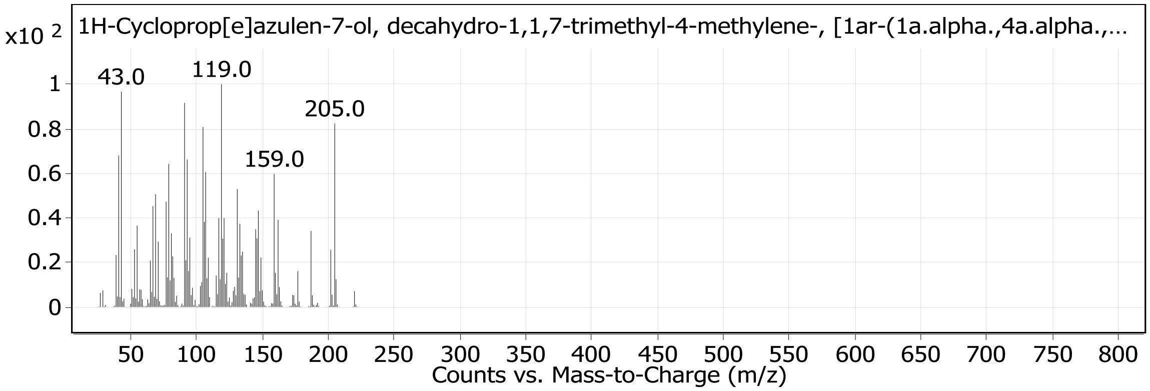

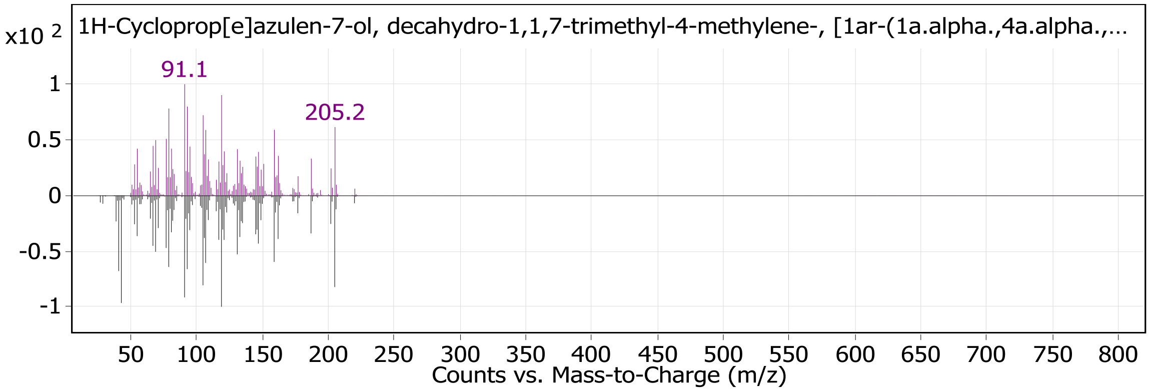

Qualitative Analysis Report

Spectrum Structure

1H-Cycloprop[e]azulen-7-ol, decahydro-1,1,7-trimethyl-4-methylene-, [1ar-(1a.alpha.,4a.alpha.,7.beta.,7a.beta.,7b.alpha.)]-

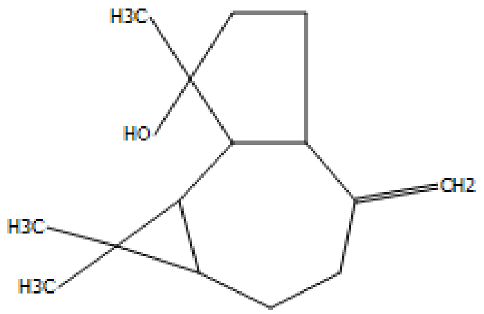

Spectrum Source	Collision Energy	Ionization Mode
Peak (24) in "+ TIC Scan"	0	EI

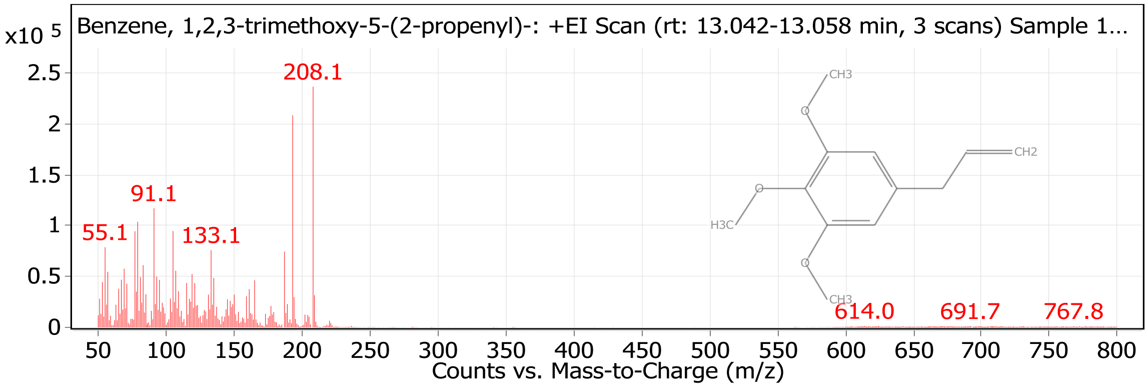

Library Spectrum

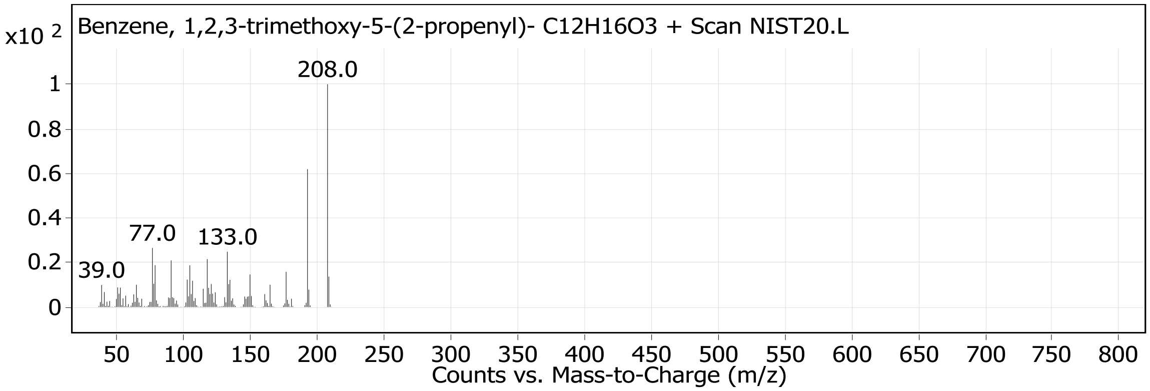

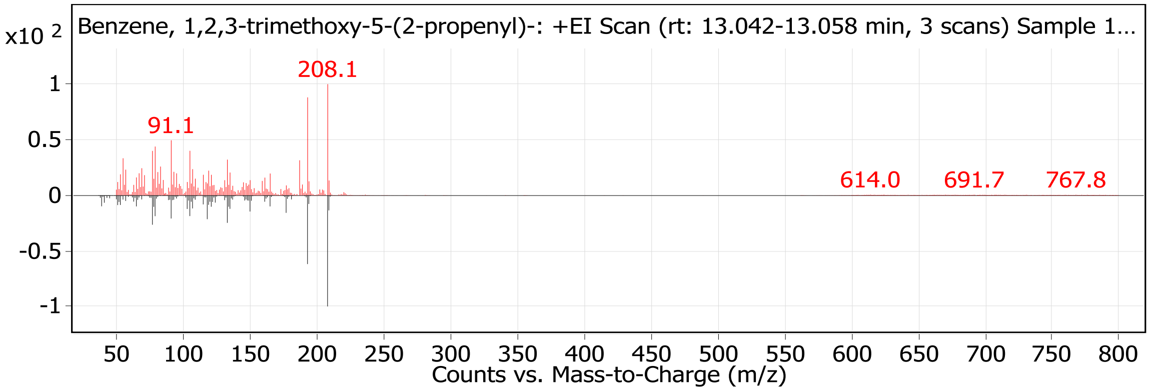

Spectrum Structure

Benzene, 1,2,3-trimethoxy-5-(2-propenyl)-

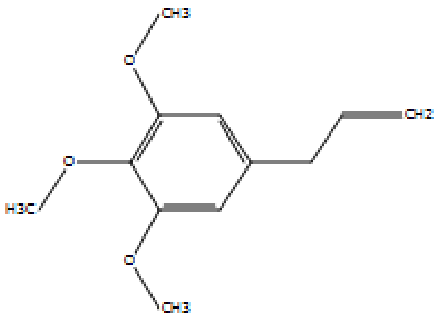

**Spectrum Source**	**Collision Energy**	**Ionization Mode**
Peak (25) in "+ TIC Scan"	0	EI

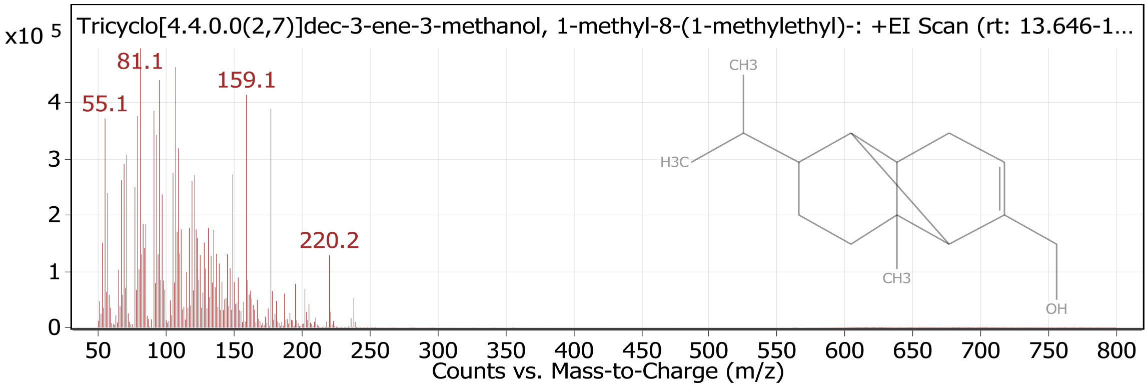

Library Spectrum

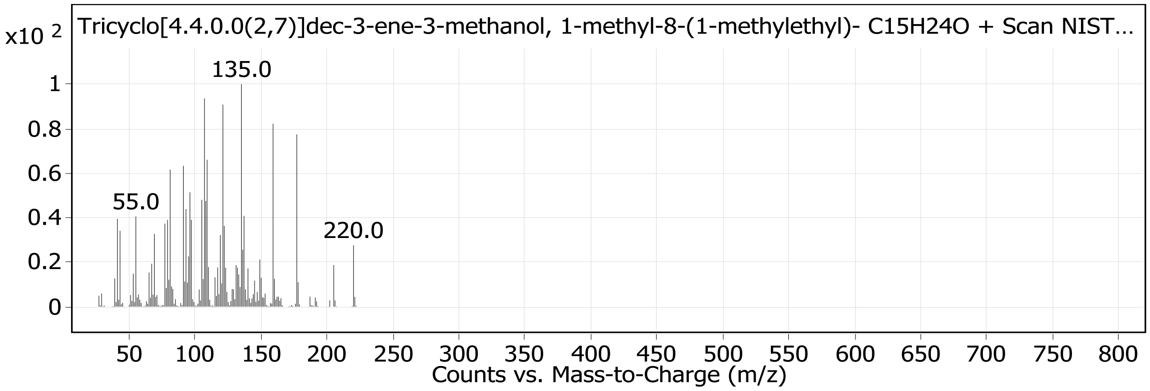

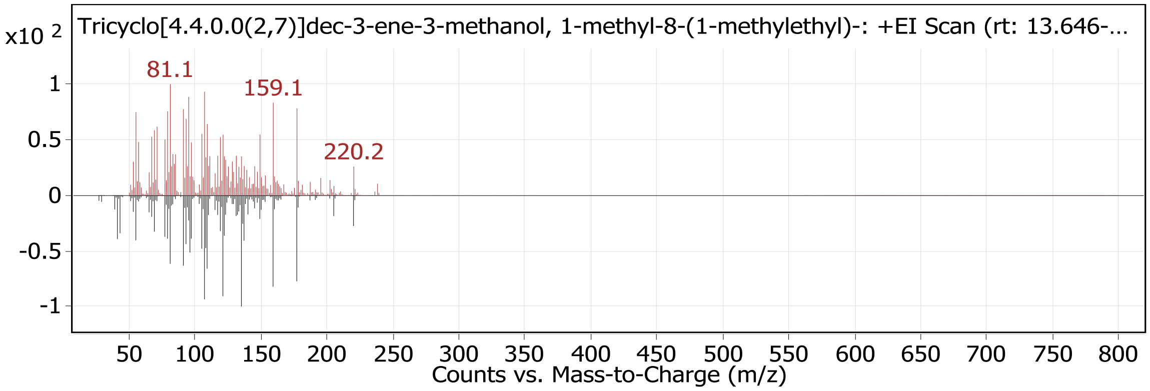

Spectrum Structure

Tricyclo[4.4.0.0(2,7)]dec-3-ene-3-methanol, 1-methyl-8-(1-methylethyl)-

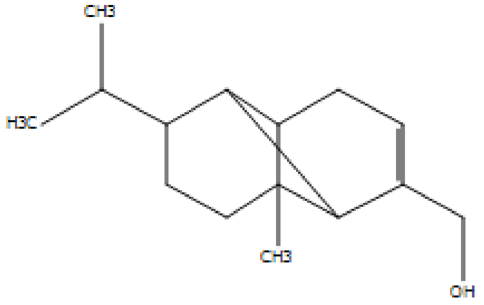

Spectrum Source	Collision Energy	Ionization Mode
Peak (26) in "+ TIC Scan"	0	EI

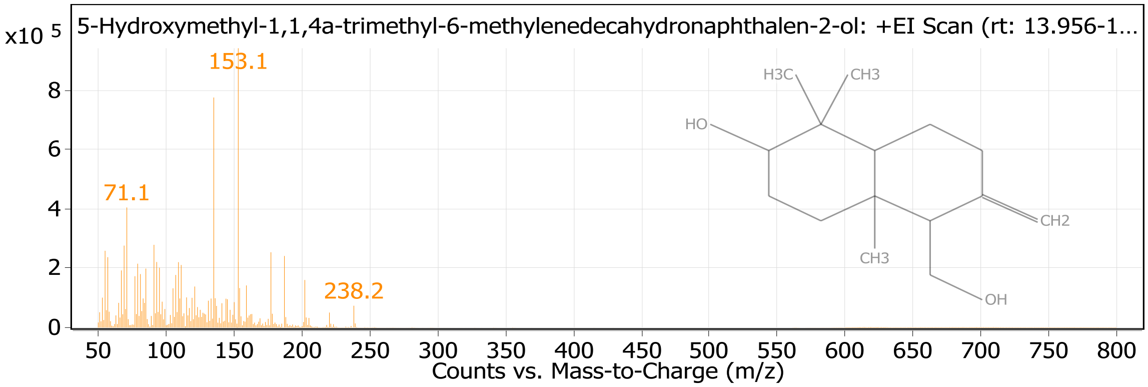

Library Spectrum

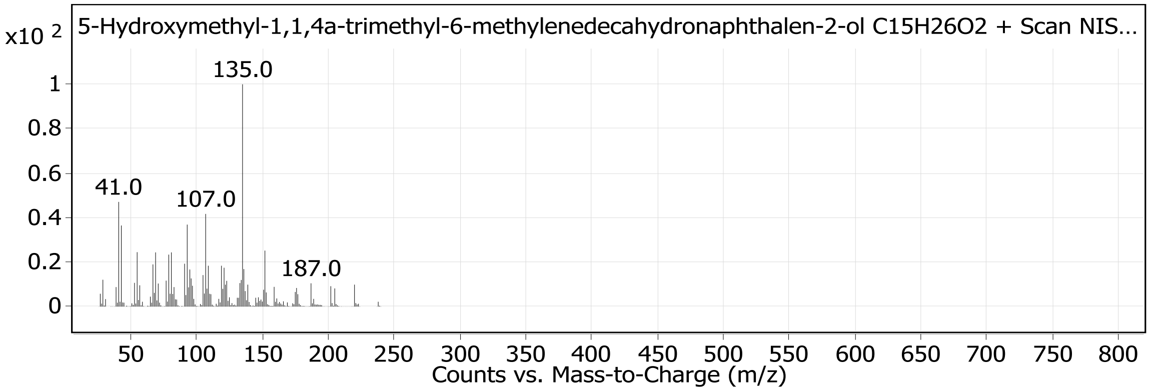

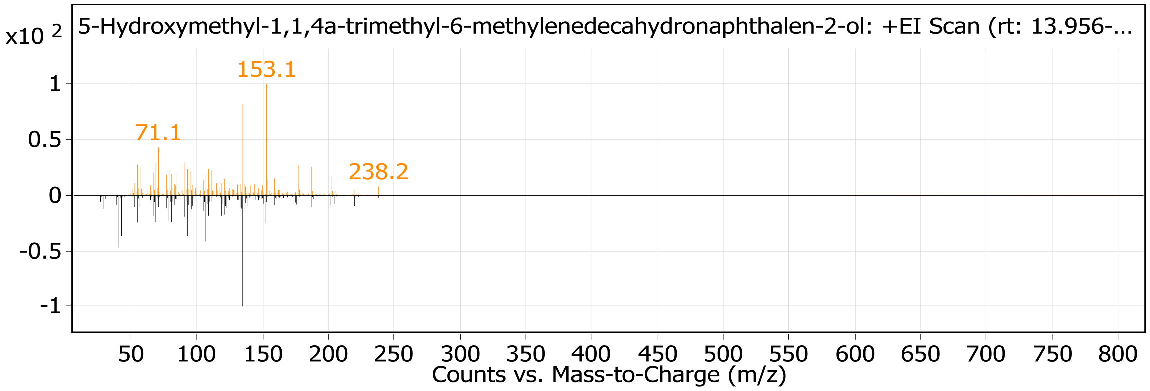

Spectrum Structure

5-Hydroxymethyl-1,1,4a-trimethyl-6-methylenedecahydronaphthalen-2-ol

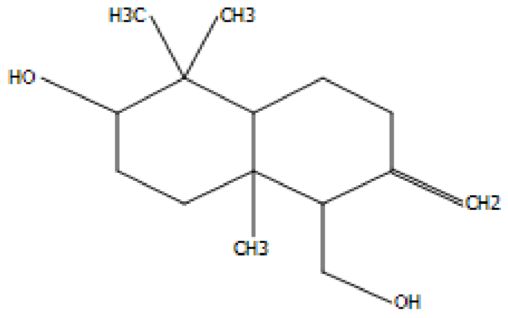

Qualitative Analysis Report

Spectrum Source	Collision Energy	Ionization Mode
Peak (27) in "+ TIC Scan"	0	EI

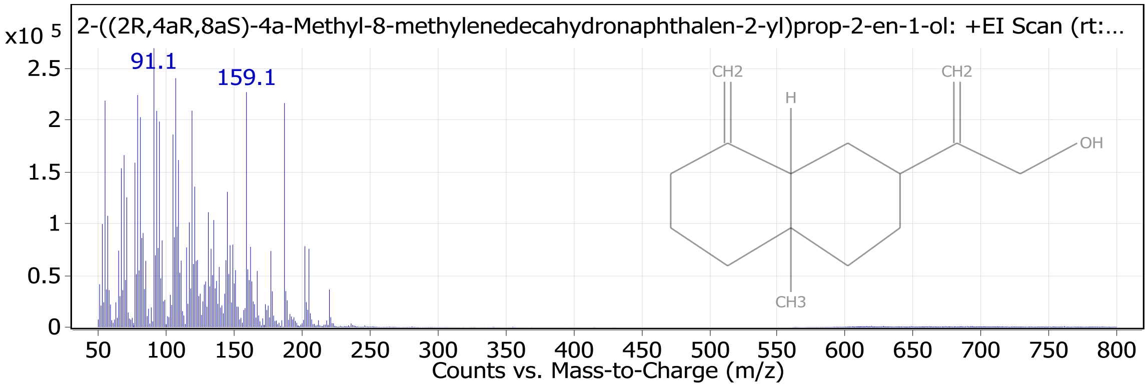

Library Spectrum

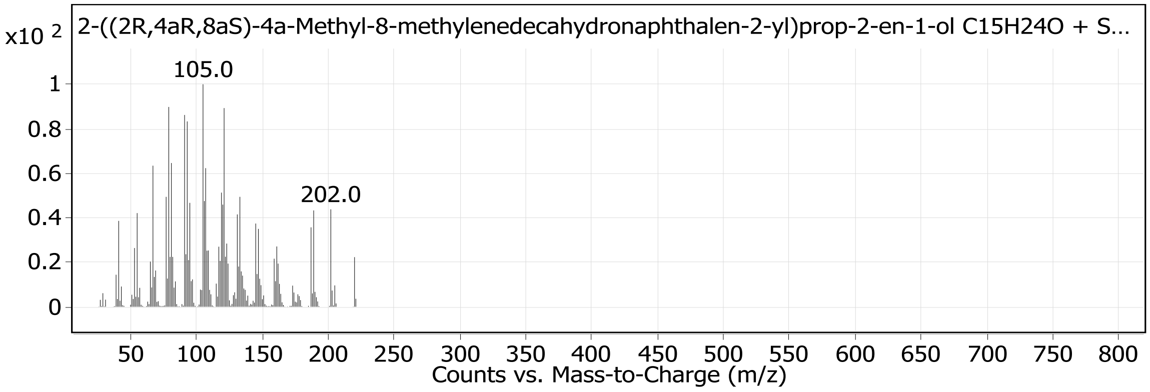

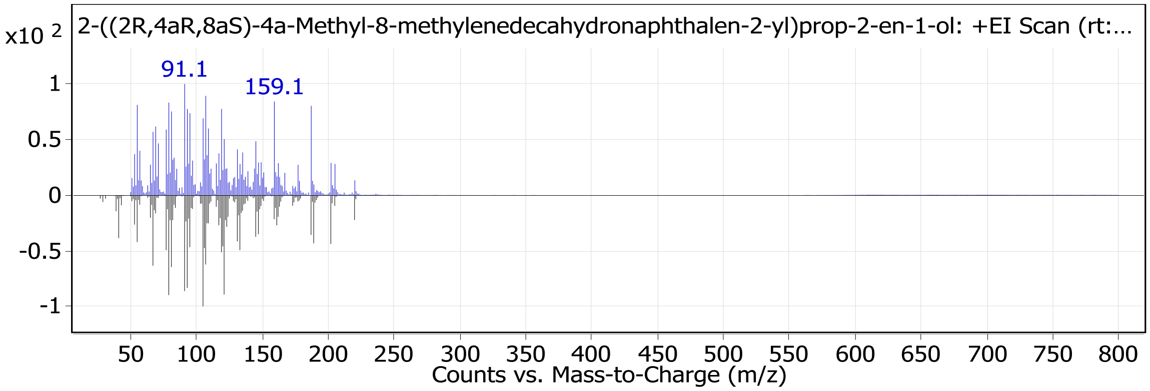

Spectrum Structure

2-((2R,4aR,8aS)-4a-Methyl-8-methylenedecahydronaphthalen-2-yl)prop-2-en-1-ol

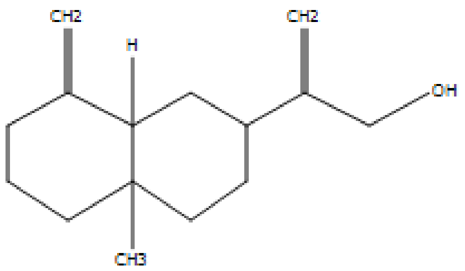

Spectrum Source	Collision Energy	Ionization Mode
Peak (28) in "+ TIC Scan"	0	EI

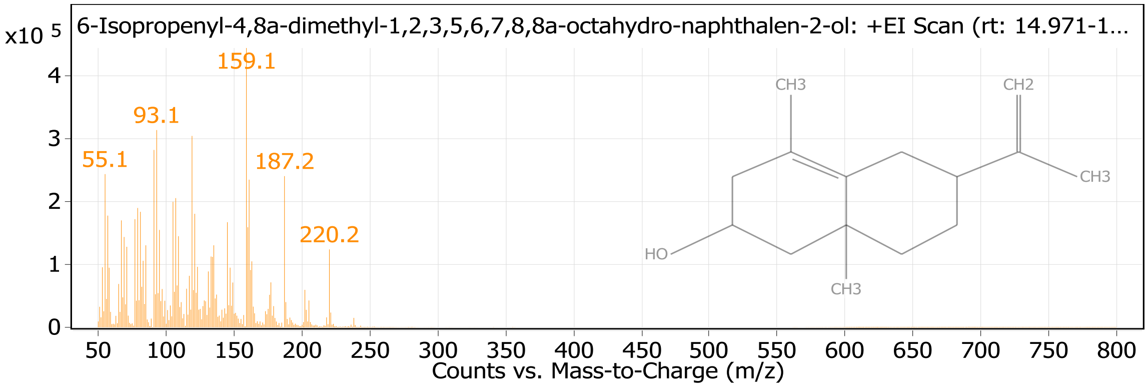

Qualitative Analysis Report

Library Spectrum

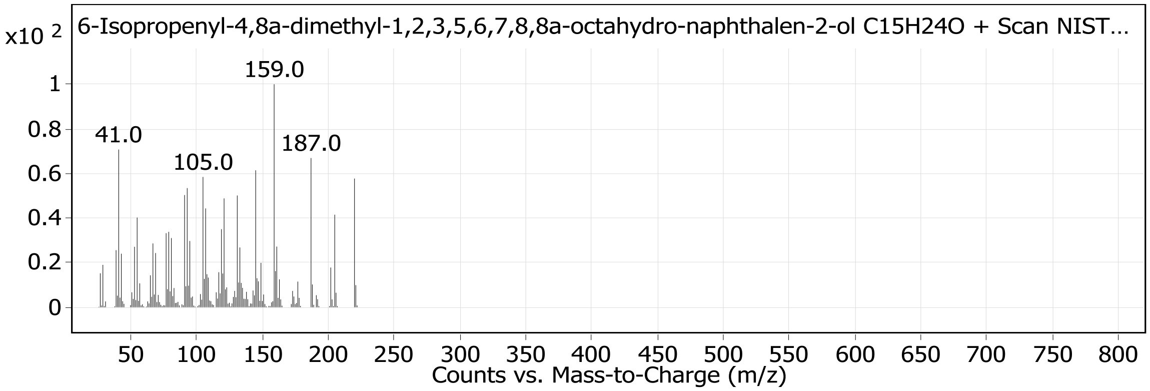

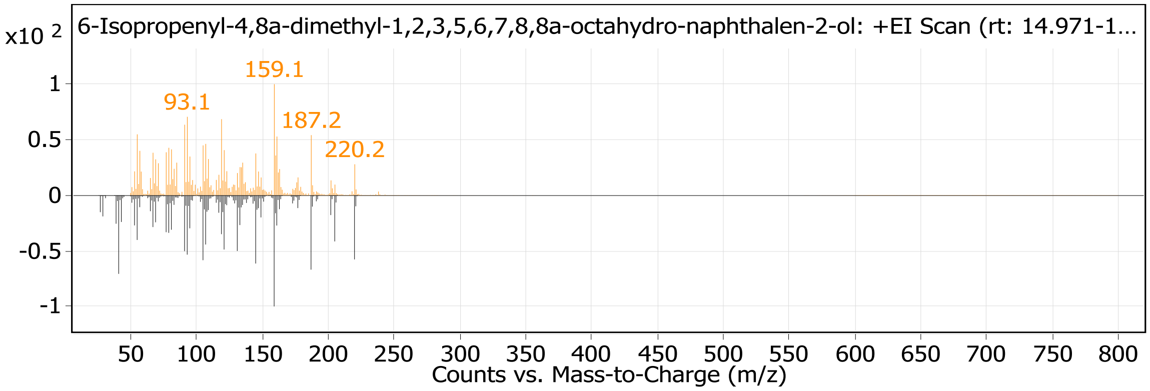

Spectrum Structure

6-Isopropenyl-4,8a-dimethyl-1,2,3,5,6,7,8,8a-octahydro-naphthalen-2-ol

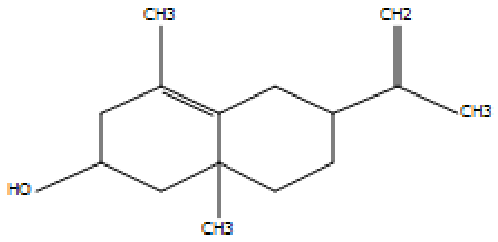

**Spectrum Source**	**Collision Energy**	**Ionization Mode**
Peak (29) in "+ TIC Scan"	0	EI

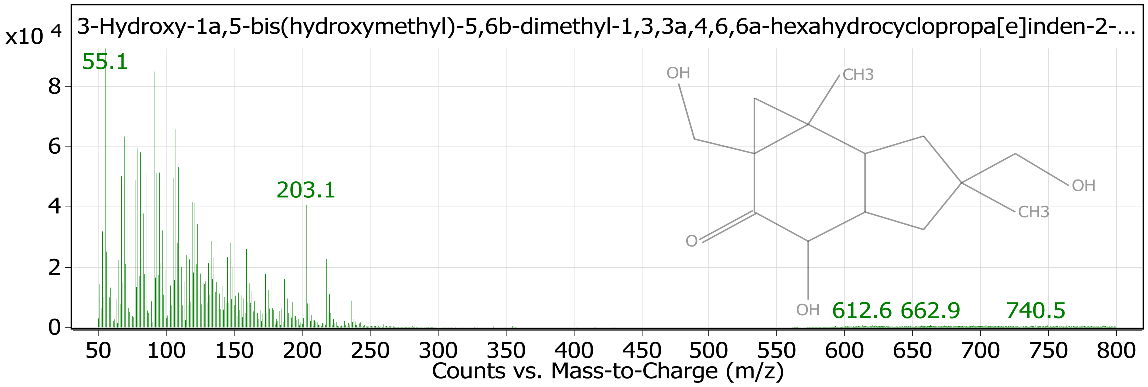

Library Spectrum

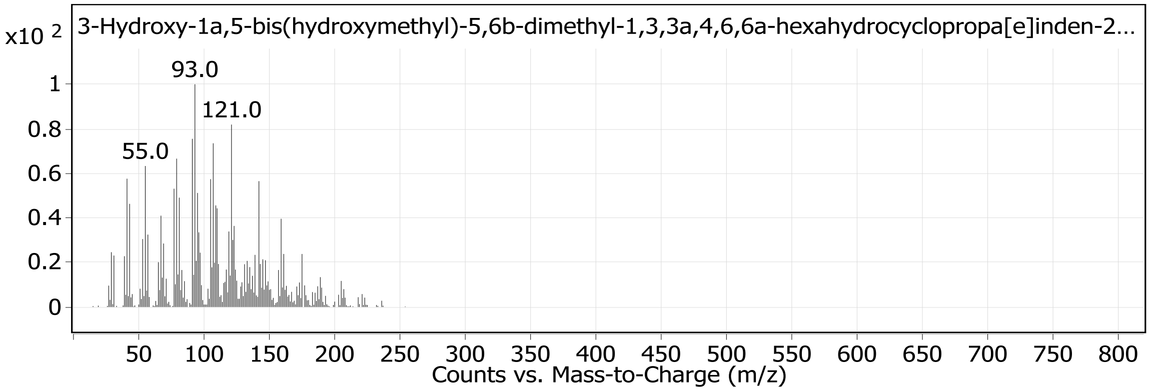

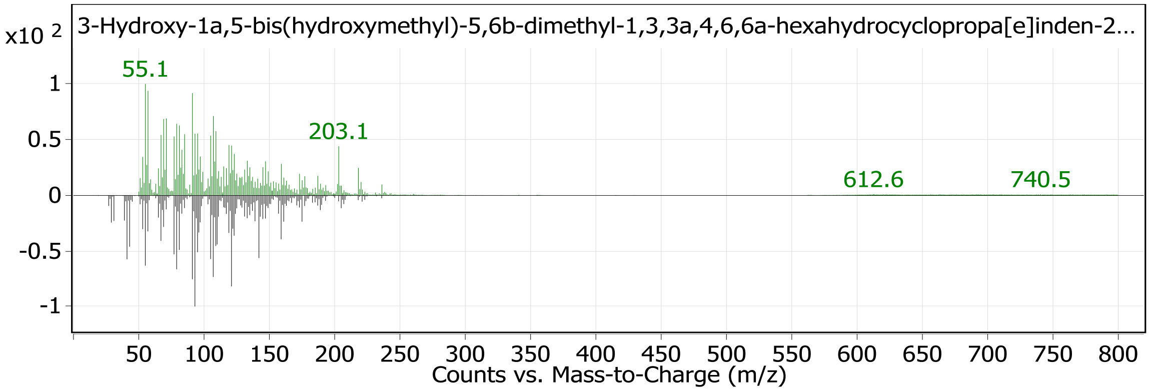

Spectrum Structure

3-Hydroxy-1a,5-bis(hydroxymethyl)-5,6b-dimethyl-1,3,3a,4,6,6a-hexahydrocyclopropa[e]inden-2-one

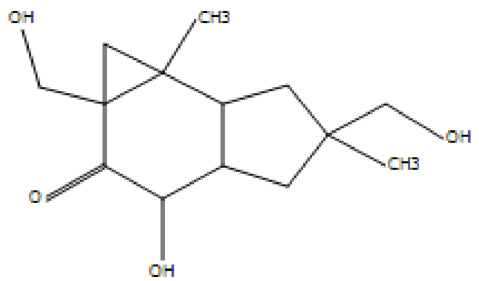

Spectrum Source	Collision Energy	Ionization Mode
Peak (30) in "+ TIC Scan"	0	EI

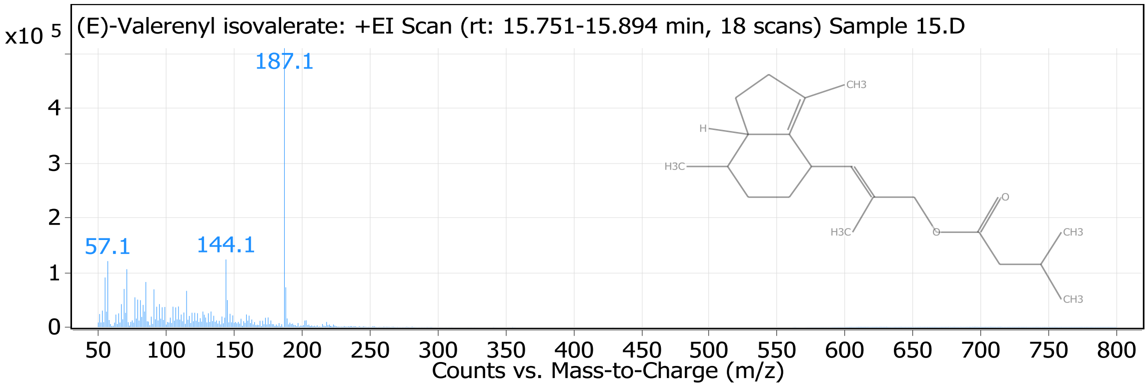

Library Spectrum

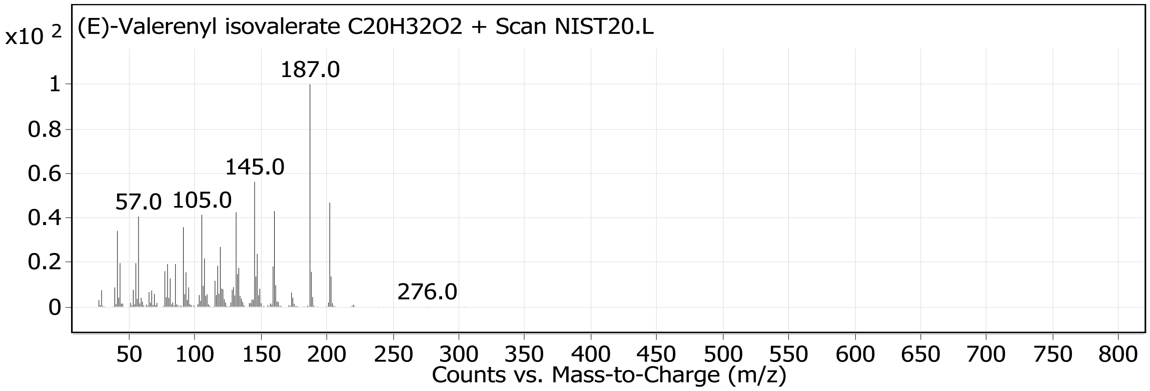

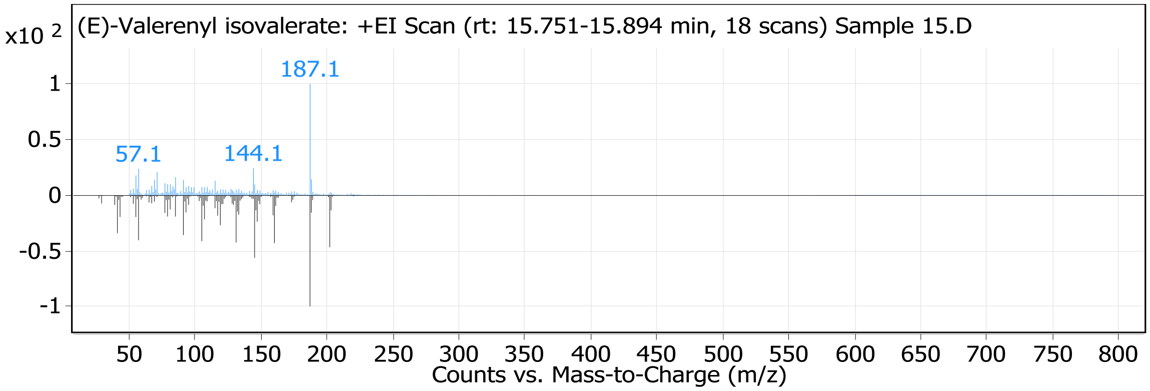

Spectrum Structure

(E)-Valerenyl isovalerate

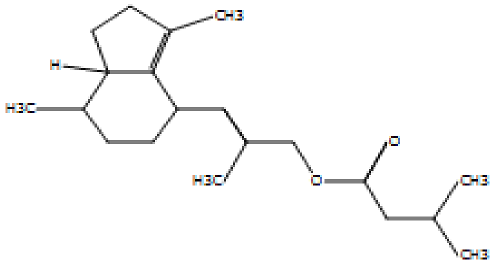

Spectrum Source	Collision Energy	Ionization Mode
Peak (31) in "+ TIC Scan"	0	EI

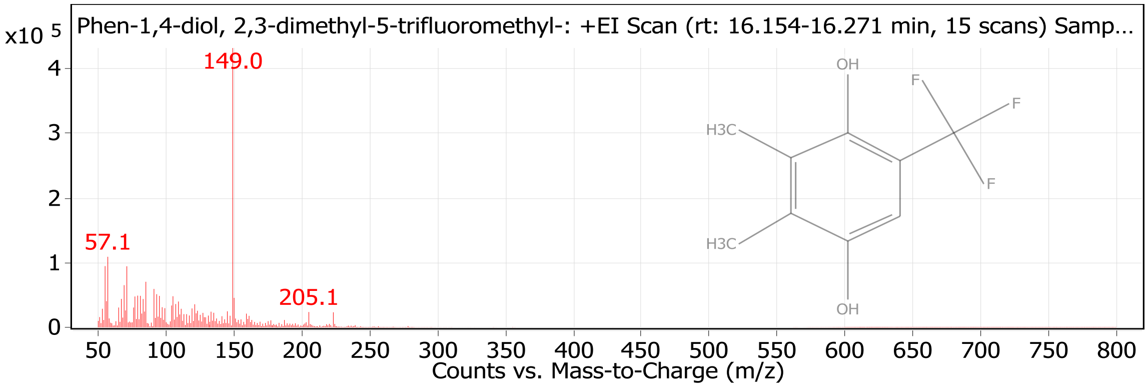

Library Spectrum

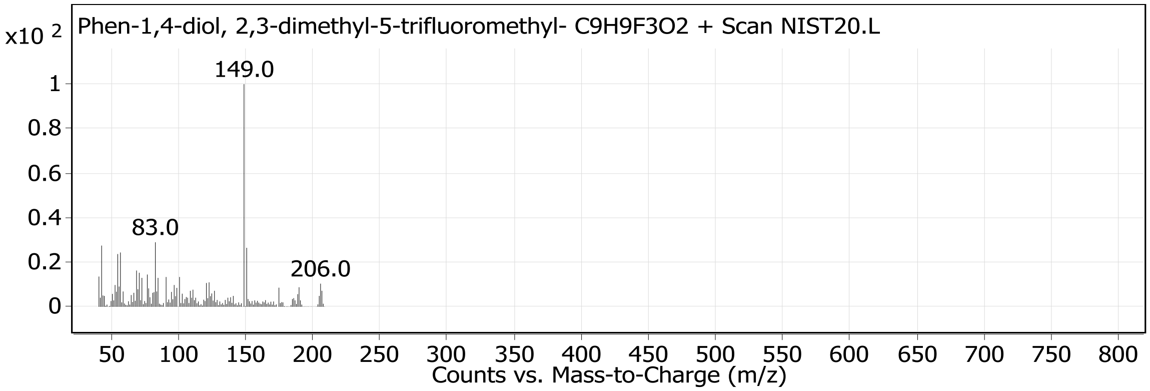

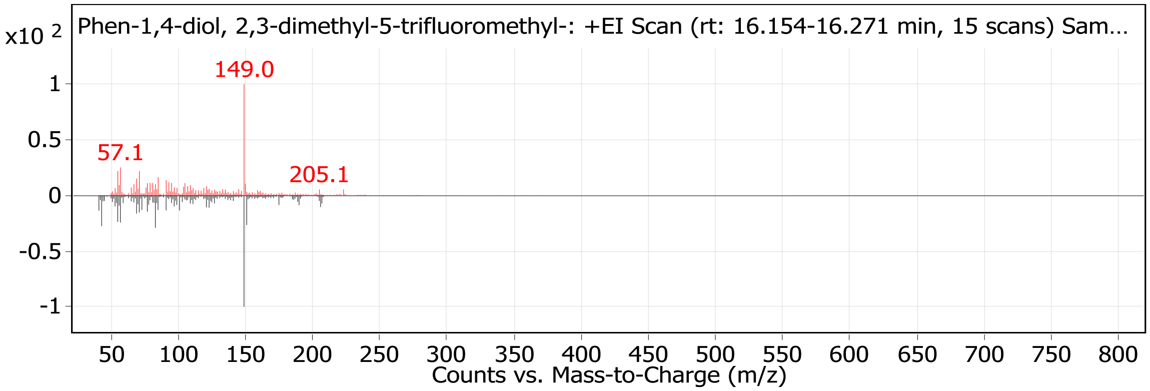

Qualitative Analysis Report

Spectrum Structure

Phen-1,4-diol, 2,3-dimethyl-5-trifluoromethyl-

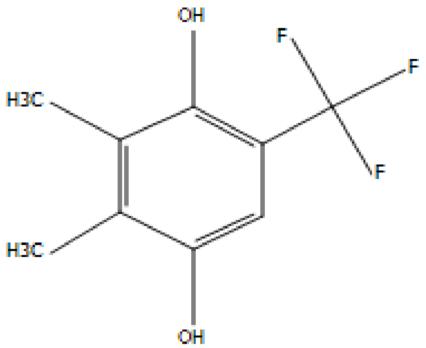

**Spectrum Source**	**Collision Energy**	**Ionization Mode**
Peak (32) in "+ TIC Scan"	0	EI

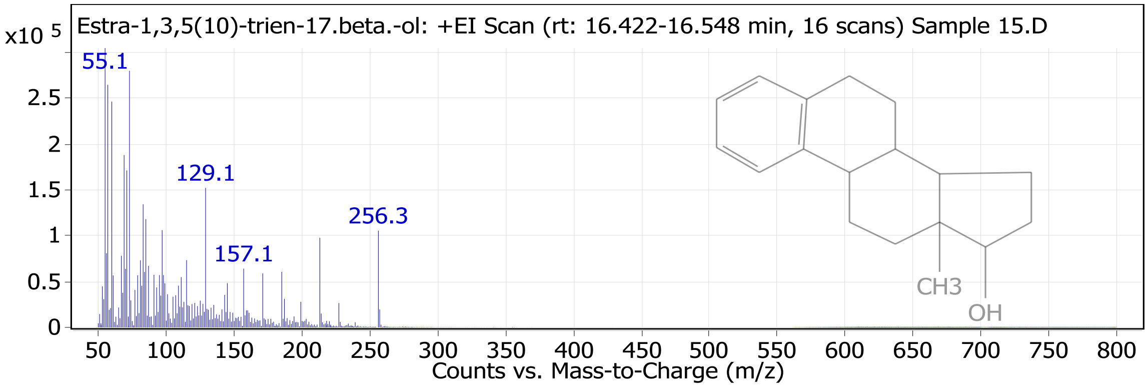

Library Spectrum

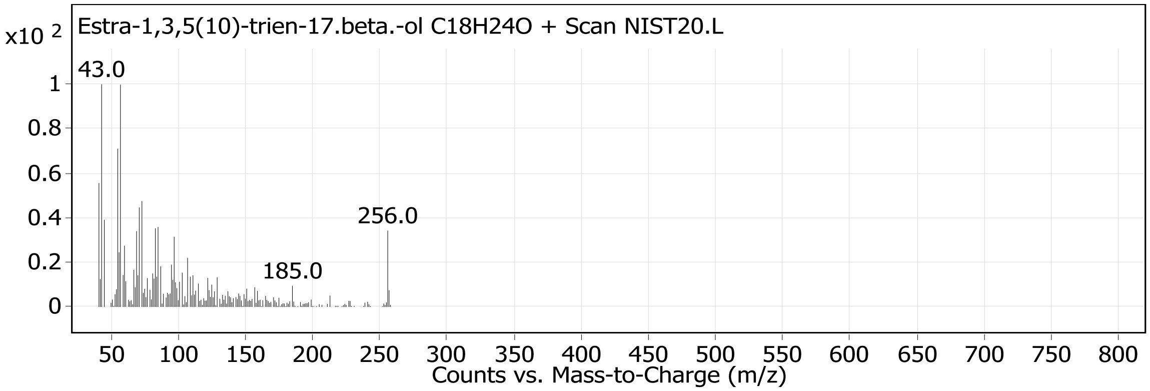

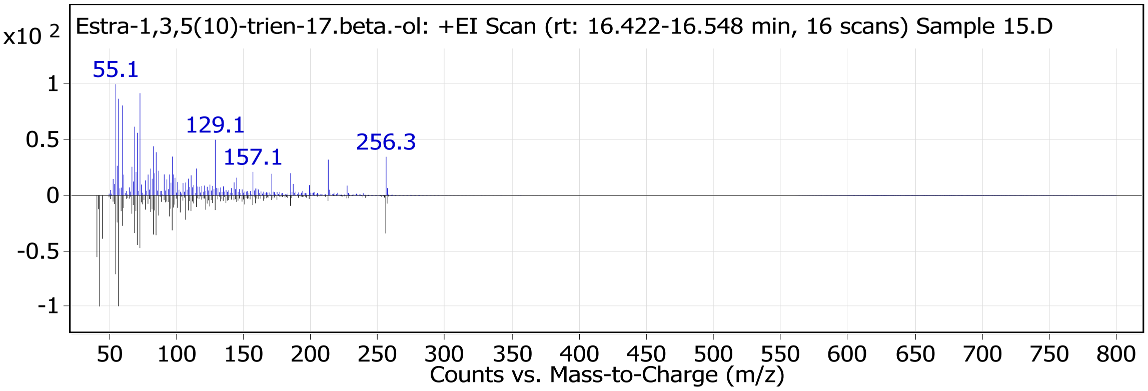

Spectrum Structure

Estra-1,3,5(10)-trien-17.beta.-ol

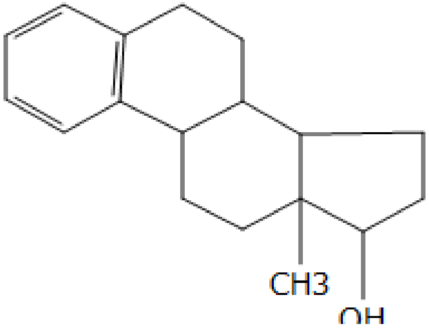

**Spectrum Source**	**Collision Energy**	**Ionization Mode**
Peak (33) in "+ TIC Scan"	0	EI

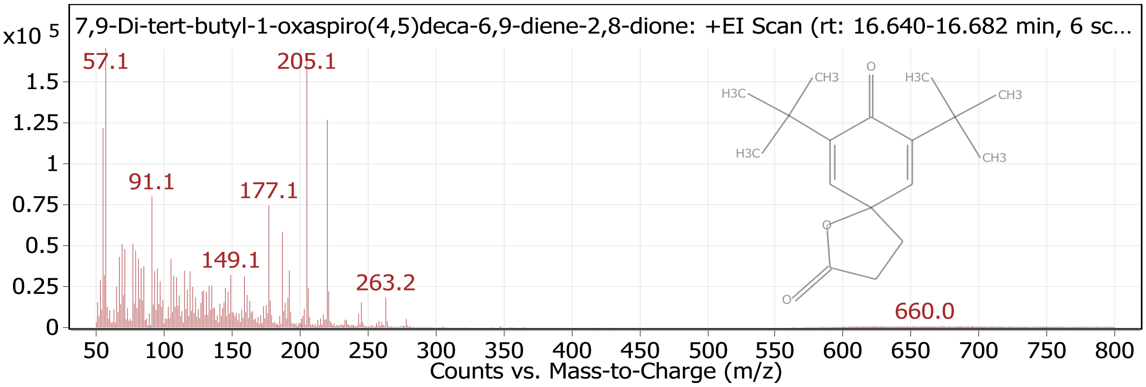

Library Spectrum

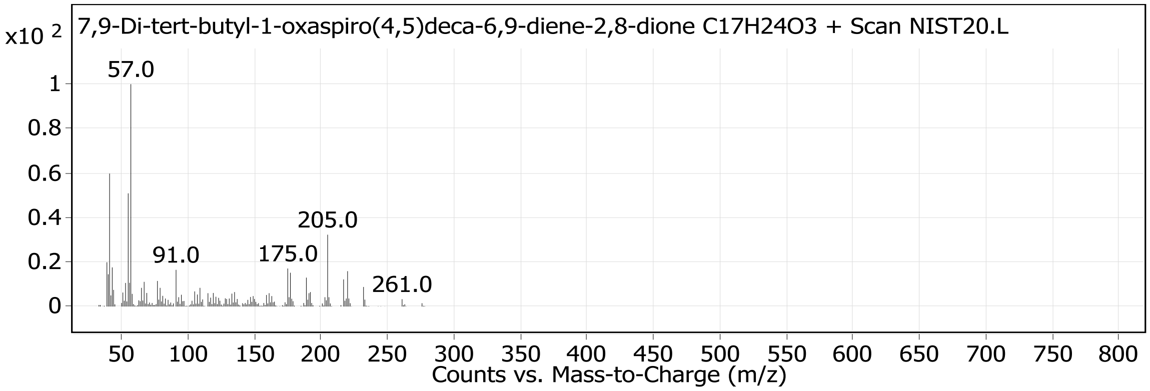

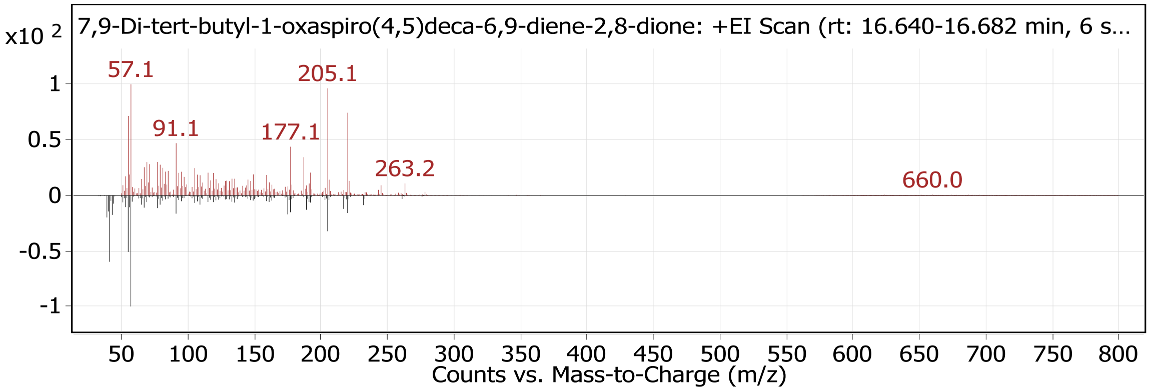

Spectrum Structure

7,9-Di-tert-butyl-1-oxaspiro(4,5)deca-6,9-diene-2,8-dione

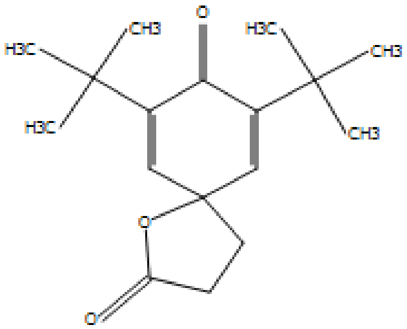

Spectrum Source	Collision Energy	Ionization Mode
Peak (34) in "+ TIC Scan"	0	EI

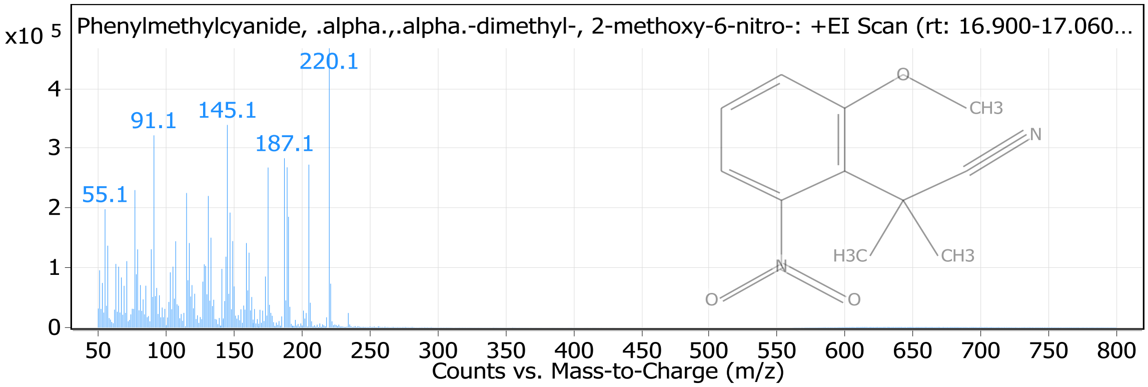

Library Spectrum

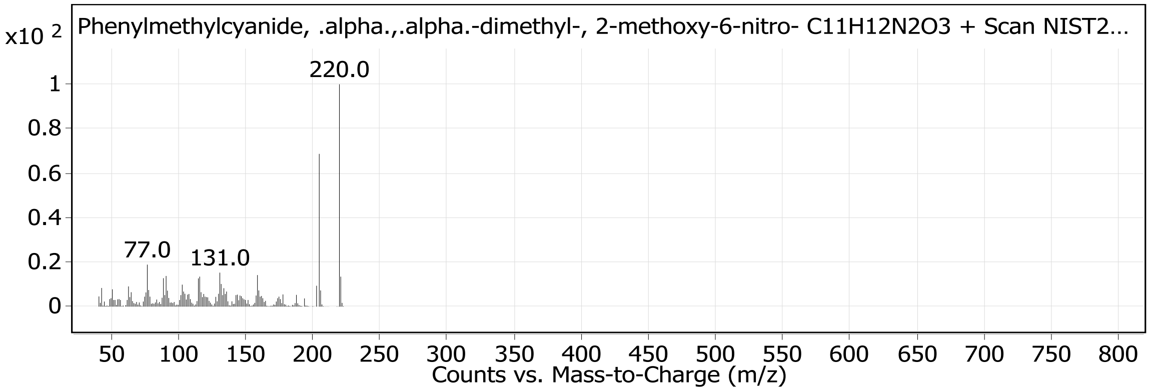

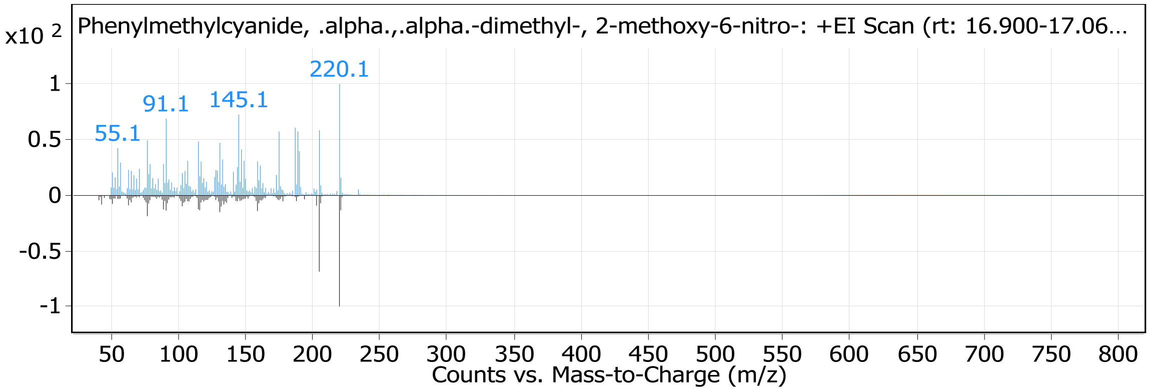

Spectrum Structure

Phenylmethylcyanide, .alpha.,.alpha.-dimethyl-, 2-methoxy-6-nitro-

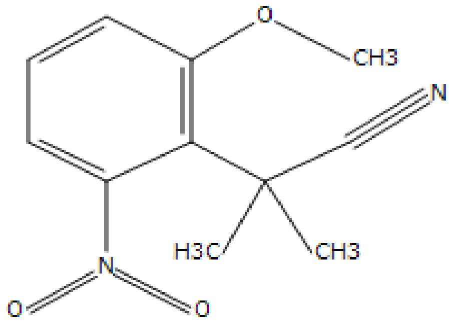

Spectrum Source	Collision Energy	Ionization Mode
Peak (35) in "+ TIC Scan"	0	EI

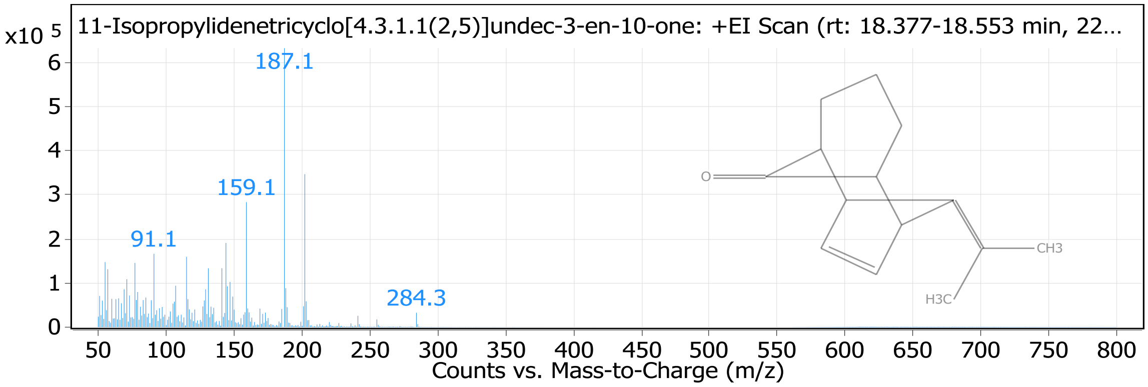

Library Spectrum

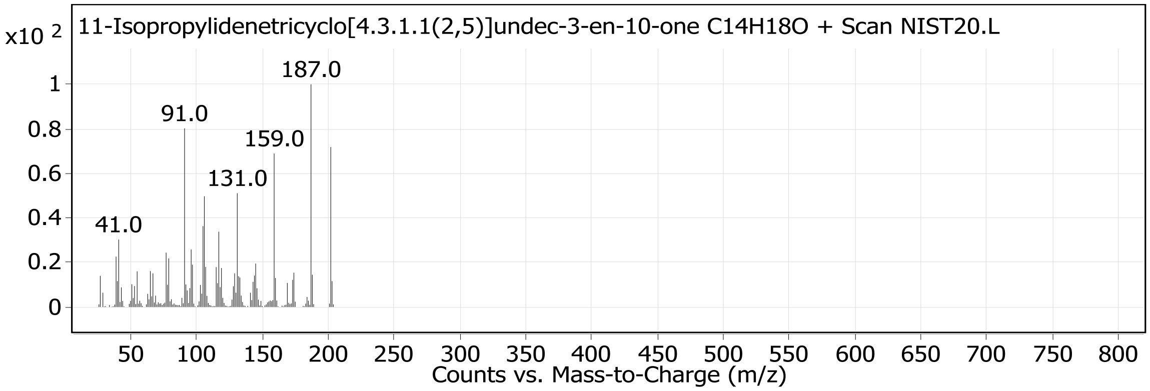

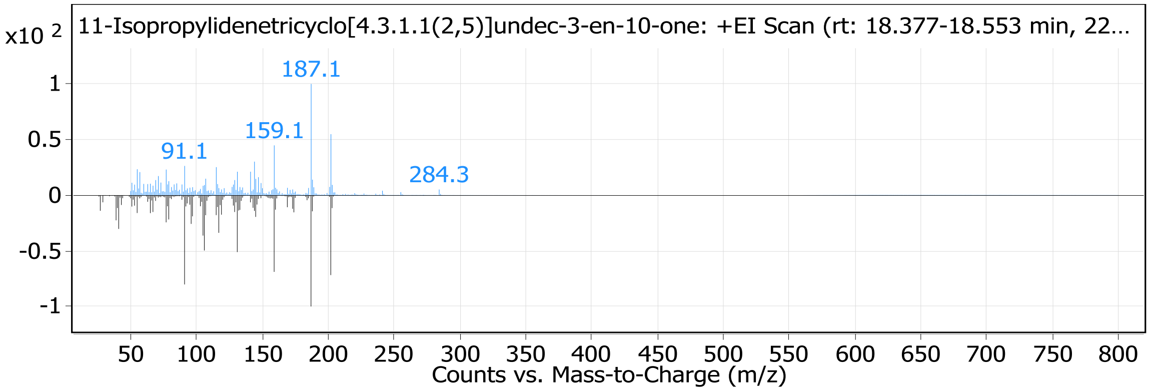

Spectrum Structure

11-Isopropylidenetricyclo[4.3.1.1(2,5)]undec-3-en-10-one

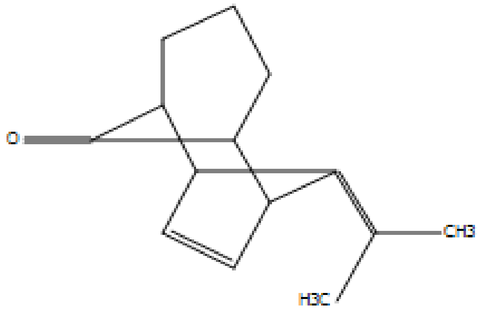

Spectrum Source	Collision Energy	Ionization Mode
Peak (36) in "+ TIC Scan"	0	EI

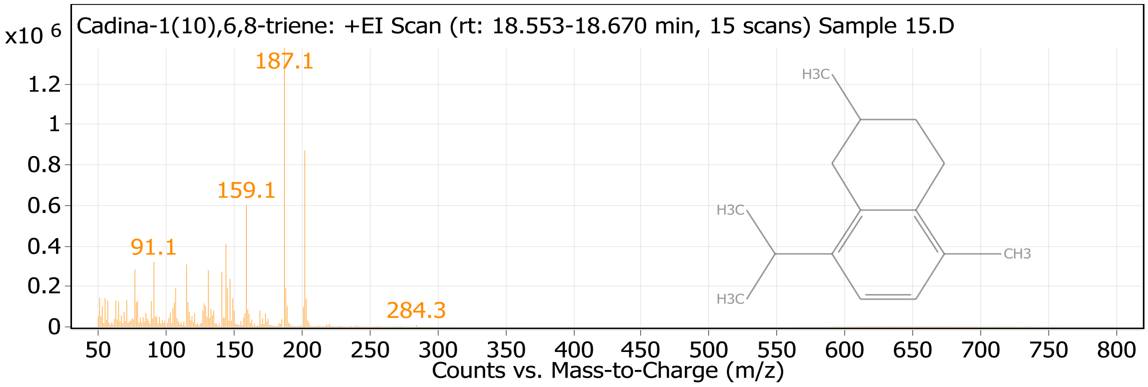

Library Spectrum

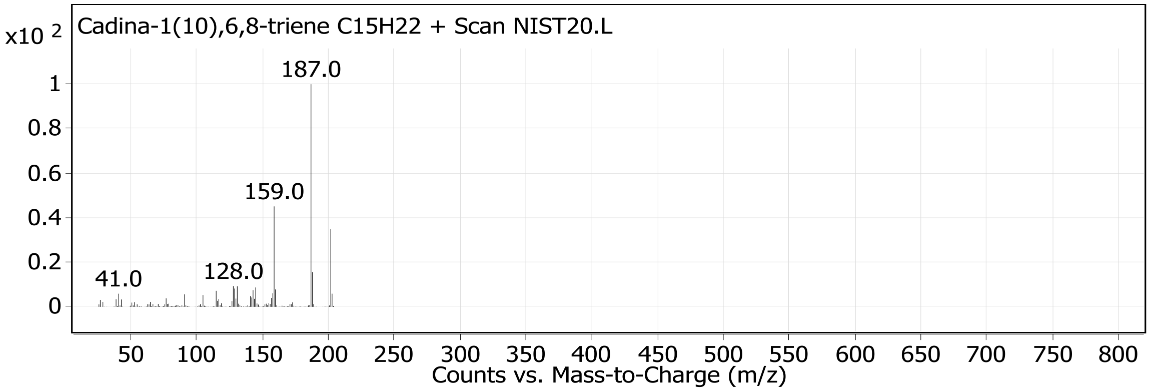

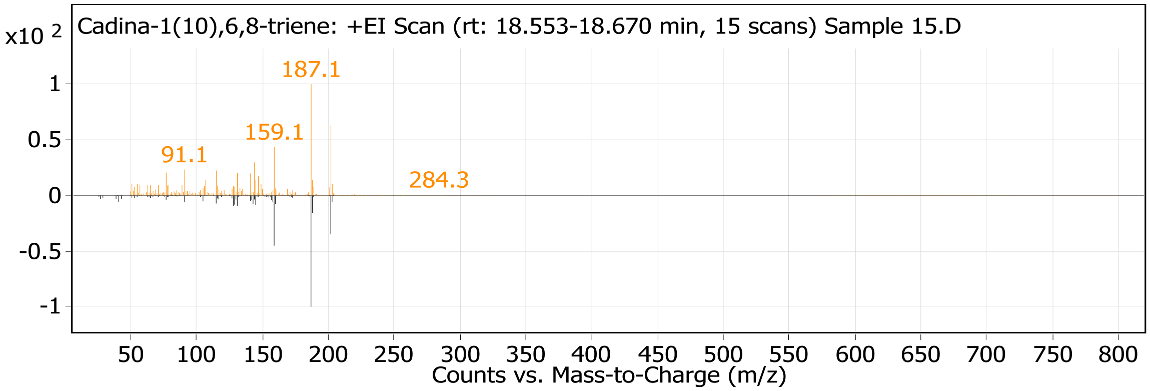

Qualitative Analysis Report

Spectrum Structure

Cadina-1(10),6,8-triene

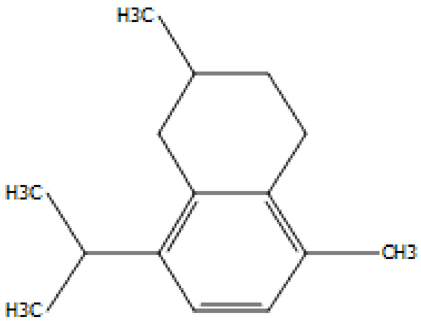

**Spectrum Source**	**Collision Energy**	**Ionization Mode**
Peak (37) in "+ TIC Scan"	0	EI

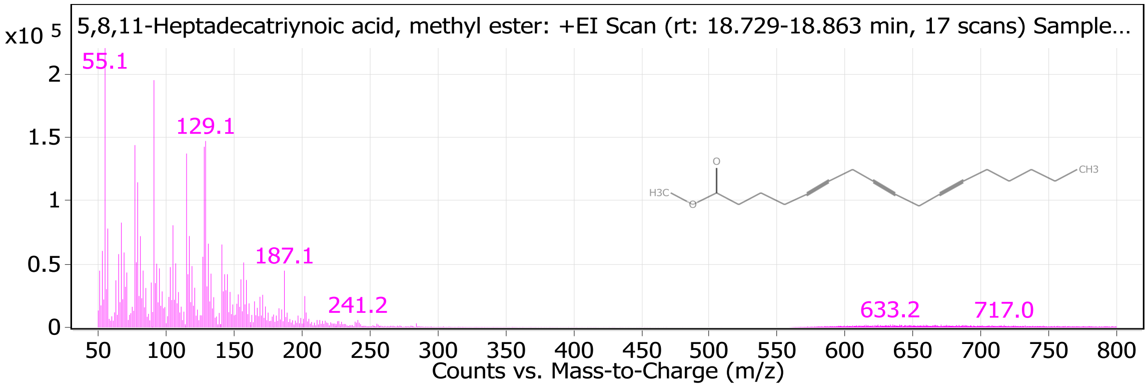

Library Spectrum

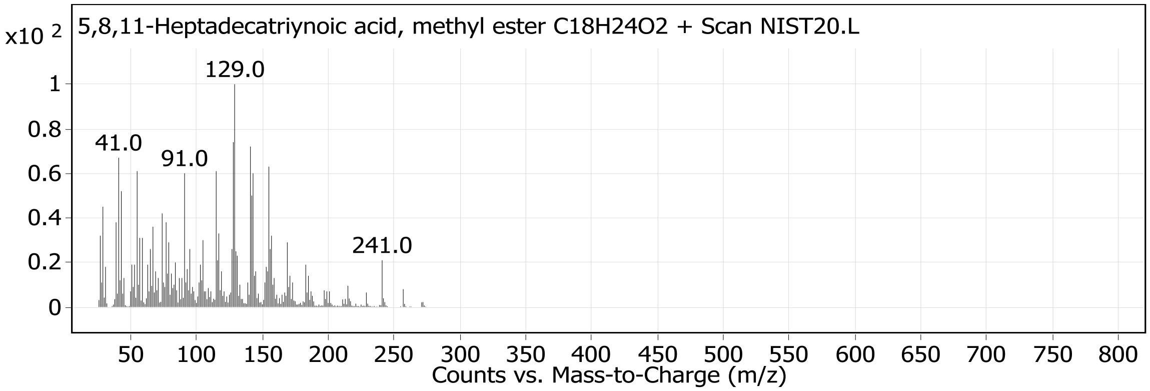

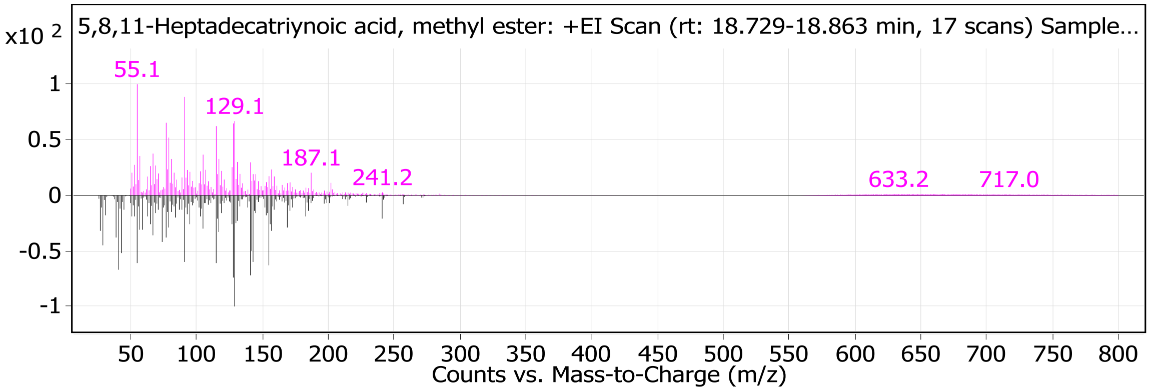
5,8,11-Heptadecatriynoic acid, methyl ester: +EI Scan (rt: 18.729-18.863 min, 17 scans) Sample...
x10 2
1
0.5
0
-0.5
-1
55.1
129.1
187.1
241.2
633.2
717.0
50
100
150
200
250
300
350
400
450
500
550
600
650
700
750
800
Counts vs. Mass-to-Charge (m/z)

Qualitative Analysis Report

Spectrum Structure

5,8,11-Heptadecatriynoic acid, methyl ester

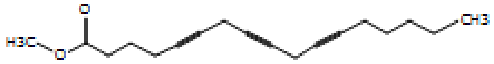

**Spectrum Source**	**Collision Energy**	**Ionization Mode**
Peak (38) in "+ TIC Scan"	0	EI

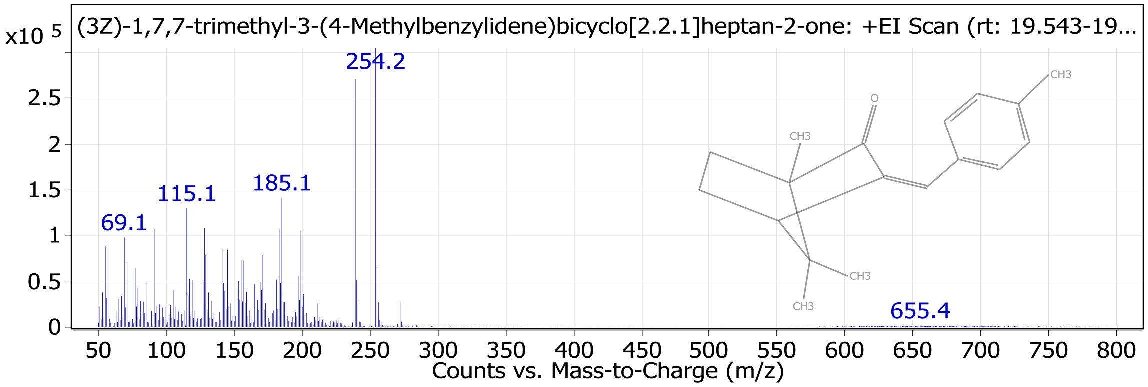

Library Spectrum

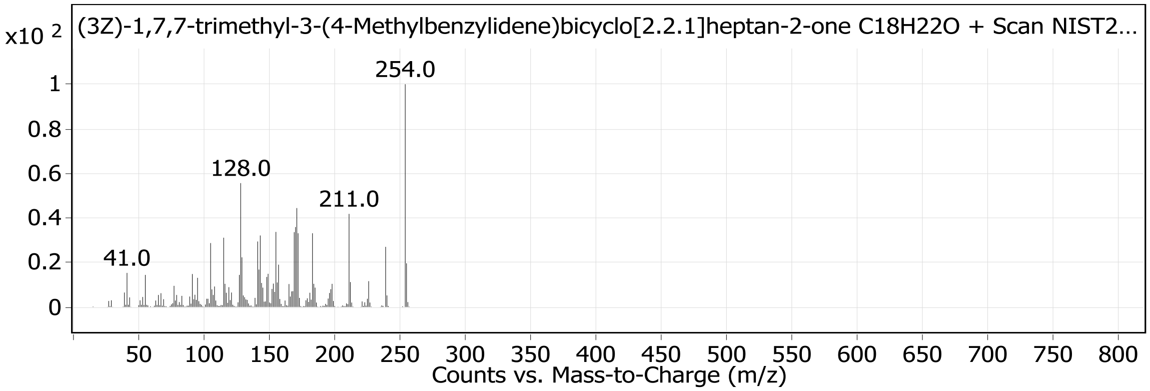

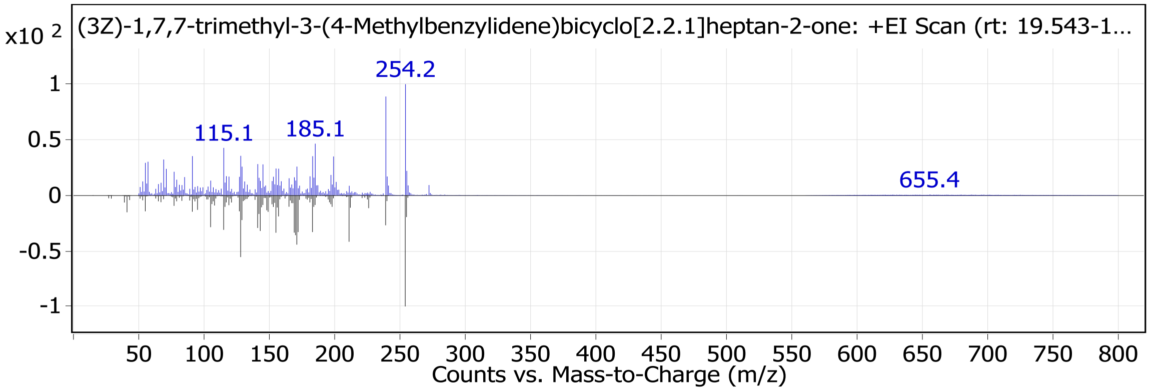

Spectrum Structure

(3Z)-1,7,7-trimethyl-3-(4-Methylbenzylidene)bicyclo[2.2.1]heptan-2-one

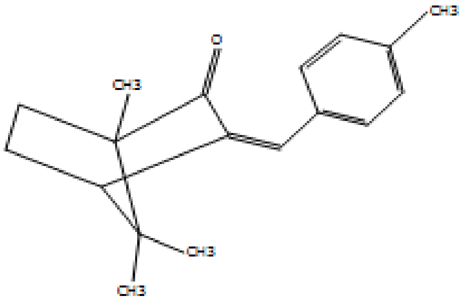

Spectrum Source	Collision Energy	Ionization Mode
Peak (39) in "+ TIC Scan"	0	EI

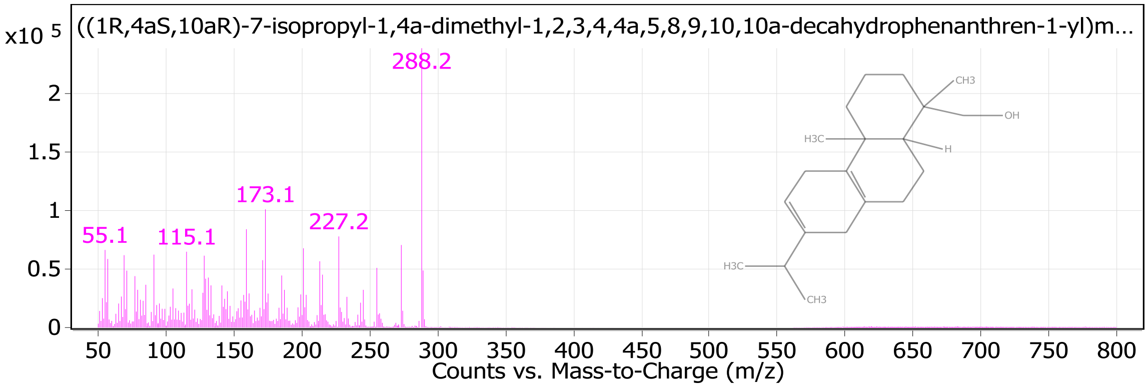

Library Spectrum

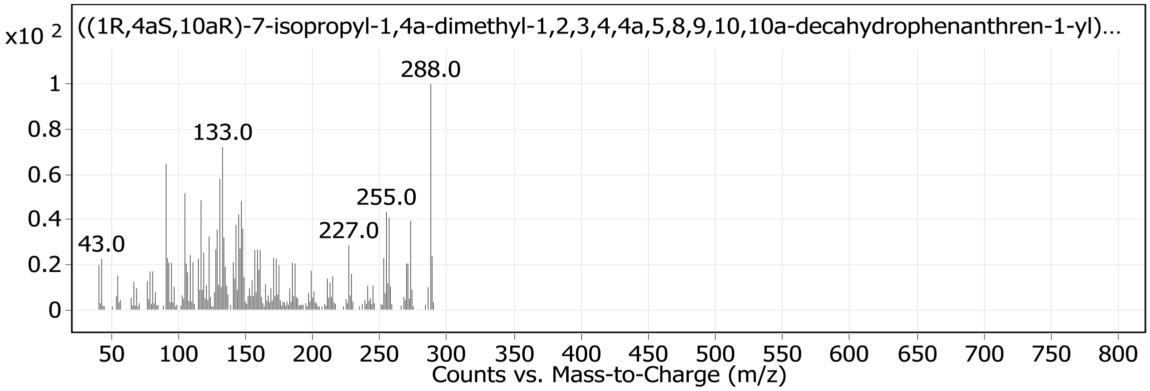

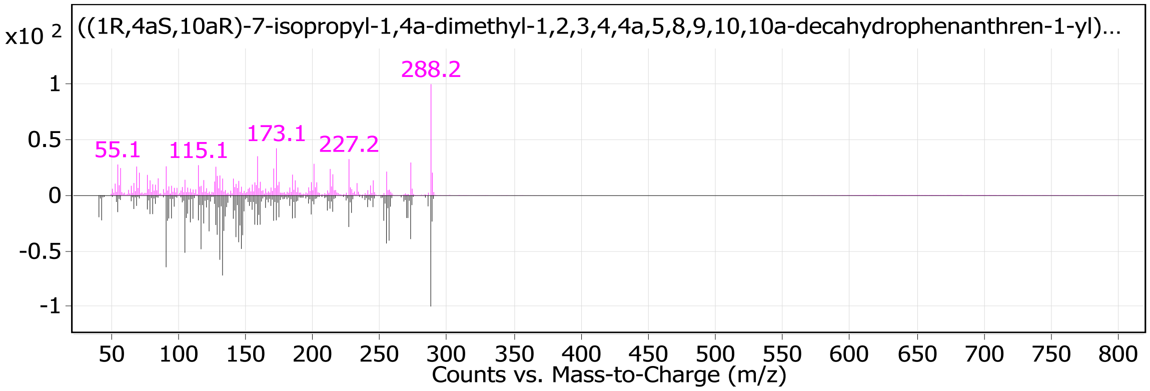

Spectrum Structure

((1R,4aS,10aR)-7-isopropyl-1,4a-dimethyl-1,2,3,4,4a,5,8,9,10,10a-decahydrophenanthren-1-yl)methanol

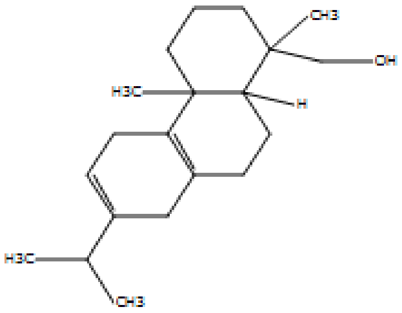

Spectrum Source	Collision Energy	Ionization Mode
Peak (40) in "+ TIC Scan"	0	EI

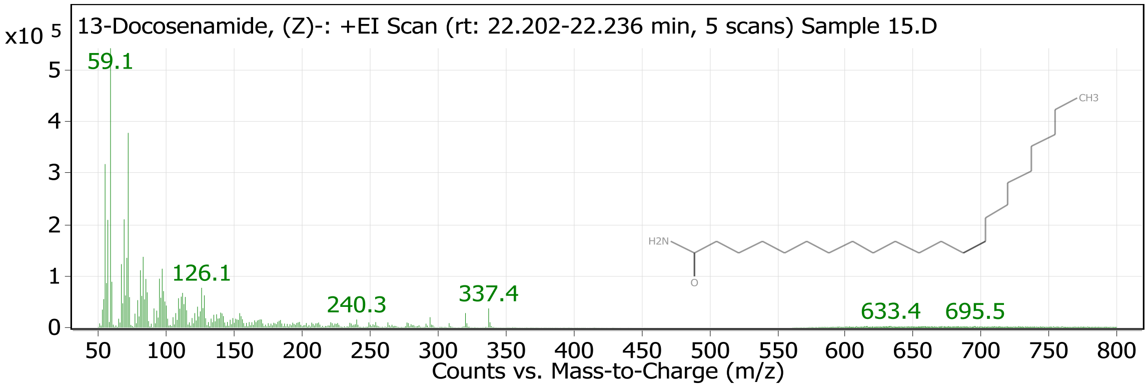

Library Spectrum

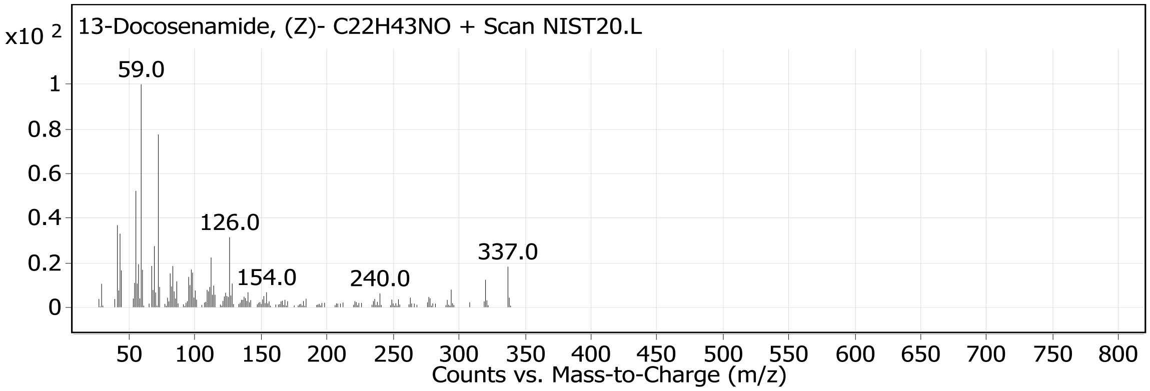

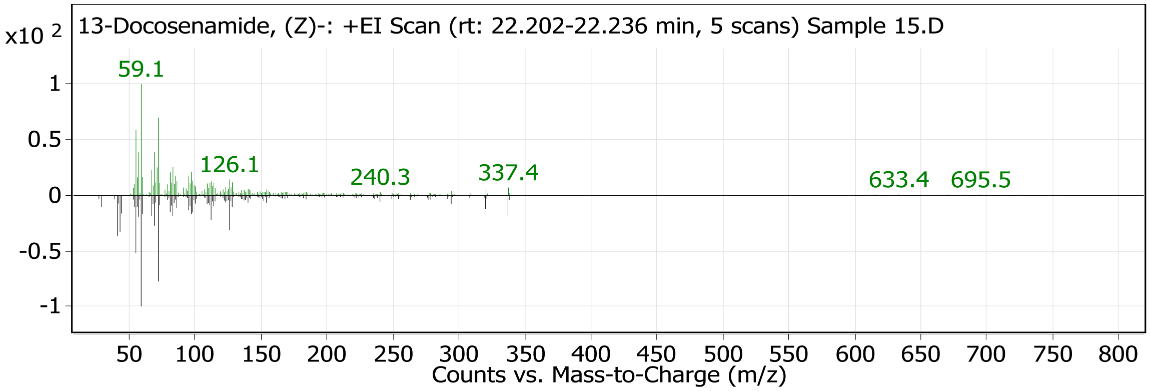

Spectrum Structure

13-Docosenamide, (Z)-

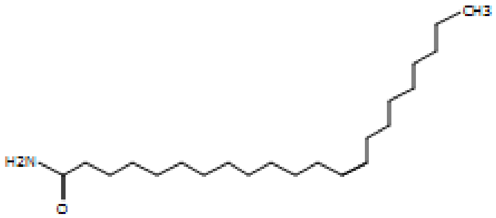

--- End Of Report ---
